# Supplementary material for: Synthesis of Four Orthogonally Protected Rare l-Hexose Thioglycosides from d-Mannose by C-5 and C-4 Epimerization
Source: Molecules. 2022 May 25;27(11):3422. doi: 10.3390/molecules27113422 (PMC9182441; doi:10.3390/molecules27113422)
Supplement: Supplementary file 1 [file molecules-27-03422-s001.zip › molecules-1734149-check CIF report.pdf]

checkCIF () running

Checking for embedded fcf data in CIF ...
Found embedded fcf data in CIF. Extracting fcf data from uploaded CIF, please wait **...**

checkCIF/PLATON (full publication check)

You have not supplied any structure factors. As a result the full set of tests cannot be run.

THIS REPORT IS FOR GUIDANCE ONLY. IF USED AS PART OF A REVIEW PROCEDURE FOR PUBLICATION, IT SHOULD NOT REPLACE THE EXPERTISE OF AN EXPERIENCED CRYSTALLOGRAPHIC REFEREE.You have not supplied any structure factors. As a result the full set of tests cannot be run.

No syntax errors found. CIF dictionary
Please wait while processing .... Interpreting this report
Structure factor report

Datablock: I

Bond precision: C-C = 0.0327 A Wavelength=0.71073

Cell:	a=21.29(2)	b=5.411(4)	c=26.79(2)
	alpha=90	beta=95.97(4)	gamma=90
Temperature:	199 K		

	Calculated	Reported
Volume	3070(4)	3069(5)
Space group	C 2	C 1 2 1
Hall group	C 2y	C 2y
Moiety formula	C37 H34 O5 S	C37 H34 O5 S
Sum formula	C37 H34 O5 S	C37 H34 O5 S
Mr	590.70	590.70
Dx,g cm-3	1.278	1.278
Z	4	4
Mu (mm-1)	0.149	0.149
F000	1248.0	1248.0
F000'	1249.06	
h,k,lmax	21,5,27	21,5,27
Nref	3556[2013]	3399
Tmin,Tmax	0.987,0.993	0.630,0.990
Tmin'	0.949	

Correction method= # Reported T Limits: Tmin=0.630 Tmax=0.990 AbsCorr = MULTI-SCAN

Data completeness= 1.69/0.96 Theta(max)= 21.520

R(reflections)= 0.0979(1822) wR2(reflections)= 0.2897(3399)

S = 1.052 Npar= 389

The following ALERTS were generated. Each ALERT has the format
test-name_ALERT_alert-type_alert-level.
Click on the hyperlinks for more details of the test.

Alert level A

THETM01_ALERT_3_A The value of sine(theta_max)/wavelength is less than 0.550
Calculated sin(theta_max)/wavelength = 0.5161

Author Response: All crystals were weakly diffracting.

PLAT023_ALERT_3_A	Resolution (too) Low [sin(theta)/Lambda < 0.6]..	0.52	Ang-1

Author Response: Also related to the fact that all crystals were weakly diffracting. The structure is considered to be correct.

PLAT331_ALERT_2_A	Small Aver Phenyl C-C Dist C31 --C36 .	1.33	Ang.

Author Response: Chemical evidence supports that the structure is correct.

Alert level B

PLAT089_ALERT_3_B	Poor Data / Parameter Ratio (Zmax < 18)	5.17	Note
PLAT242_ALERT_2_B	Low 'MainMol' Ueq as Compared to Neighbors of	C31	Check
PLAT340_ALERT_3_B	Low Bond Precision on C-C Bonds	0.0327	Ang.

Alert level C

RINTA01_ALERT_3_C	The value of Rint is greater than 0.12		
	Rint given 0.164		
PLAT020_ALERT_3_C	The Value of Rint is Greater Than 0.12	0.164	Report
PLAT029_ALERT_3_C	_diffrn_measured_fraction_theta_full value Low .	0.971	Why?
PLAT084_ALERT_3_C	High wR2 Value (i.e. > 0.25)	0.29	Report
PLAT220_ALERT_2_C	NonSolvent Resd 1 C Ueq(max)/Ueq(min) Range	3.4	Ratio
PLAT241_ALERT_2_C	High 'MainMol' Ueq as Compared to Neighbors of	C20	Check

And 4 other PLAT241 Alerts

PLAT241_ALERT_2_C	High 'MainMol' Ueq as Compared to Neighbors of	C32	Check
PLAT241_ALERT_2_C	High 'MainMol' Ueq as Compared to Neighbors of	C33	Check
PLAT241_ALERT_2_C	High 'MainMol' Ueq as Compared to Neighbors of	C36	Check
PLAT241_ALERT_2_C	High 'MainMol' Ueq as Compared to Neighbors of	C45	Check
PLAT242_ALERT_2_C	Low 'MainMol' Ueq as Compared to Neighbors of	C34	Check
PLAT331_ALERT_2_C	Small Aver Phenyl C-C Dist C21 --C26 .	1.37	Ang.

Author Response: Chemical evidence supports that the structure is correct.

PLAT332_ALERT_2_C	Large Phenyl C-C Range C31 -C36 .	0.22	Ang.
PLAT334_ALERT_2_C	Small Aver. Benzene C-C Dist C44A -C48A	1.36	Ang.
PLAT368_ALERT_2_C	Short C(sp2)-C(sp2) Bond C34 - C35 .	1.22	Ang.

Alert level G

PLAT072_ALERT_2_G	SHELXL First Parameter in WGHT Unusually Large	0.13	Report
PLAT187_ALERT_4_G	The CIF-Embedded .res File Contains RIGU Records	1	Report
PLAT335_ALERT_2_G	Check Large C6 Ring C-C Range C41 -C48A	0.16	Ang.
PLAT335_ALERT_2_G	Check Large C6 Ring C-C Range C44A -C48A	0.16	Ang.
PLAT398_ALERT_2_G	Deviating C-O-C Angle From 120 for O4 .	109.3	Degree
PLAT398_ALERT_2_G	Deviating C-O-C Angle From 120 for O60 .	109.5	Degree
PLAT720_ALERT_4_G	Number of Unusual/Non-Standard Labels	1	Note
PLAT791_ALERT_4_G	Model has Chirality at C1 (Sohnke SpGr)	R	Verify

And 5 other PLAT791 Alerts

PLAT791_ALERT_4_G	Model has Chirality at C2 (Sohnke SpGr)	S	Verify
PLAT791_ALERT_4_G	Model has Chirality at C3 (Sohnke SpGr)	S	Verify
PLAT791_ALERT_4_G	Model has Chirality at C4 (Sohnke SpGr)	S	Verify
PLAT791_ALERT_4_G	Model has Chirality at C5 (Sohnke SpGr)	S	Verify
PLAT791_ALERT_4_G	Model has Chirality at C40 (Sohnke SpGr)	R	Verify
PLAT860_ALERT_3_G	Number of Least-Squares Restraints	343	Note
PLAT933_ALERT_2_G	Number of HKL-OMIT Records in Embedded .res File	13	Note

3 **ALERT level A** = Most likely a serious problem - resolve or explain
3 **ALERT level B** = A potentially serious problem, consider carefully
15 **ALERT level C** = Check. Ensure it is not caused by an omission or oversight
15 **ALERT level G** = General information/check it is not something unexpected

```
 0 ALERT type 1 CIF construction/syntax error, inconsistent or missing data
19 ALERT type 2 Indicator that the structure model may be wrong or deficient
 9 ALERT type 3 Indicator that the structure quality may be low
 8 ALERT type 4 Improvement, methodology, query or suggestion
 0 ALERT type 5 Informative message, check
```

checkCIF publication errors

Alert level A

```
PUBL006_ALERT_1_A   _publ_requested_journal is missing
            e.g. 'Acta Crystallographica Section C'
```

Author Response: Organic Chemistry Frontiers

```
 1 ALERT level A = Data missing that is essential or data in wrong format
 0 ALERT level G = General alerts. Data that may be required is missing
```

Publication of your CIF

You should attempt to resolve as many as possible of the alerts in all categories. Often the minor alerts point to easily fixed oversights, errors and omissions in your CIF or refinement strategy, so attention to these fine details can be worthwhile. In order to resolve some of the more serious problems it may be necessary to carry out additional measurements or structure refinements. However, the nature of your study may justify the reported deviations from journal submission requirements and the more serious of these should be commented upon in the discussion or experimental section of a paper or in the "special_details" fields of the CIF. *checkCIF* was carefully designed to identify outliers and unusual parameters, but every test has its limitations and alerts that are not important in a particular case may appear. Conversely, the absence of alerts does not guarantee there are no aspects of the results needing attention. It is up to the individual to critically assess their own results and, if necessary, seek expert advice.

If you wish to submit your CIF for publication in Acta Crystallographica Section C or E, you should upload your CIF via the web. If you wish to submit your CIF for publication in IUCrData, you should upload your CIF via the web. If your CIF is to form part of a submission to another IUCr journal, you will be asked, either during electronic submission or by the Co-editor handling your paper, to upload your CIF via our web site.

PLATON version of 19/02/2022; check.def file version of 19/01/2022

Datablock I - ellipsoid plot

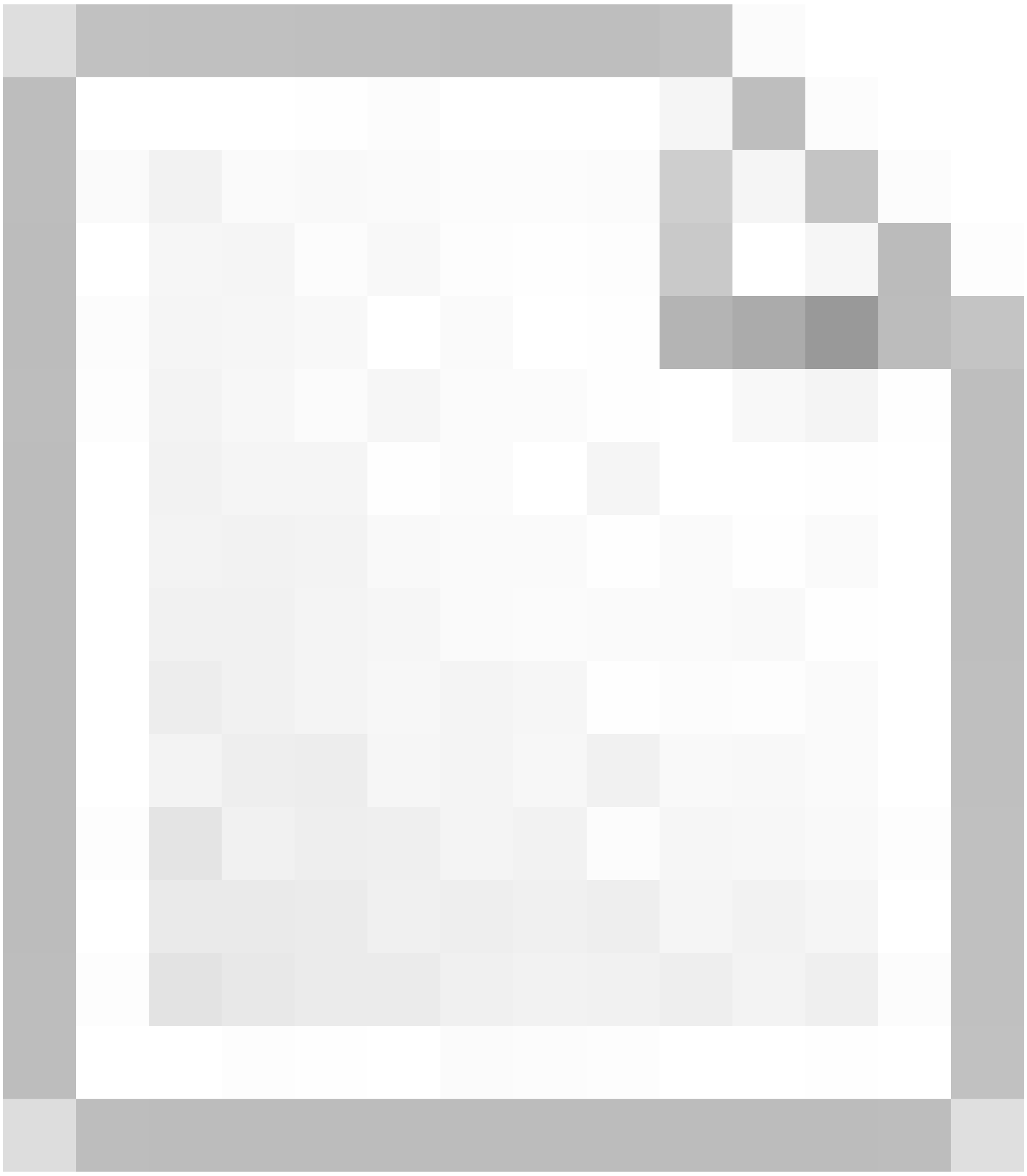

Download CIF editor (publCIF) from the IUCr
Download CIF editor (enCIFer) from the CCDC
Test a new CIF entry
