# Supplementary material for: Synthesis of Four Orthogonally Protected Rare l-Hexose Thioglycosides from d-Mannose by C-5 and C-4 Epimerization
Source: Molecules. 2022 May 25;27(11):3422. doi: 10.3390/molecules27113422 (PMC9182441; doi:10.3390/molecules27113422)
Supplement: Supplementary file 1 [file molecules-27-03422-s001.zip › molecules-1734149-supplementary.pdf]

Supporting Information

Synthesis of four orthogonally protected rare L-hexose thioglycosides from D-mannose by C-5 and C-4 epimerization

Fruzsina Demeter [1], Ilona Bereczki [1], Anikó Borbás [1*] and Mihály Herczeg [1,2*]

[1]*Department of Pharmaceutical Chemistry, University of Debrecen, Egyetem tér 1, H-4032 Debrecen, Hungary.*

[2]*Research Group for Oligosaccharide Chemistry of Hungarian Academy of Sciences, ELKH, Egyetem tér 1, H-4032 Debrecen, Hungary.*

E-mail: borbas.aniko@pharm.unideb.hu; herczeg.mihaly@pharm.unideb.hu

Table of Contents

^{1}H and ^{13}C NMR spectra of the synthesized compounds **1**

X-ray structure analysis of compound 83 **129**

^{1}H and ^{13}C NMR spectra of compound **24**

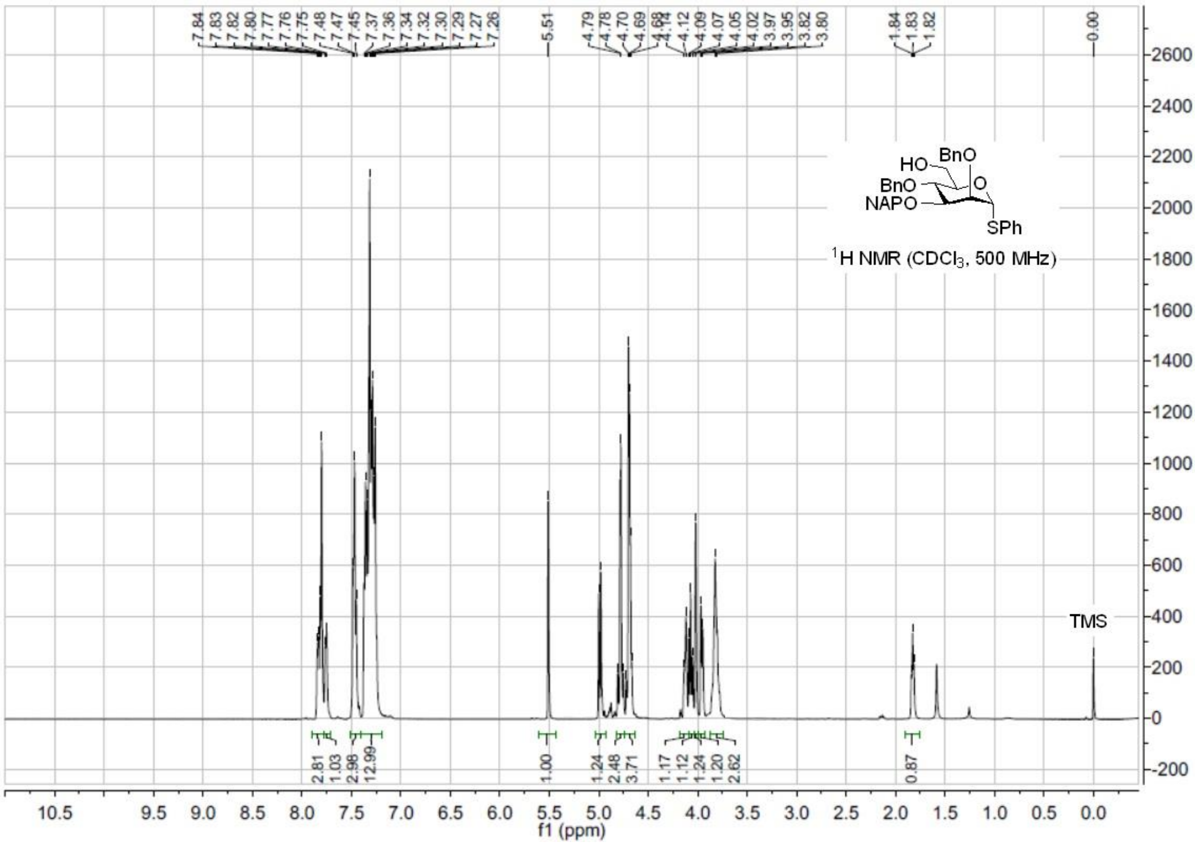

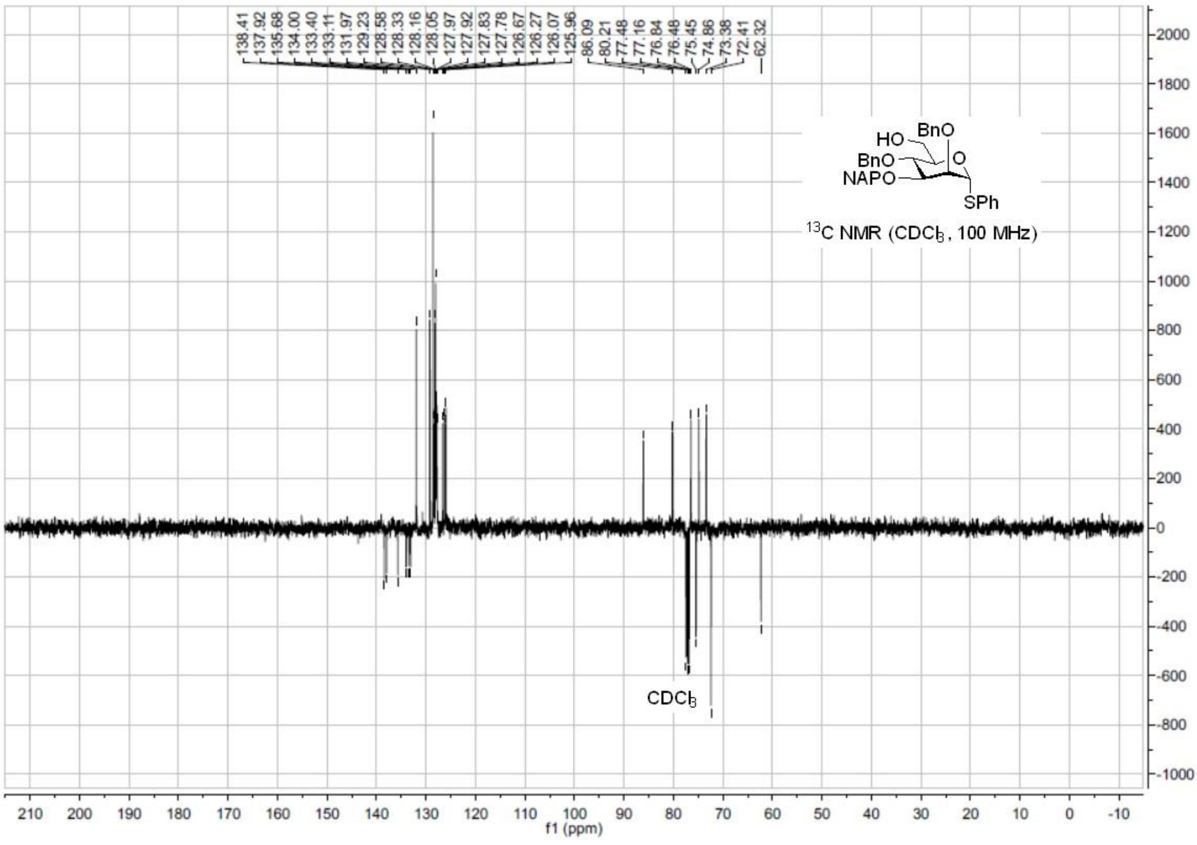

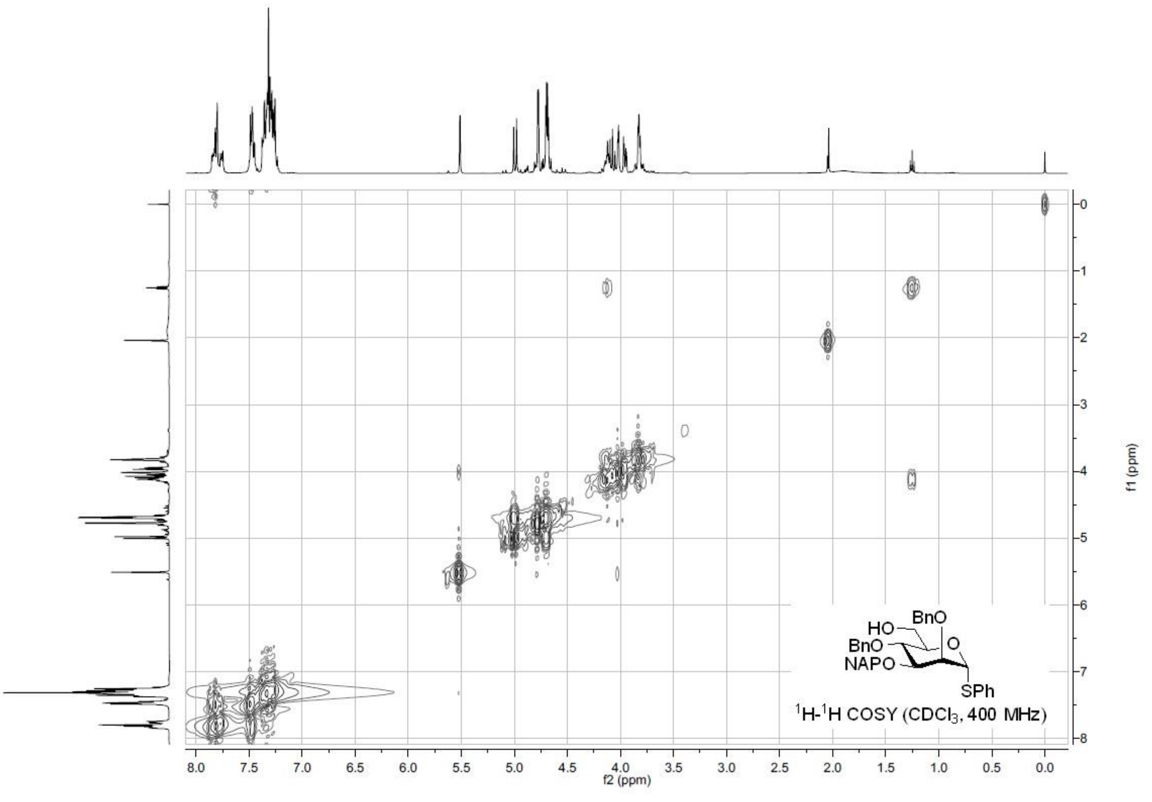
HO
BnO
BnO
NAPO
O
SPh
^{1}H-^{1}H COSY (CDCl$_3$, 400 MHz)
f2 (ppm)
f1 (ppm)

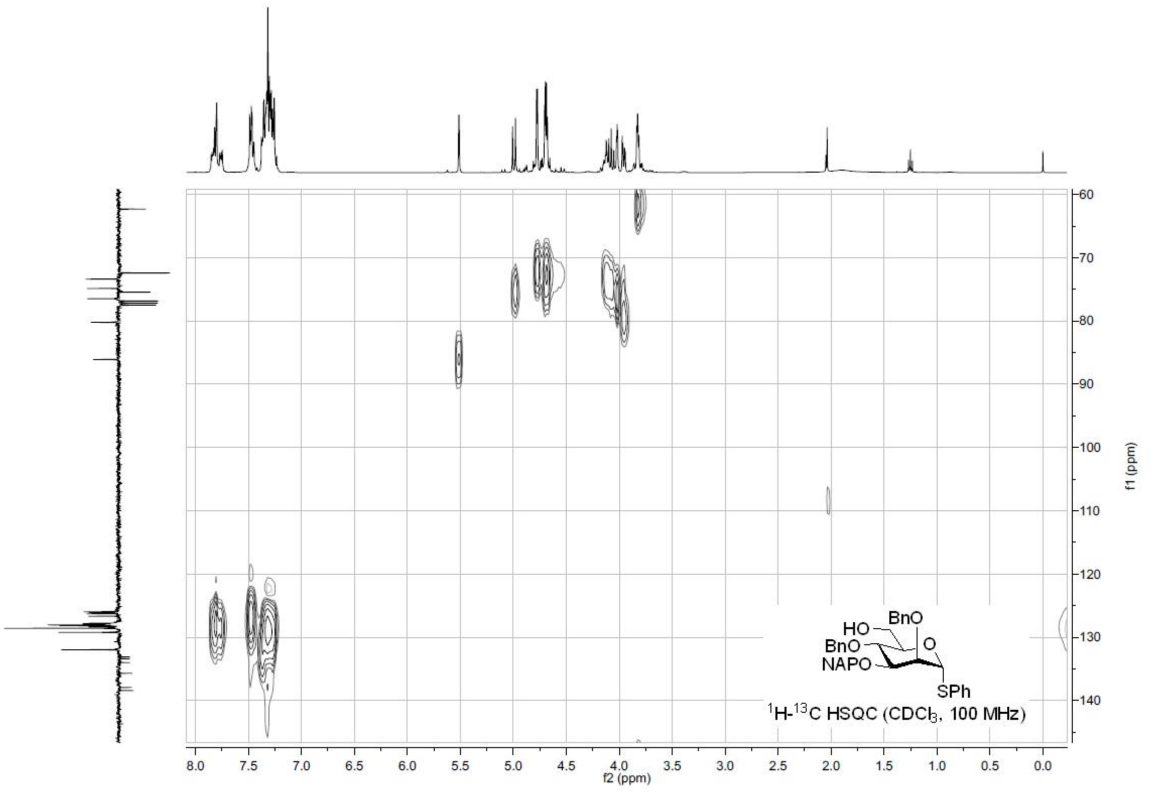
HO
BnO
BnO
NAPO
O
SPh
^{1}H-^{13}C HSQC (CDCl$_3$, 100 MHz)
f2 (ppm)
f1 (ppm)

^{1}H and ^{13}C NMR spectra of compound **25**

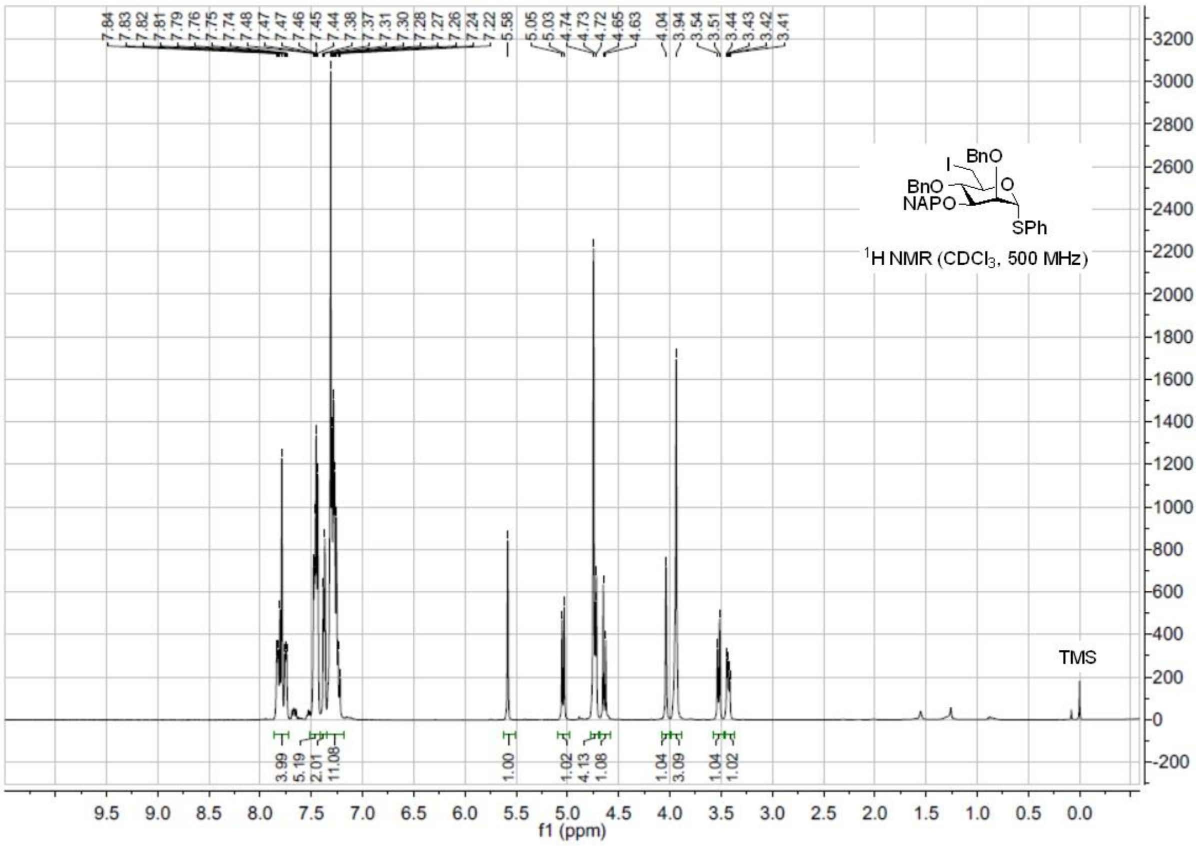

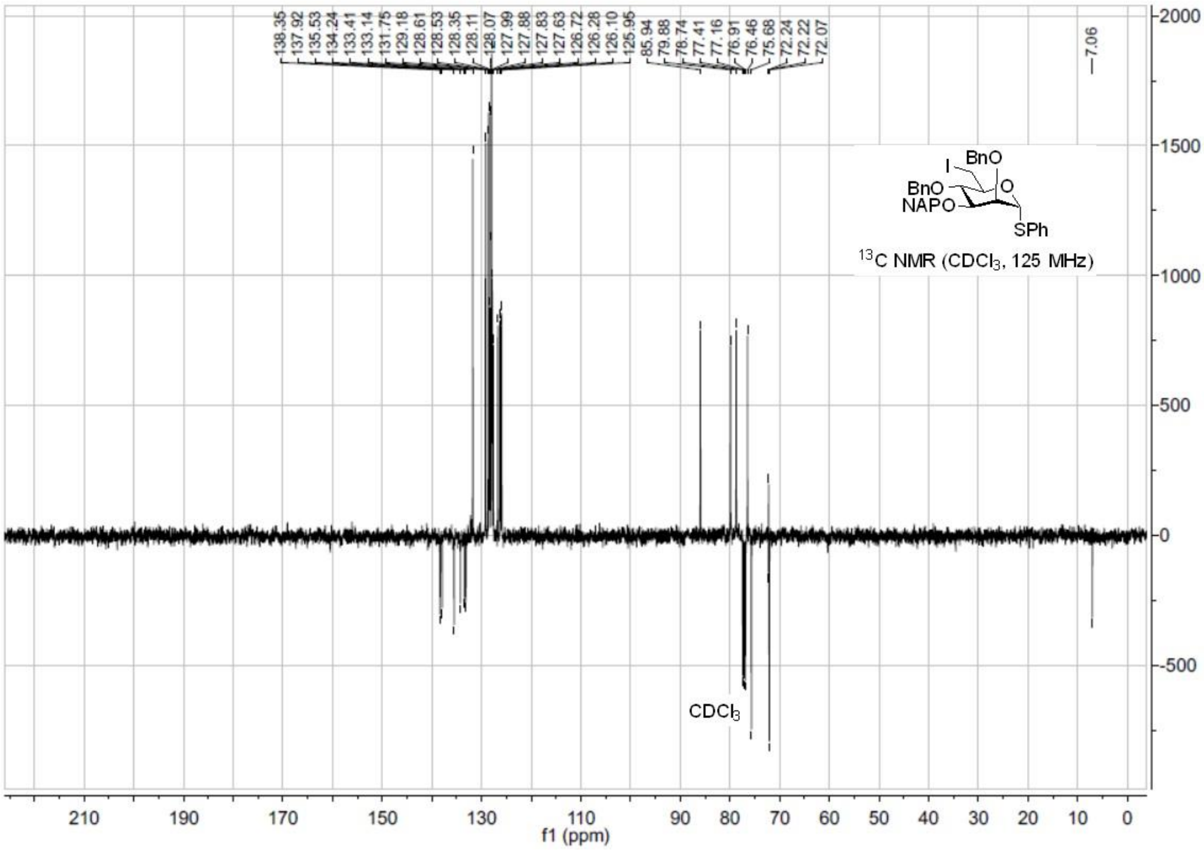

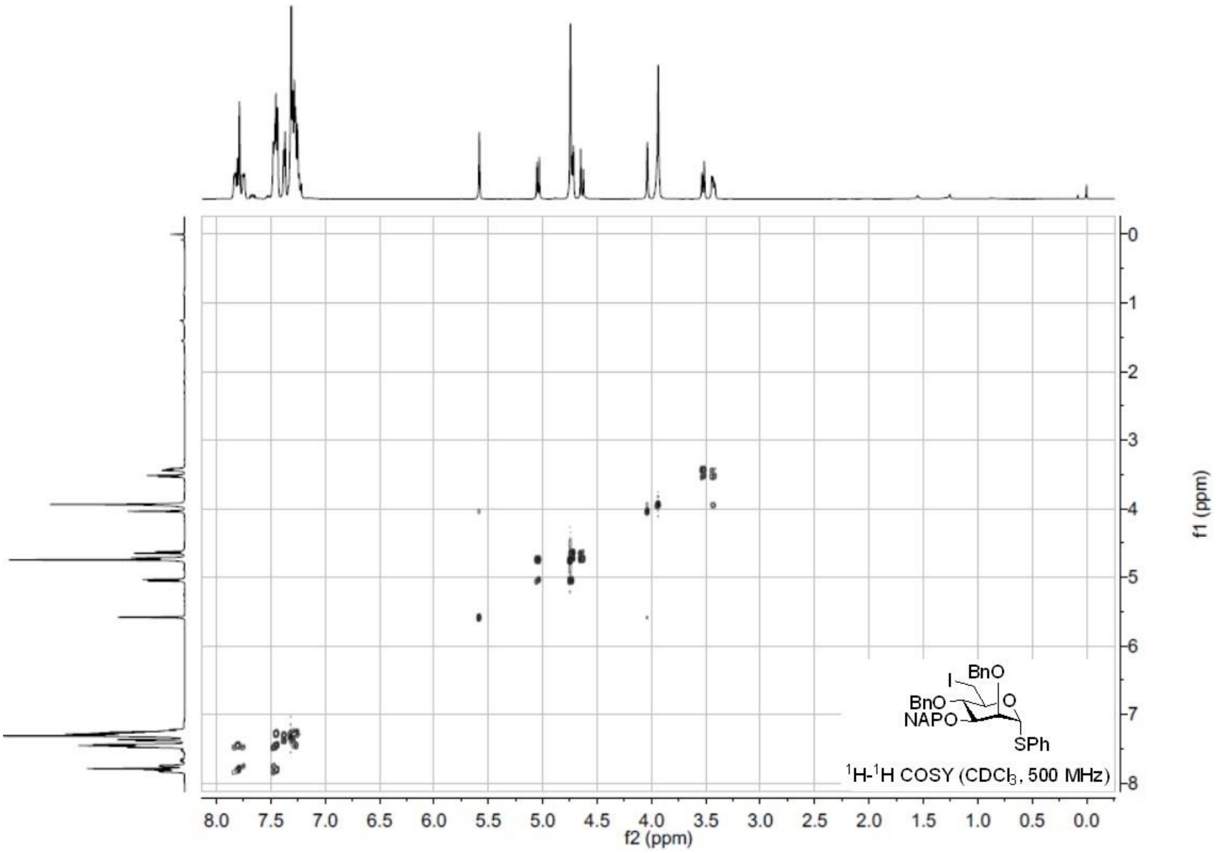
BnO
I
BnO
O
NAPO
SPh
^{1}H-^{1}H COSY (CDCl$_3$, 500 MHz)
f1 (ppm)
f2 (ppm)

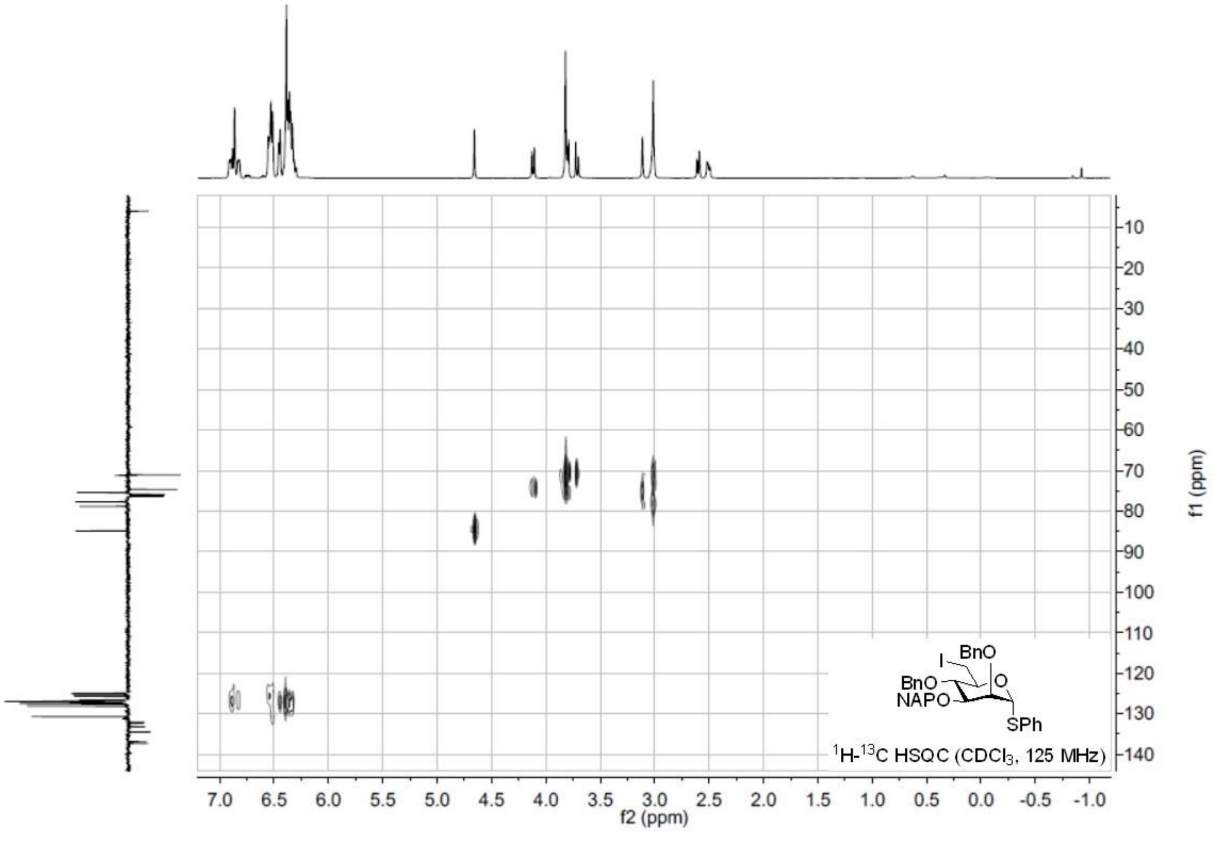
BnO
I
BnO
O
NAPO
SPh
^{1}H-^{13}C HSQC (CDCl$_3$, 125 MHz)
f1 (ppm)
f2 (ppm)

^{1}H and ^{13}C NMR spectra of compound **26**

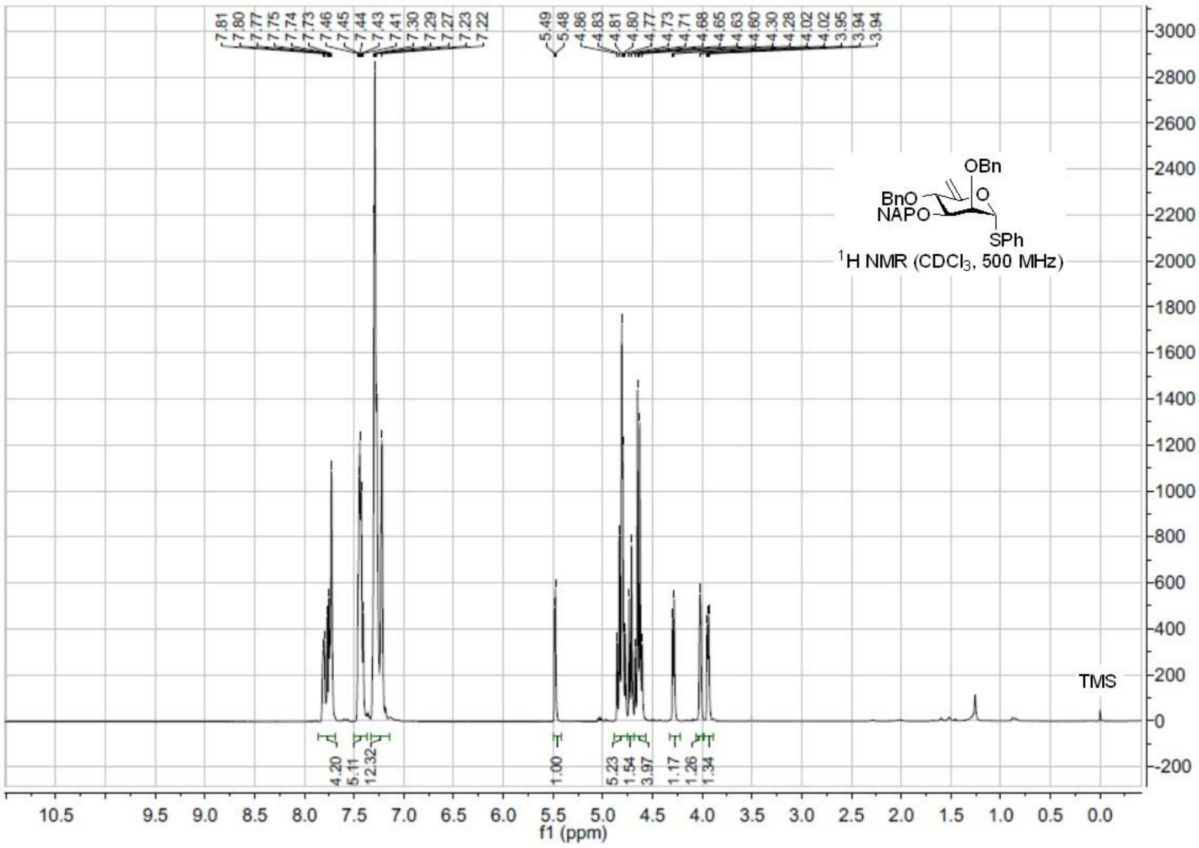

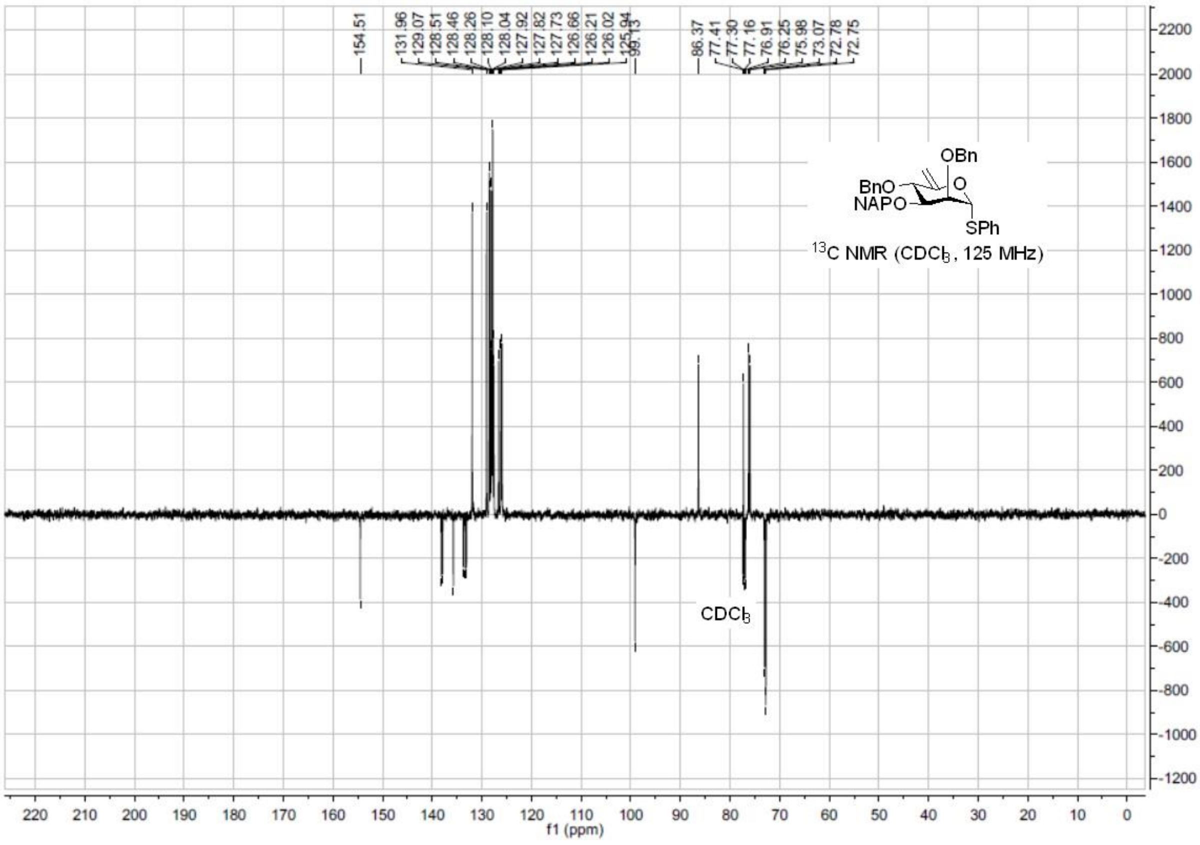

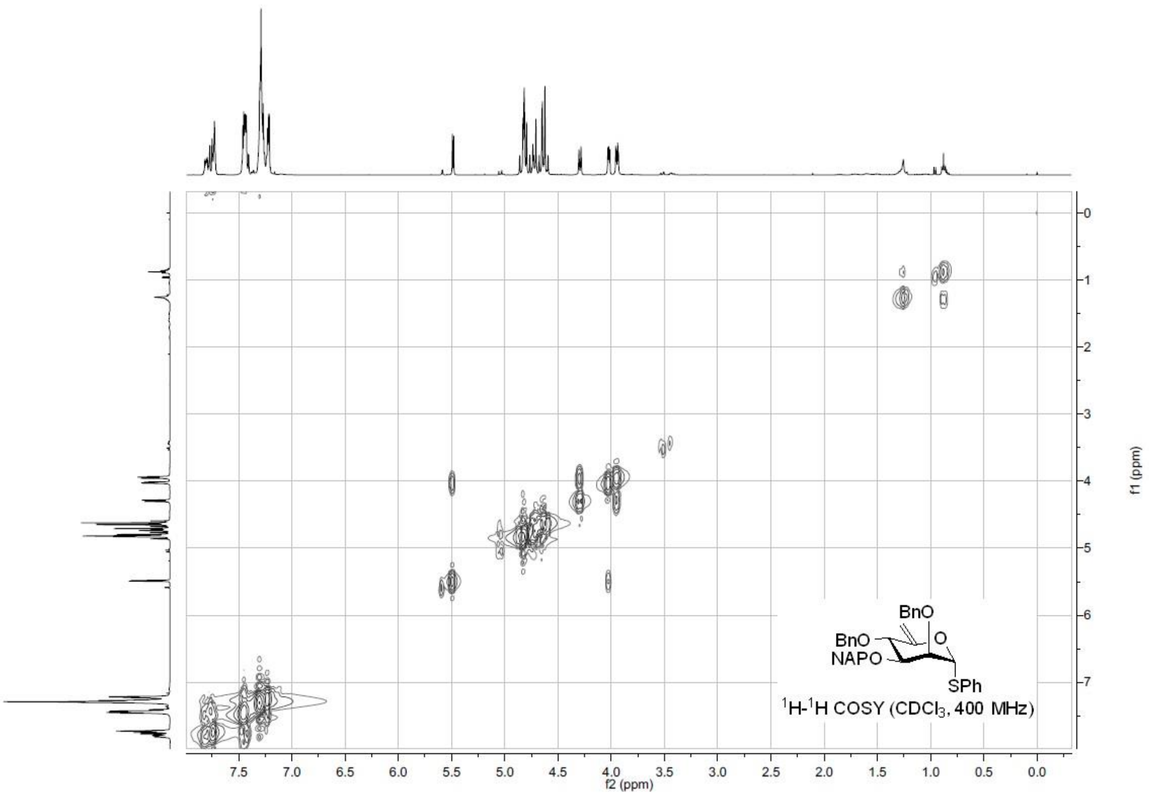
BnO
BnO
NAPO
O
SPh
^{1}H-^{1}H COSY ($CDCl_3$, 400 MHz)
f2 (ppm)
f1 (ppm)

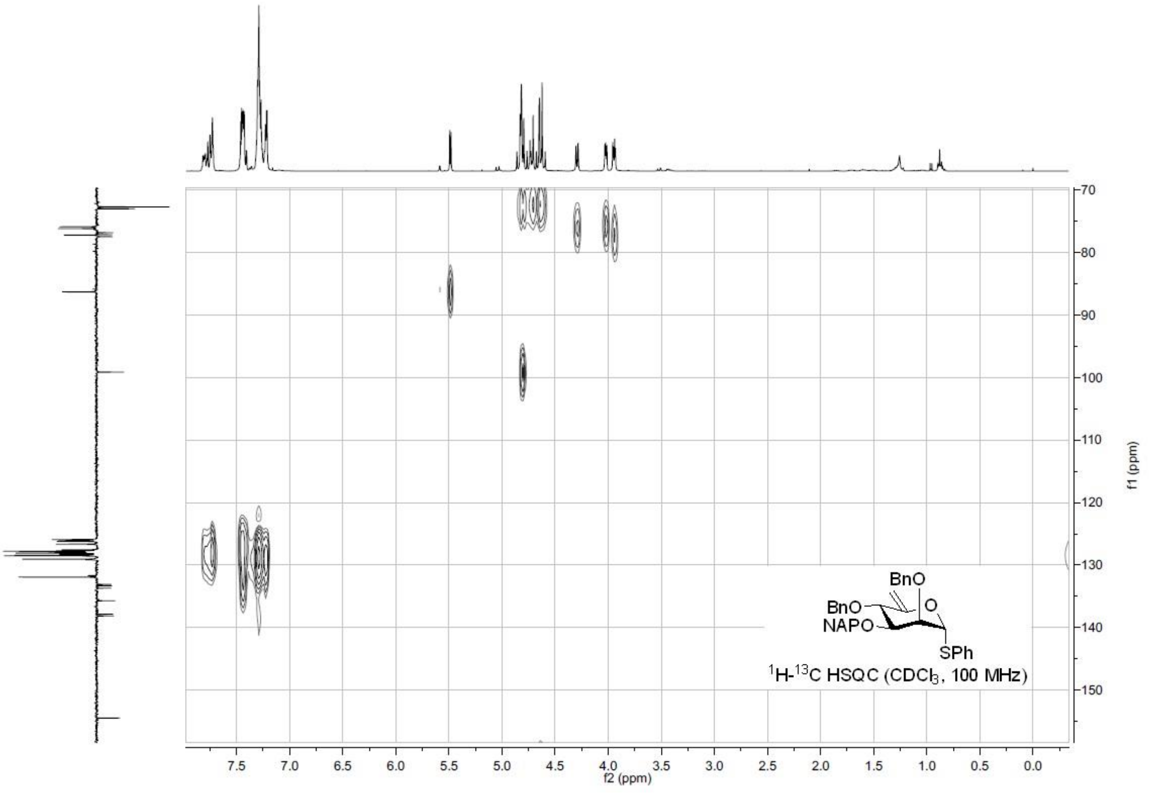
BnO
BnO
NAPO
O
SPh
^{1}H-^{13}C HSQC ($CDCl_3$, 100 MHz)
f2 (ppm)
f1 (ppm)

^{1}H and ^{13}C NMR spectra of compound **27**

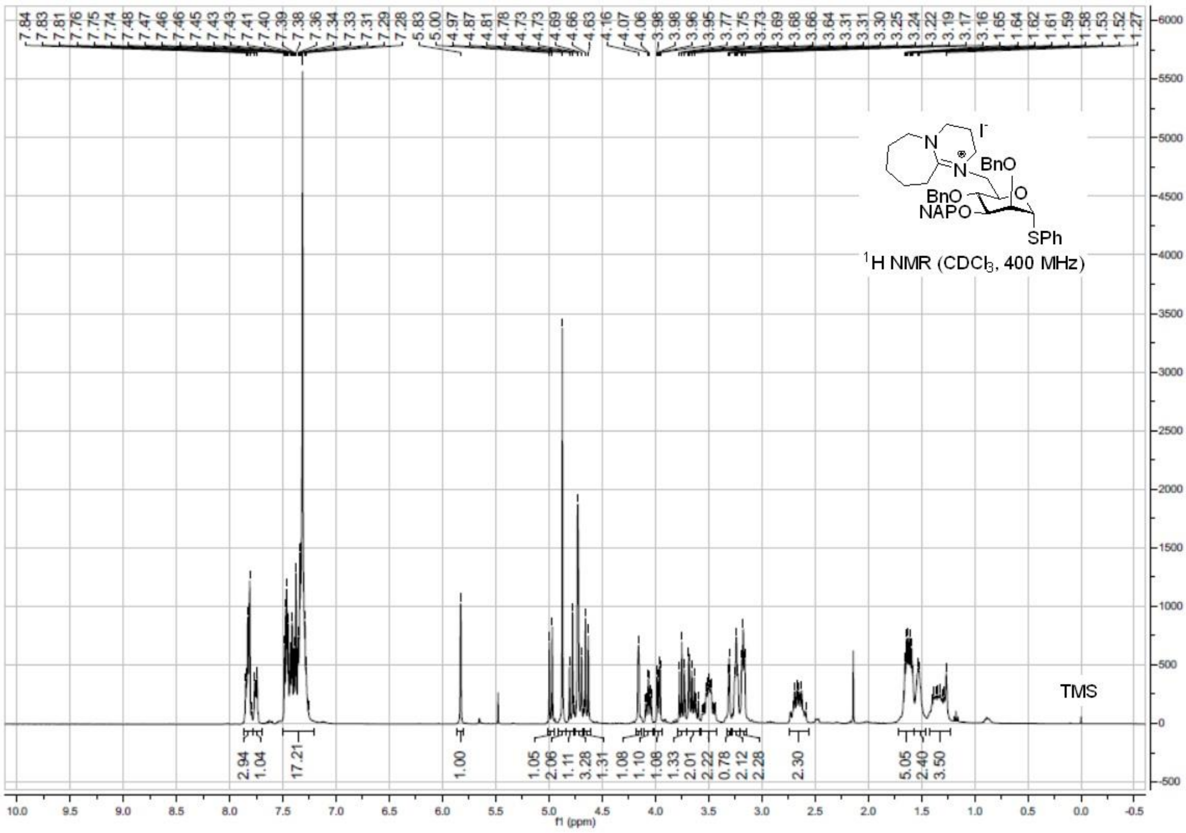

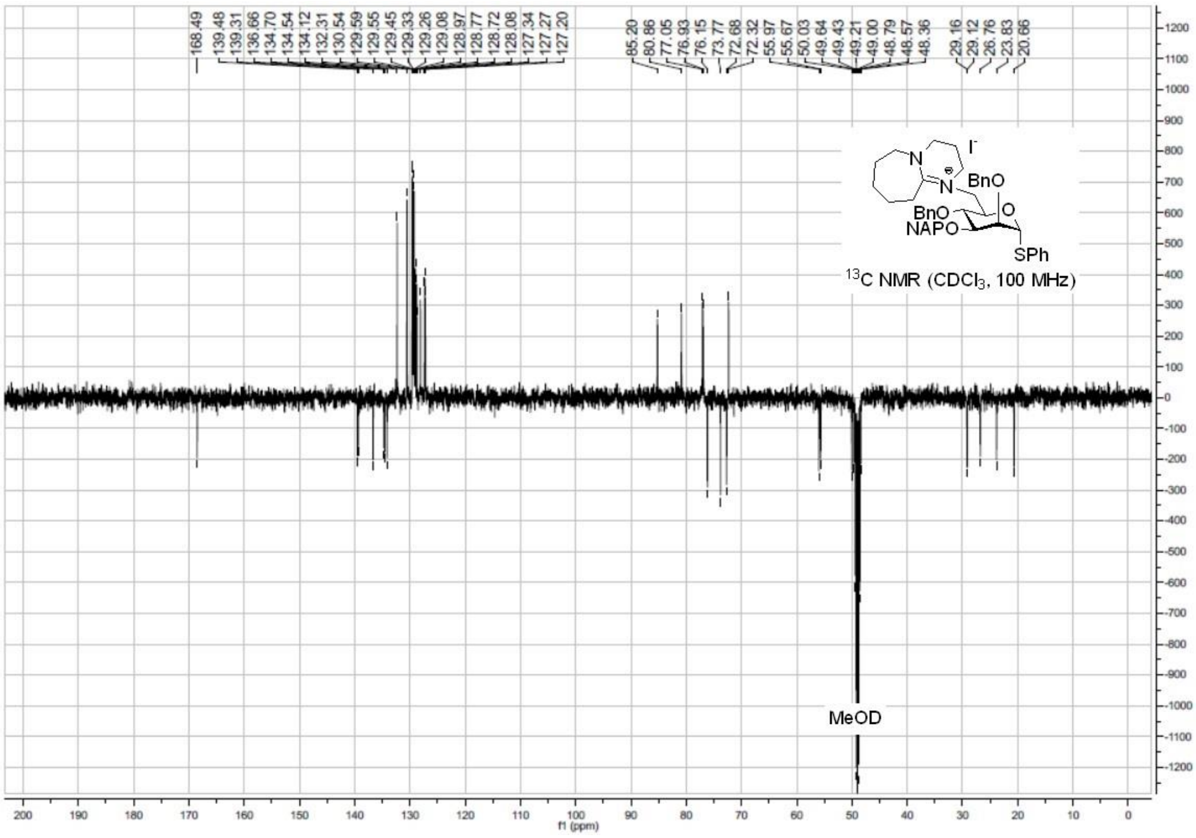

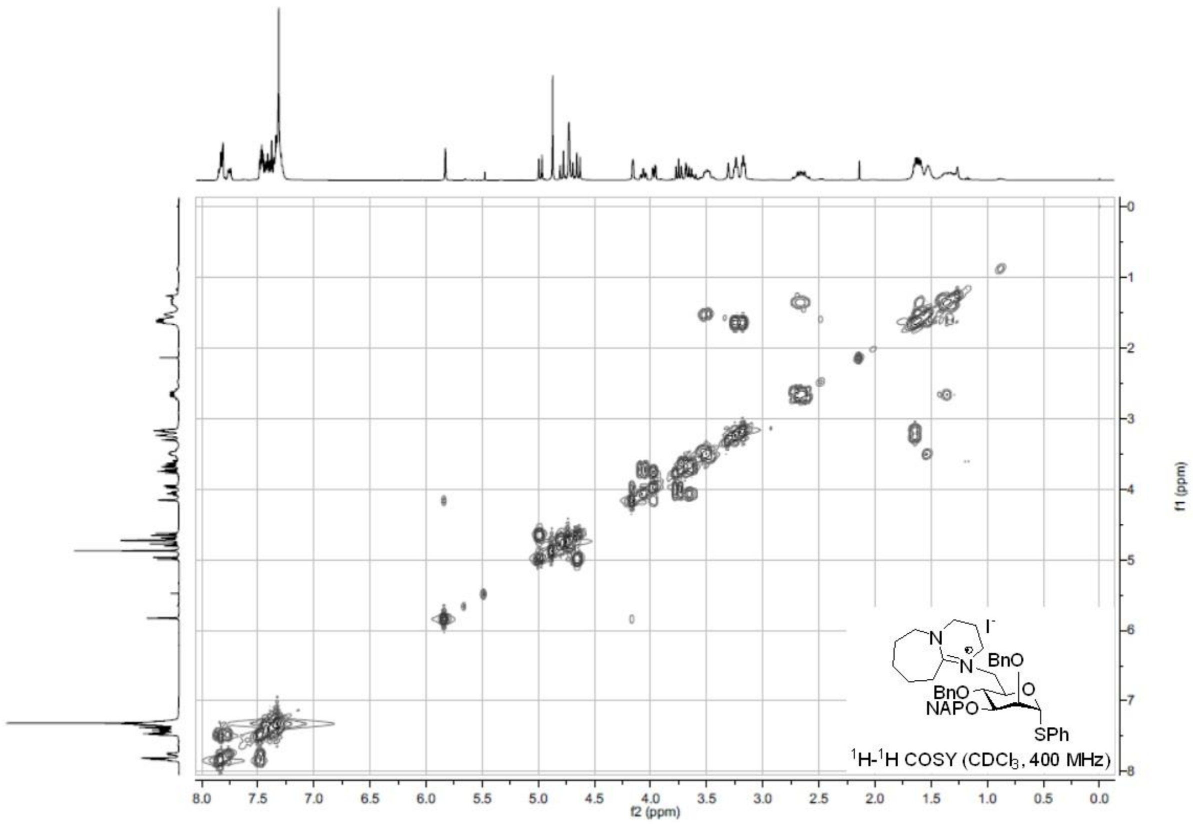

I⁻
BnO
BnO
NAPO
O
SPh
^{1}H-^{1}H COSY (CDCl$_3$, 400 MHz)
f2 (ppm)
f1 (ppm)

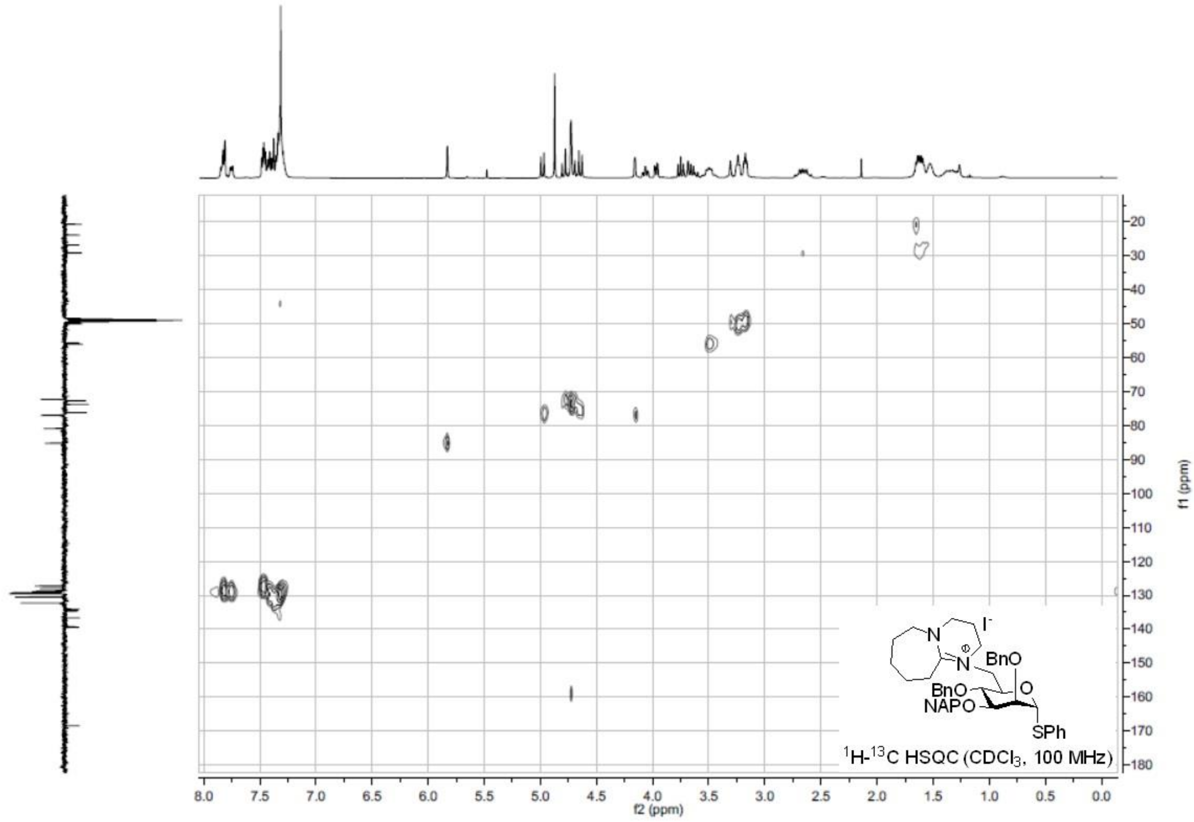

I⁻
BnO
BnO
NAPO
O
SPh
^{1}H-^{13}C HSQC (CDCl$_3$, 100 MHz)
f2 (ppm)
f1 (ppm)

^1H and ^{13}C NMR spectra of compound **28**

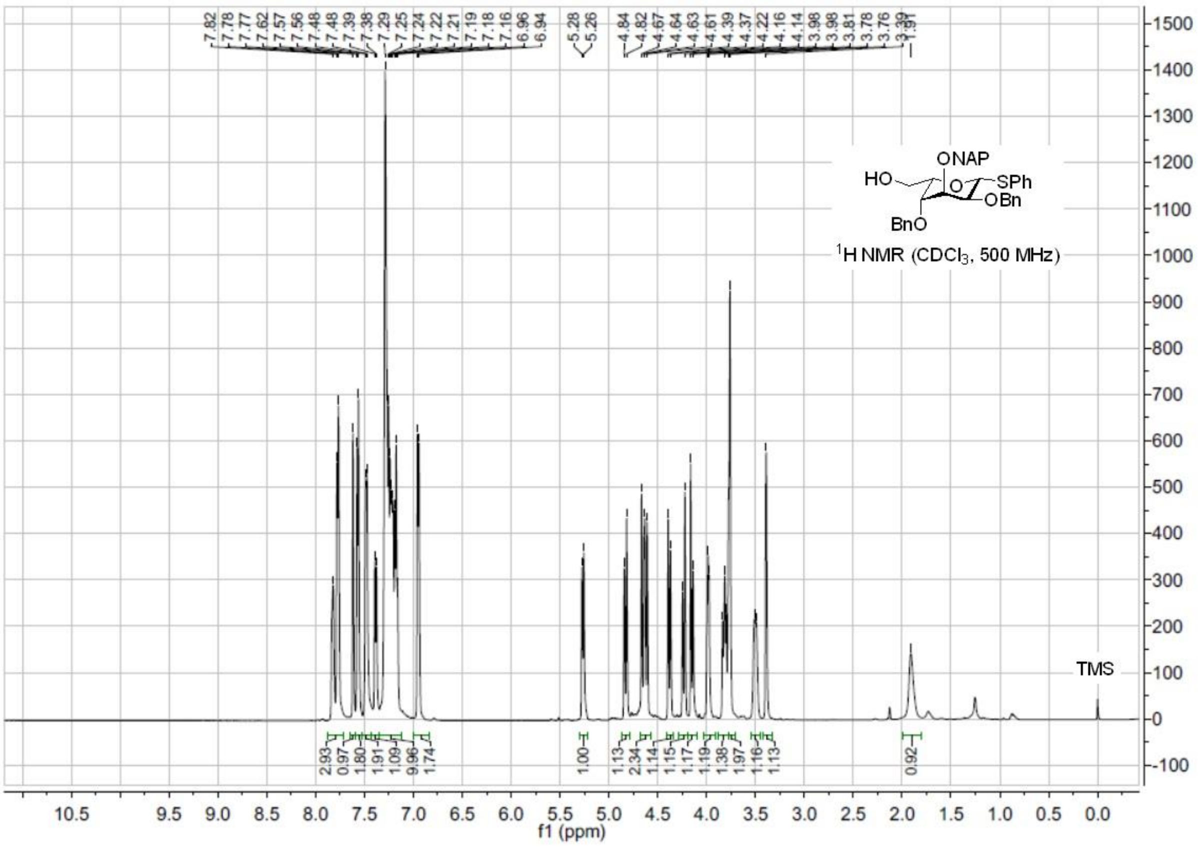

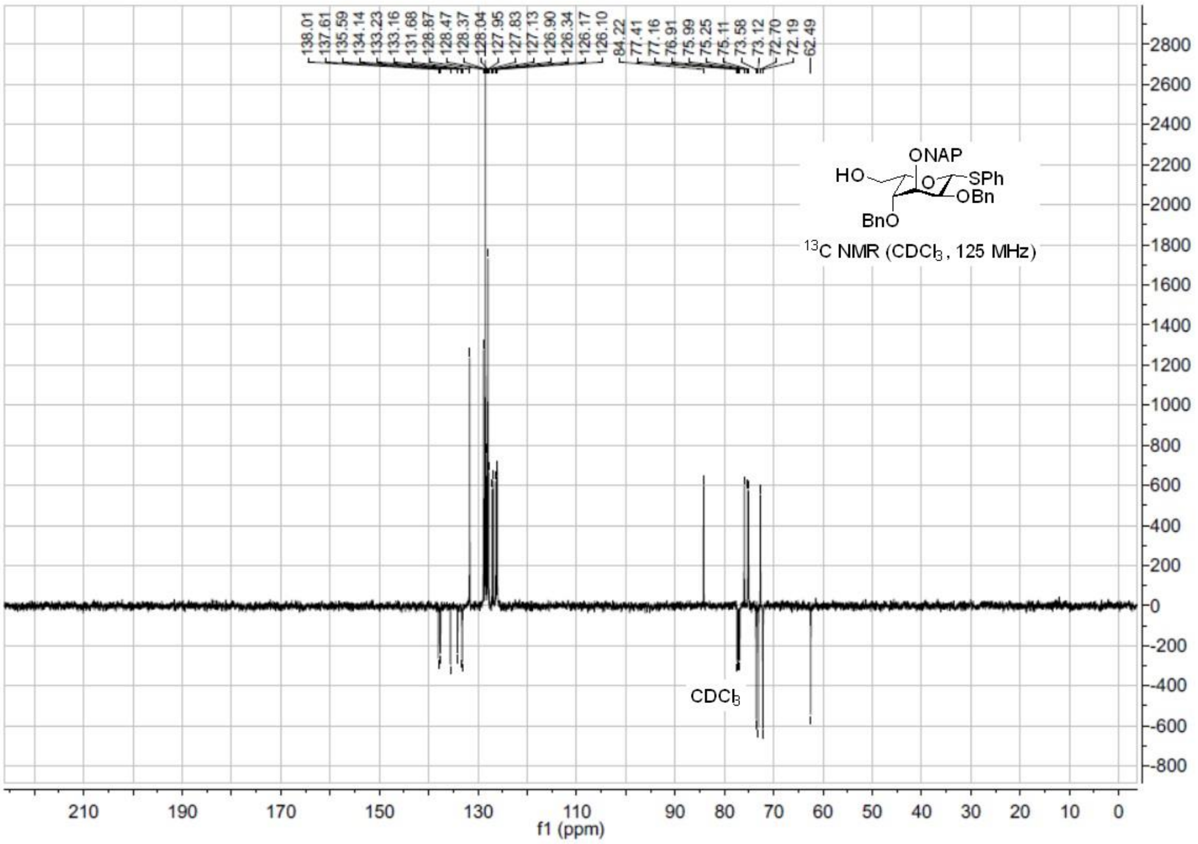

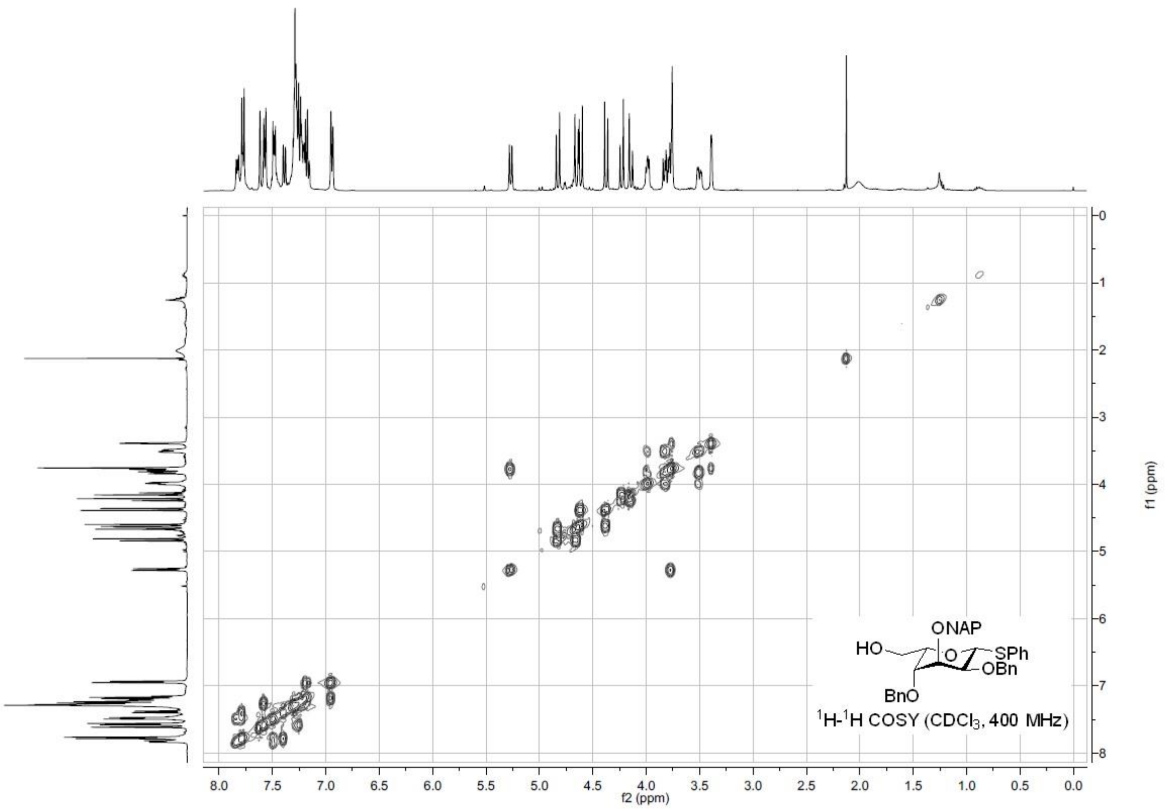
ONAP
HO
O
SPh
OBn
BnO
¹H-¹H COSY (CDCl₃, 400 MHz)
f2 (ppm)
f1 (ppm)

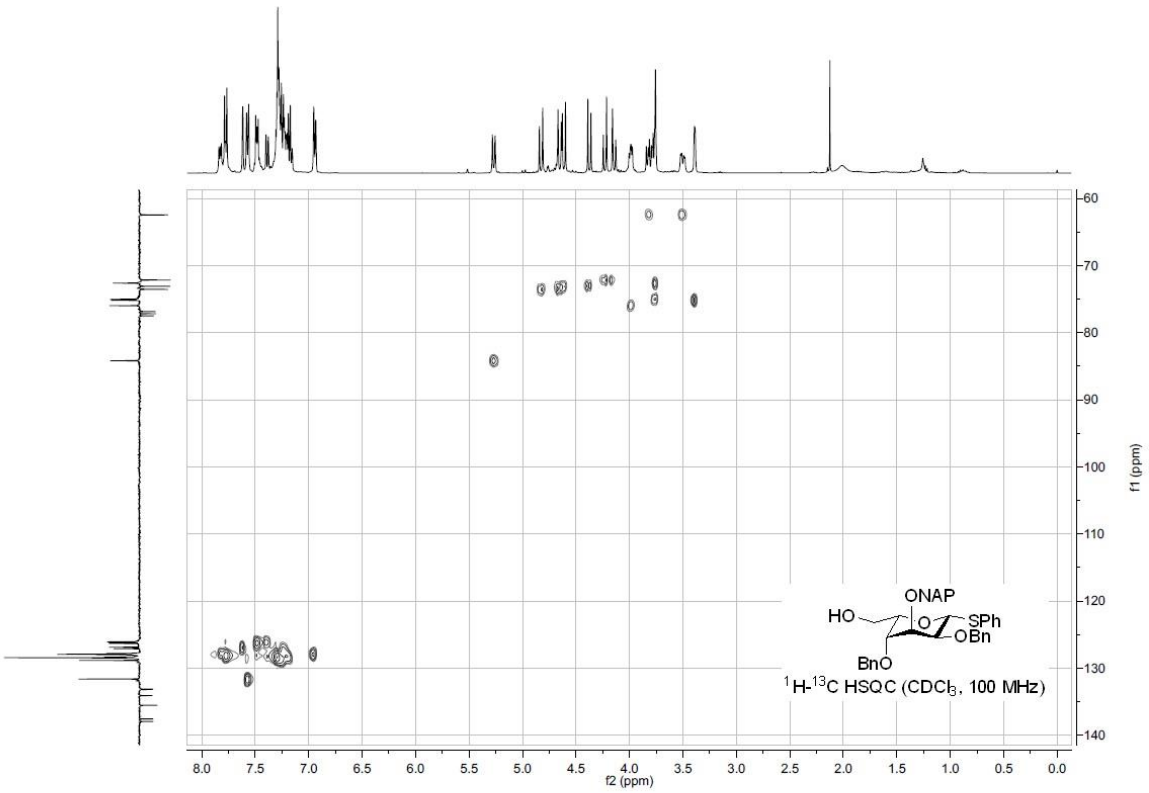
ONAP
HO
O
SPh
OBn
BnO
¹H-¹³C HSQC (CDCl₃, 100 MHz)
f2 (ppm)
f1 (ppm)

^{1}H and ^{13}C NMR spectra of compound **30**

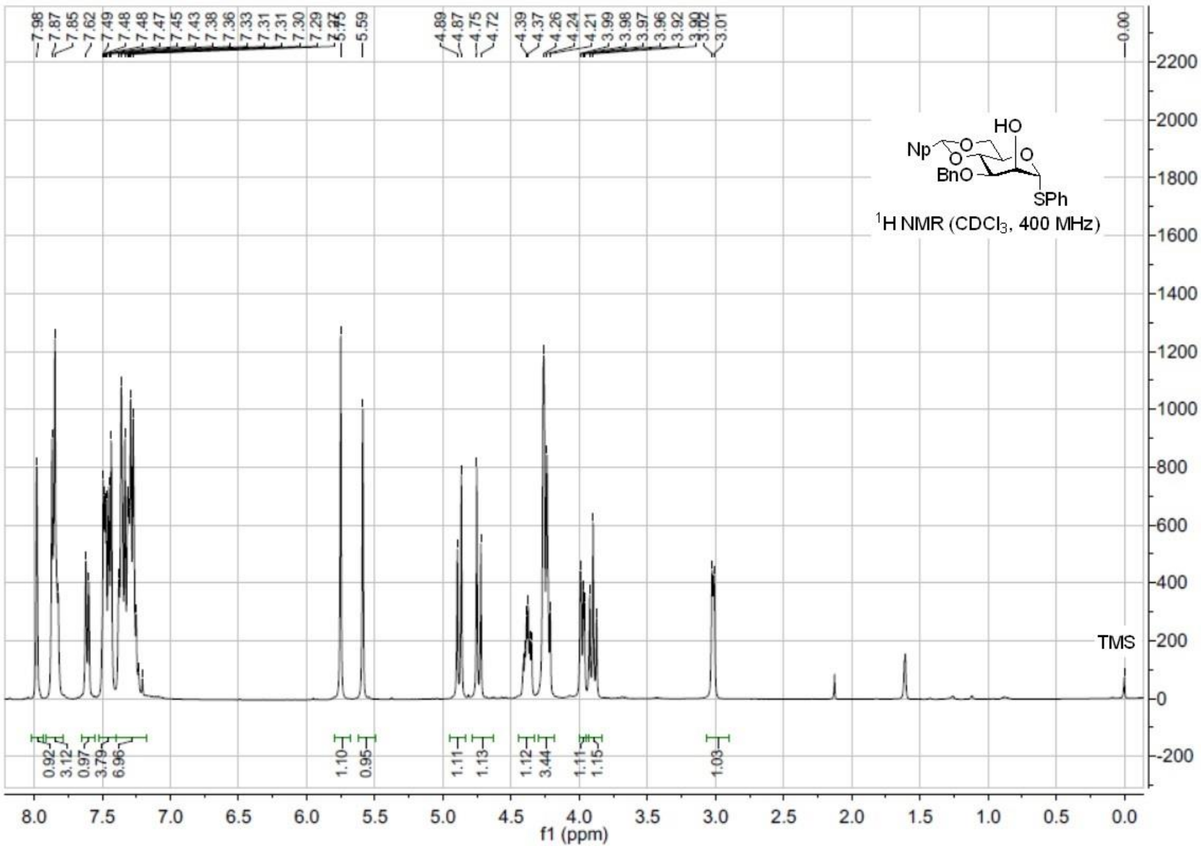

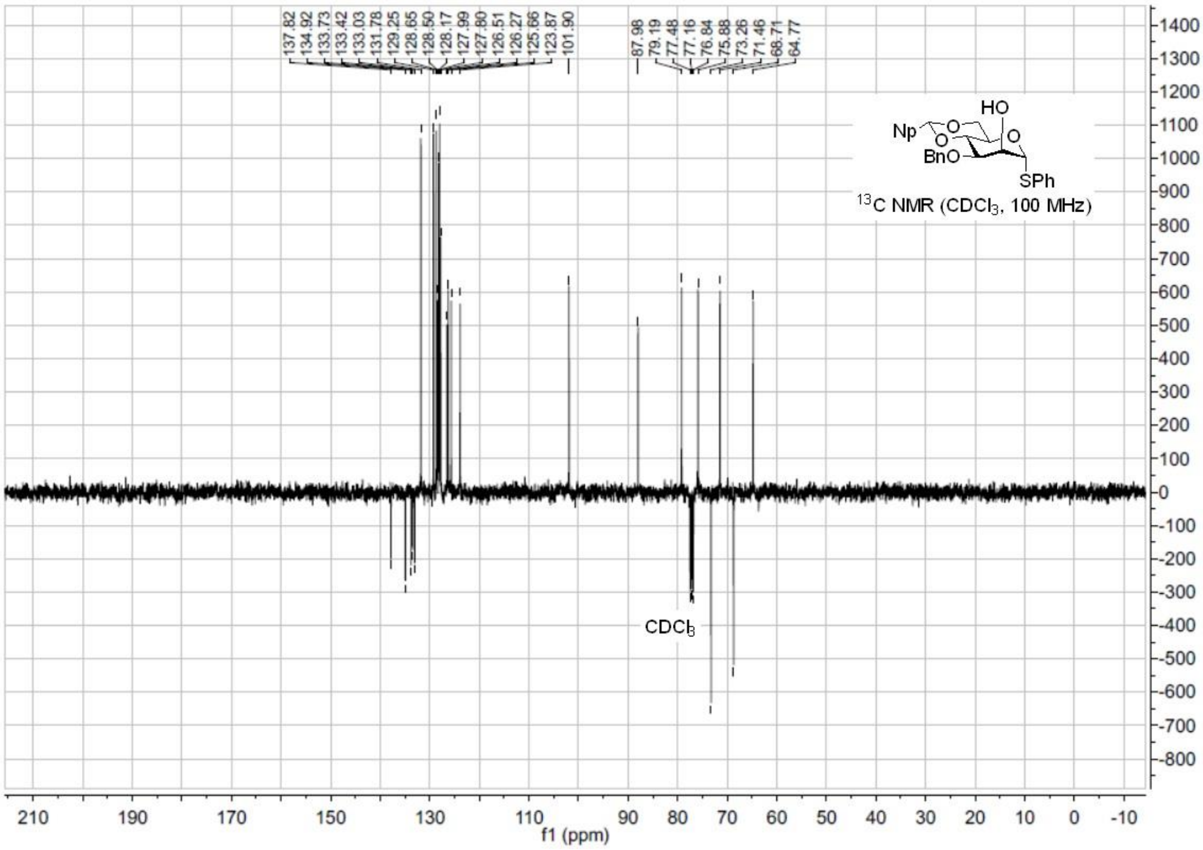

^{1}H and ^{13}C NMR spectra of compound **31**

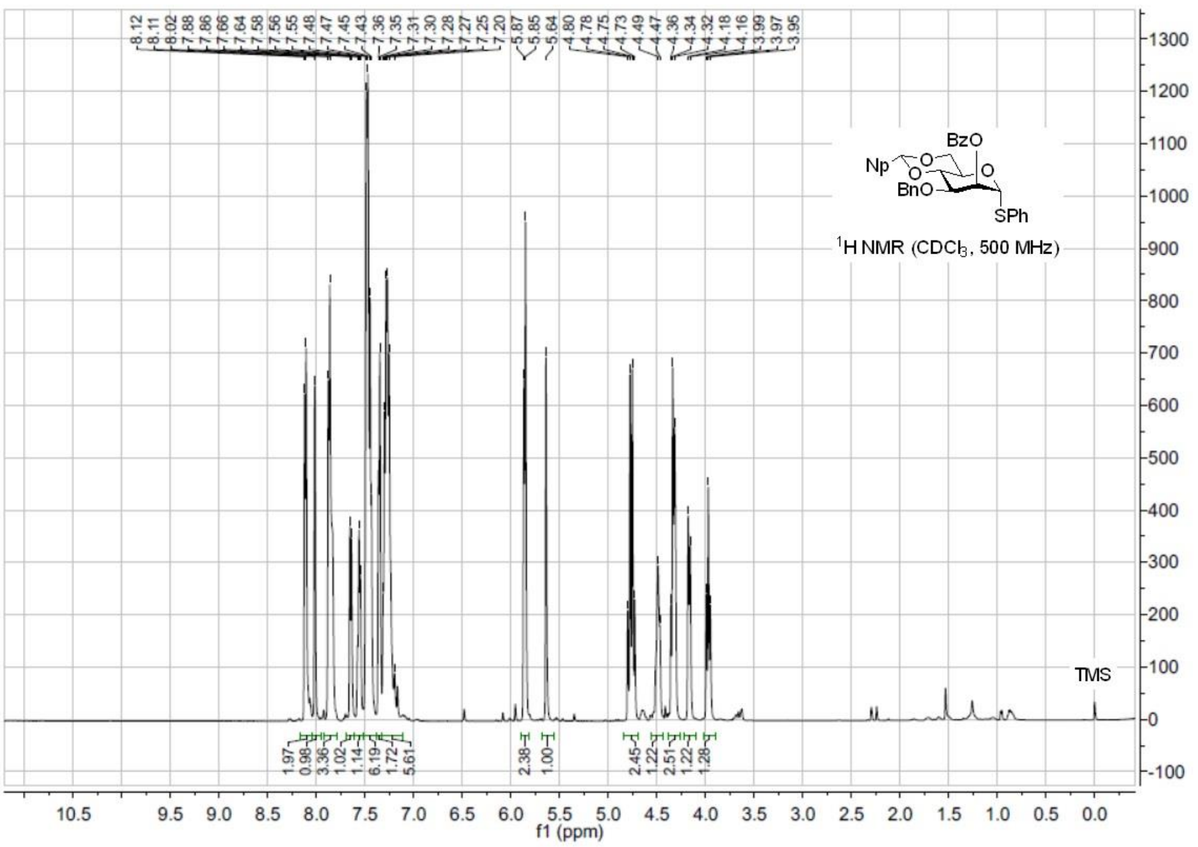

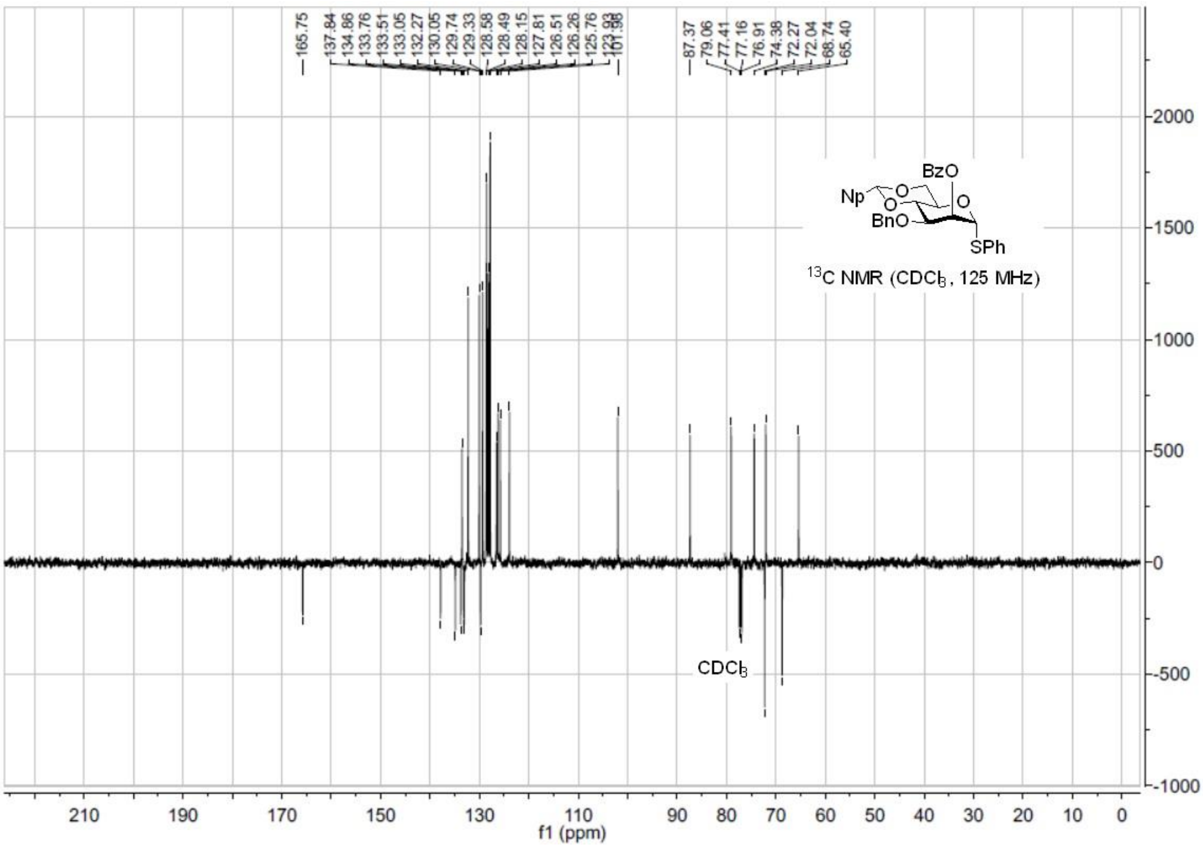

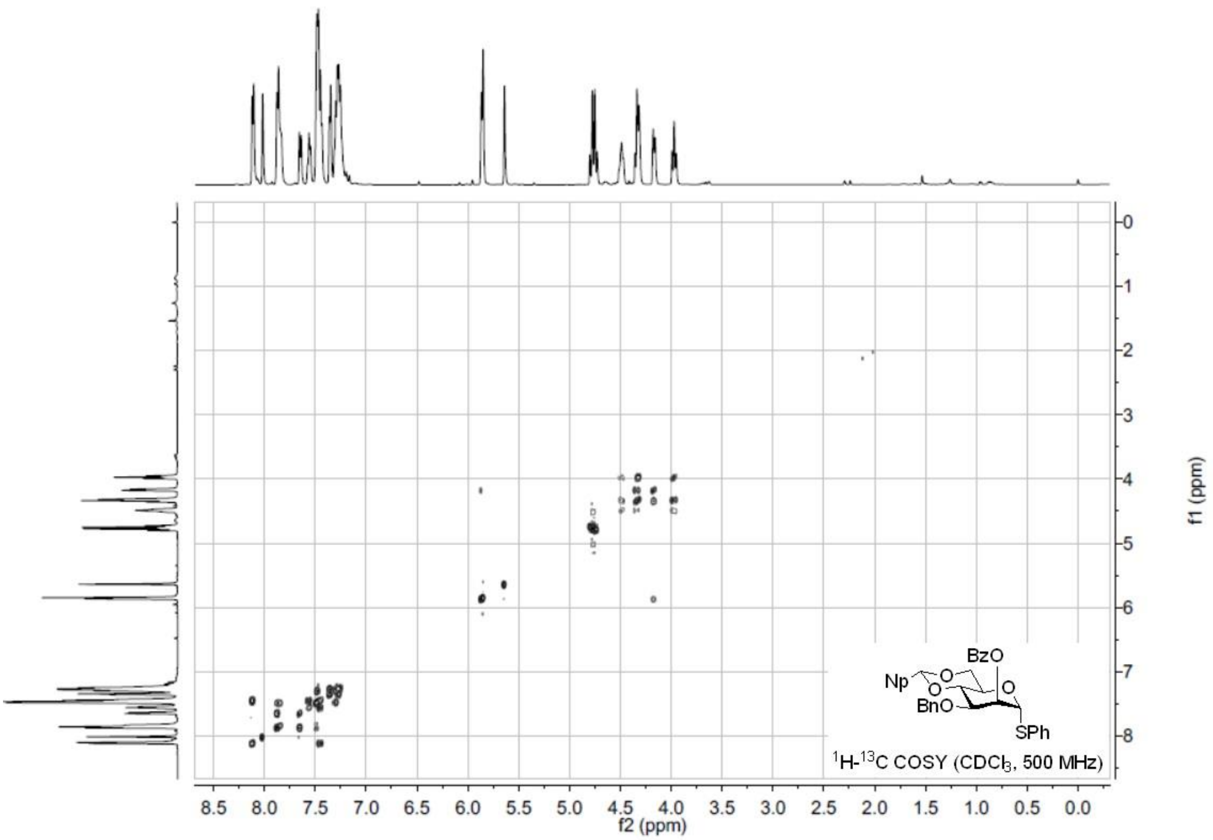
BzO
Np
O
O
O
BnO
SPh
^{1}H-^{13}C COSY (CDCl$_3$, 500 MHz)
f2 (ppm)
f1 (ppm)

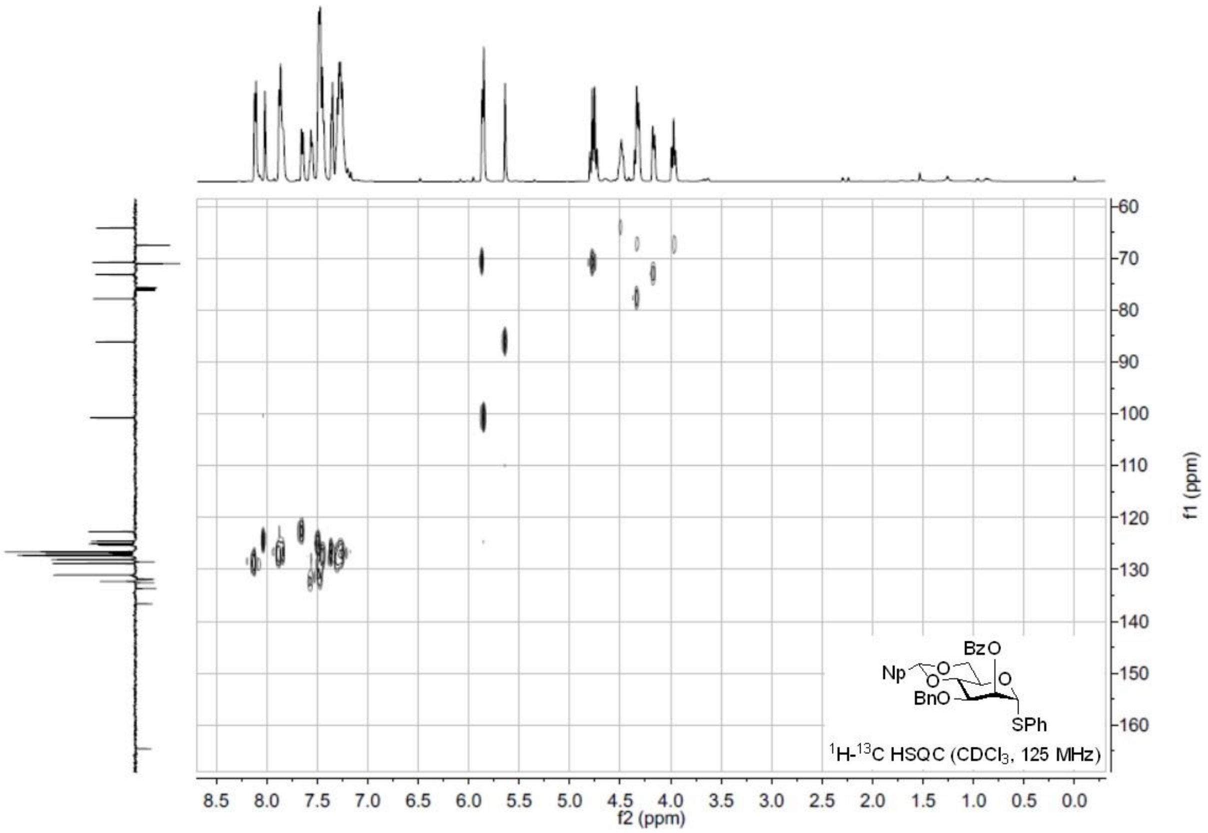
BzO
Np
O
O
O
BnO
SPh
^{1}H-^{13}C HSQC (CDCl$_3$, 125 MHz)
f2 (ppm)
f1 (ppm)

^{1}H and ^{13}C NMR spectra of compound **32**

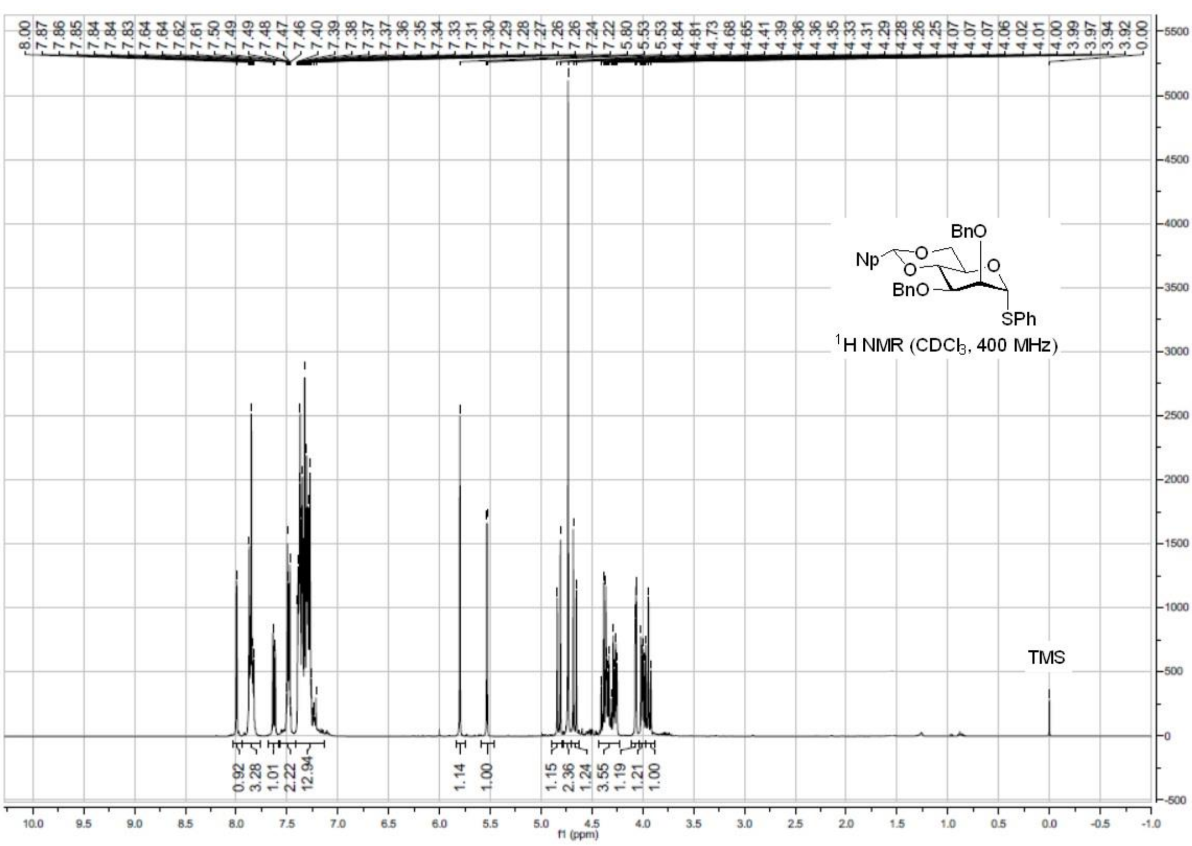

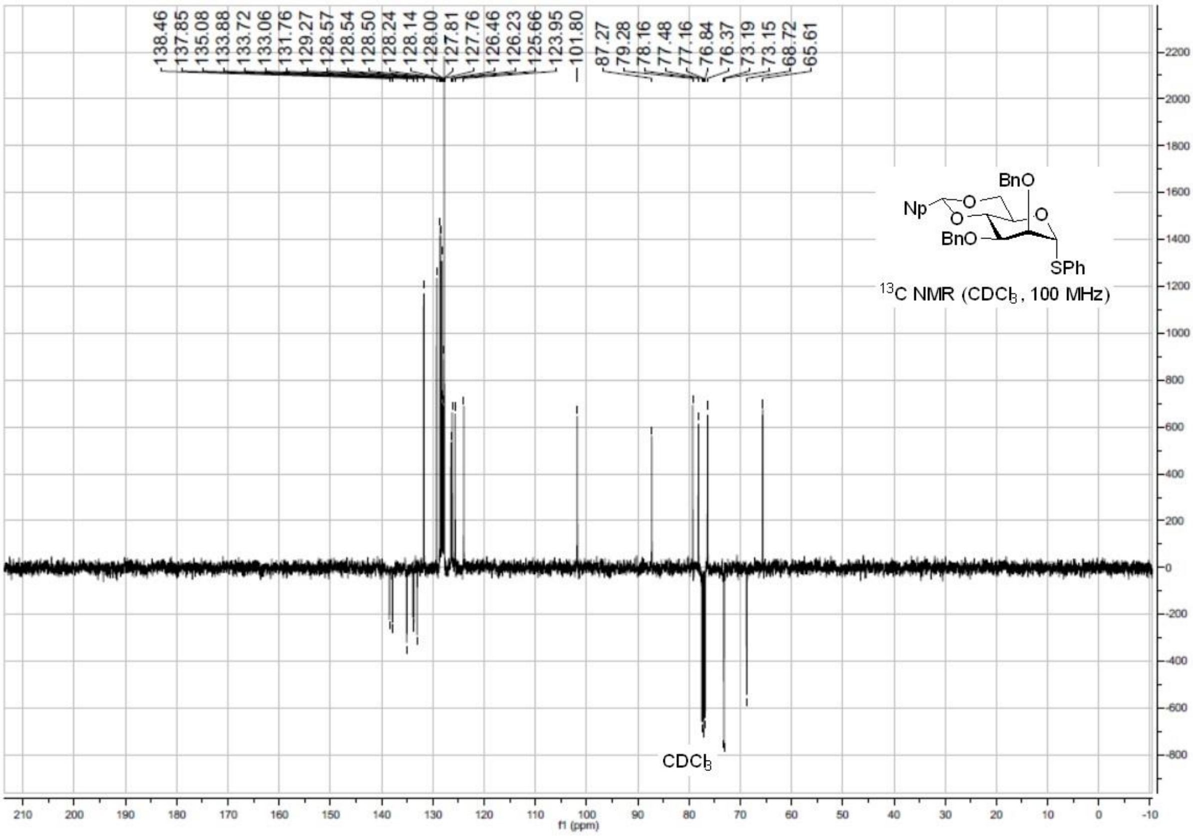

^{1}H and ^{13}C NMR spectra of compound **33**

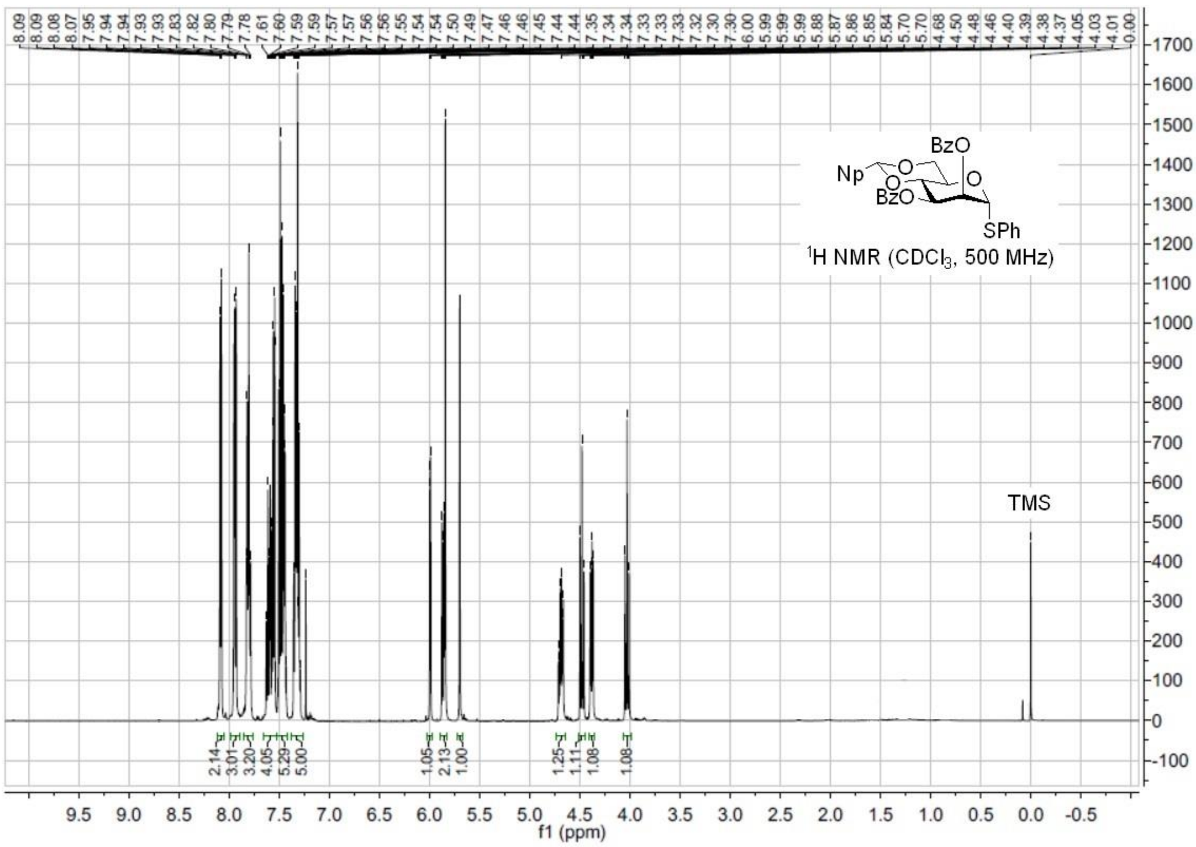

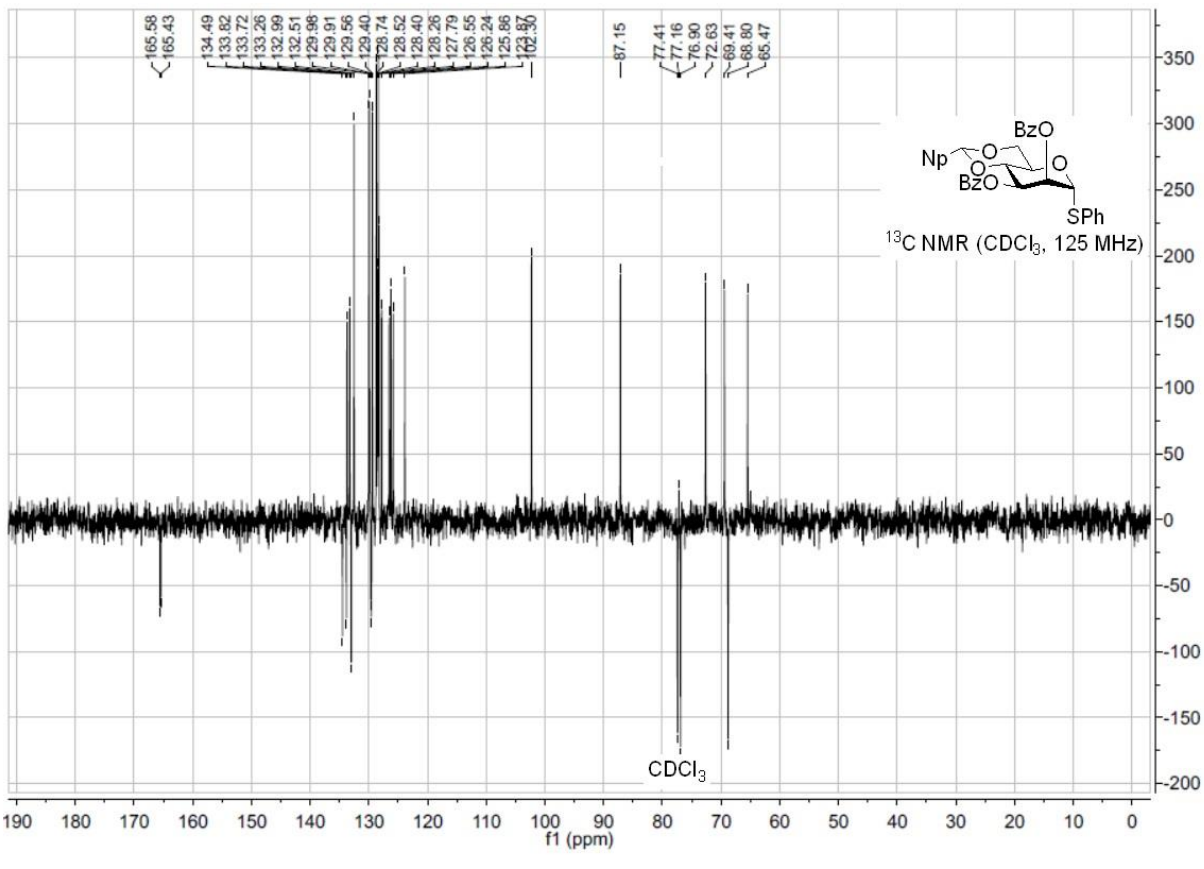

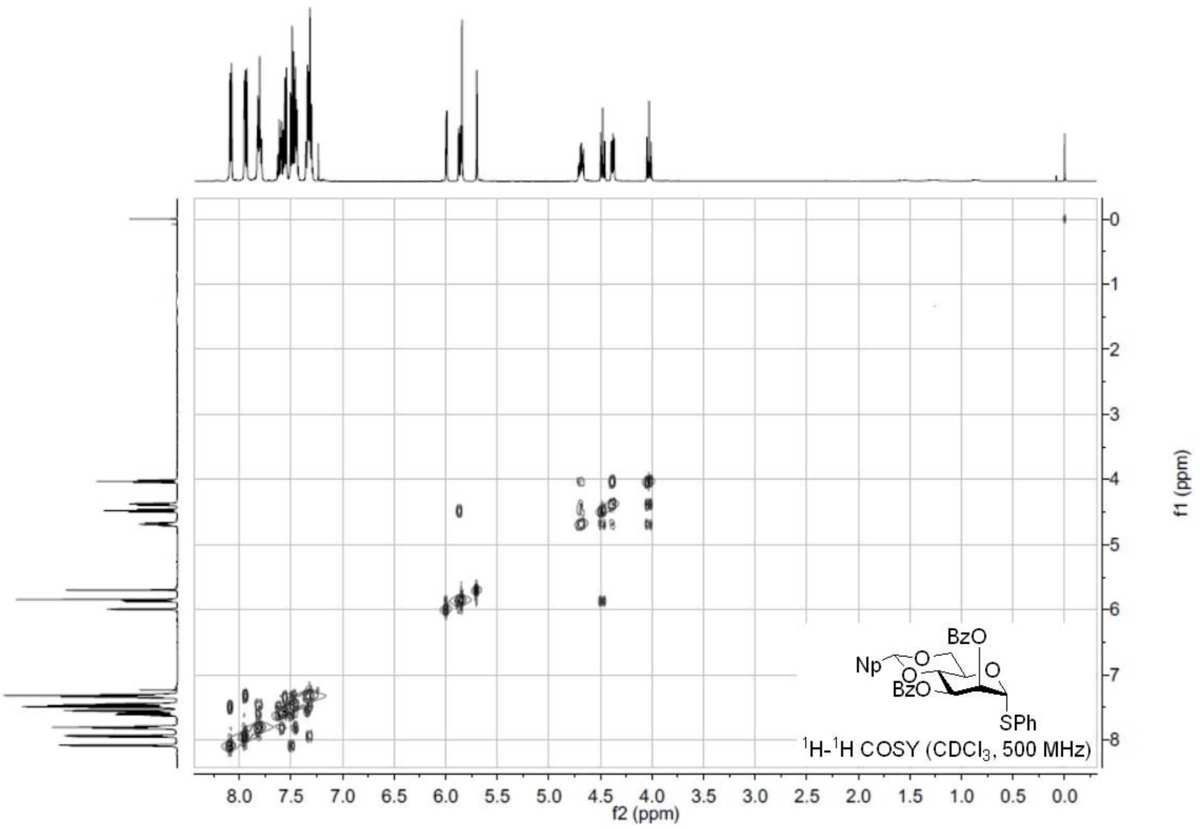
BzO
Np
O
O
BzO
O
SPh
^{1}H-^{1}H COSY (CDCl$_3$, 500 MHz)
f1 (ppm)
f2 (ppm)

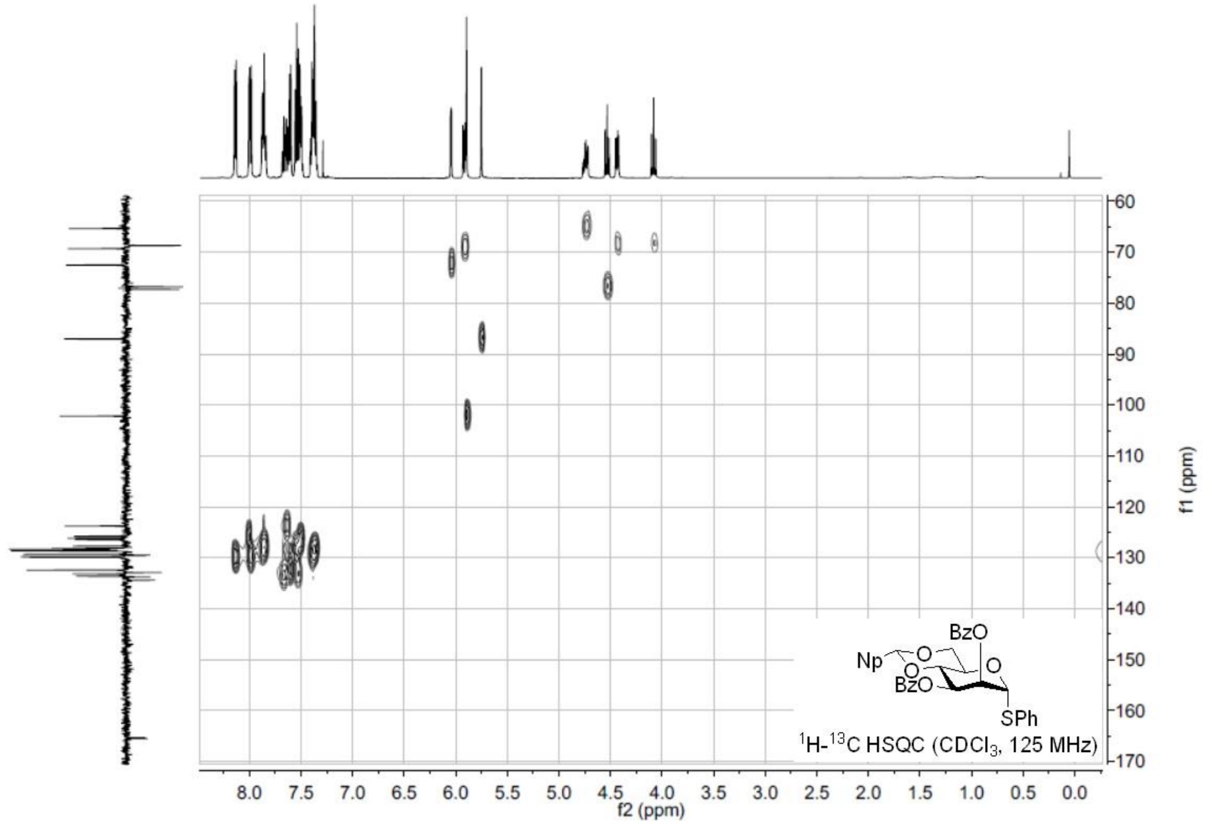
BzO
Np
O
O
BzO
O
SPh
^{1}H-^{13}C HSQC (CDCl$_3$, 125 MHz)
f1 (ppm)
f2 (ppm)

^{1}H and ^{13}C NMR spectra of compound **34**

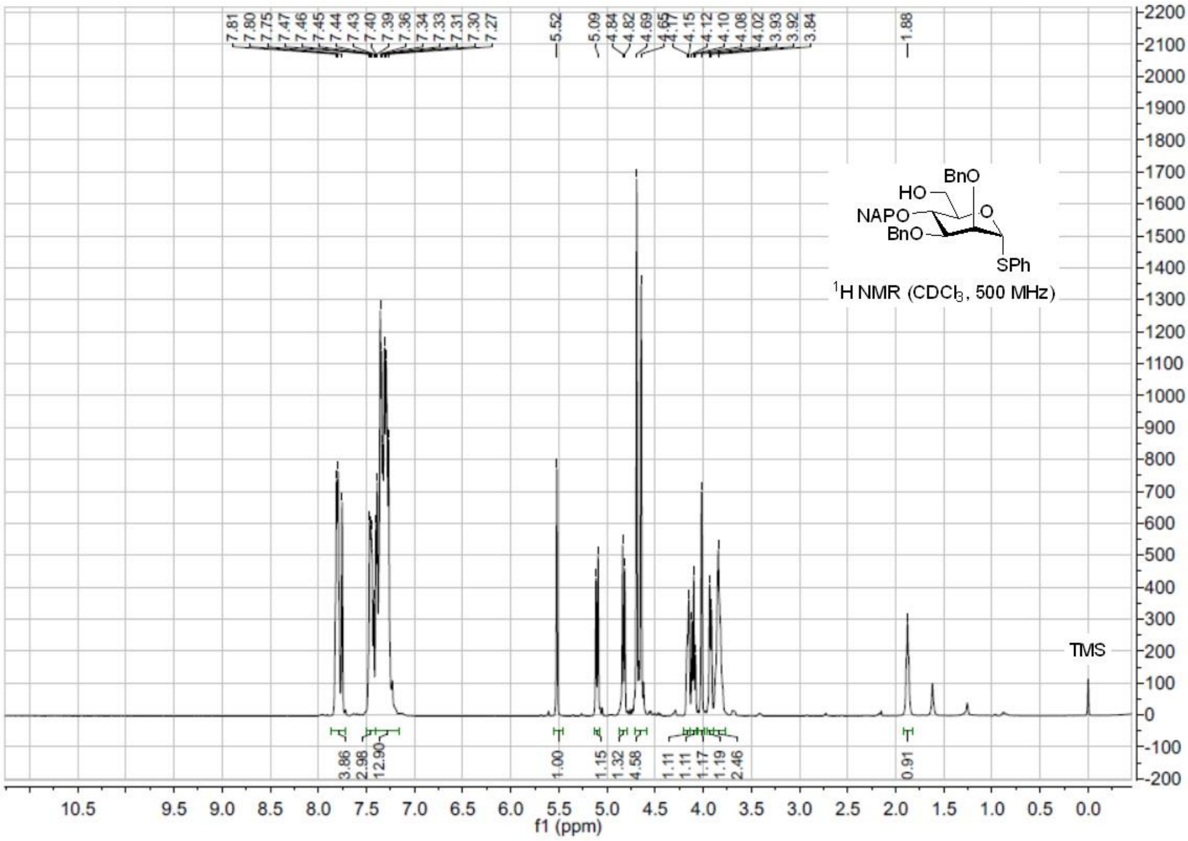

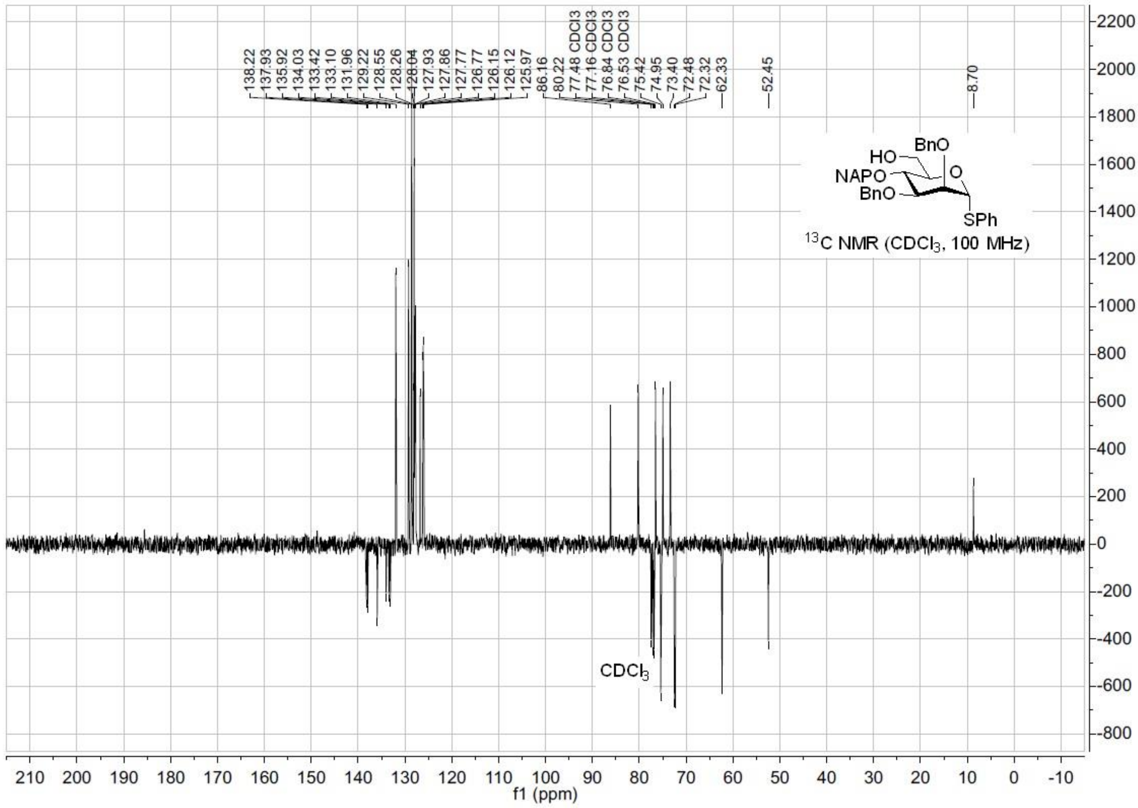

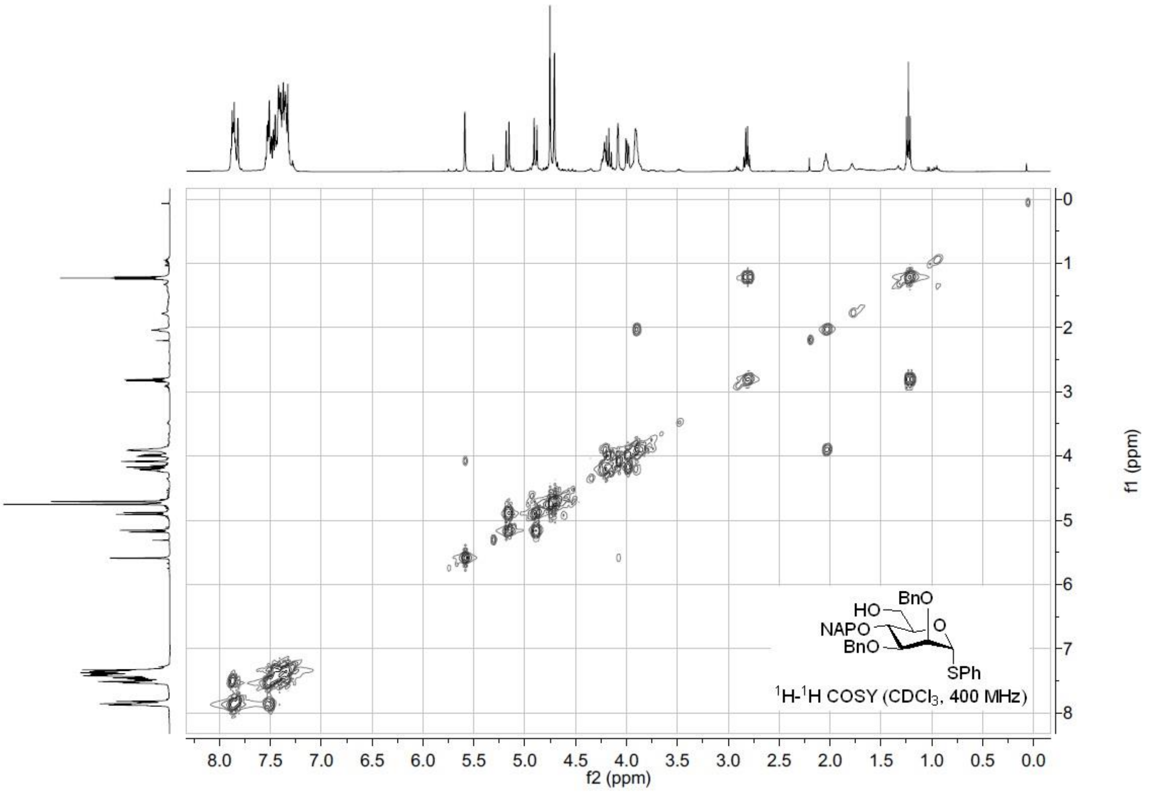
HO
BnO
NAPO
BnO
O
SPh
^{1}H-^{1}H COSY (CDCl$_3$, 400 MHz)
f2 (ppm)
f1 (ppm)

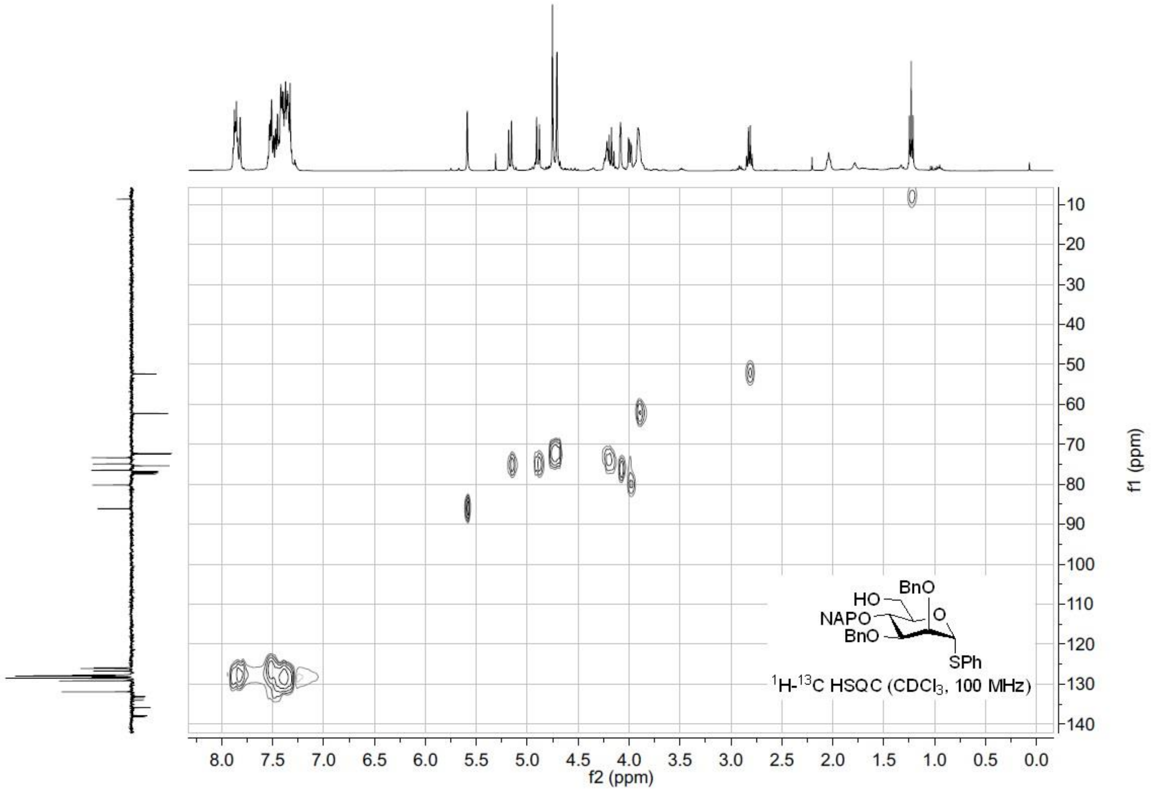
HO
BnO
NAPO
BnO
O
SPh
^{1}H-^{13}C HSQC (CDCl$_3$, 100 MHz)
f2 (ppm)
f1 (ppm)

^{1}H and ^{13}C NMR spectra of compound **35**

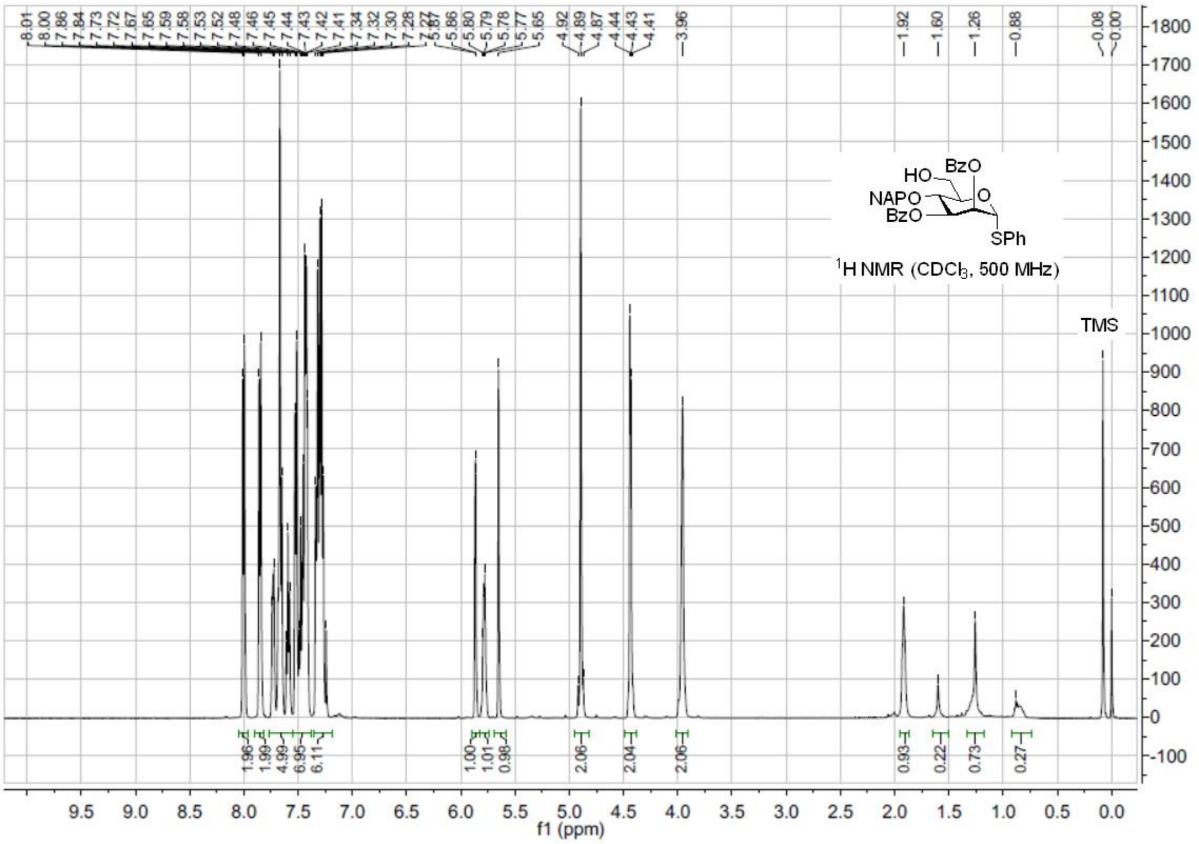

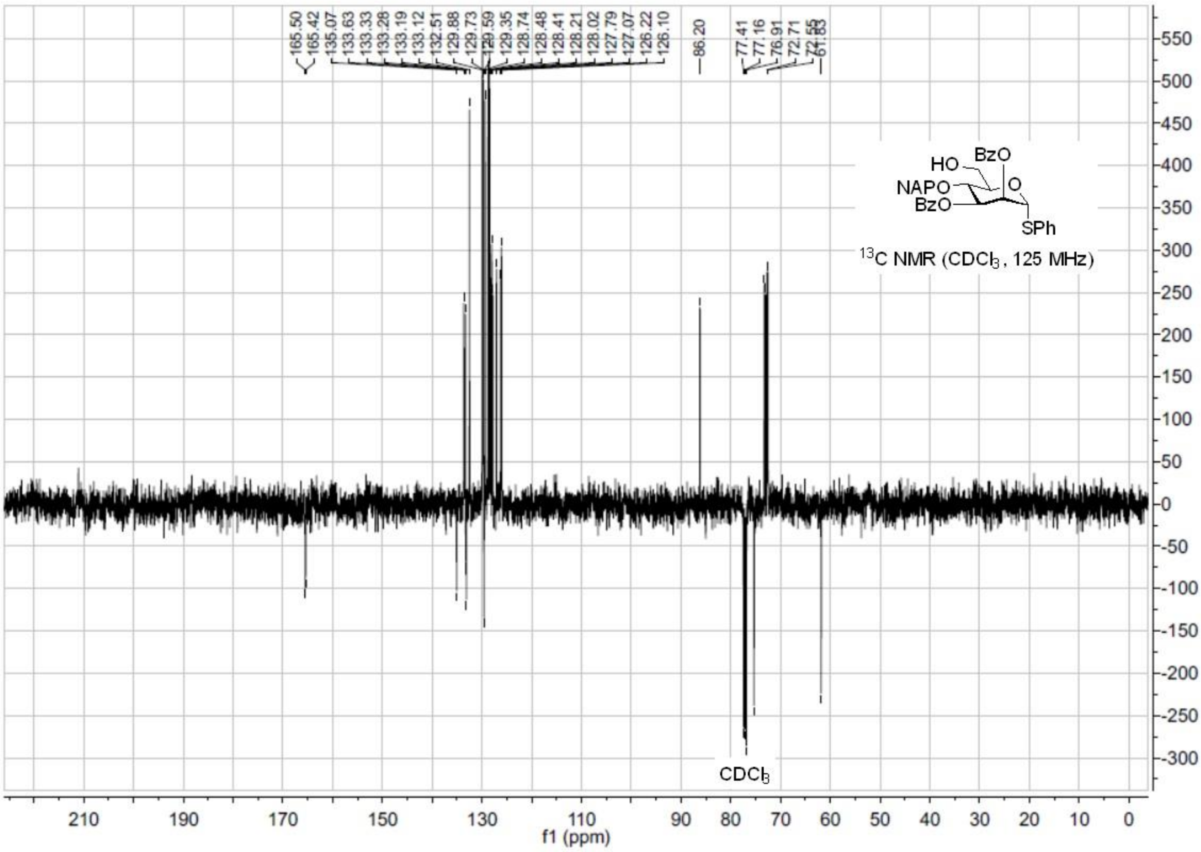

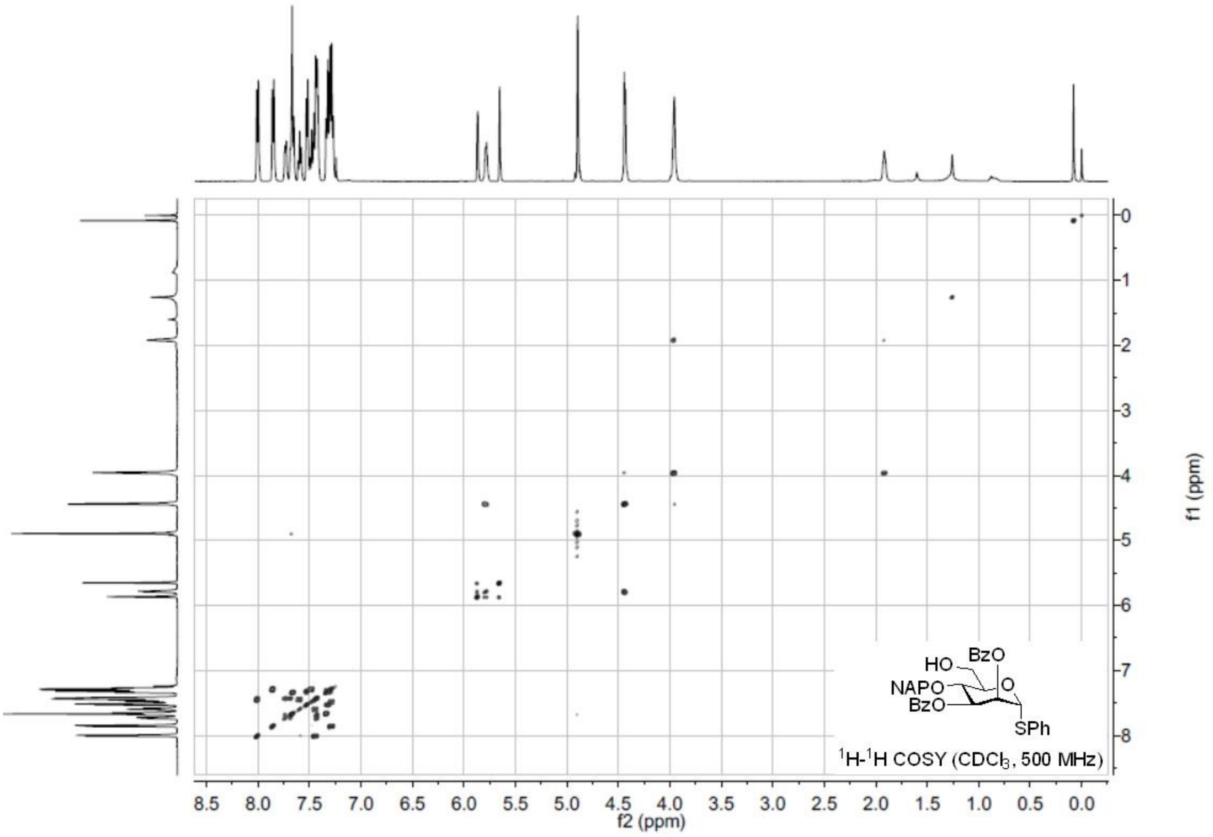
HO
BzO
NAPO
BzO
O
SPh
^{1}H-^{1}H COSY (CDCl$_3$, 500 MHz)
f2 (ppm)
f1 (ppm)

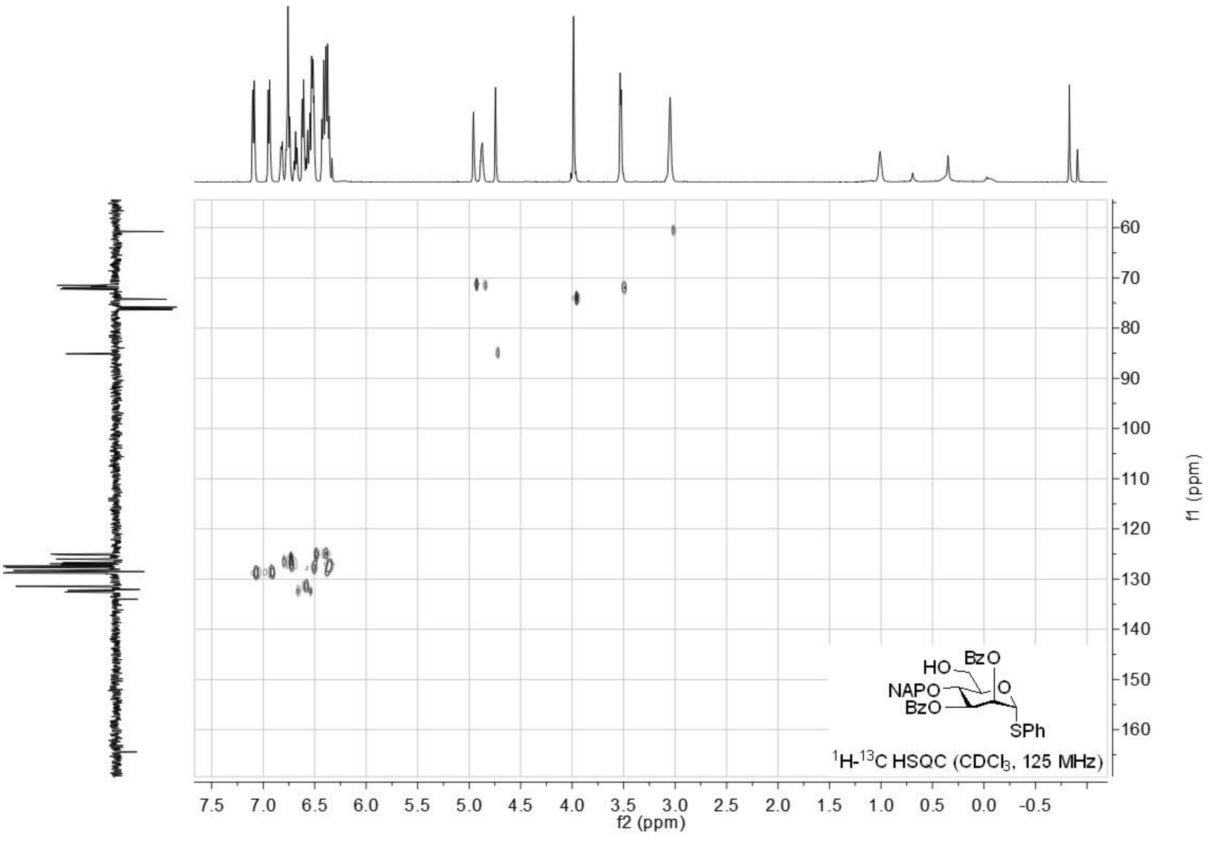
HO
BzO
NAPO
BzO
O
SPh
^{1}H-^{13}C HSQC (CDCl$_3$, 125 MHz)
f2 (ppm)
f1 (ppm)

^{1}H and ^{13}C NMR spectra of compound **36**

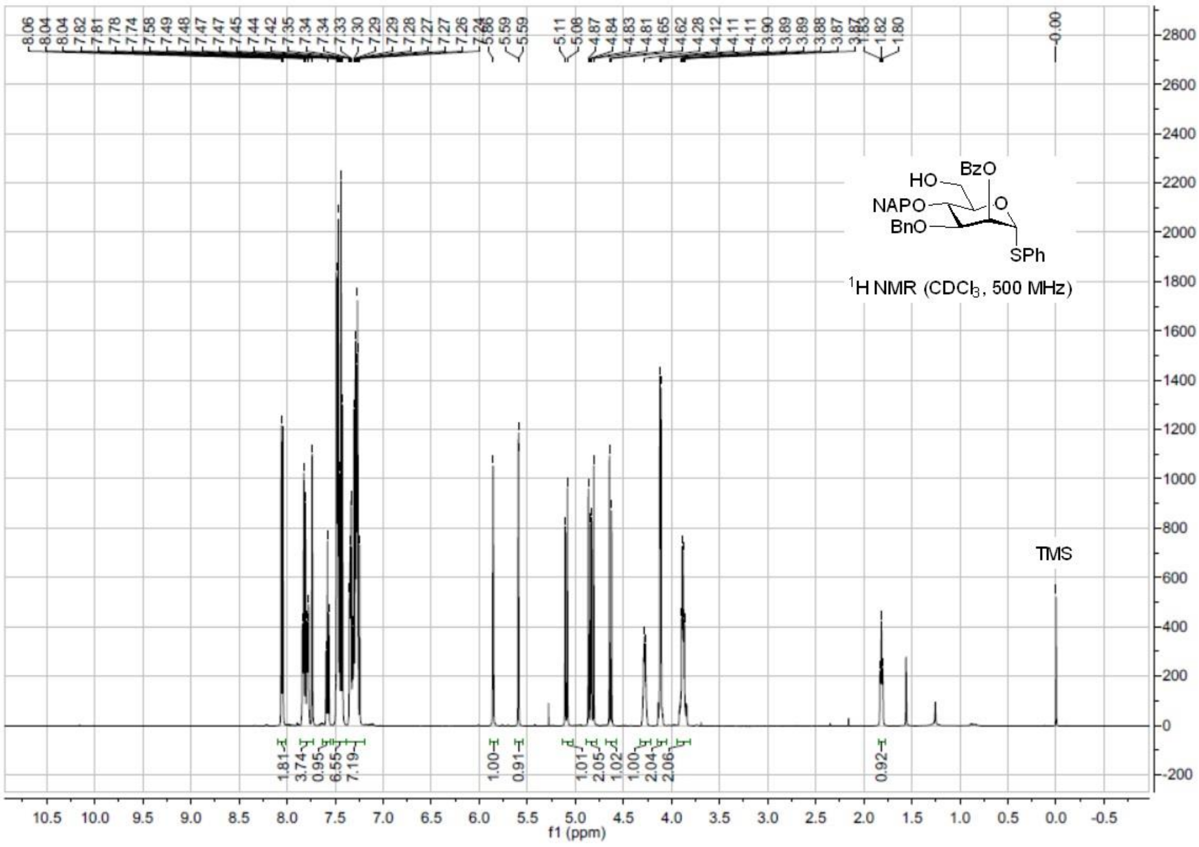

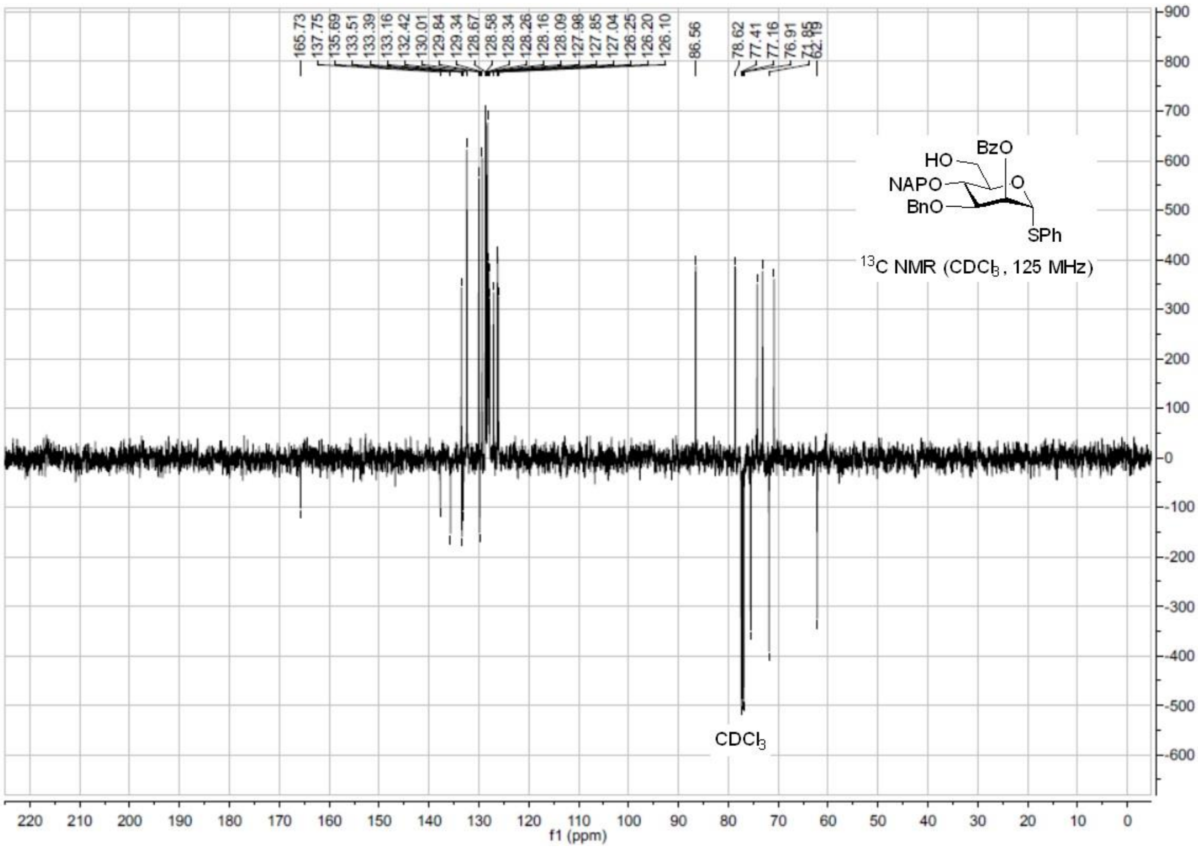

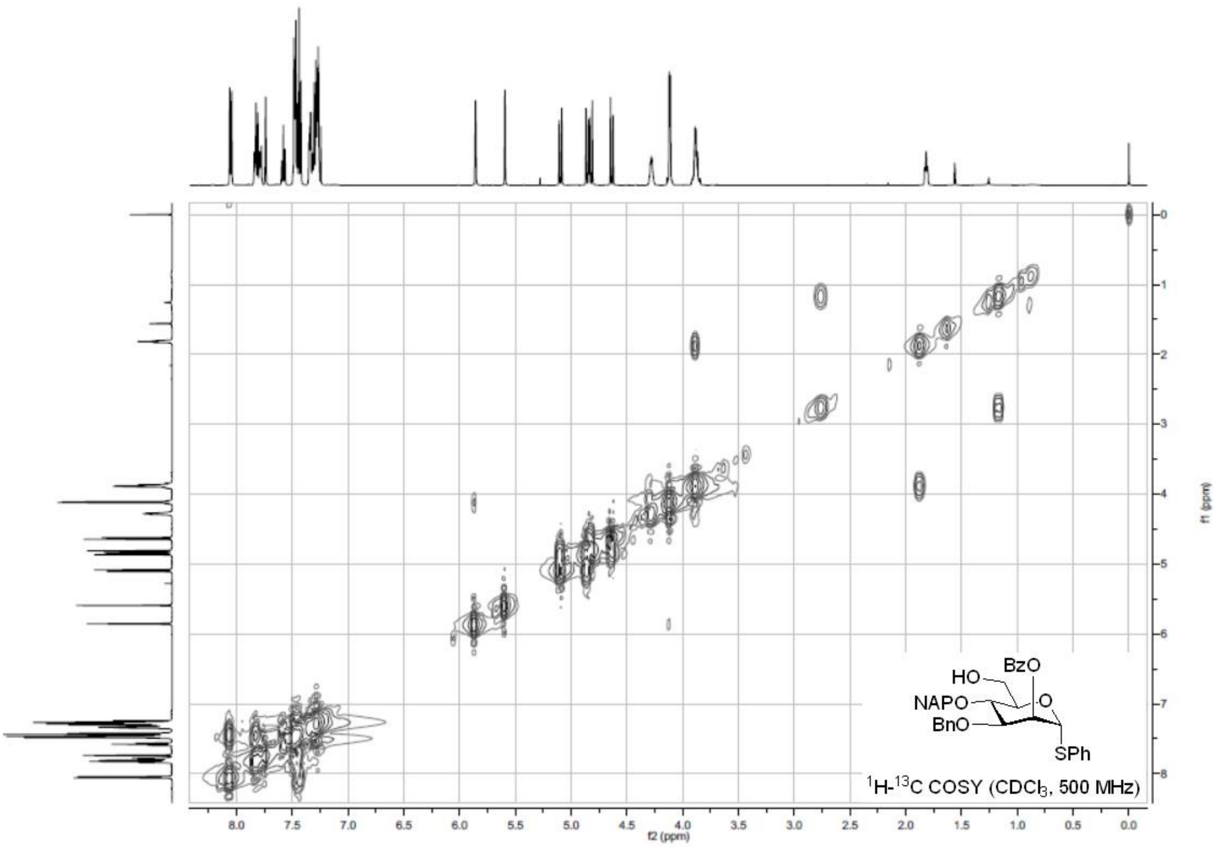
HO
BzO
NAPO
O
BnO
SPh
^{1}H-^{13}C COSY (CDCl$_3$, 500 MHz)
f2 (ppm)
f1 (ppm)

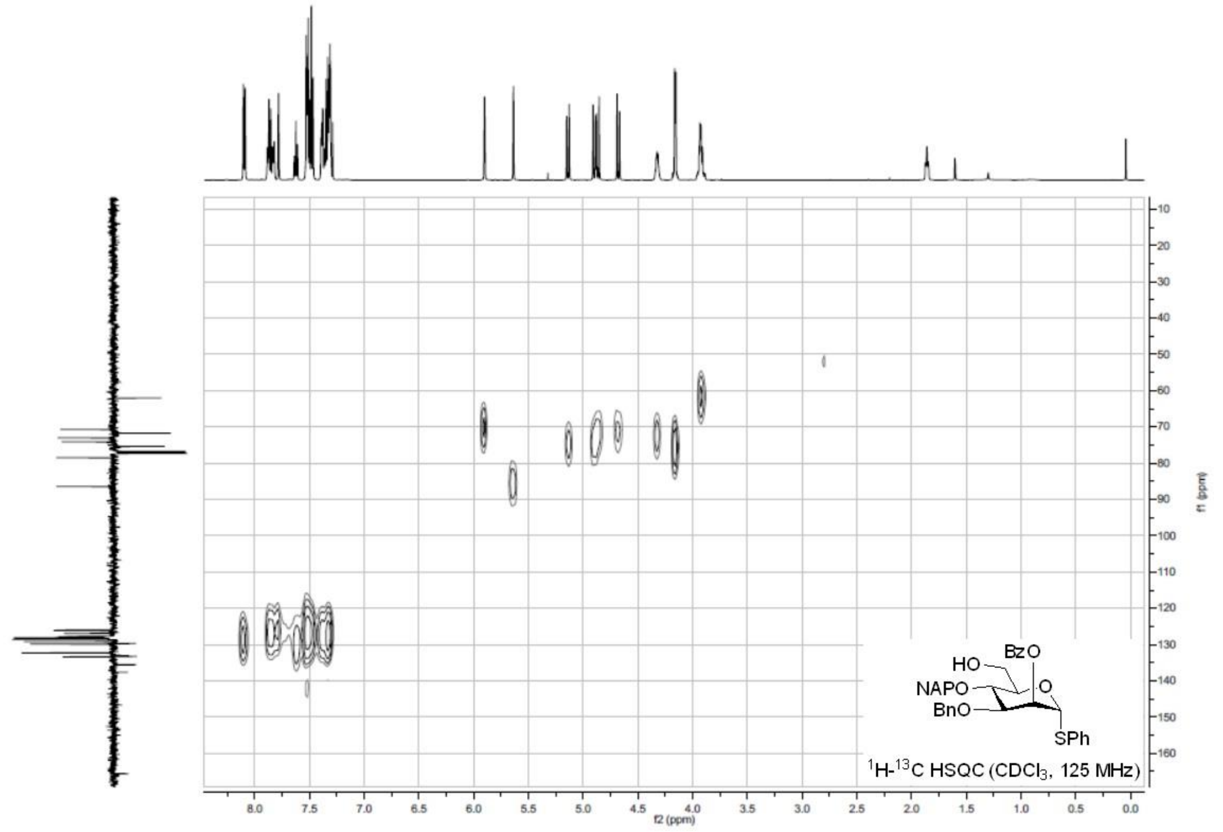
HO
BzO
NAPO
O
BnO
SPh
^{1}H-^{13}C HSQC (CDCl$_3$, 125 MHz)
f2 (ppm)
f1 (ppm)

^{1}H and ^{13}C NMR spectra of compound **37**

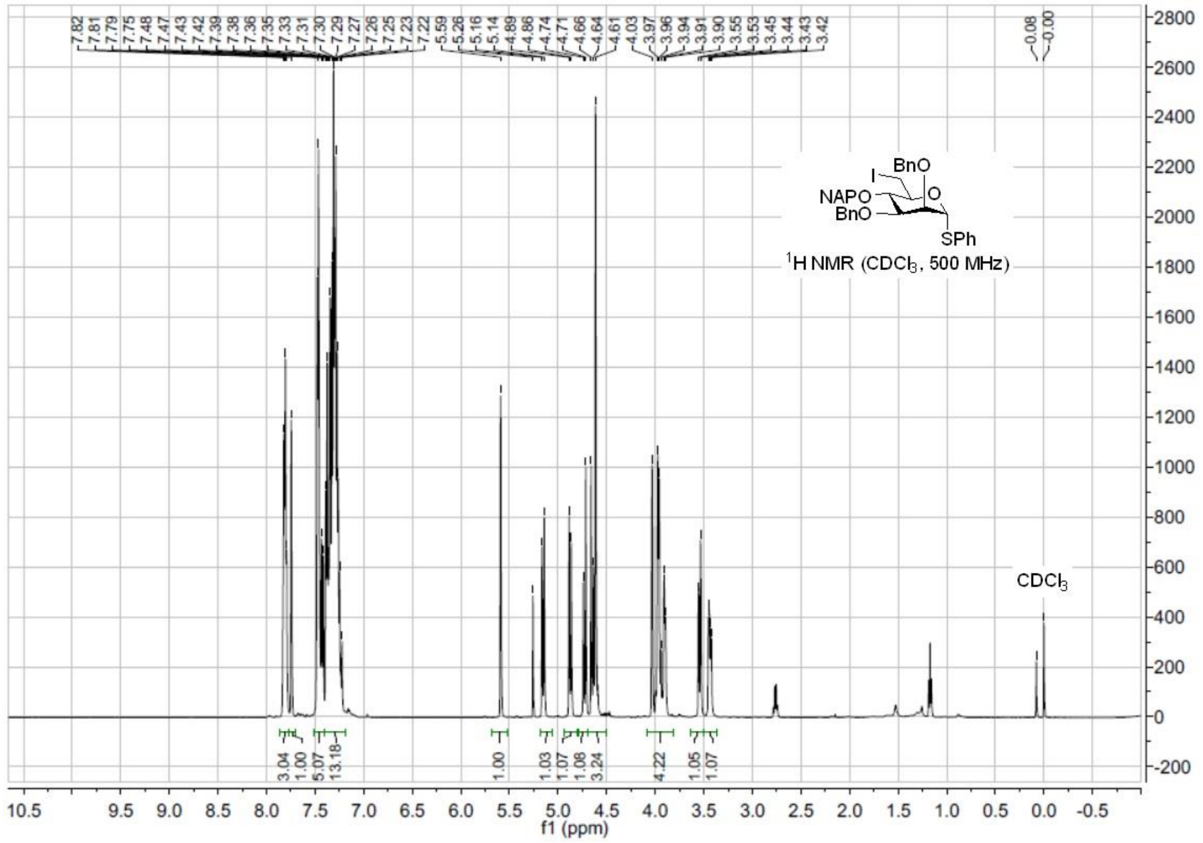

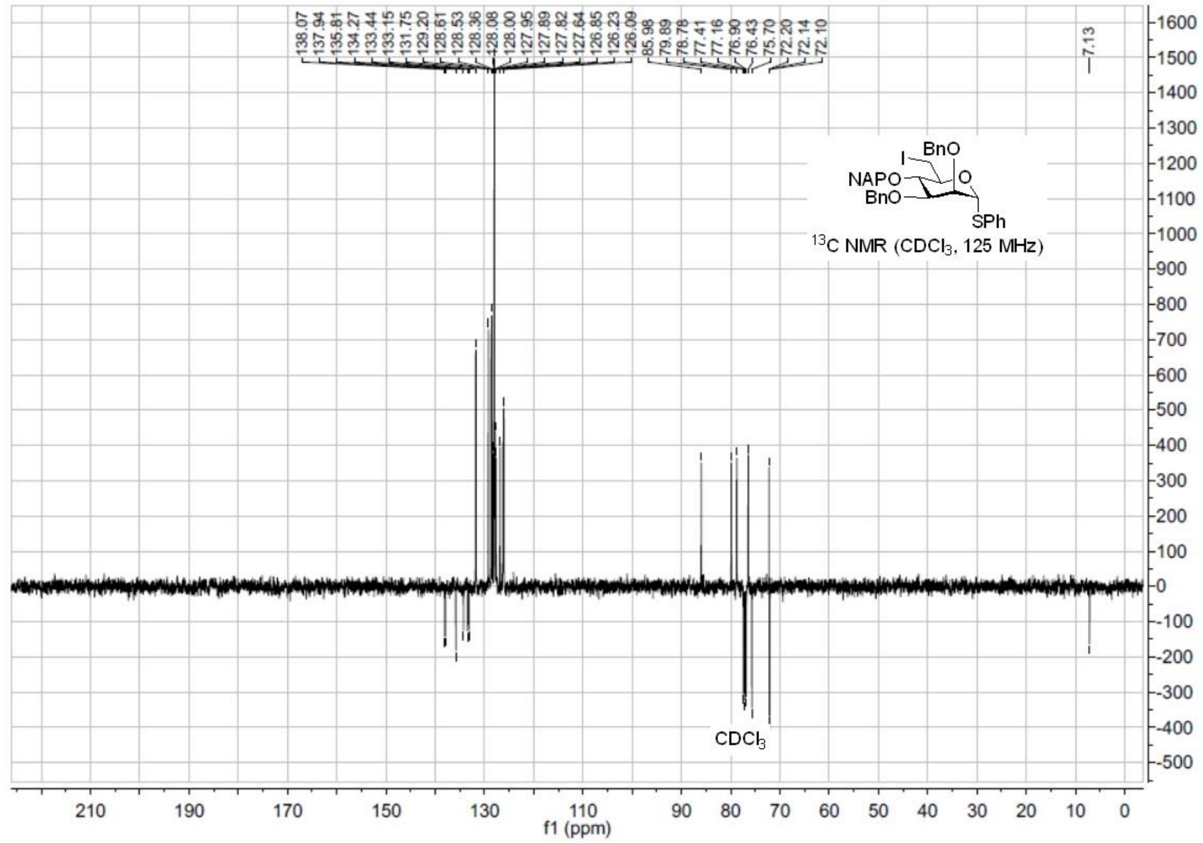

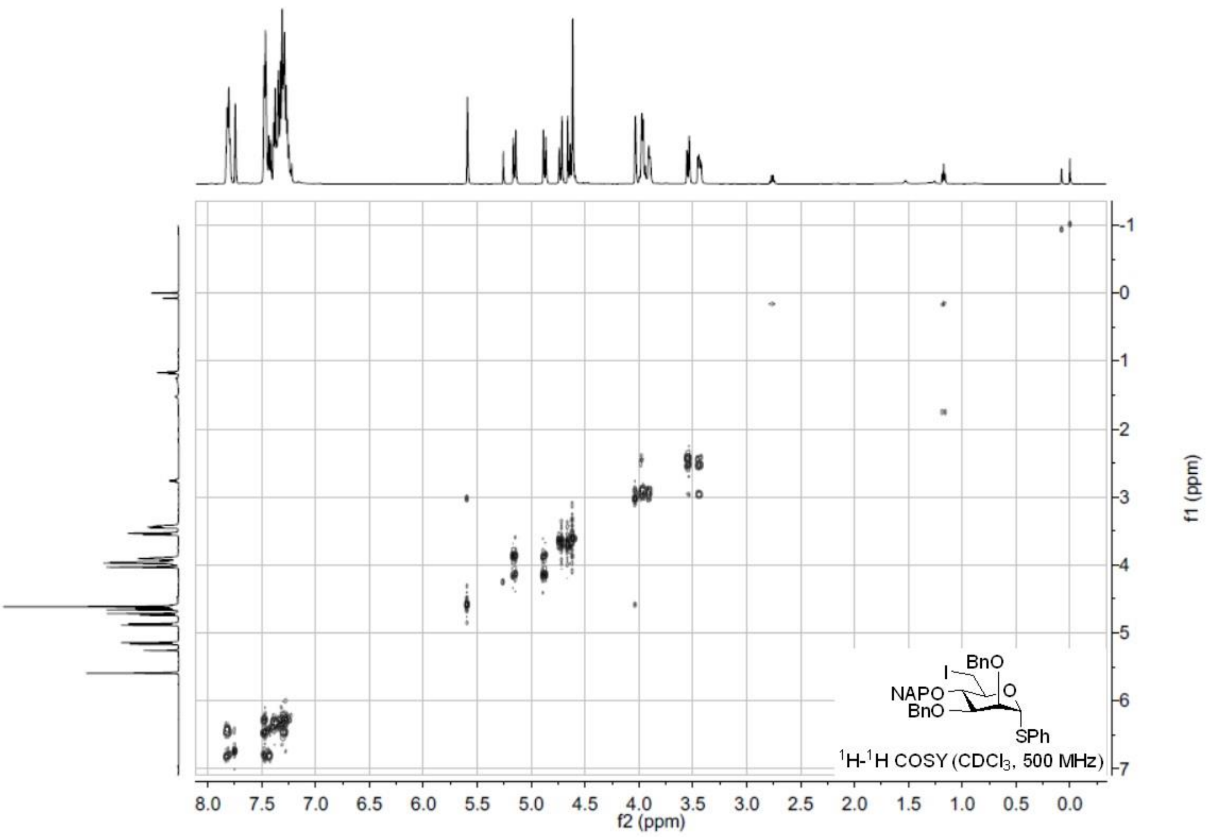
BnO
I
O
NAPO
BnO
SPh
1H-1H COSY (CDCl3, 500 MHz)
f1 (ppm)
f2 (ppm)

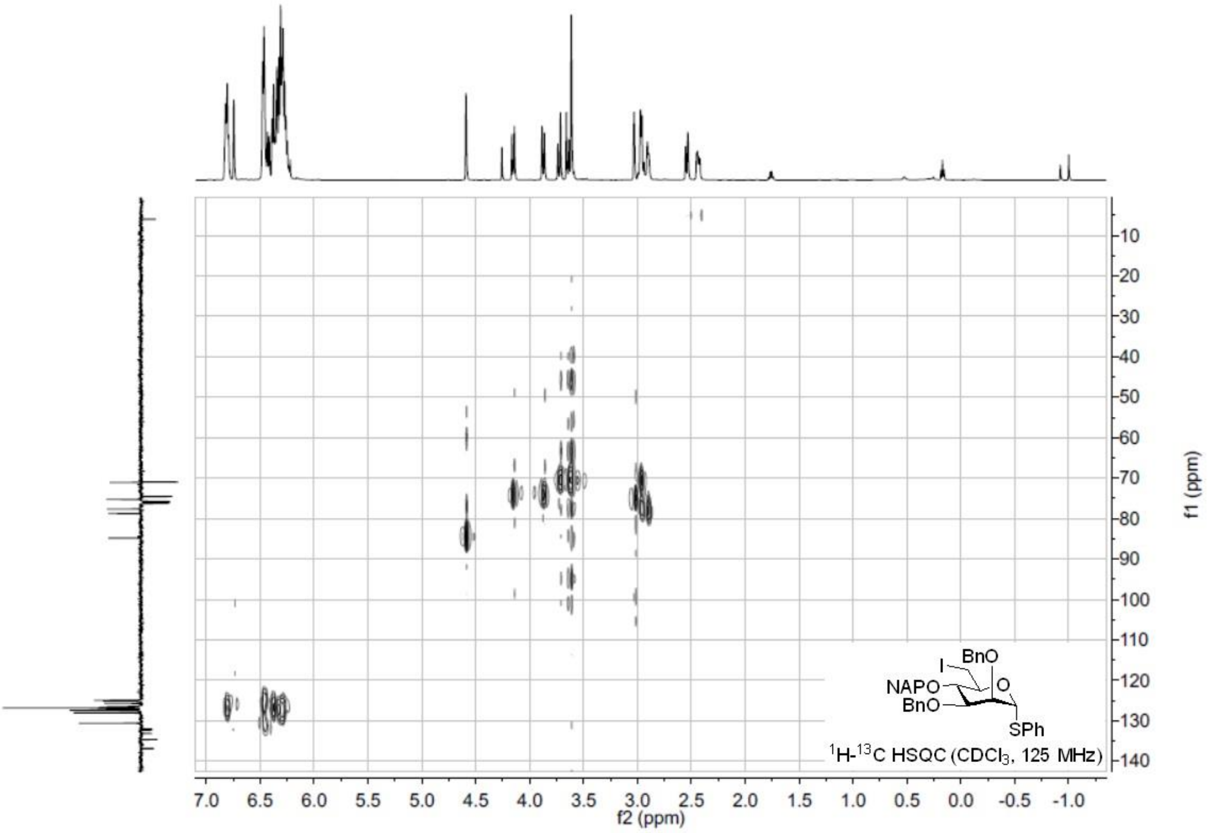
BnO
I
O
NAPO
BnO
SPh
1H-13C HSQC (CDCl3, 125 MHz)
f1 (ppm)
f2 (ppm)

^{1}H and ^{13}C NMR spectra of compound **38**

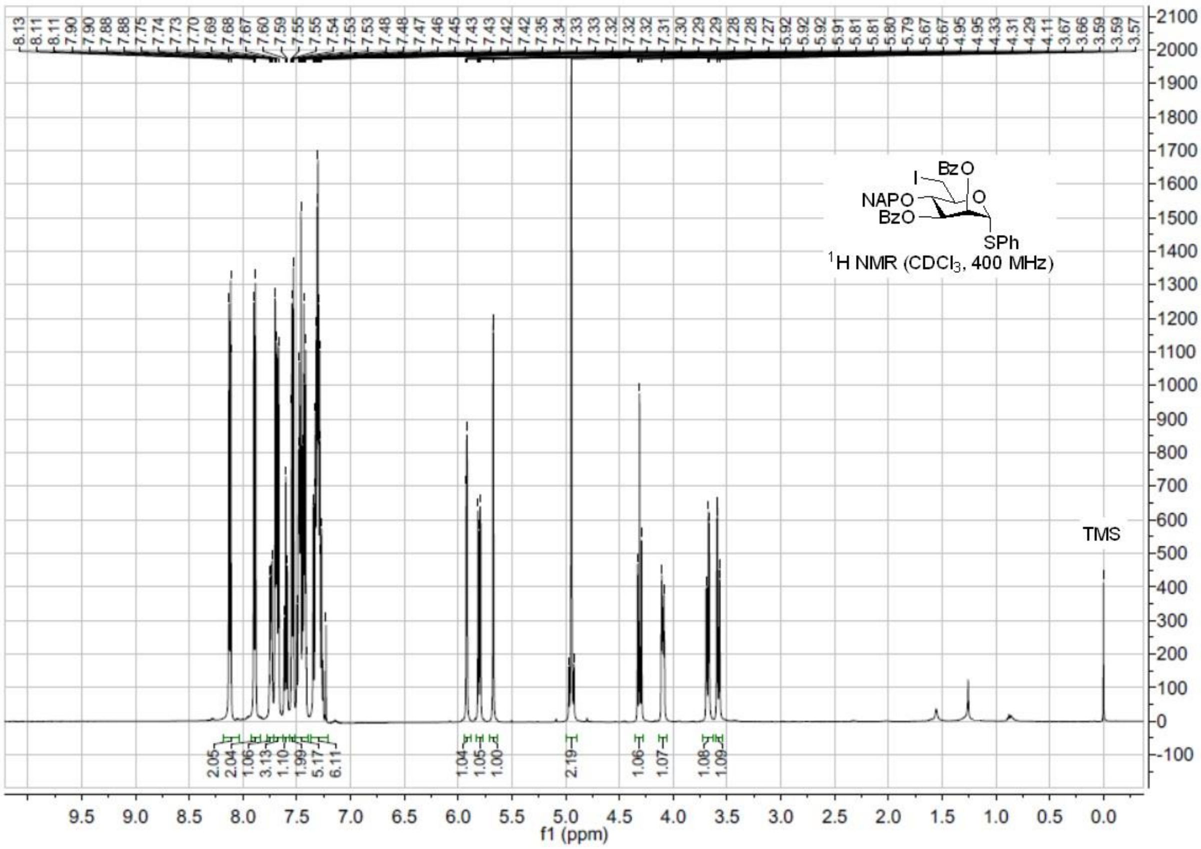

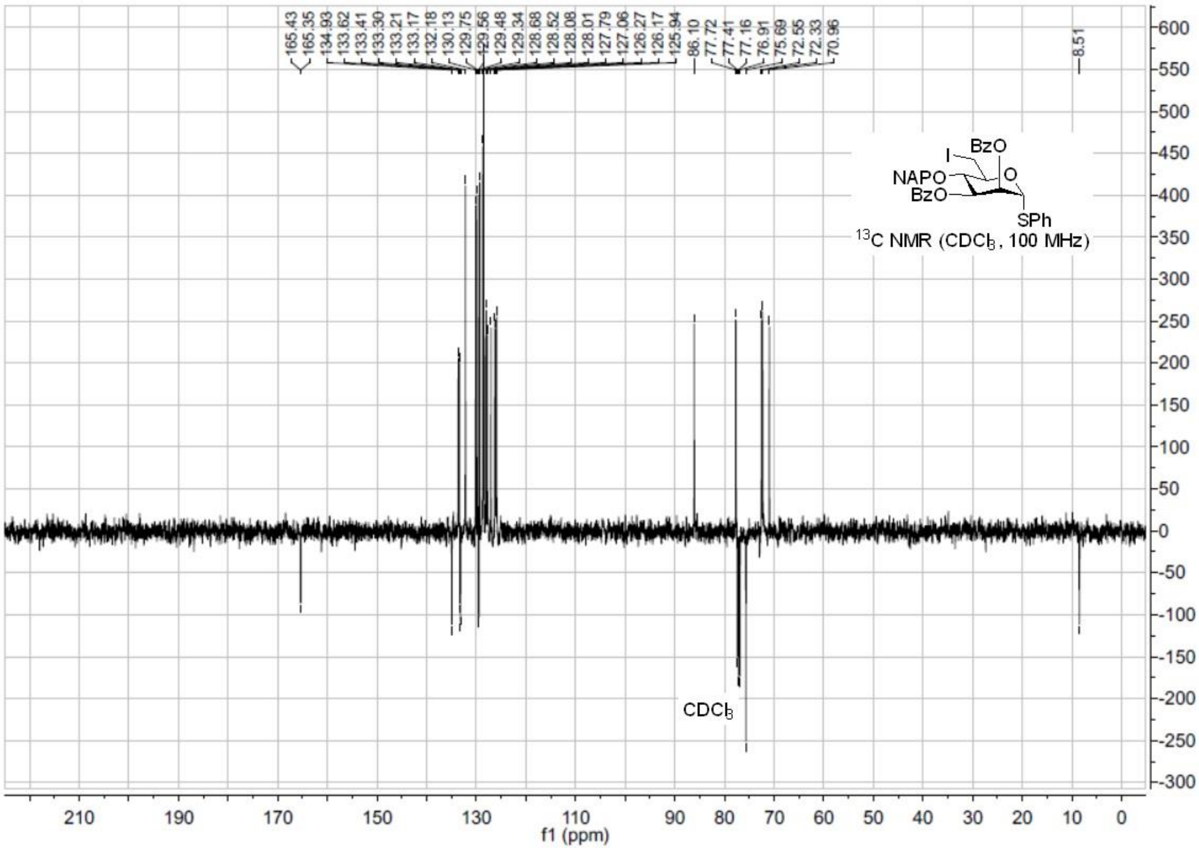

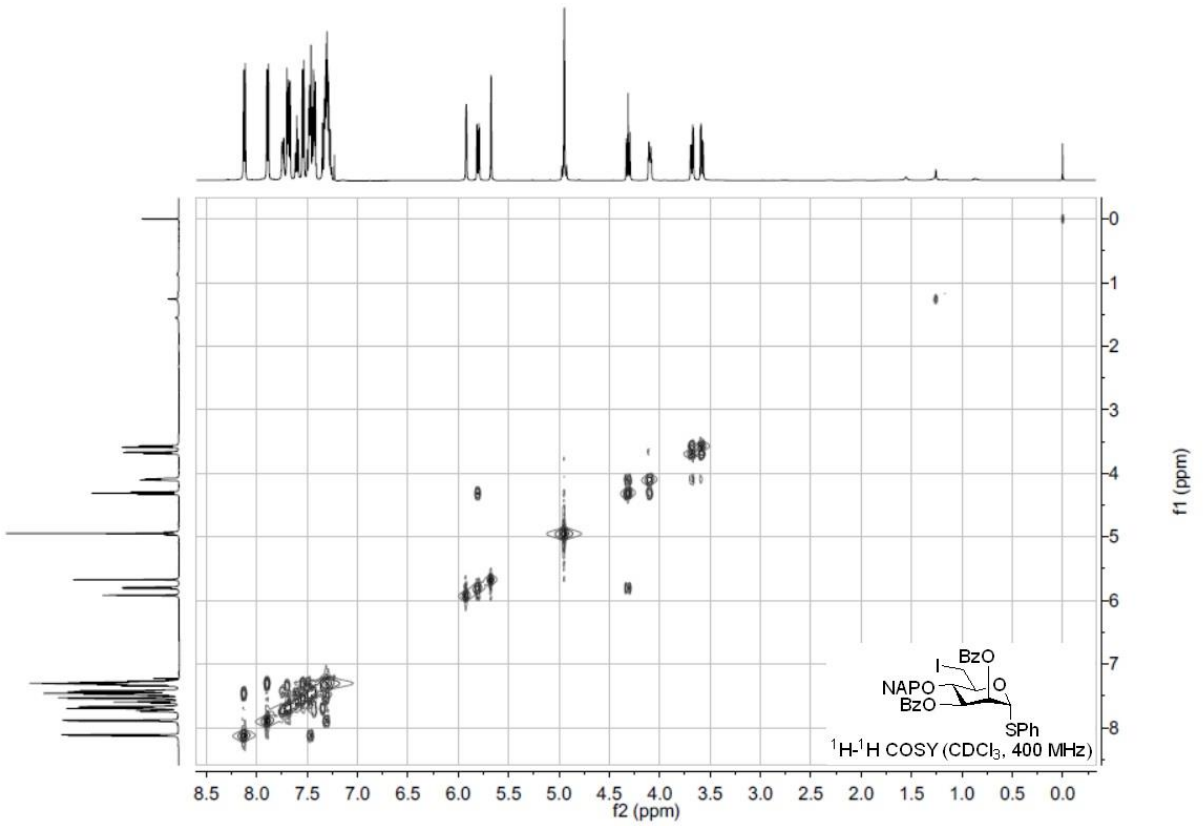
BzO
I
NAPO
O
BzO
SPh
^{1}H-^{1}H COSY (CDCl$_3$, 400 MHz)
f1 (ppm)
f2 (ppm)

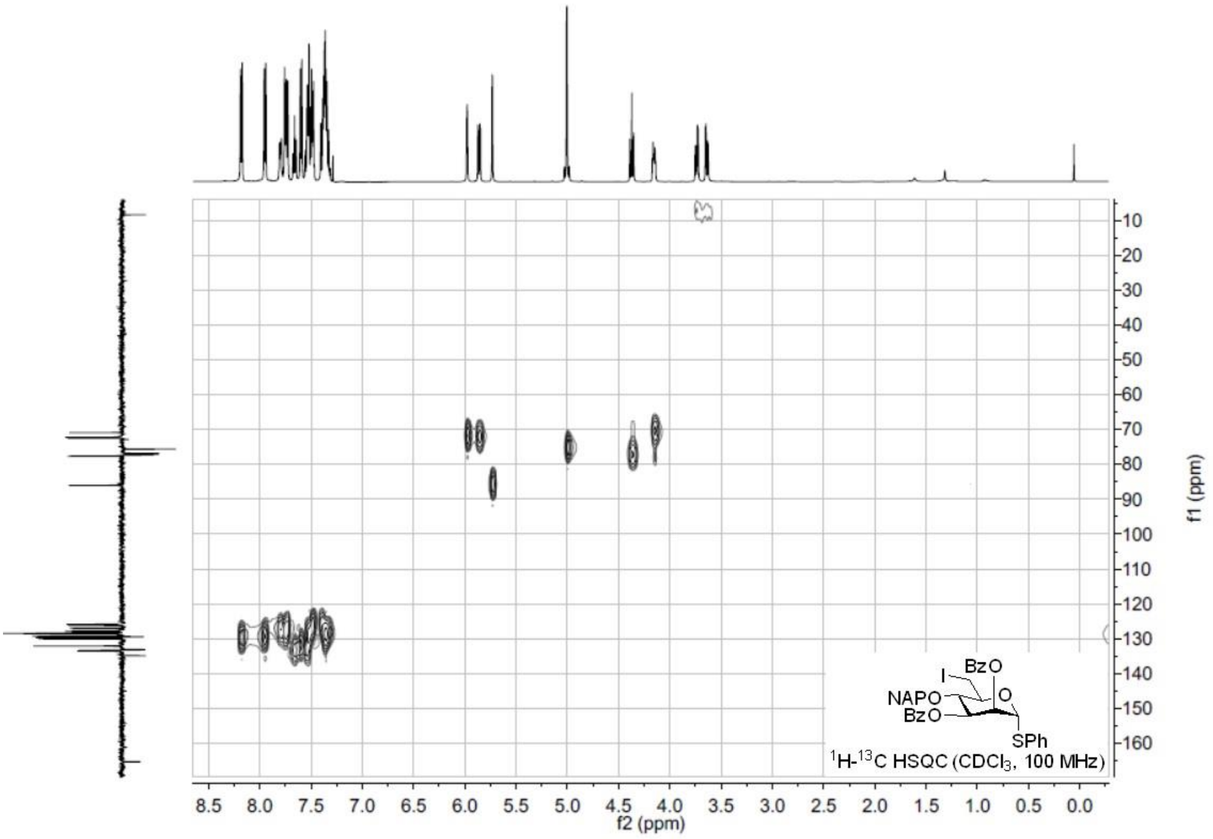
BzO
I
NAPO
O
BzO
SPh
^{1}H-^{13}C HSQC (CDCl$_3$, 100 MHz)
f1 (ppm)
f2 (ppm)

^{1}H and ^{13}C NMR spectra of compound **39**

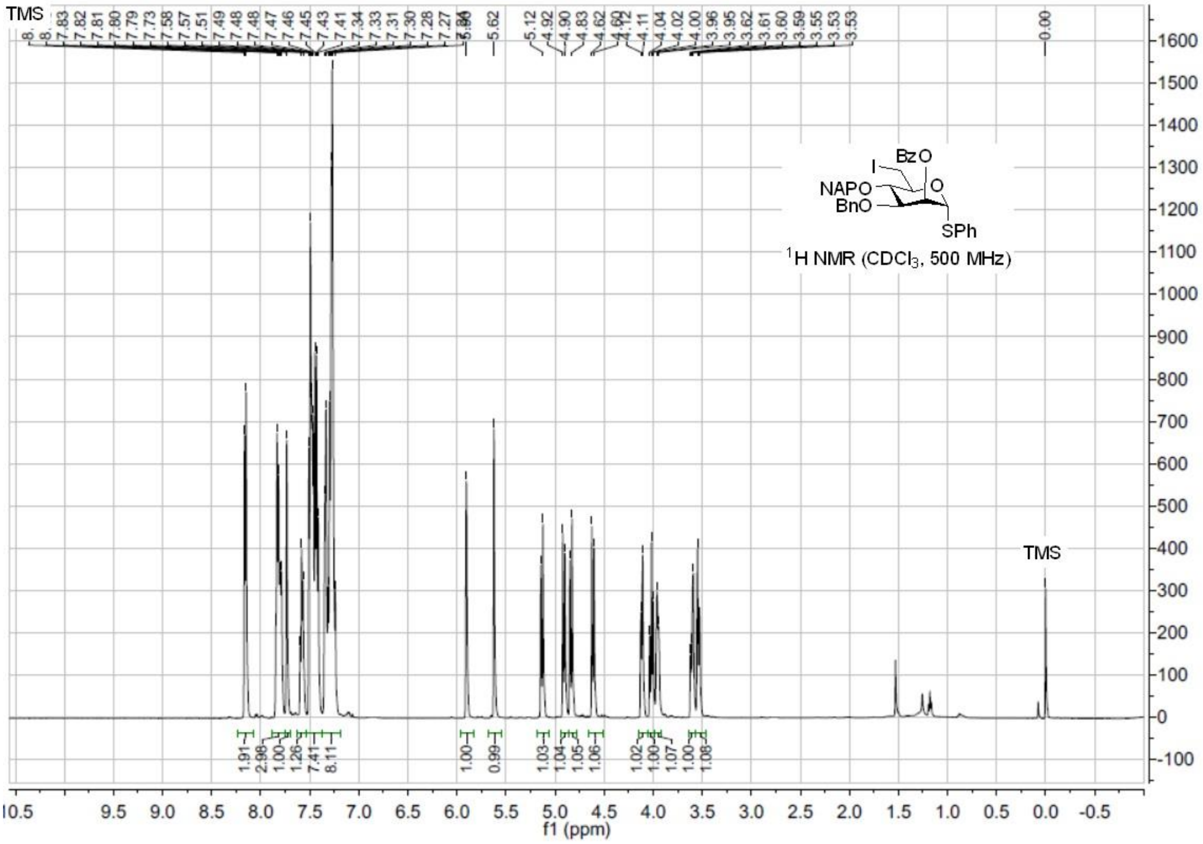

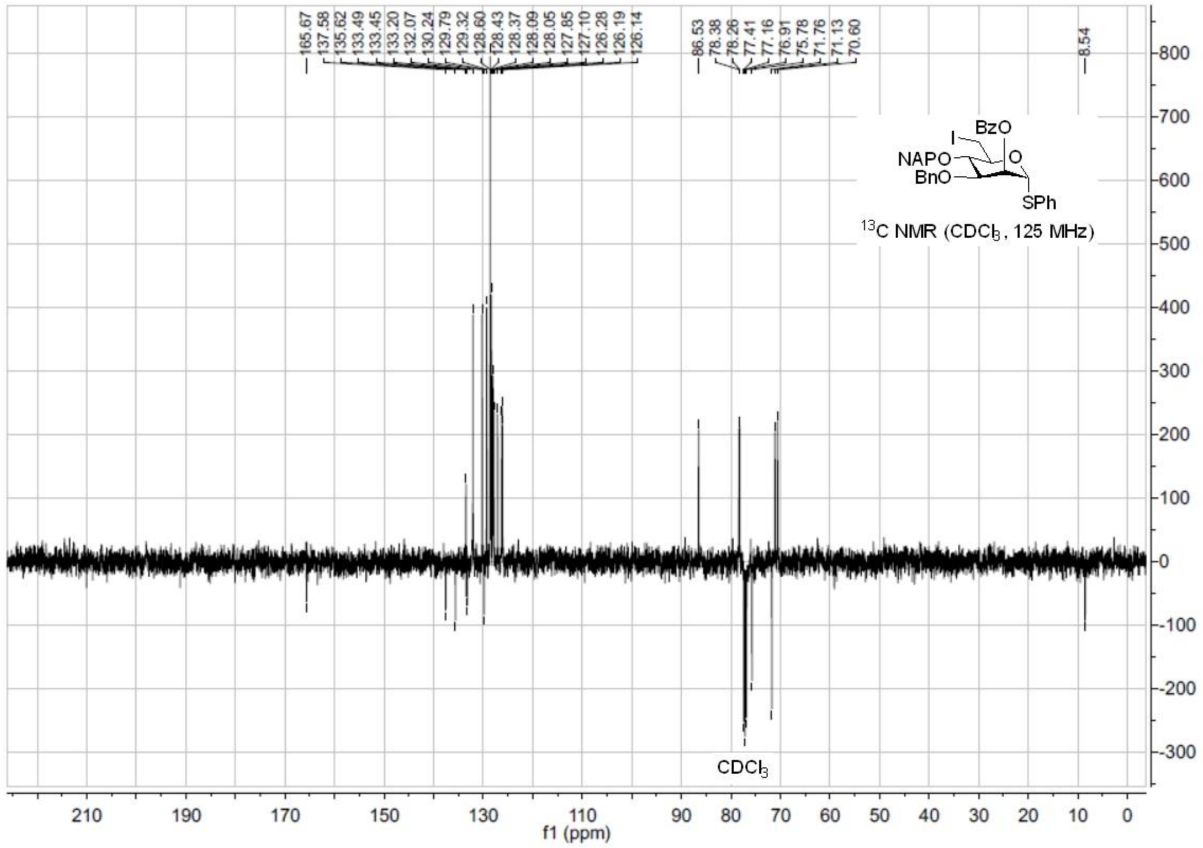

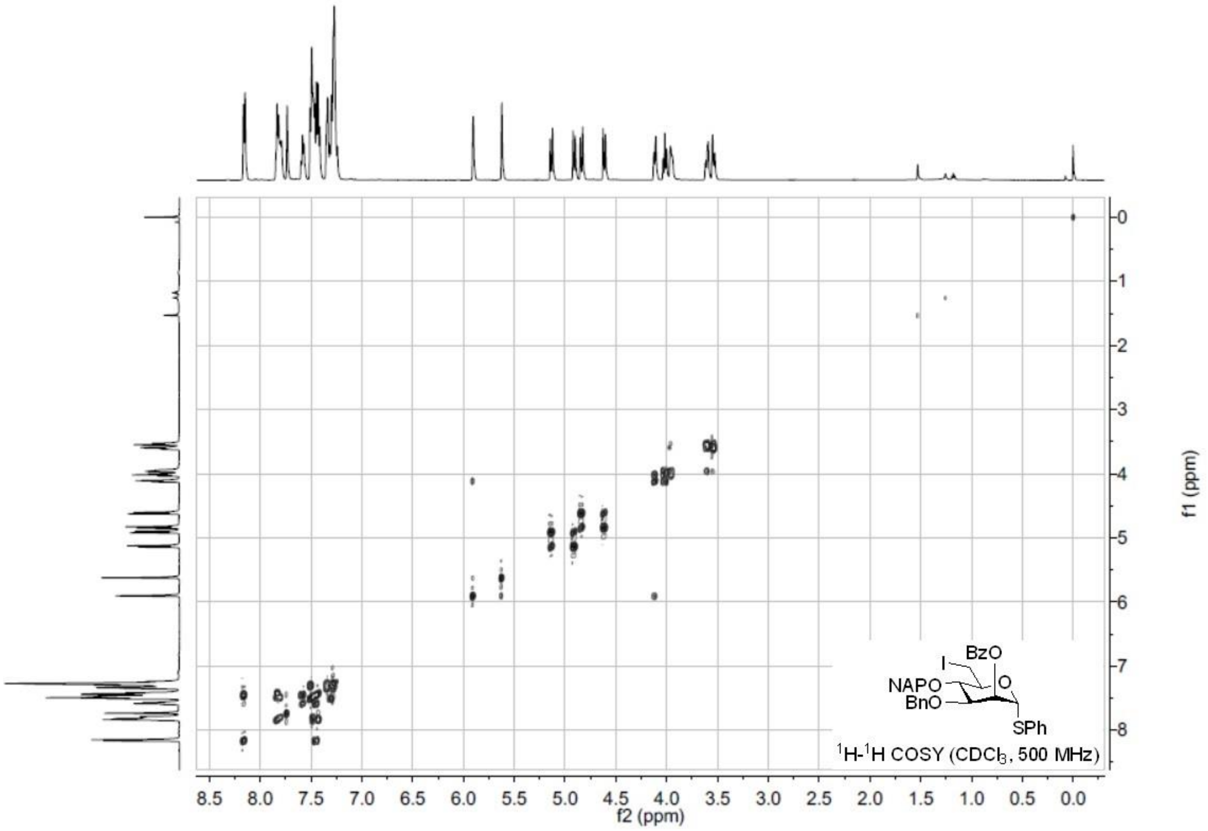
BzO
I
NAPO
BnO
O
SPh
^{1}H-^{1}H COSY (CDCl$_3$, 500 MHz)
f2 (ppm)
f1 (ppm)

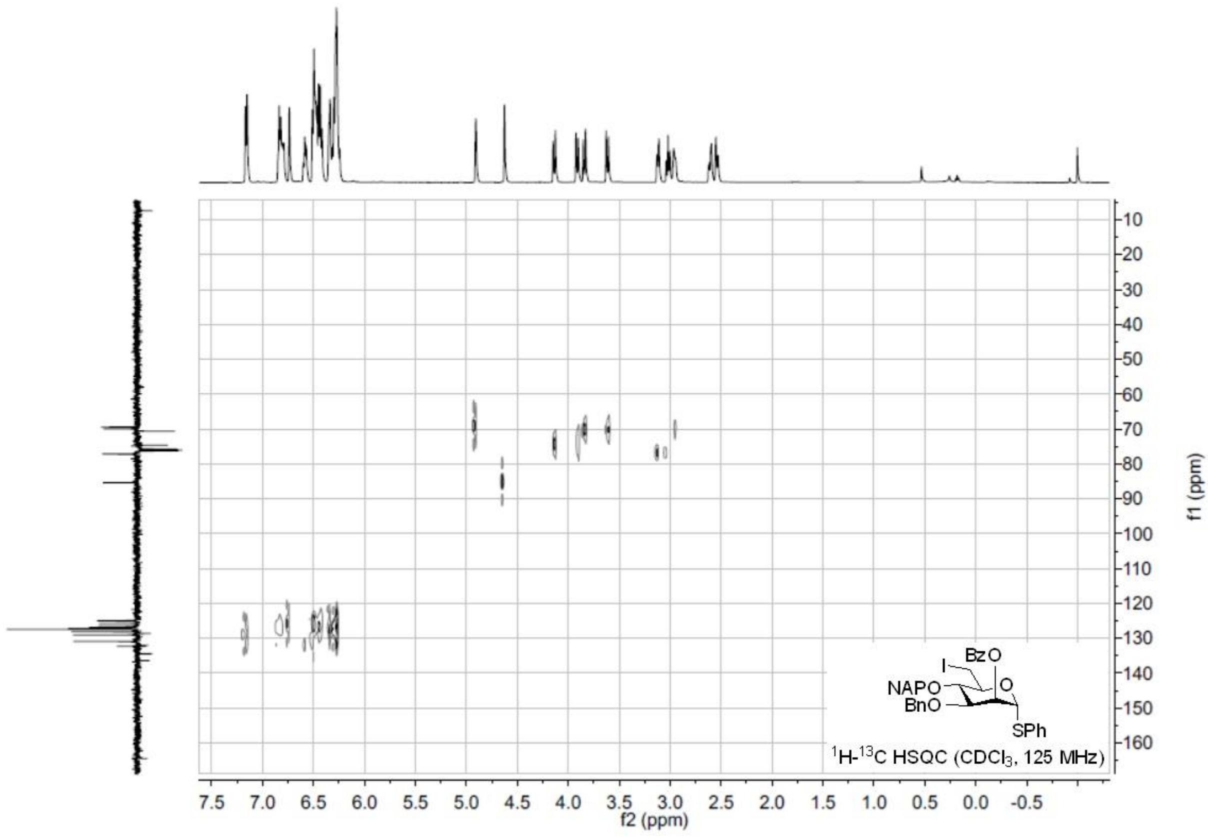
BzO
I
NAPO
BnO
O
SPh
^{1}H-^{13}C HSQC (CDCl$_3$, 125 MHz)
f2 (ppm)
f1 (ppm)

^{1}H and ^{13}C NMR spectra of compound **40**

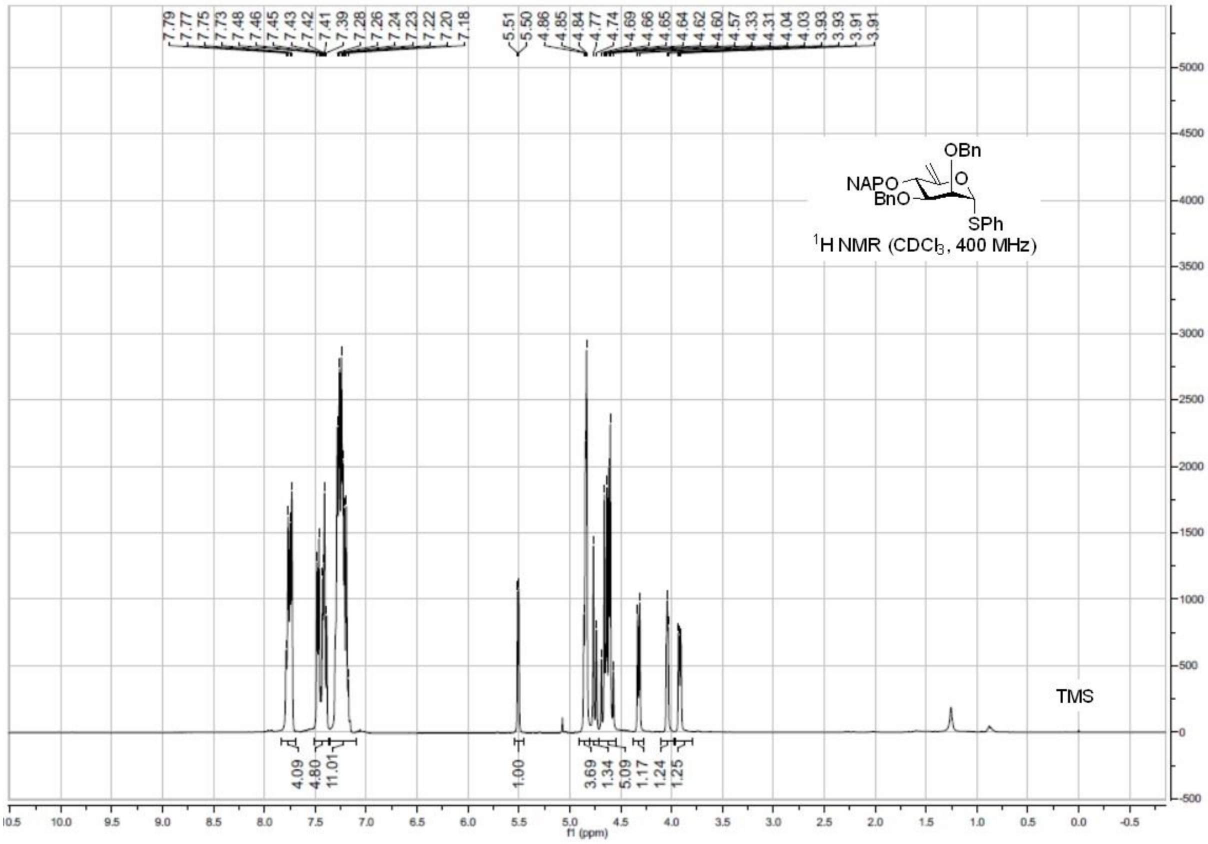

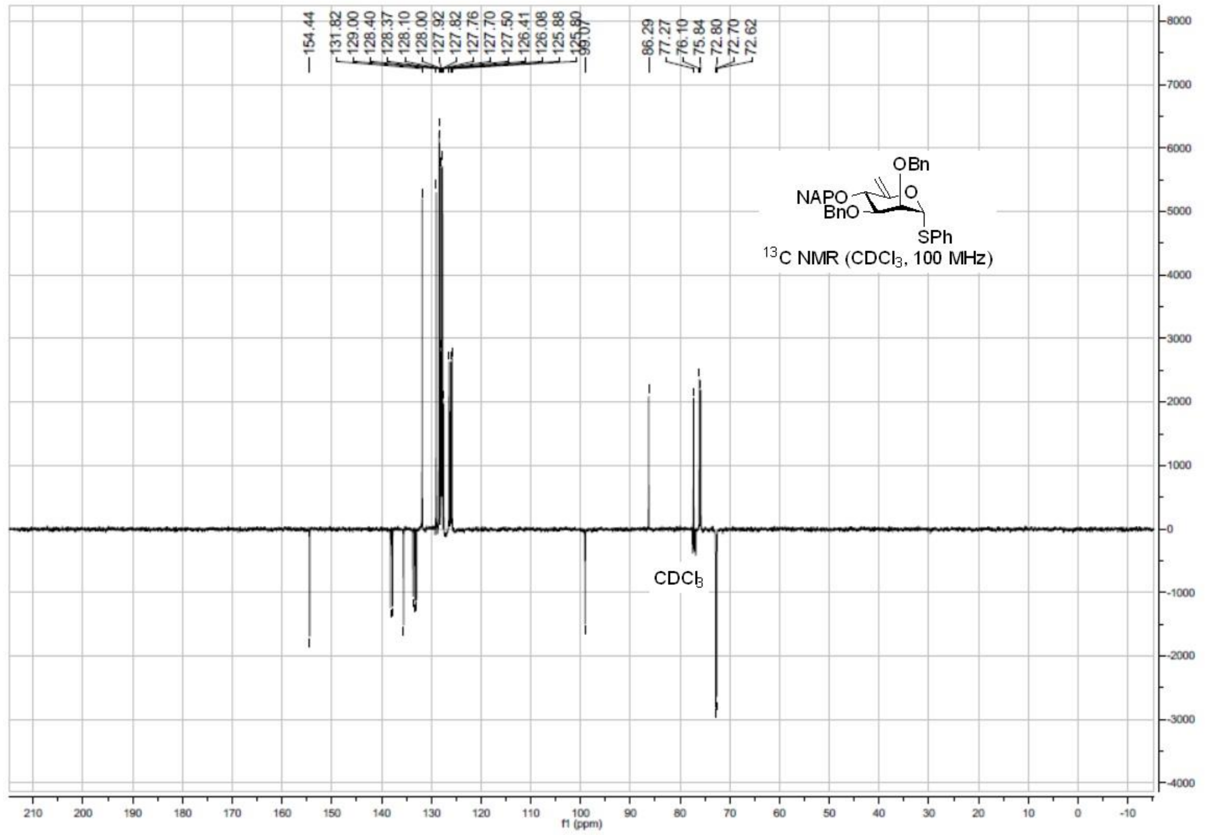

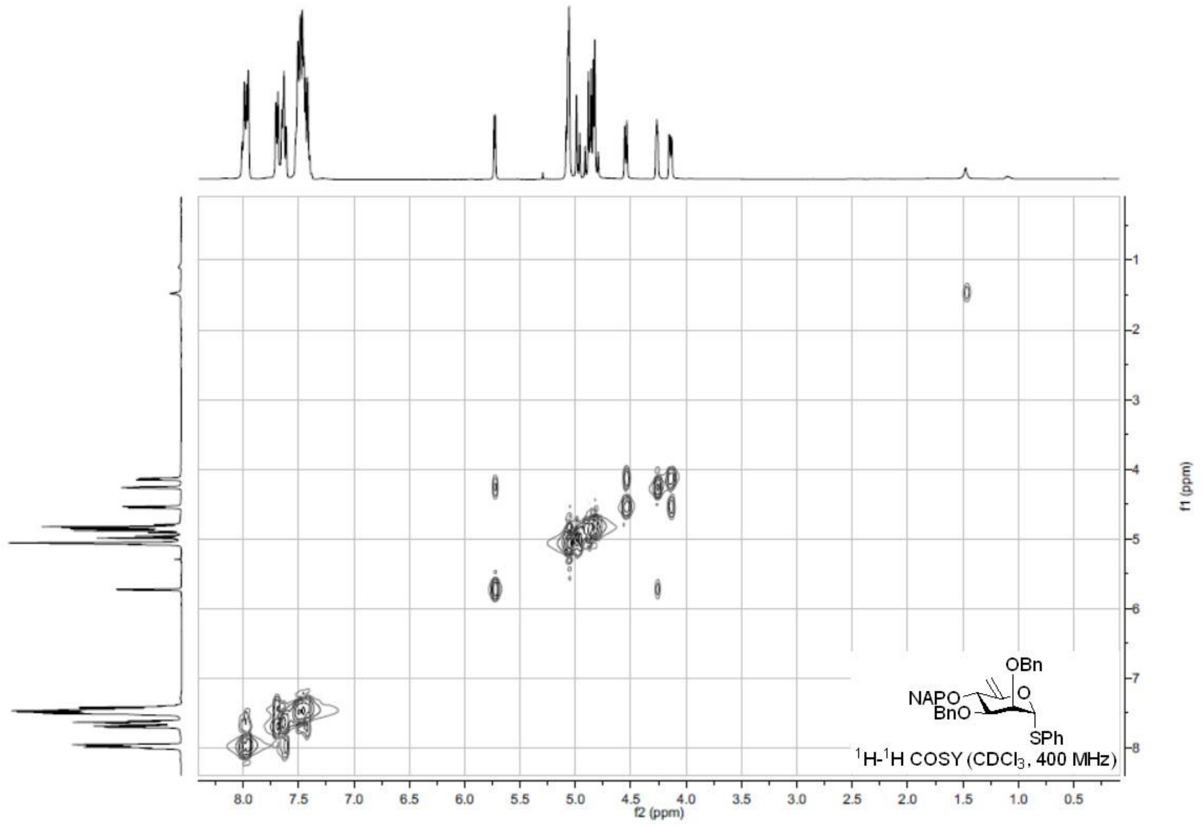
OBn
NAPO
BnO
O
SPh
^{1}H-^{1}H COSY (CDCl$_3$, 400 MHz)
f2 (ppm)
f1 (ppm)

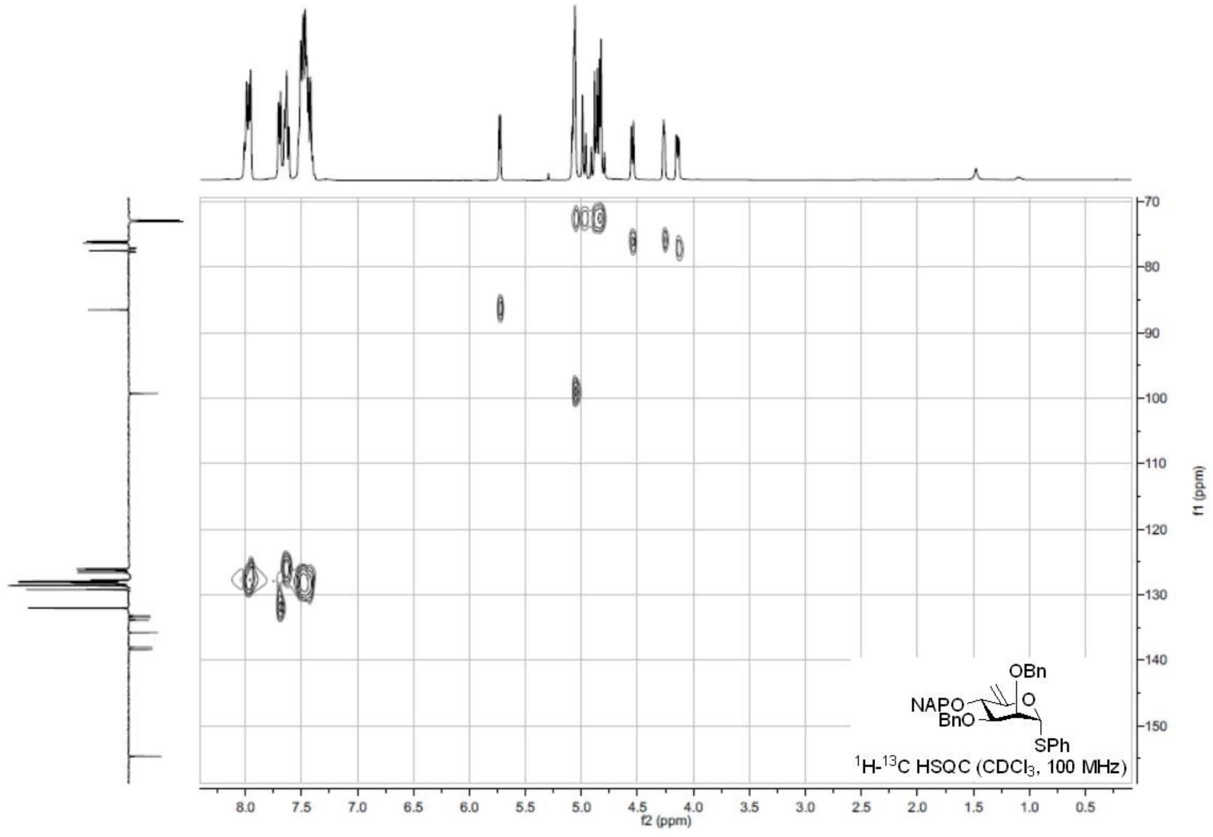
OBn
NAPO
BnO
O
SPh
^{1}H-^{13}C HSQC (CDCl$_3$, 100 MHz)
f2 (ppm)
f1 (ppm)

^{1}H and ^{13}C NMR spectra of compound **41**

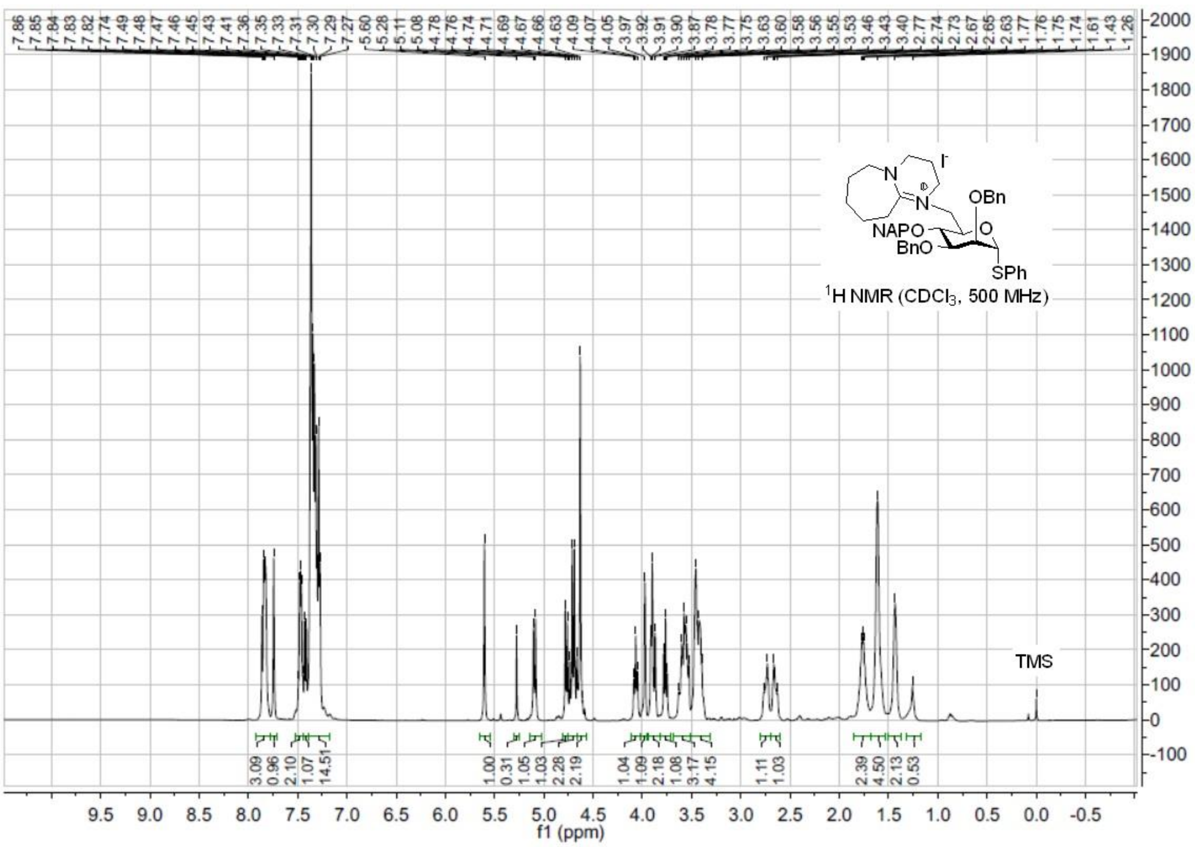

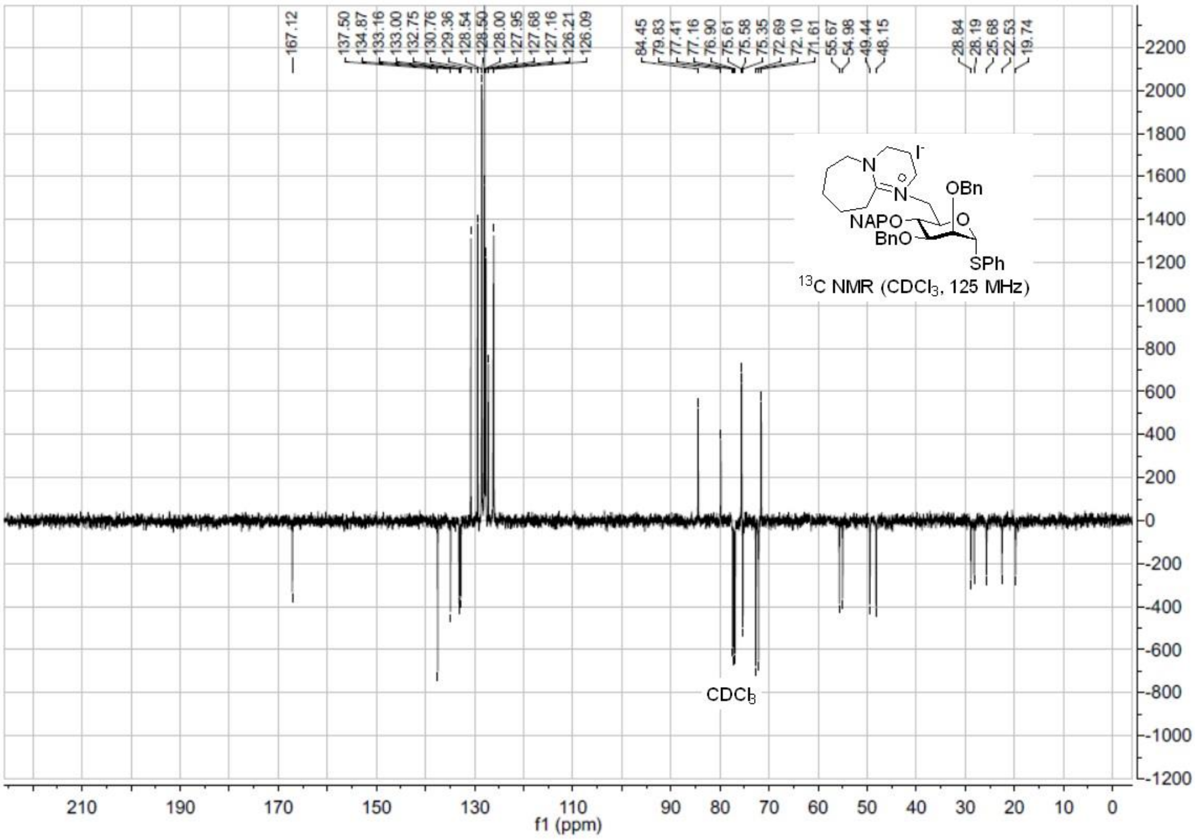

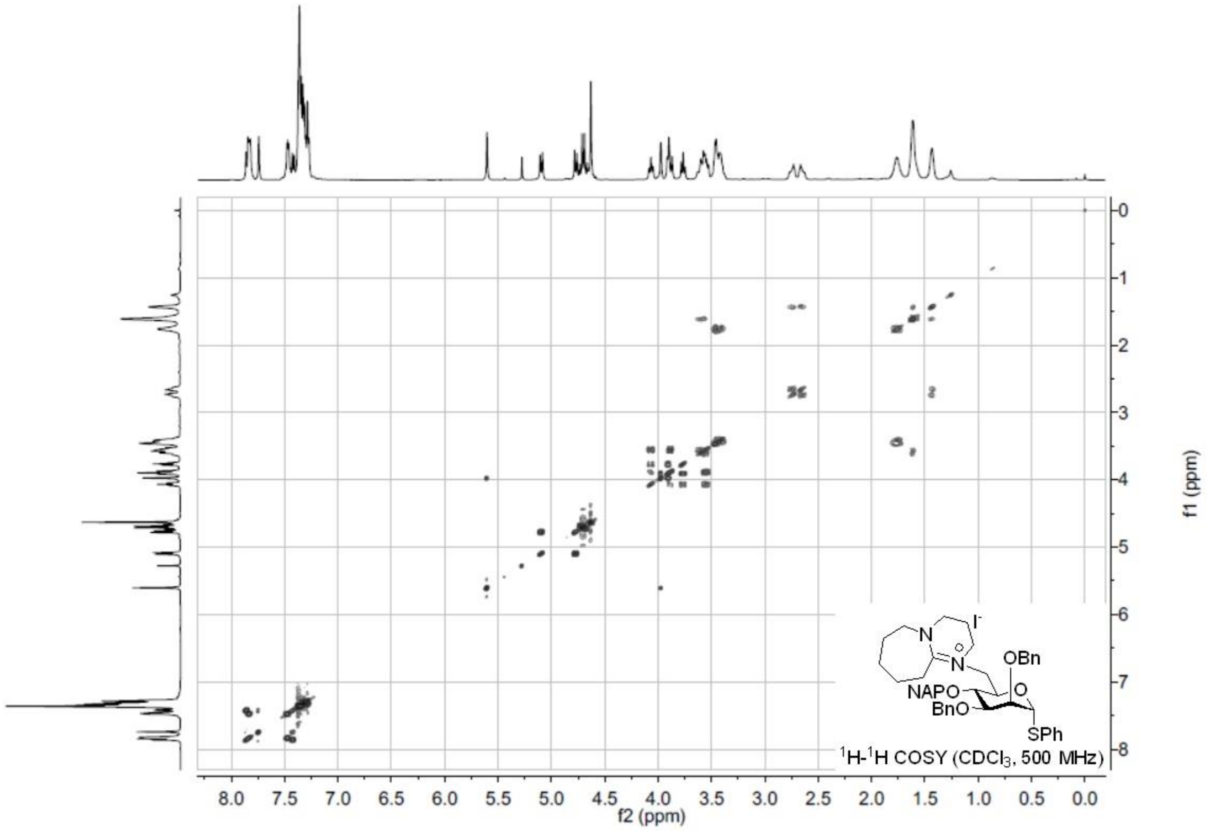

^{1}H-^{1}H COSY (CDCl3, 500 MHz)
NAPO
BnO
OBn
SPh
f2 (ppm)
f1 (ppm)

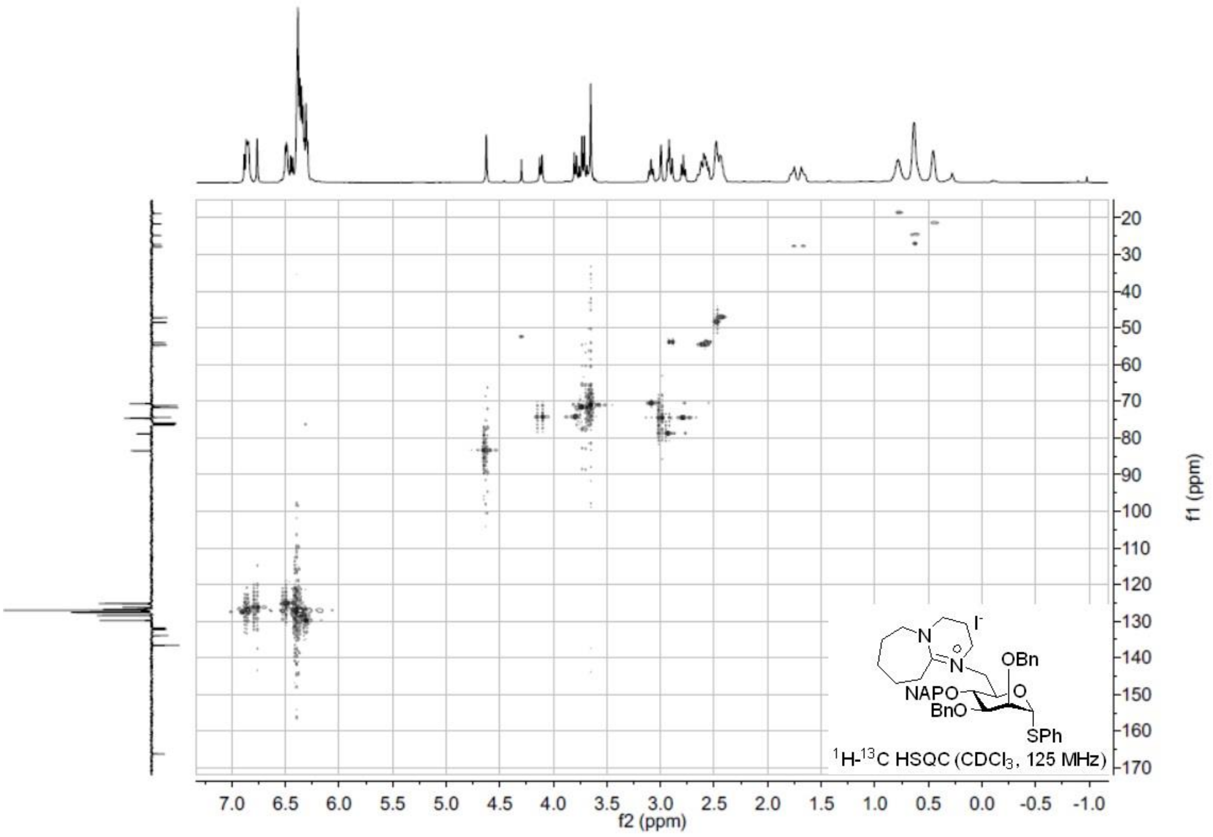

^{1}H-^{13}C HSQC (CDCl3, 125 MHz)
NAPO
BnO
OBn
SPh
f2 (ppm)
f1 (ppm)

^{1}H and ^{13}C NMR spectra of compound **42**

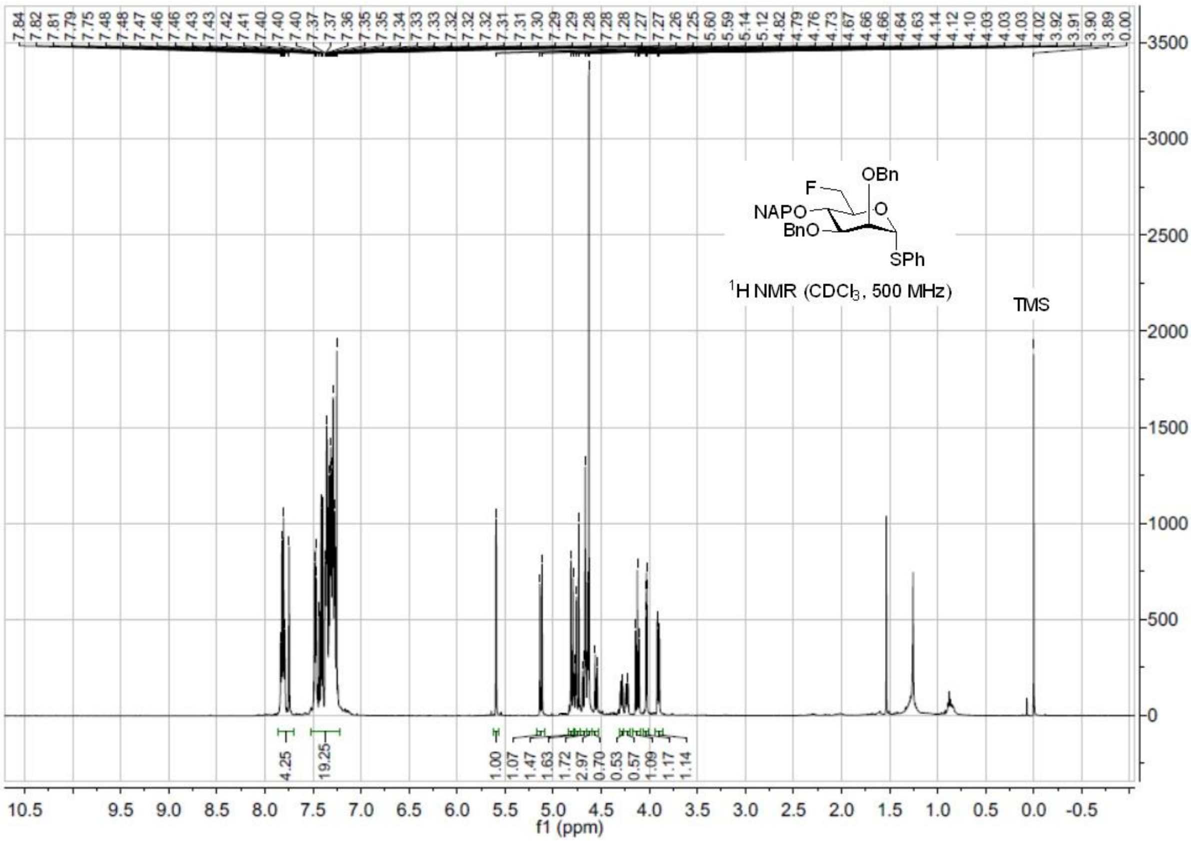

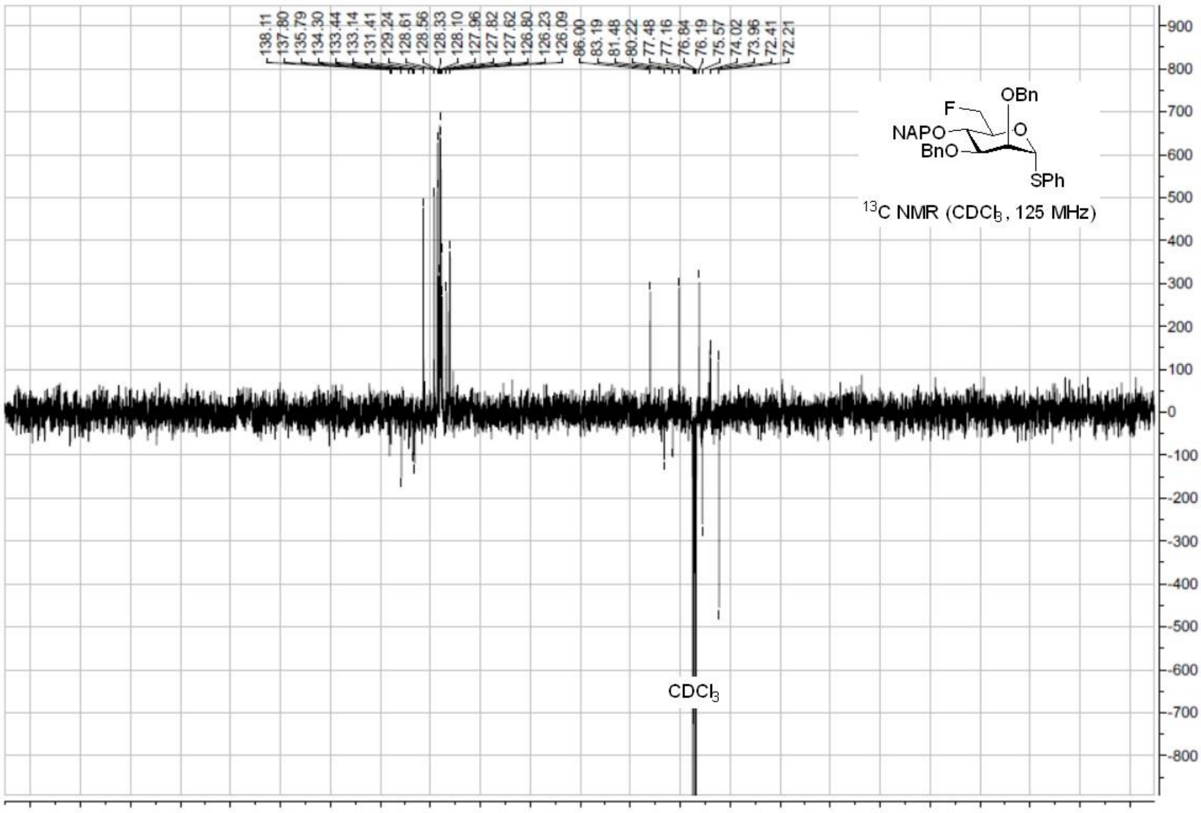

^{1}H and ^{13}C NMR spectra of compound **43**

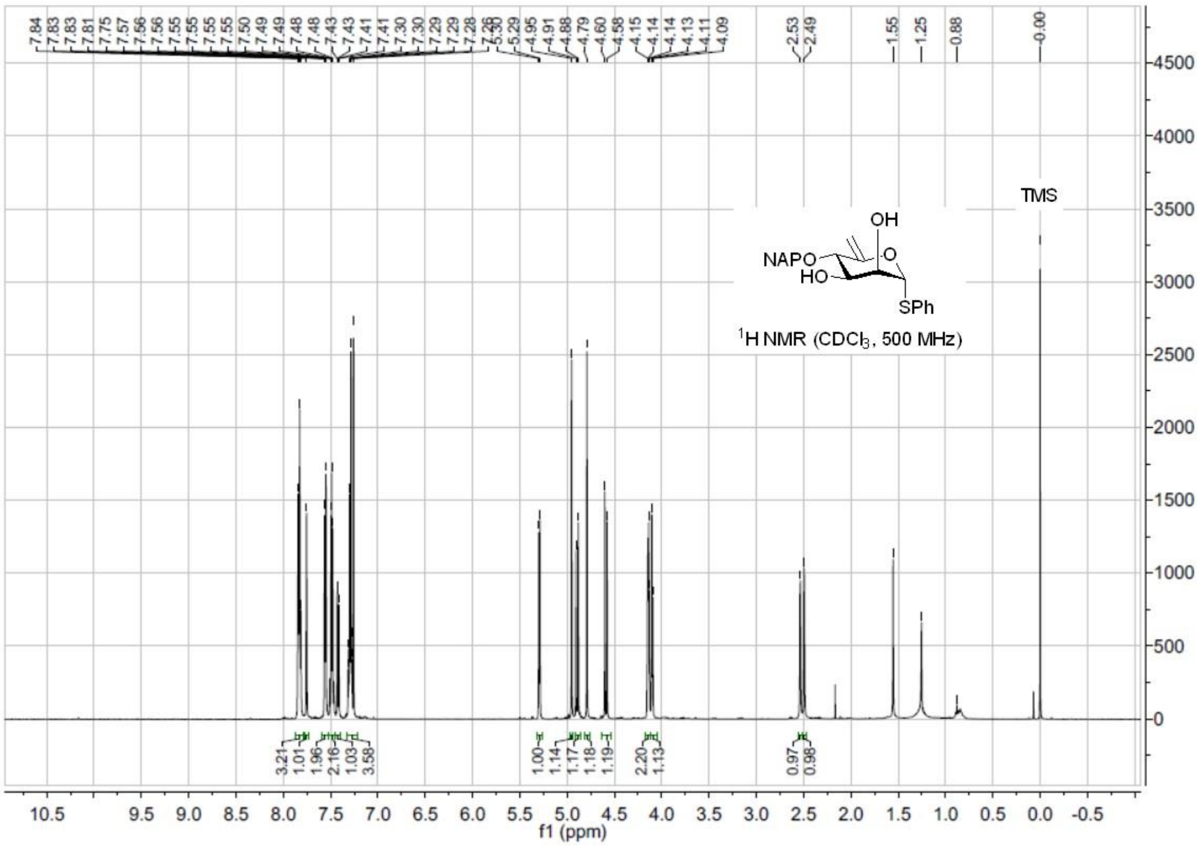

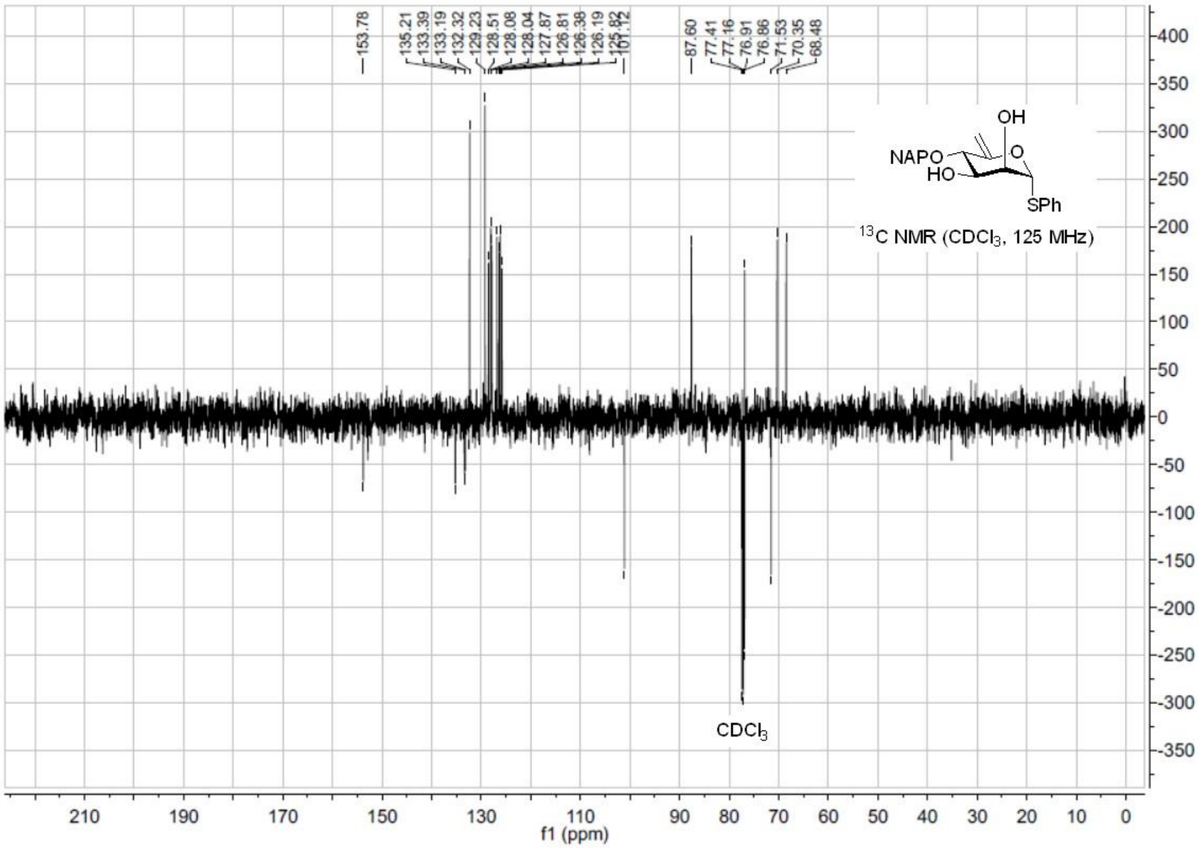

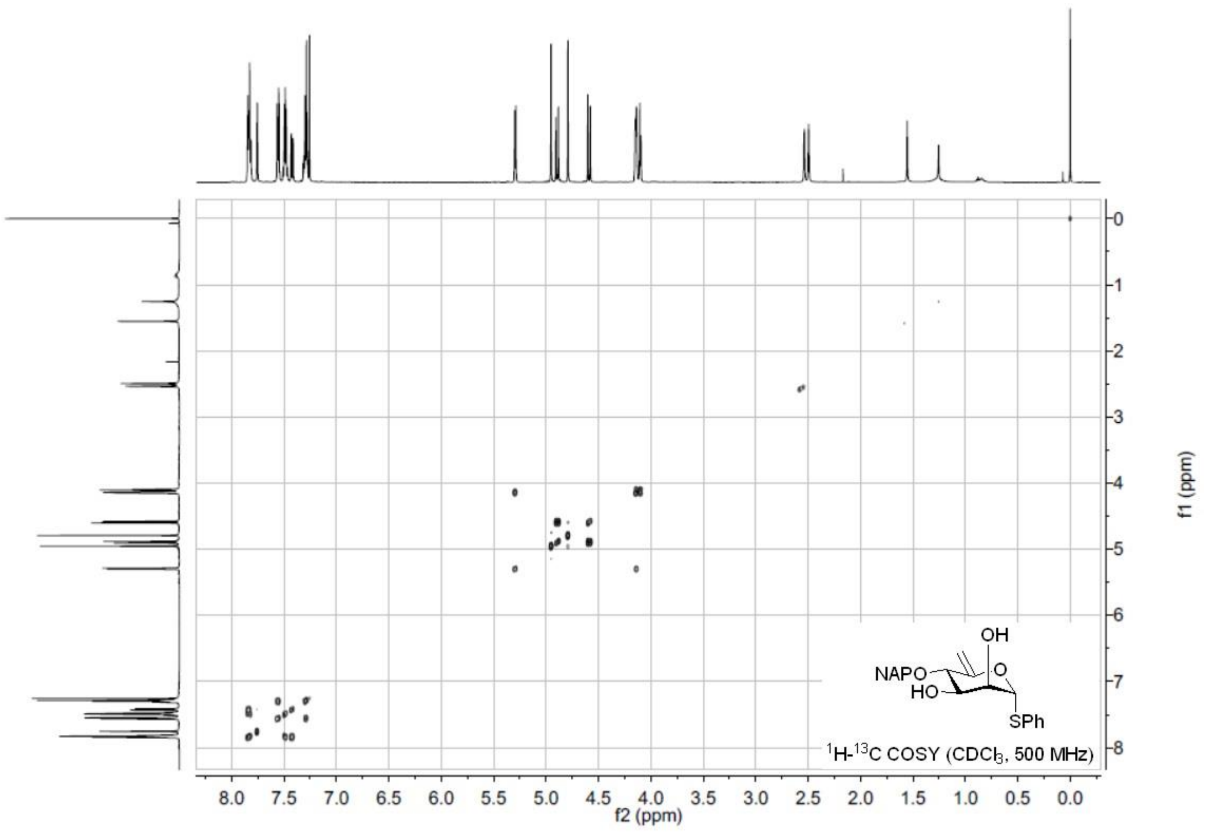
OH
NAPO
O
HO
SPh
^{1}H-^{13}C COSY (CDCl$_3$, 500 MHz)
f1 (ppm)
f2 (ppm)

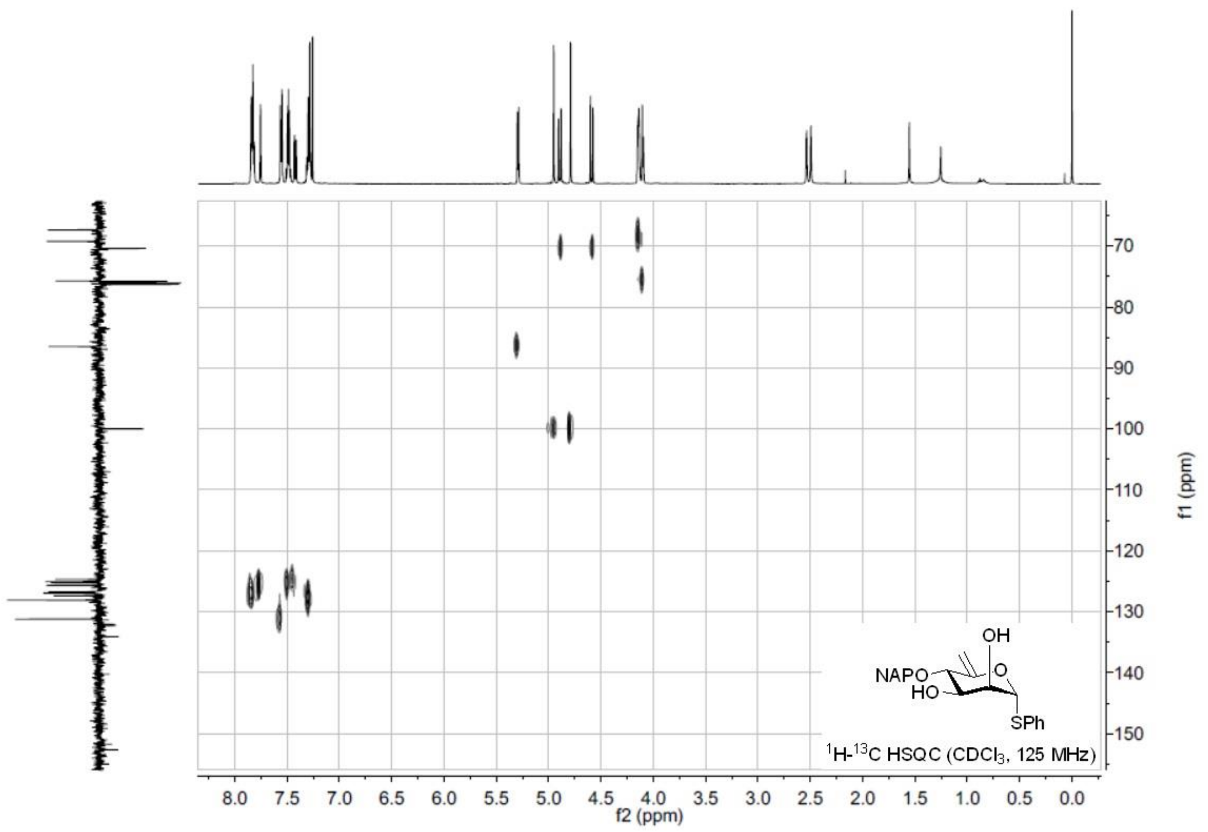
OH
NAPO
O
HO
SPh
^{1}H-^{13}C HSQC (CDCl$_3$, 125 MHz)
f1 (ppm)
f2 (ppm)

^{1}H and ^{13}C NMR spectra of compound **44**

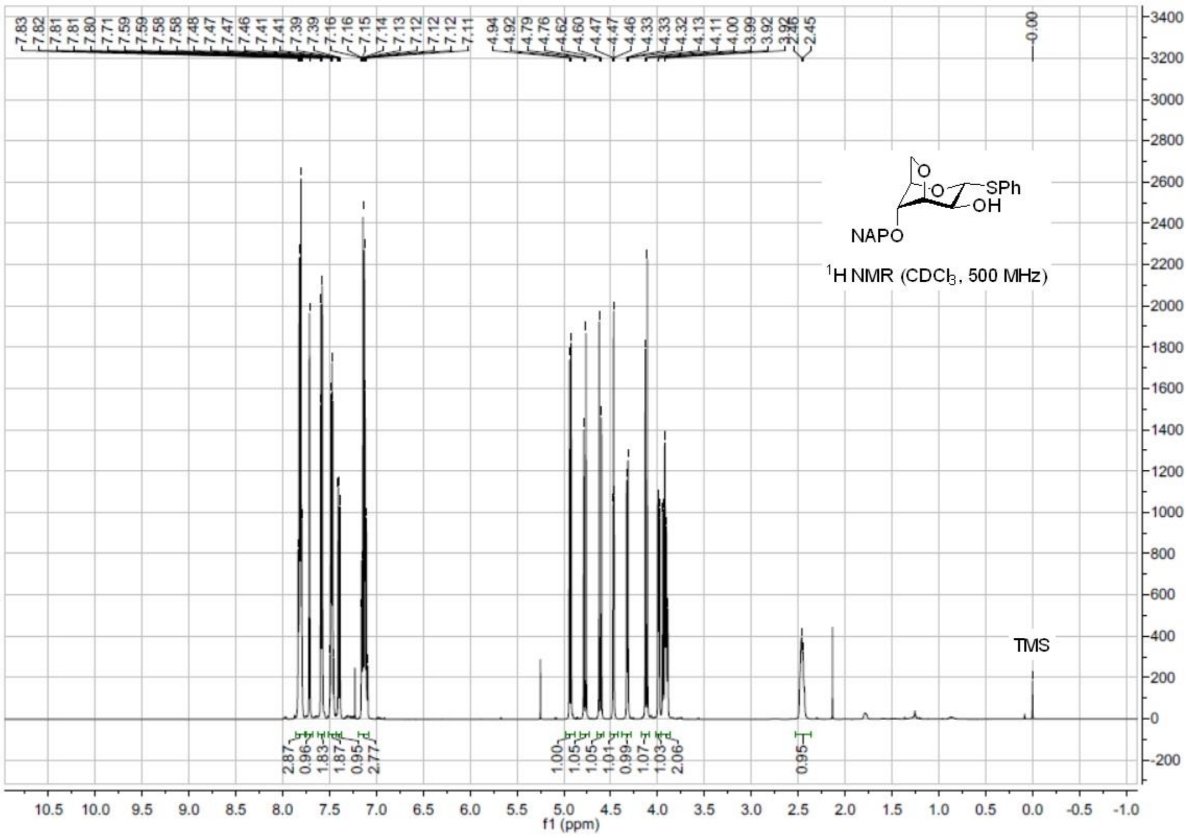

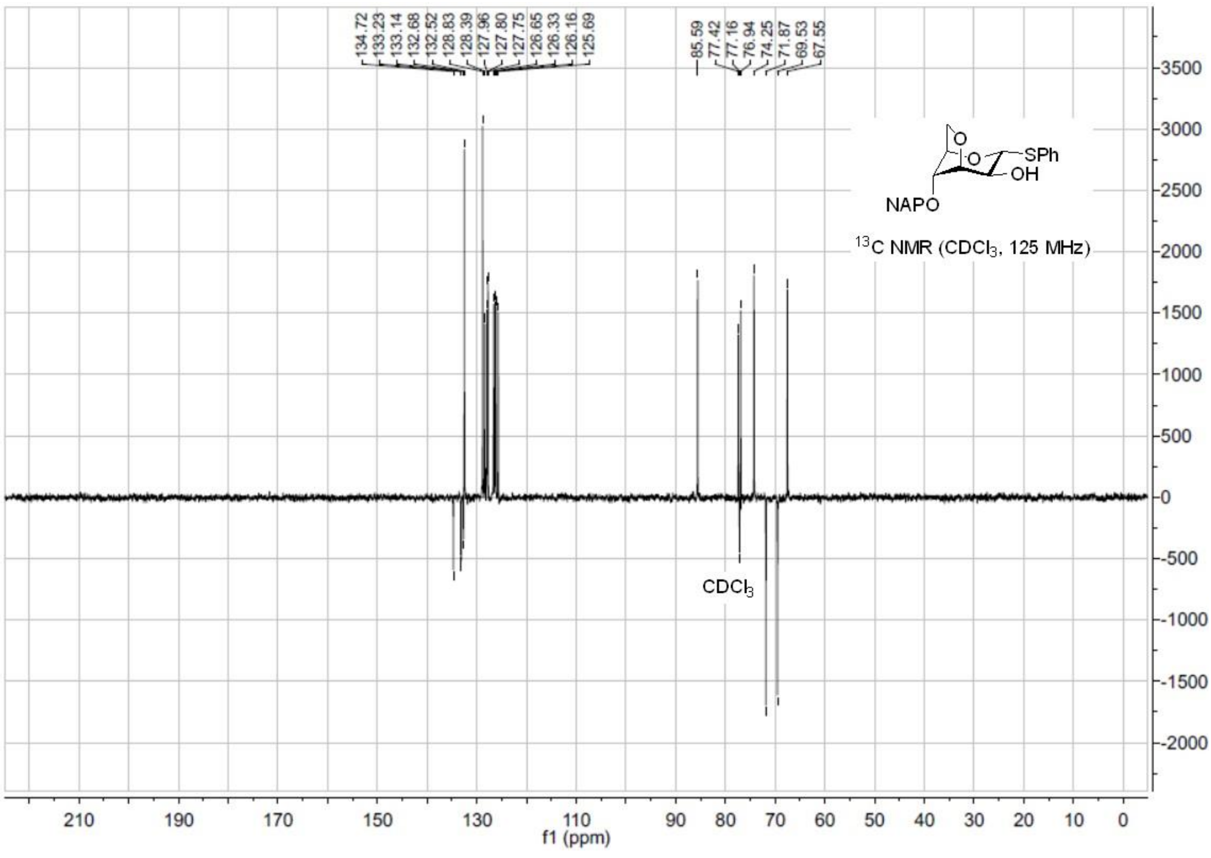

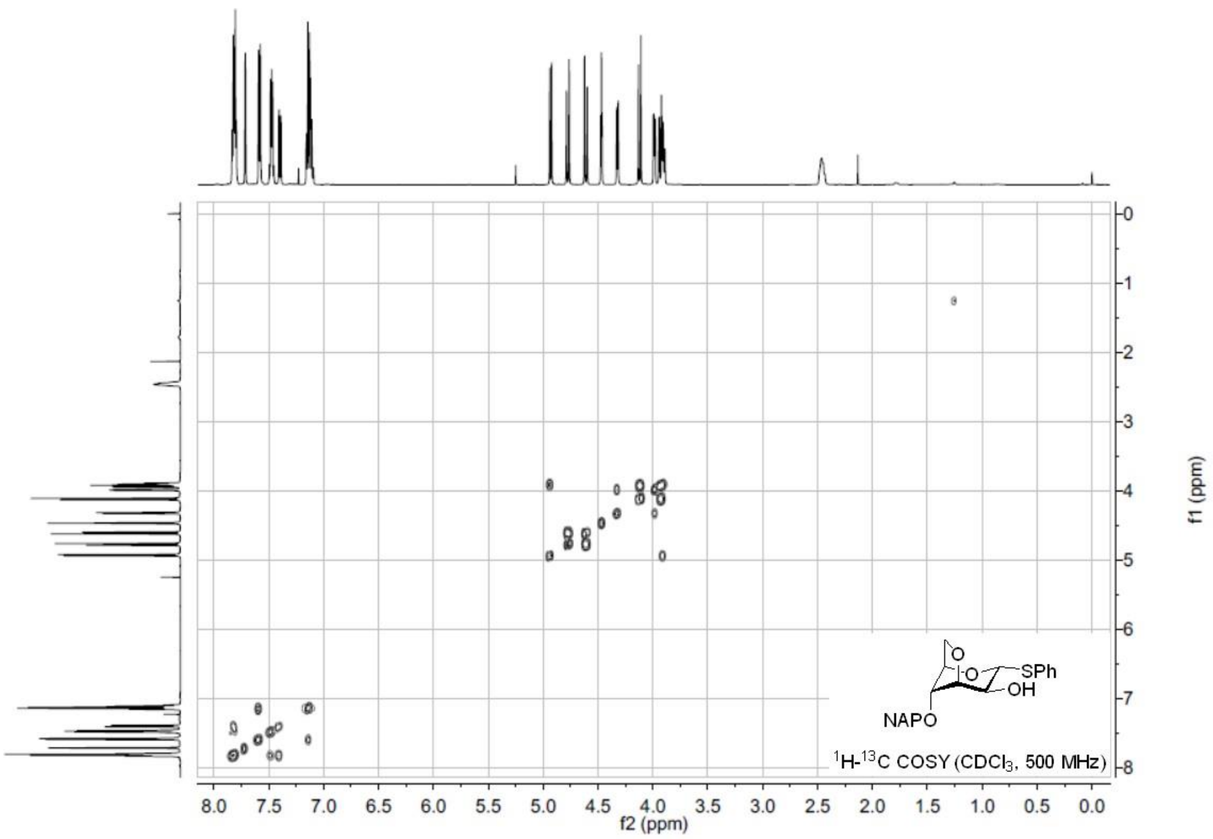
SPh
OH
NAPO
^{1}H-^{13}C COSY (CDCl3, 500 MHz)
f2 (ppm)
f1 (ppm)

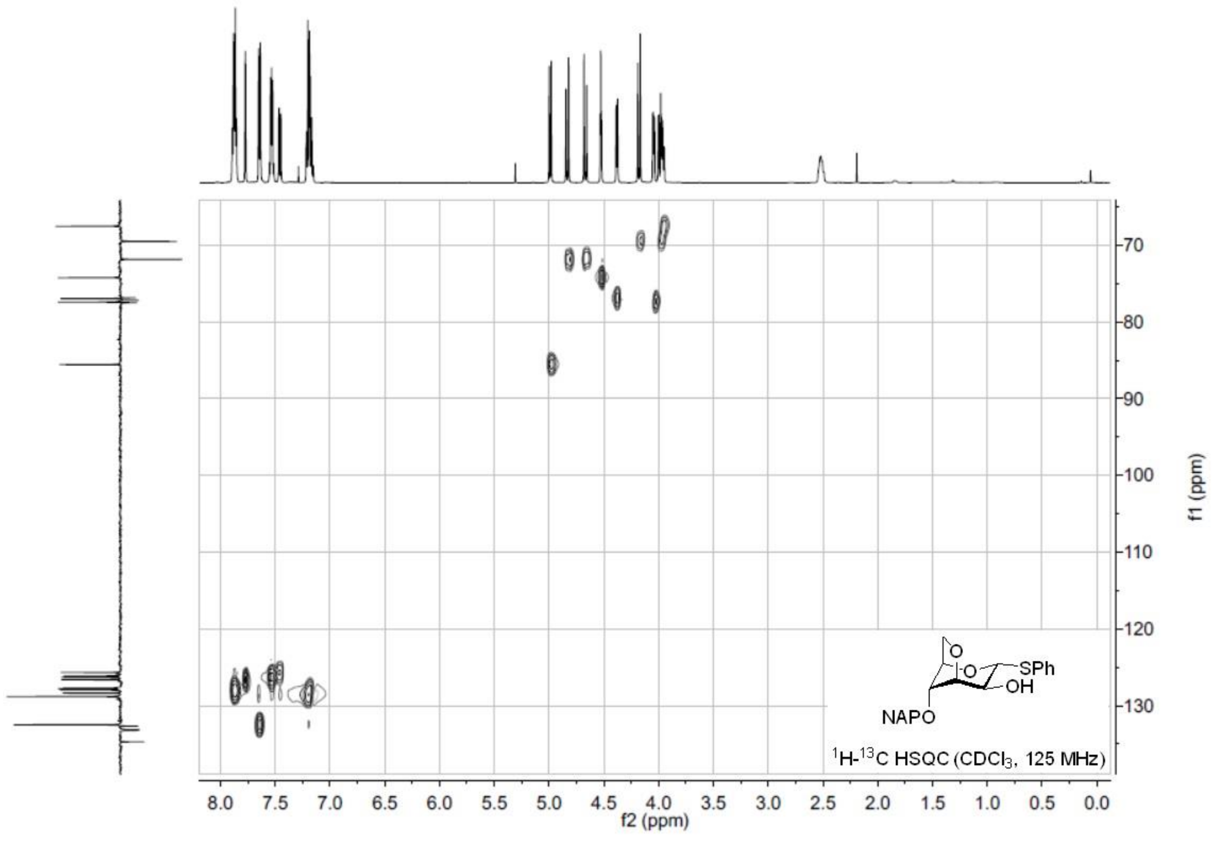
SPh
OH
NAPO
^{1}H-^{13}C HSQC (CDCl3, 125 MHz)
f2 (ppm)
f1 (ppm)

^{1}H and ^{13}C NMR spectra of compound **45**

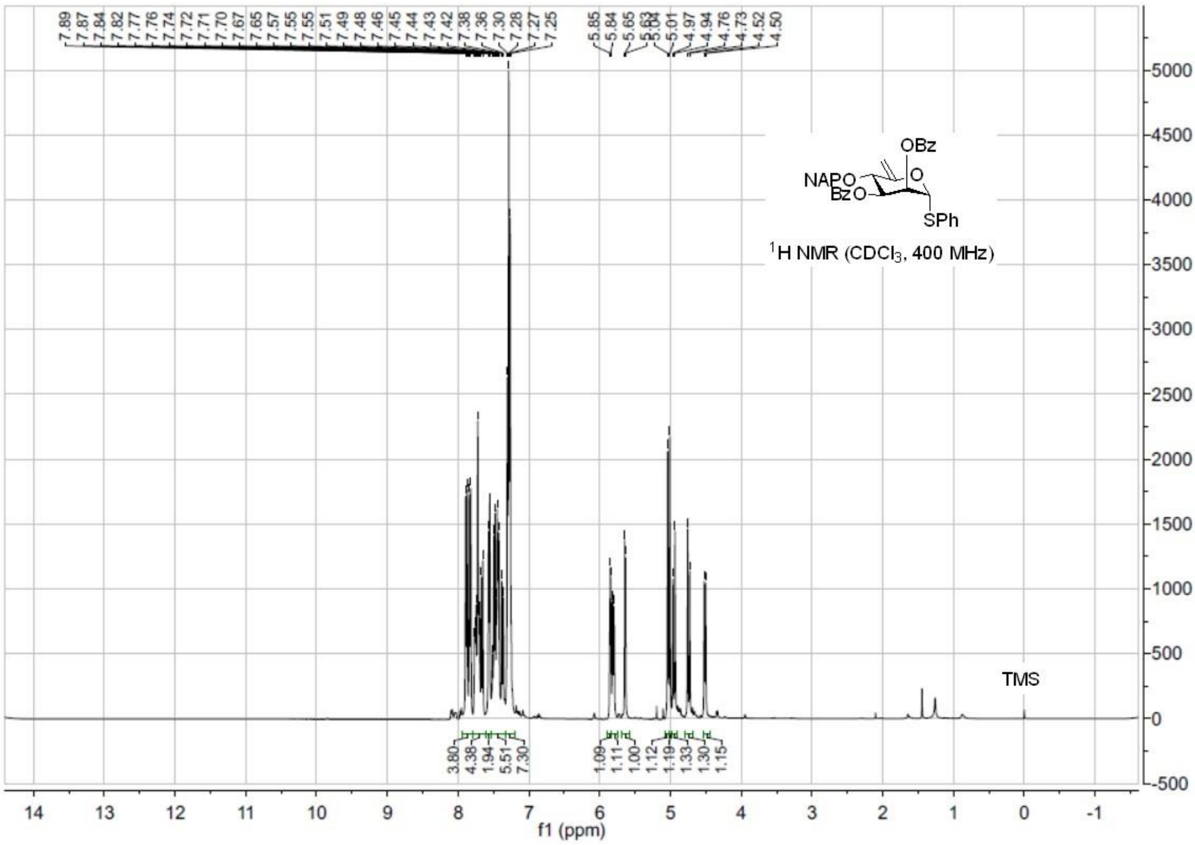

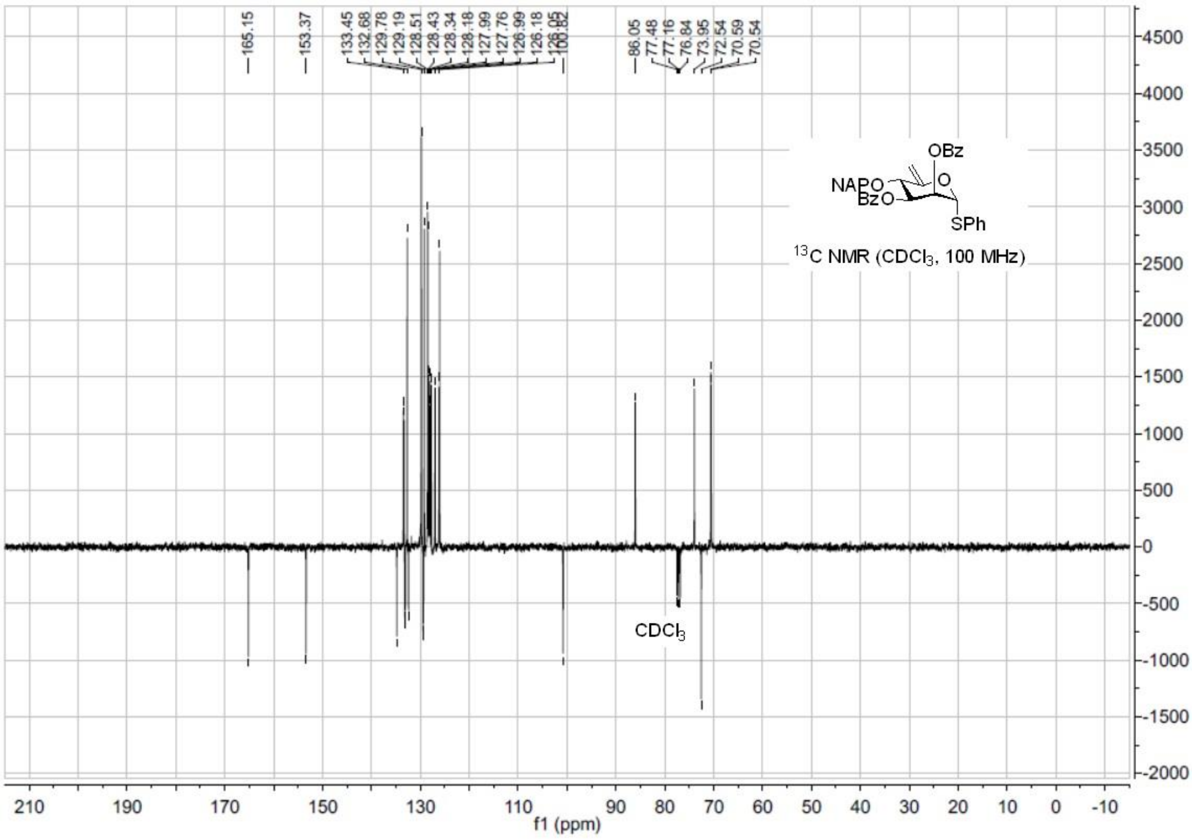

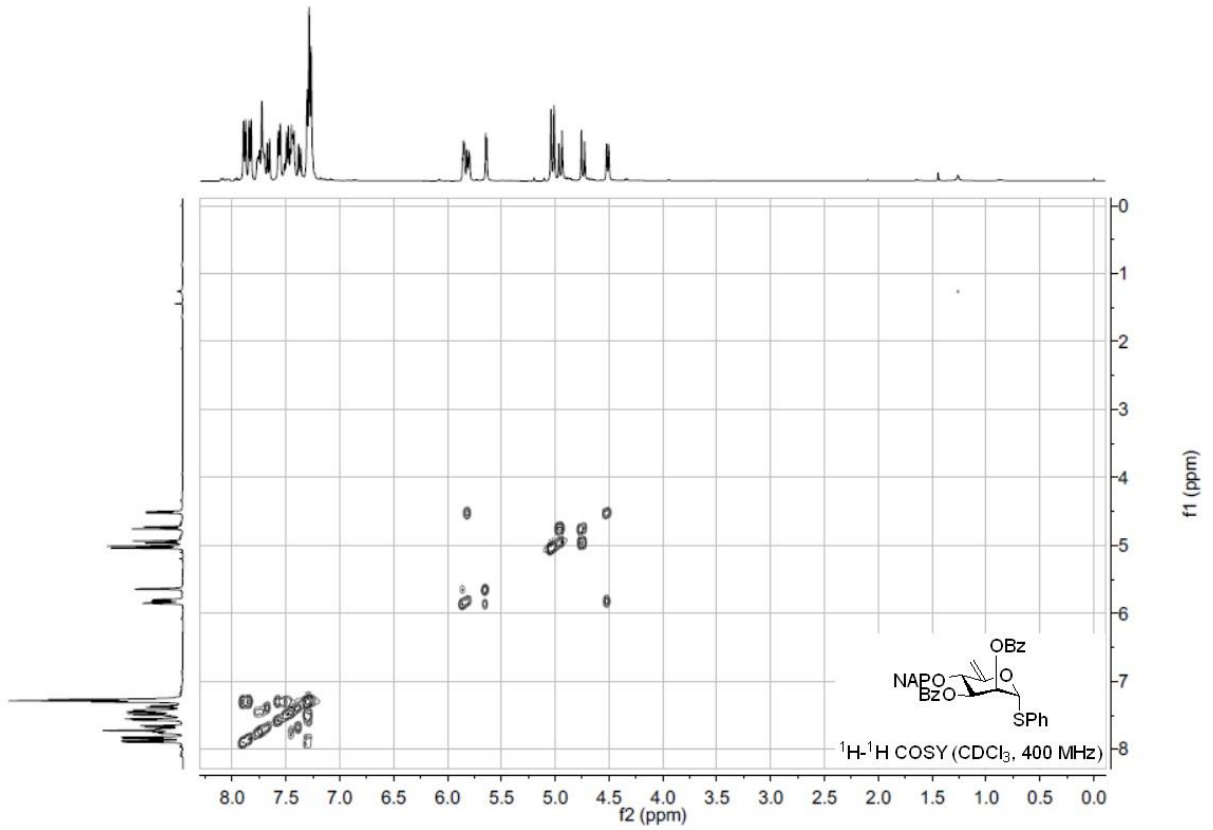
f1 (ppm)
f2 (ppm)
OBz
NAPO
BzO
O
SPh
1H-1H COSY (CDCl3, 400 MHz)

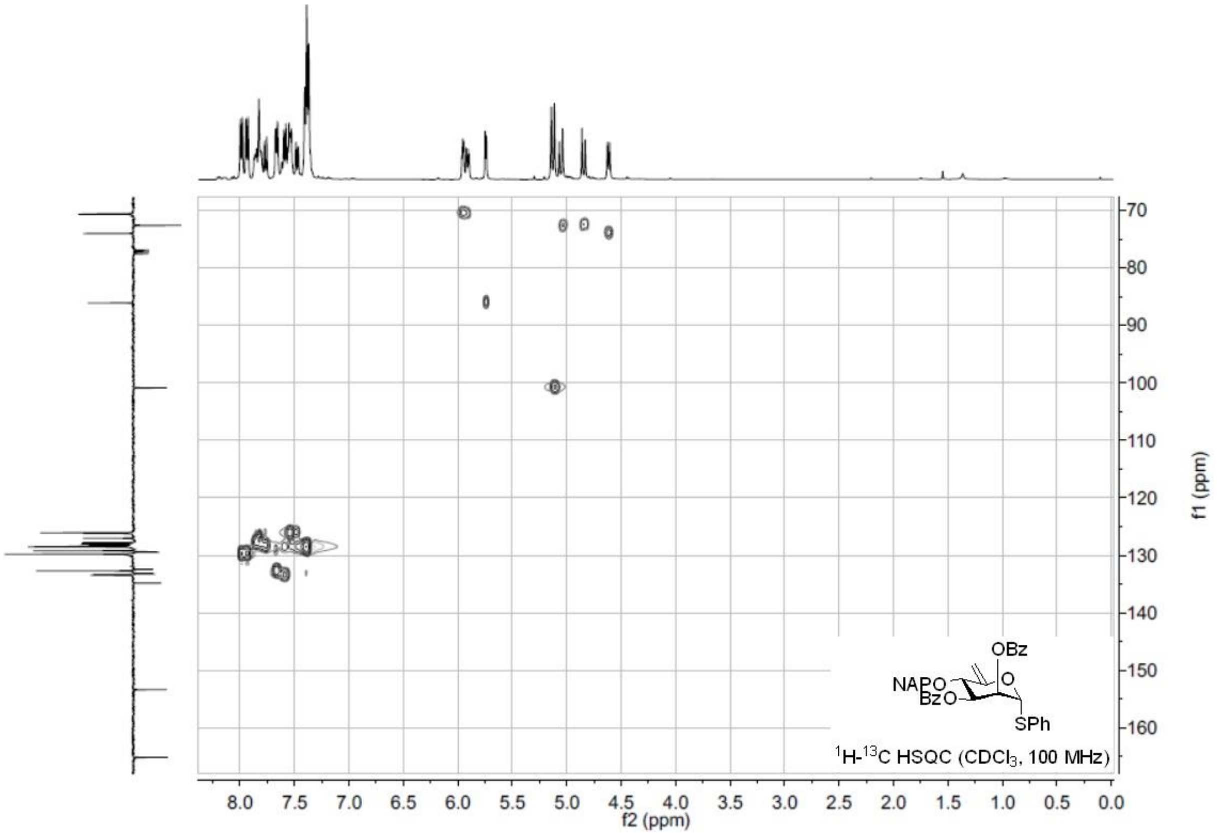
f1 (ppm)
f2 (ppm)
OBz
NAPO
BzO
O
SPh
1H-13C HSQC (CDCl3, 100 MHz)

^{1}H and ^{13}C NMR spectra of compound **46**

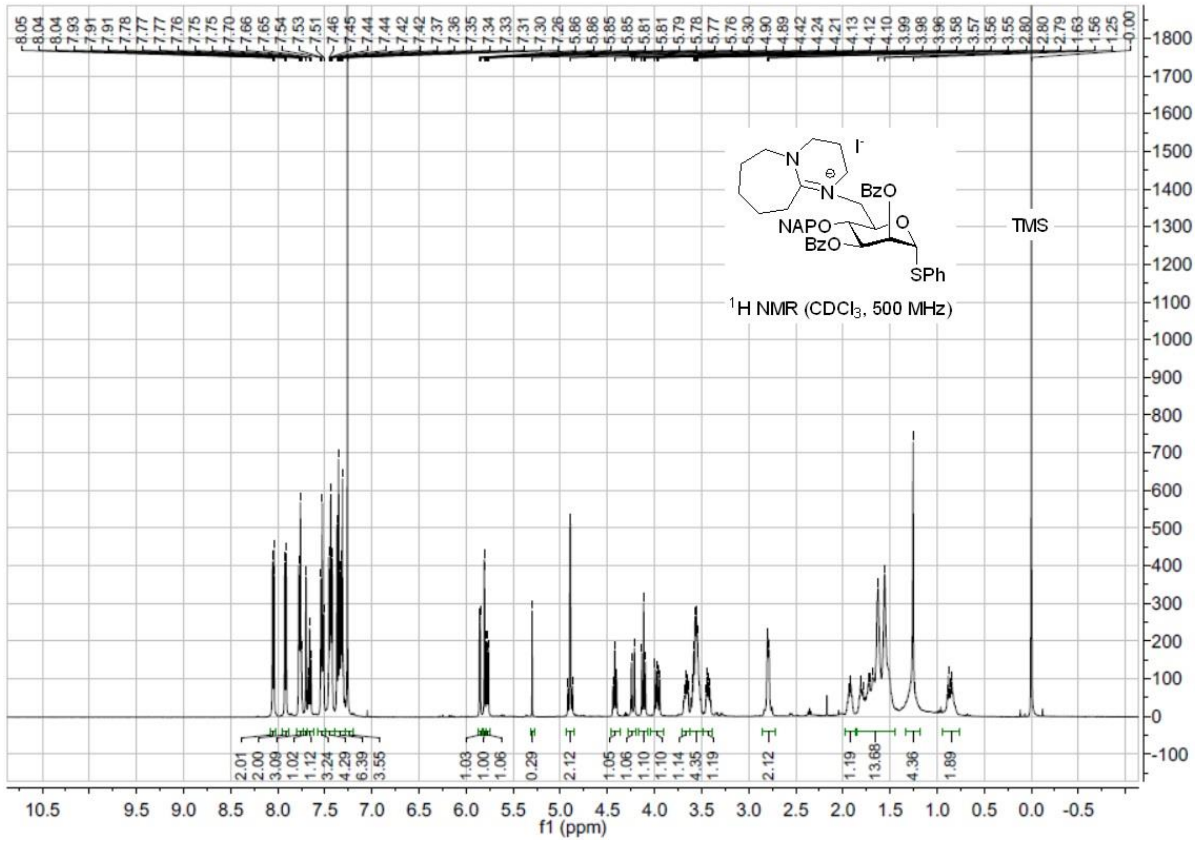

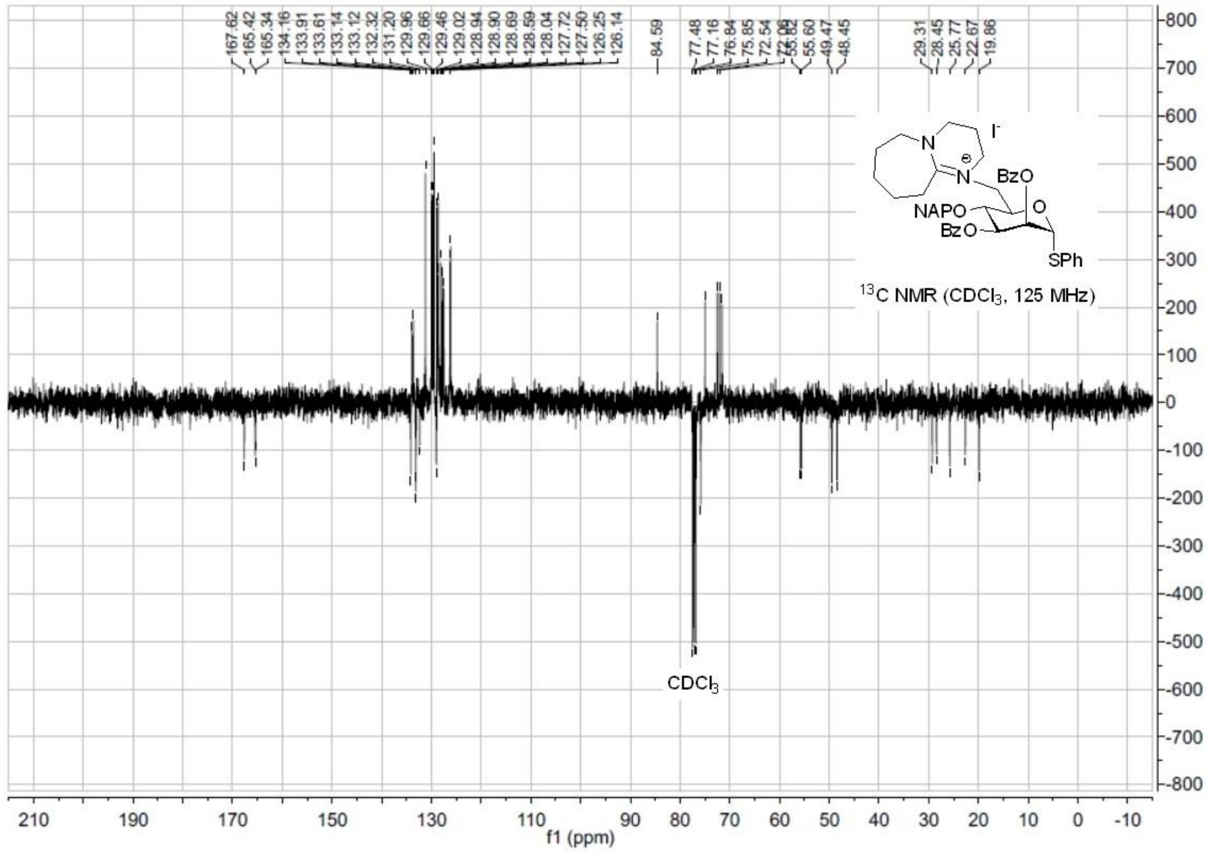

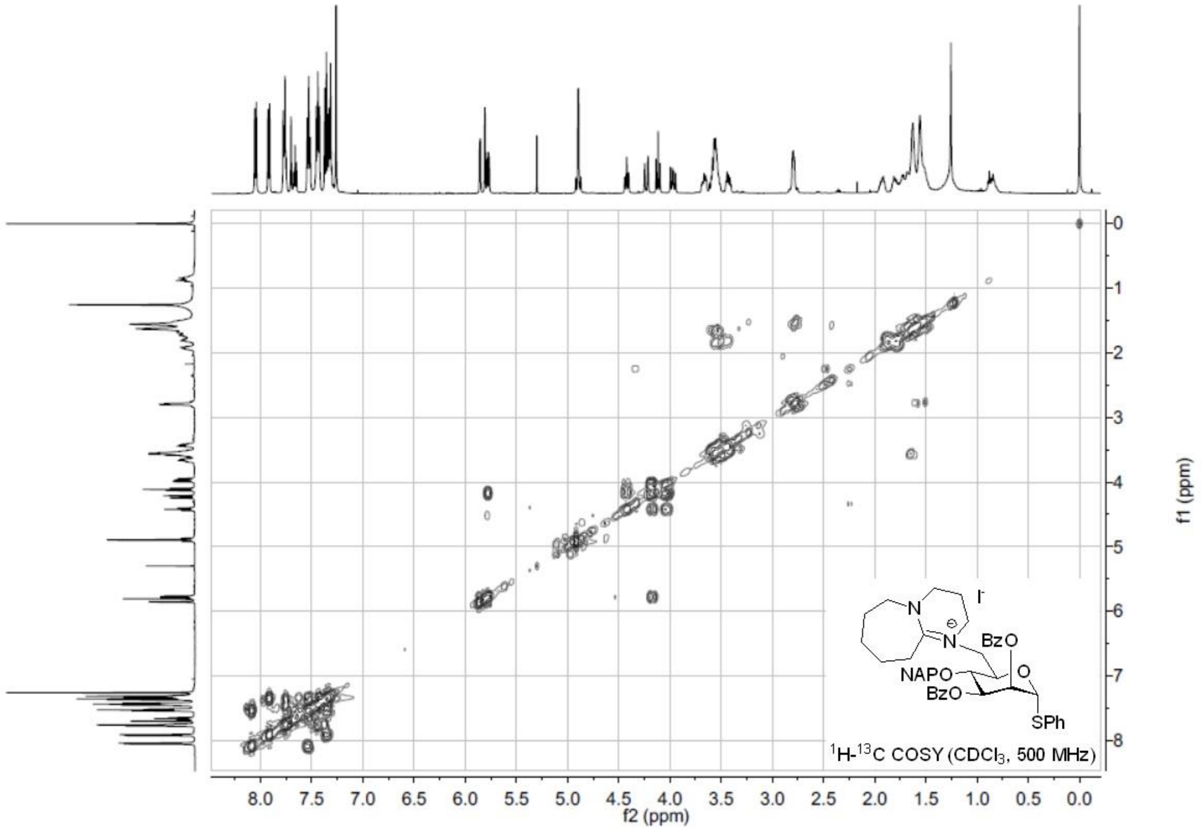

I⁻
N
N
BzO
NAPO
BzO
O
SPh
^{1}H-^{13}C COSY (CDCl$_3$, 500 MHz)
f1 (ppm)
f2 (ppm)

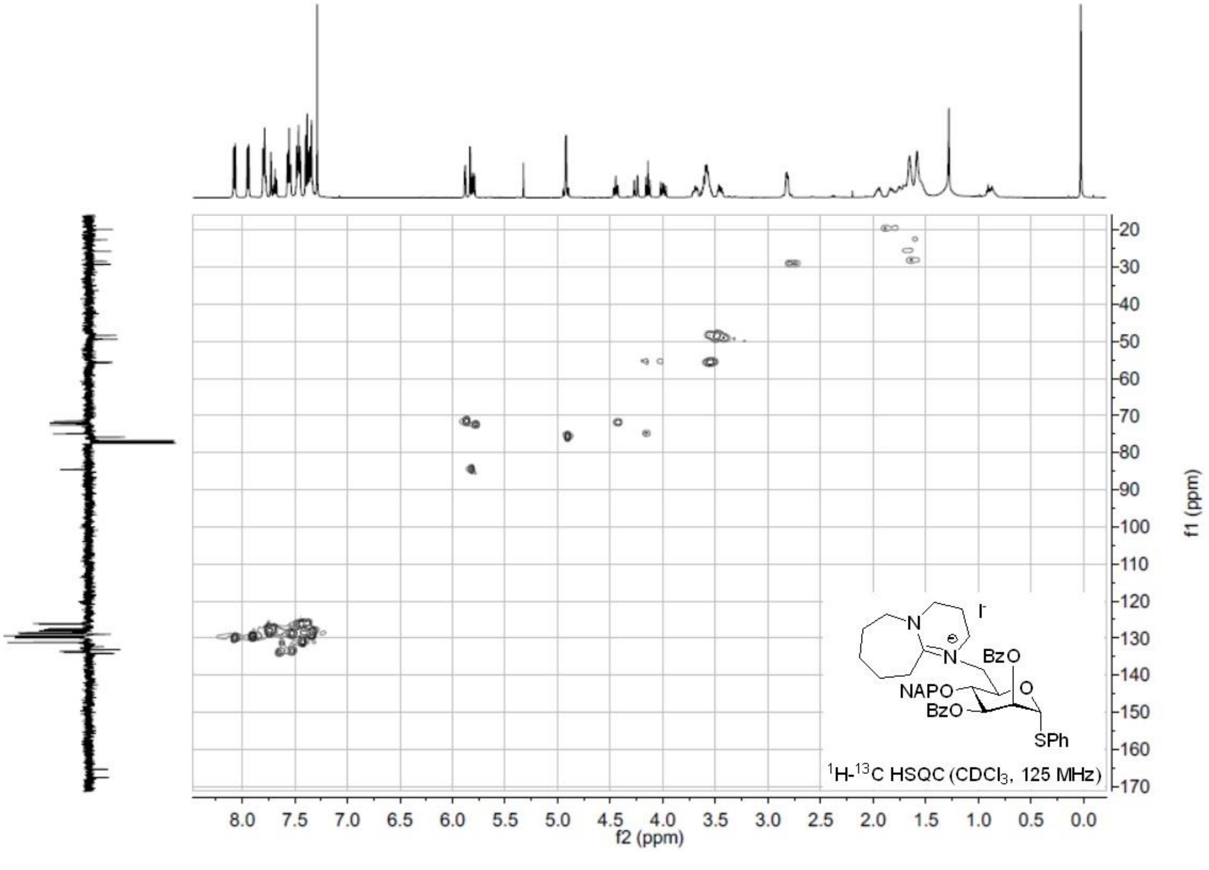

I⁻
N
N
BzO
NAPO
BzO
O
SPh
^{1}H-^{13}C HSQC (CDCl$_3$, 125 MHz)
f1 (ppm)
f2 (ppm)

^{1}H and ^{13}C NMR spectra of compound **47**

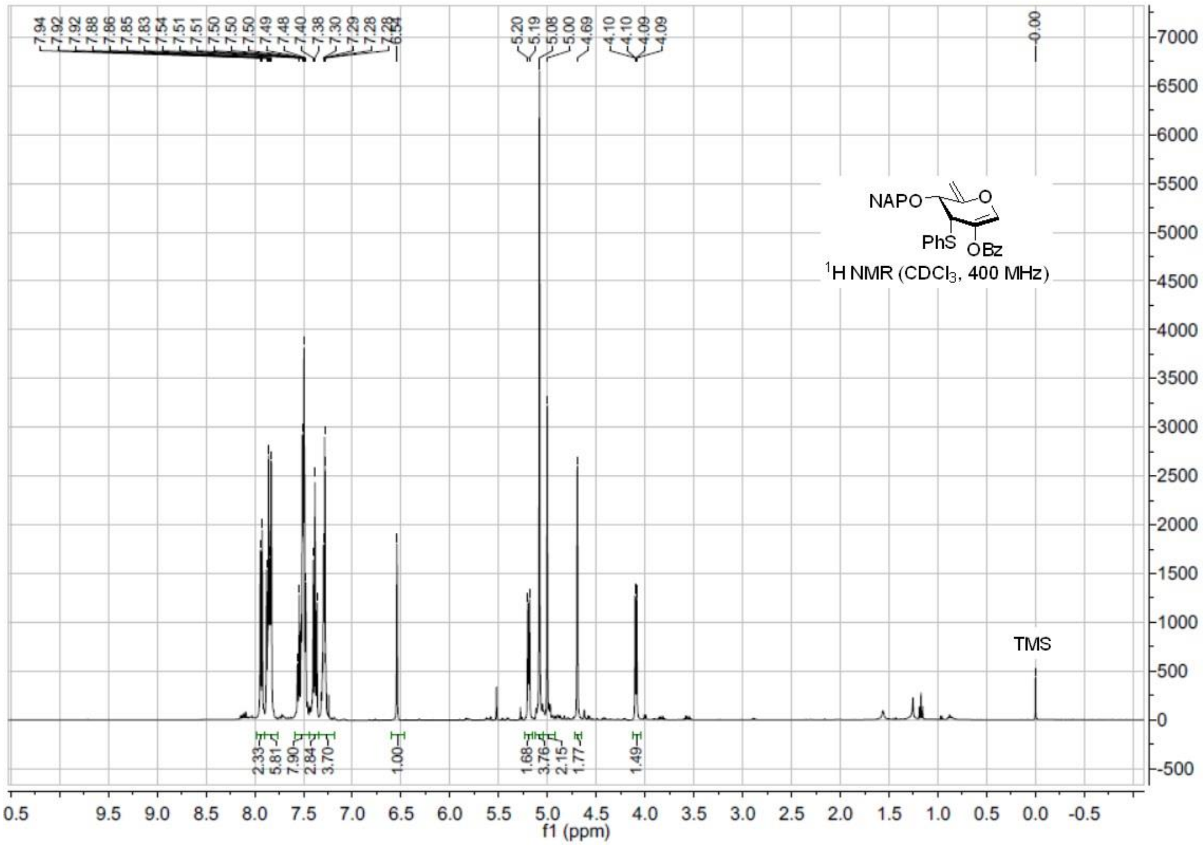

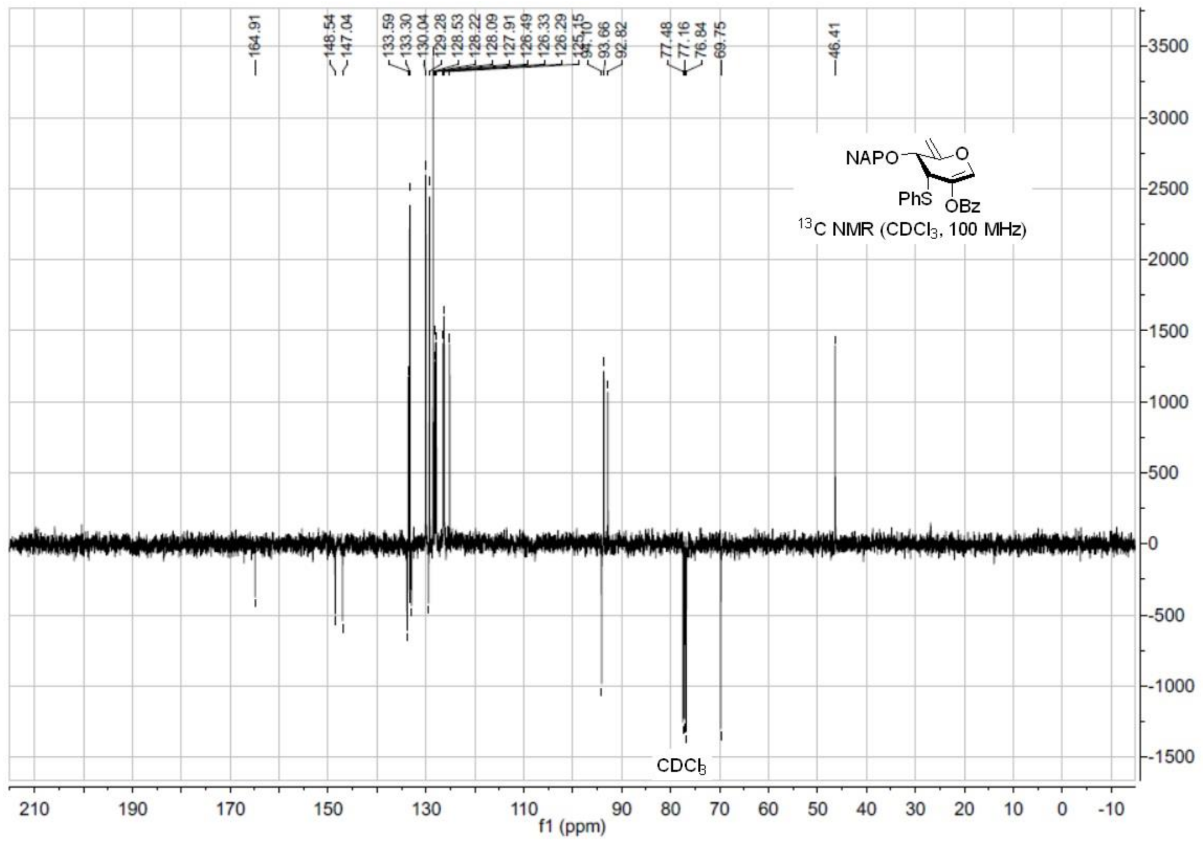

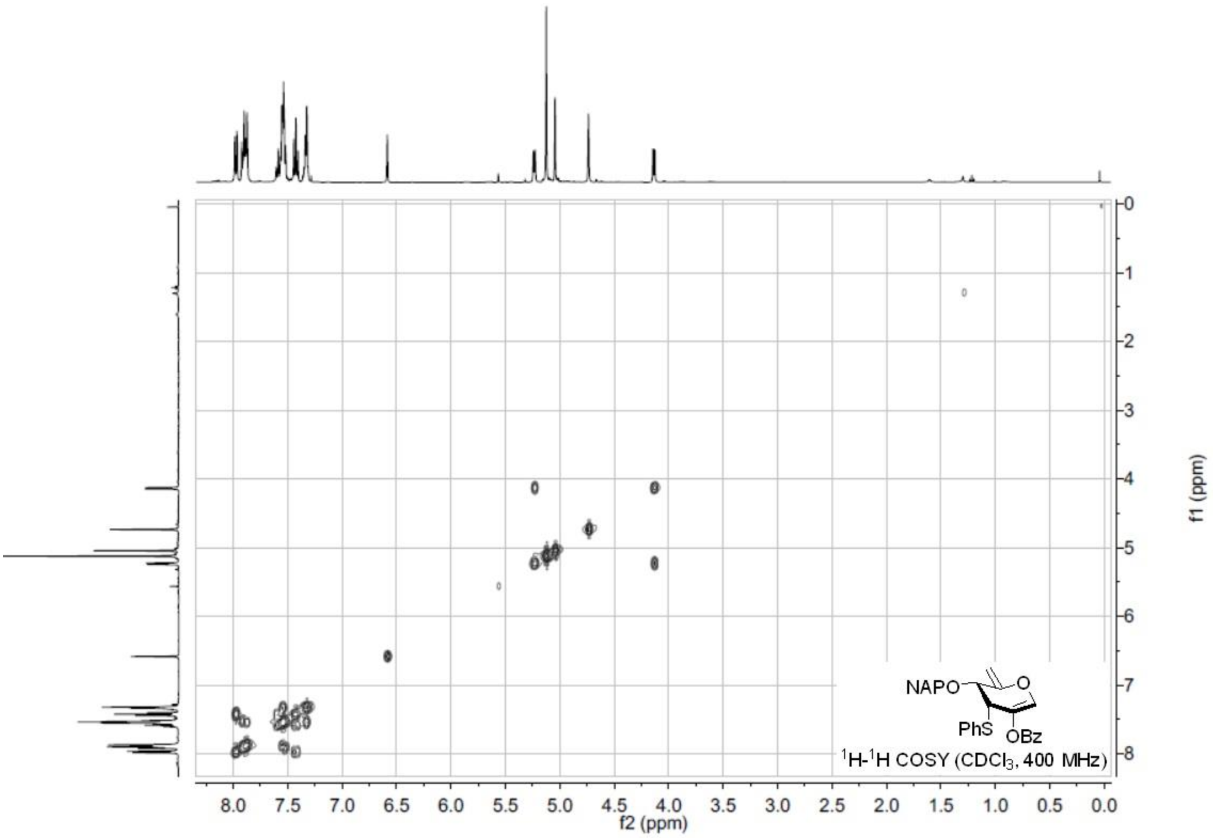
NAPO
O
PhS
OBz
^{1}H-^{1}H COSY (CDCl$_3$, 400 MHz)
f2 (ppm)
f1 (ppm)

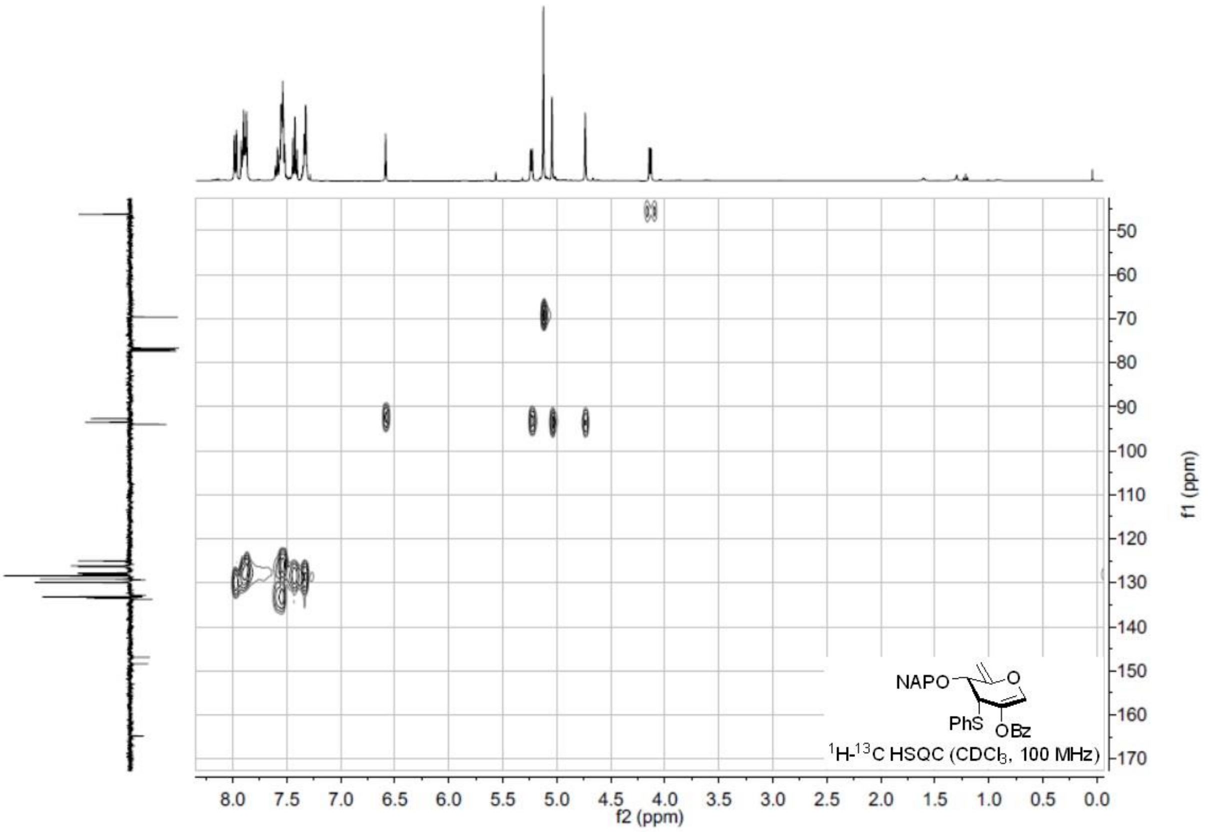
NAPO
O
PhS
OBz
^{1}H-^{13}C HSQC (CDCl$_3$, 100 MHz)
f2 (ppm)
f1 (ppm)

^{1}H and ^{13}C NMR spectra of compound **48**

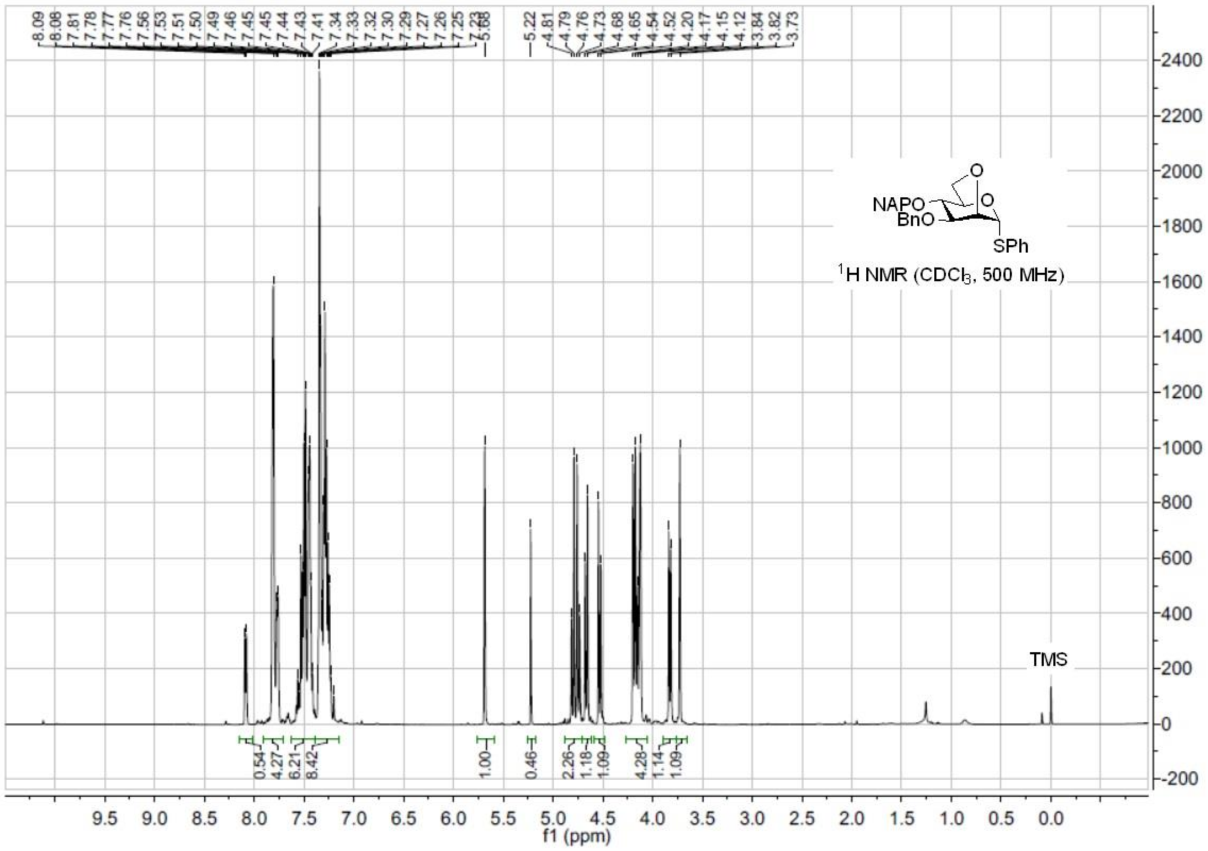

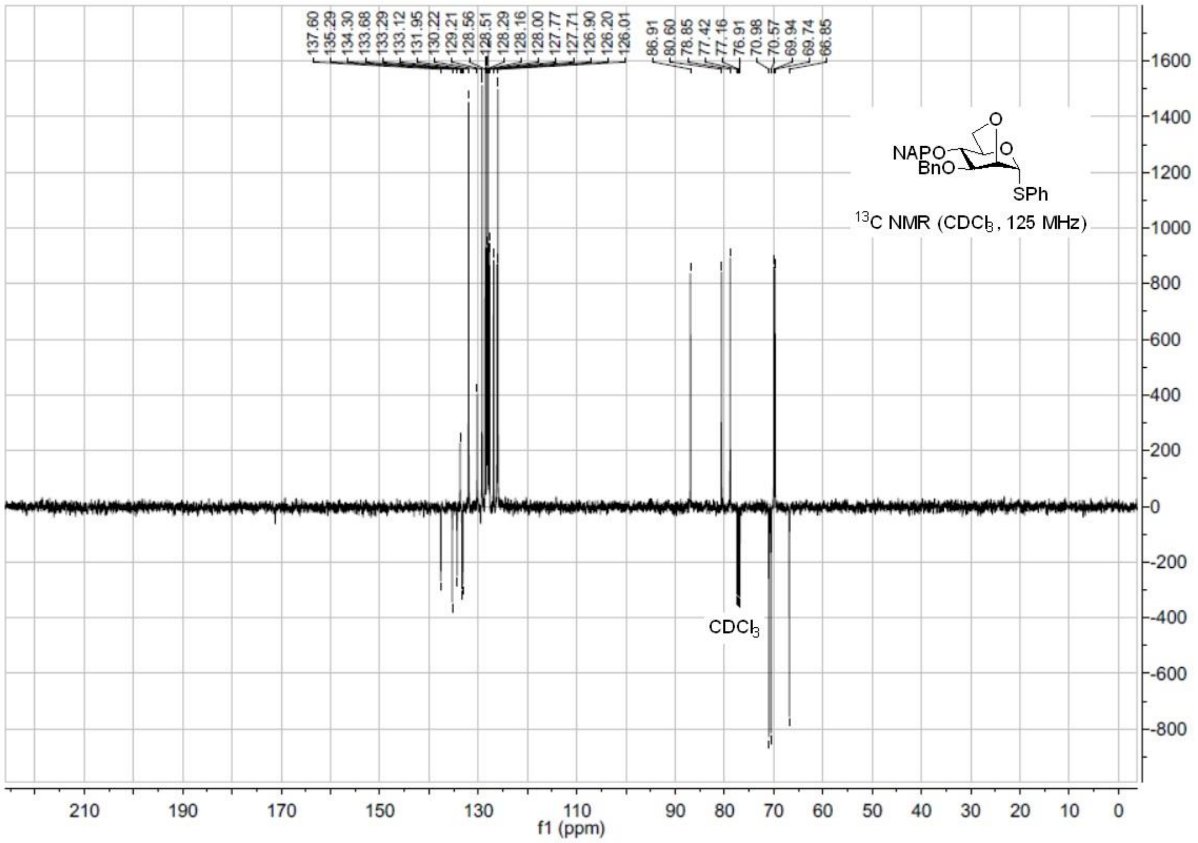

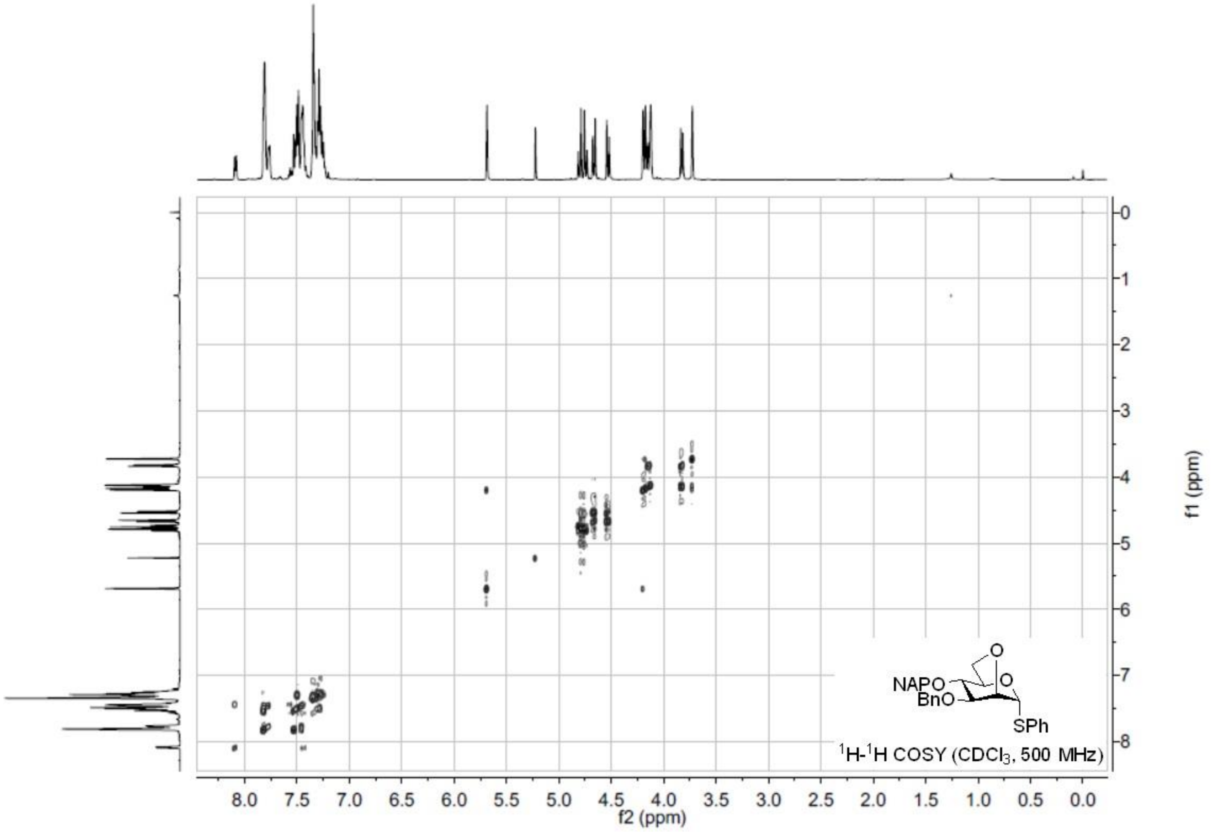
NAPO
BnO
O
O
SPh
1H-1H COSY (CDCl3, 500 MHz)
f2 (ppm)
f1 (ppm)

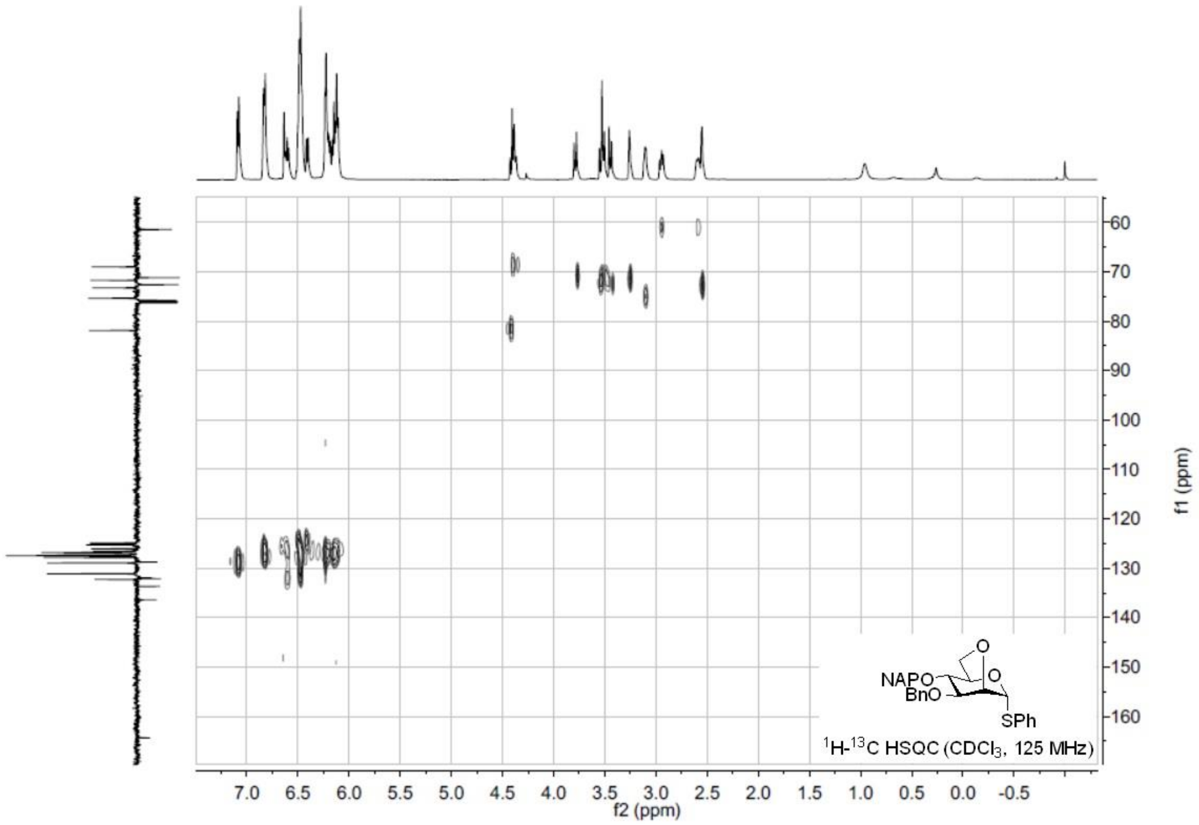
NAPO
BnO
O
O
SPh
1H-13C HSQC (CDCl3, 125 MHz)
f2 (ppm)
f1 (ppm)

^1H and ^{13}C NMR spectra of compound **49**

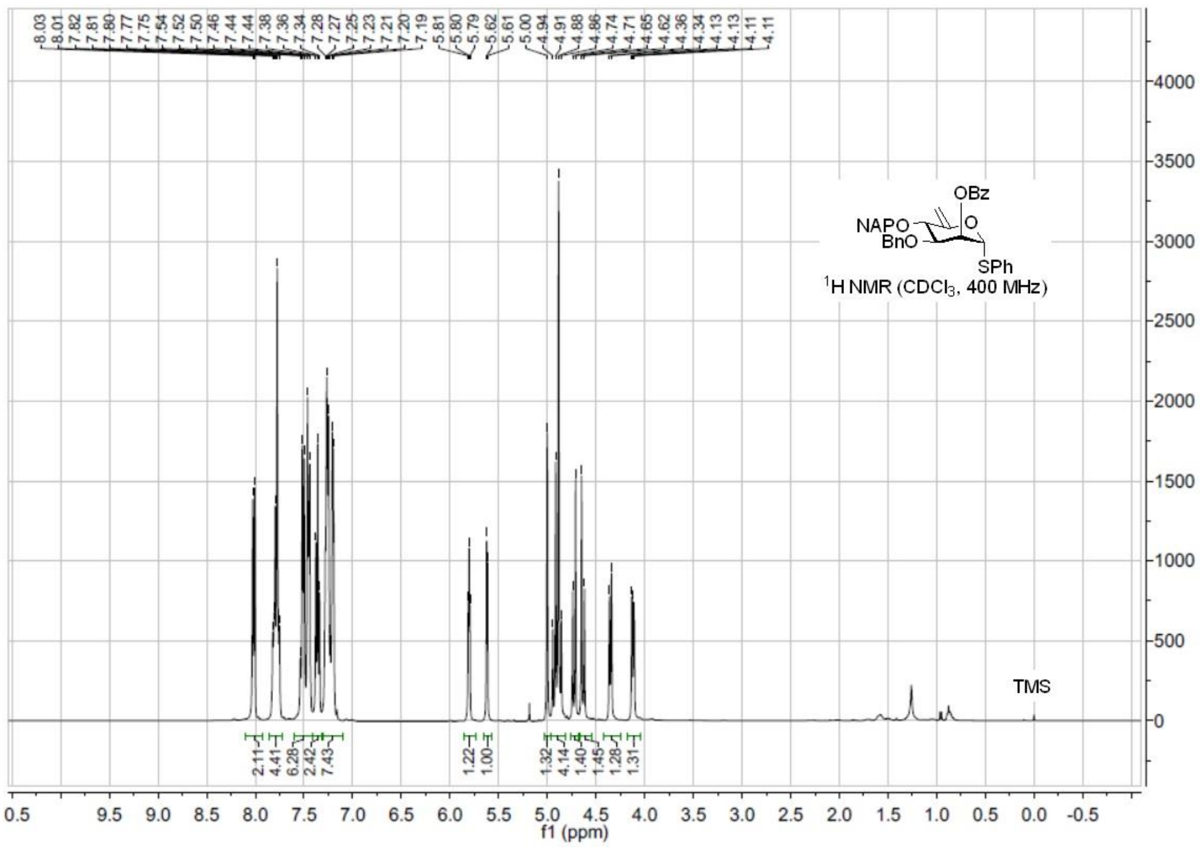

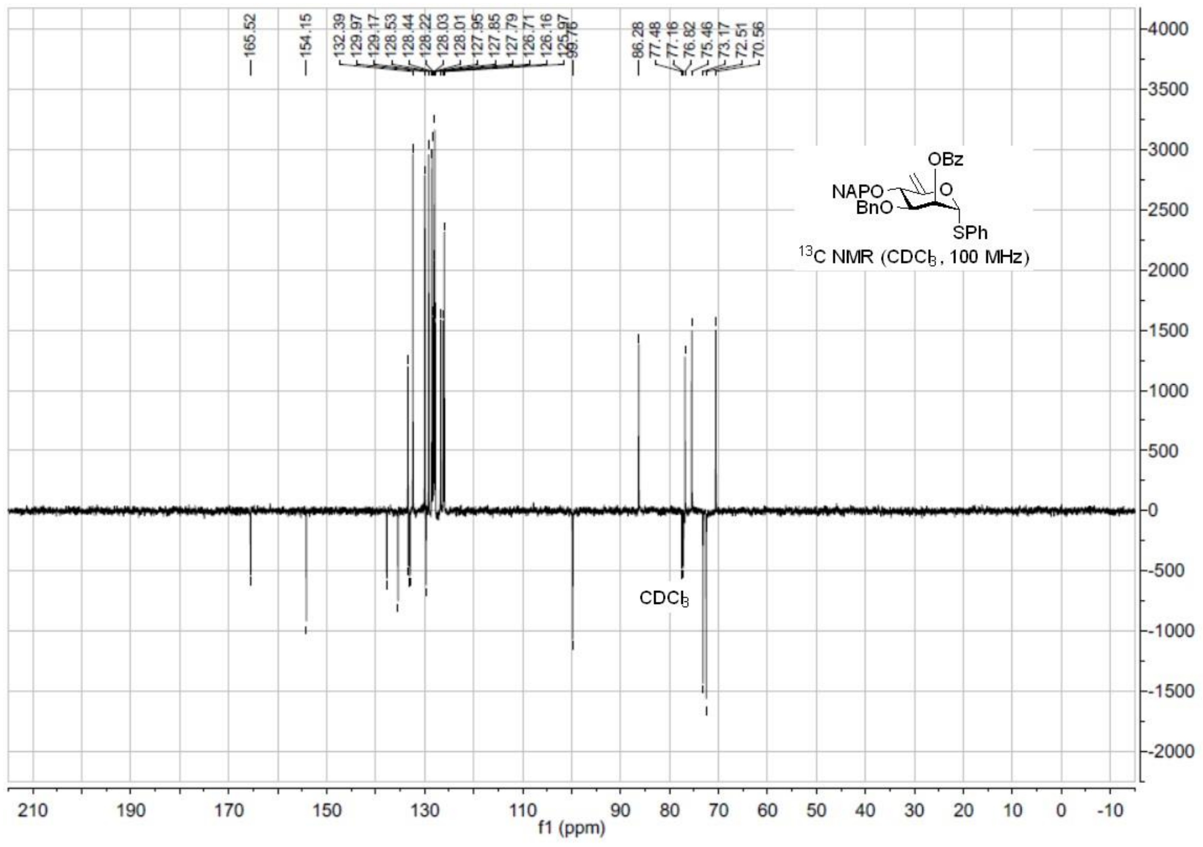

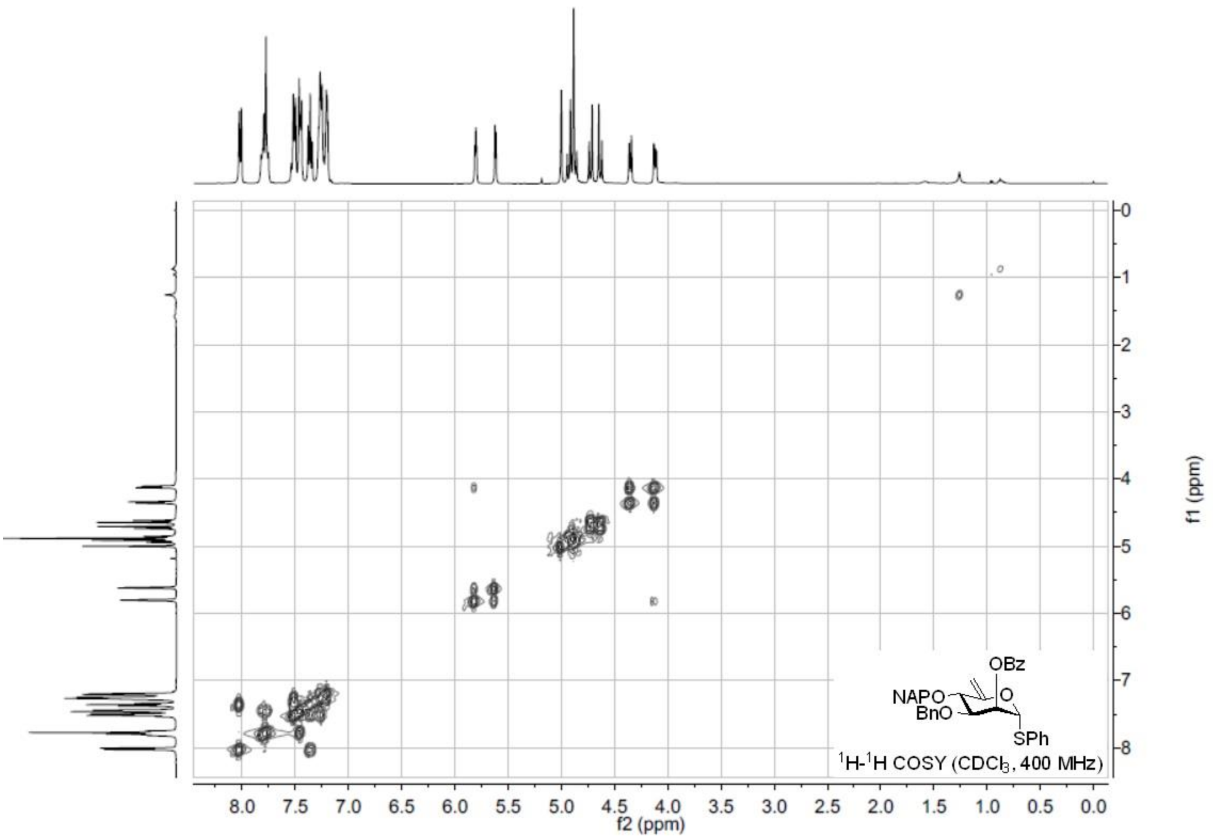
OBz
NAPO
BnO
O
SPh
^{1}H-^{1}H COSY (CDCl$_3$, 400 MHz)
f1 (ppm)
f2 (ppm)

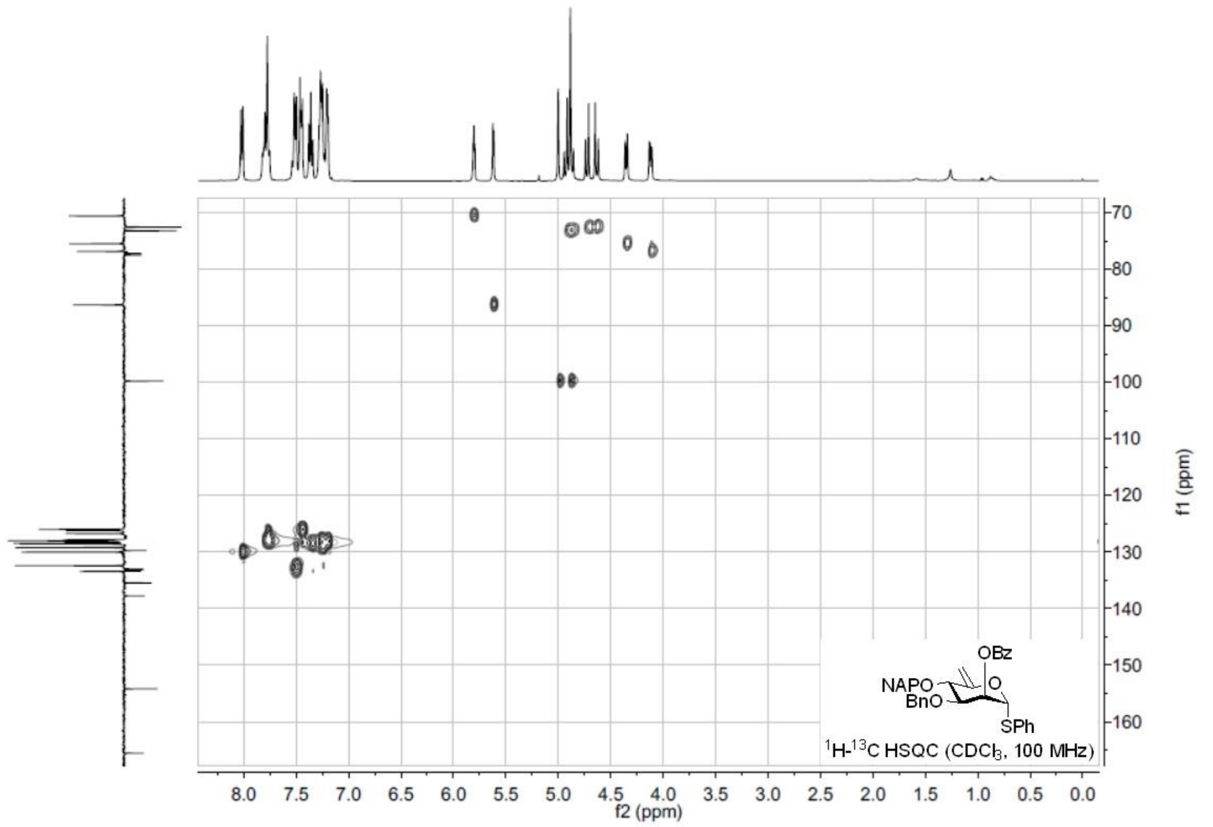
OBz
NAPO
BnO
O
SPh
^{1}H-^{13}C HSQC (CDCl$_3$, 100 MHz)
f1 (ppm)
f2 (ppm)

^{1}H and ^{13}C NMR spectra of compound **50**

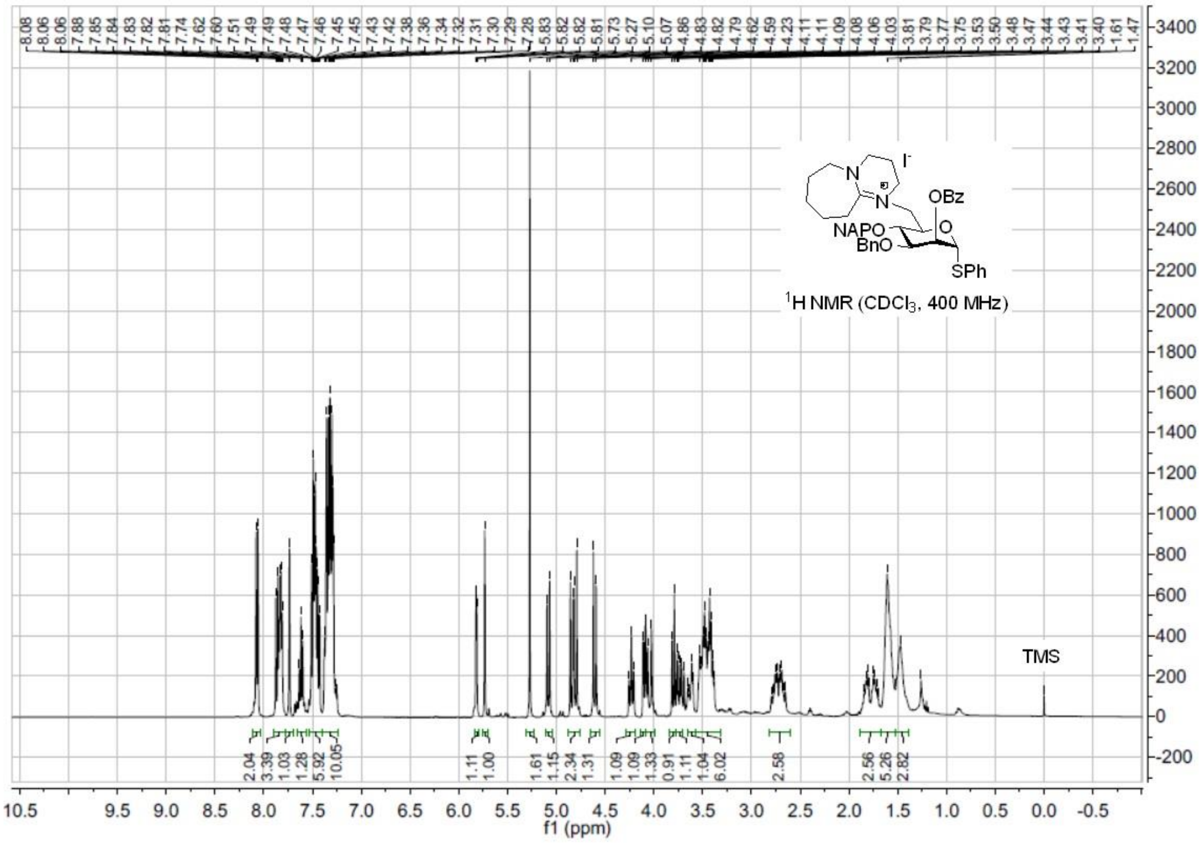

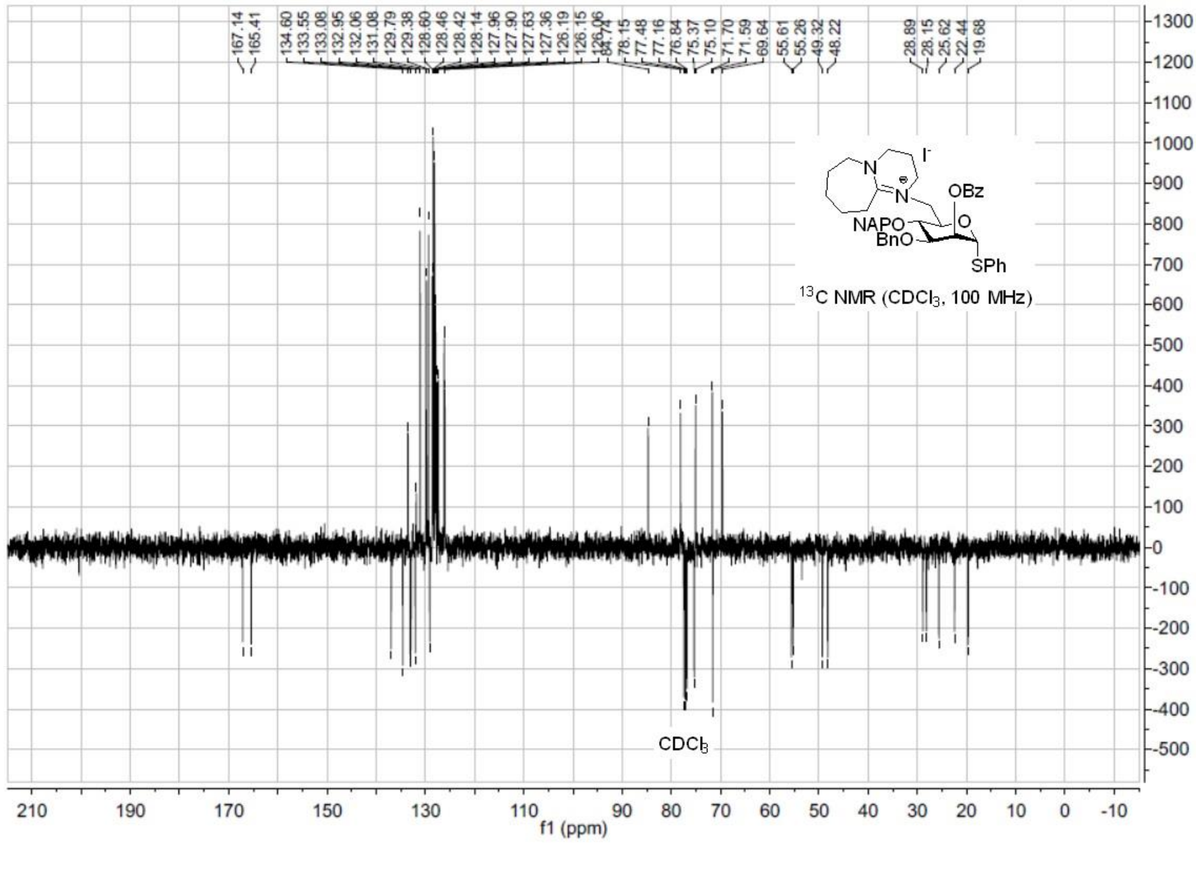

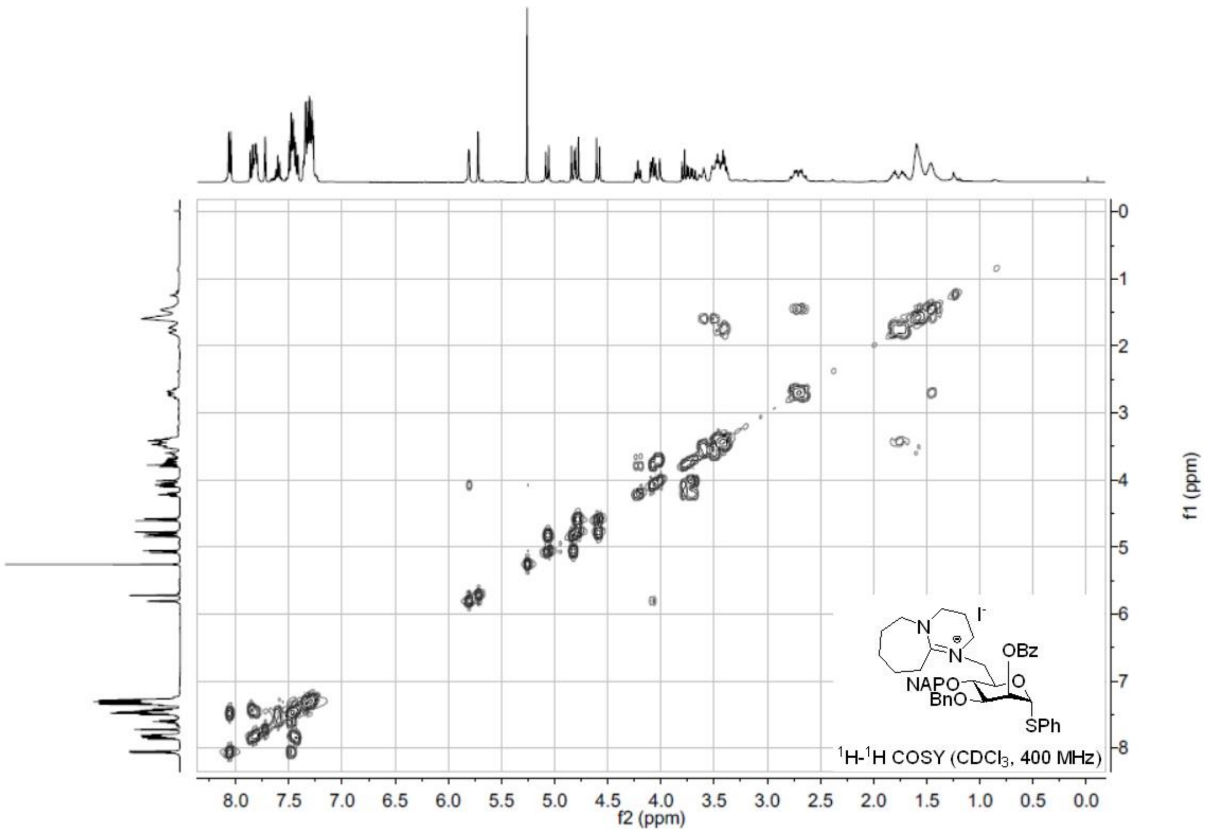

OBz
NAPO
BnO
O
SPh
I⁻
N
^{1}H-^{1}H COSY (CDCl$_3$, 400 MHz)
f2 (ppm)
f1 (ppm)

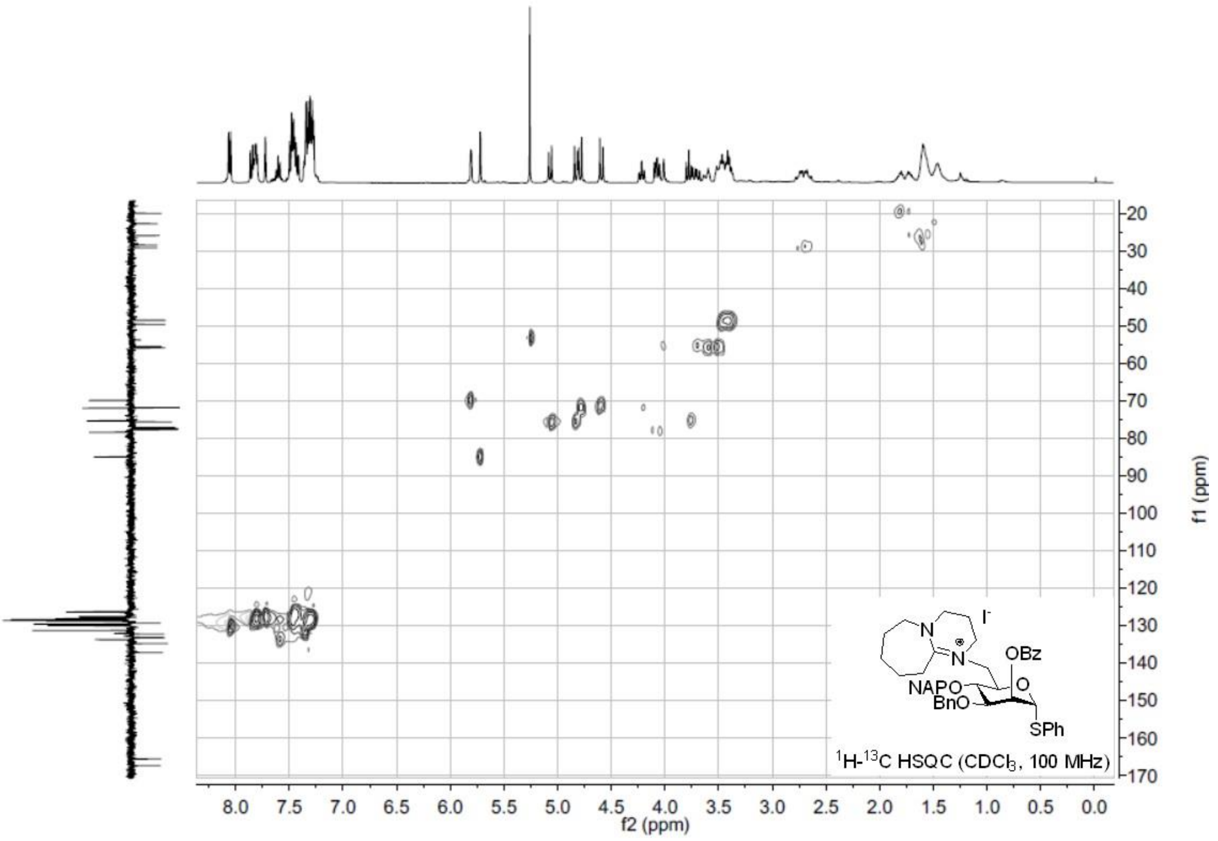

OBz
NAPO
BnO
O
SPh
I⁻
N
^{1}H-^{13}C HSQC (CDCl$_3$, 100 MHz)
f2 (ppm)
f1 (ppm)

^{1}H and ^{13}C NMR spectra of compound **51**

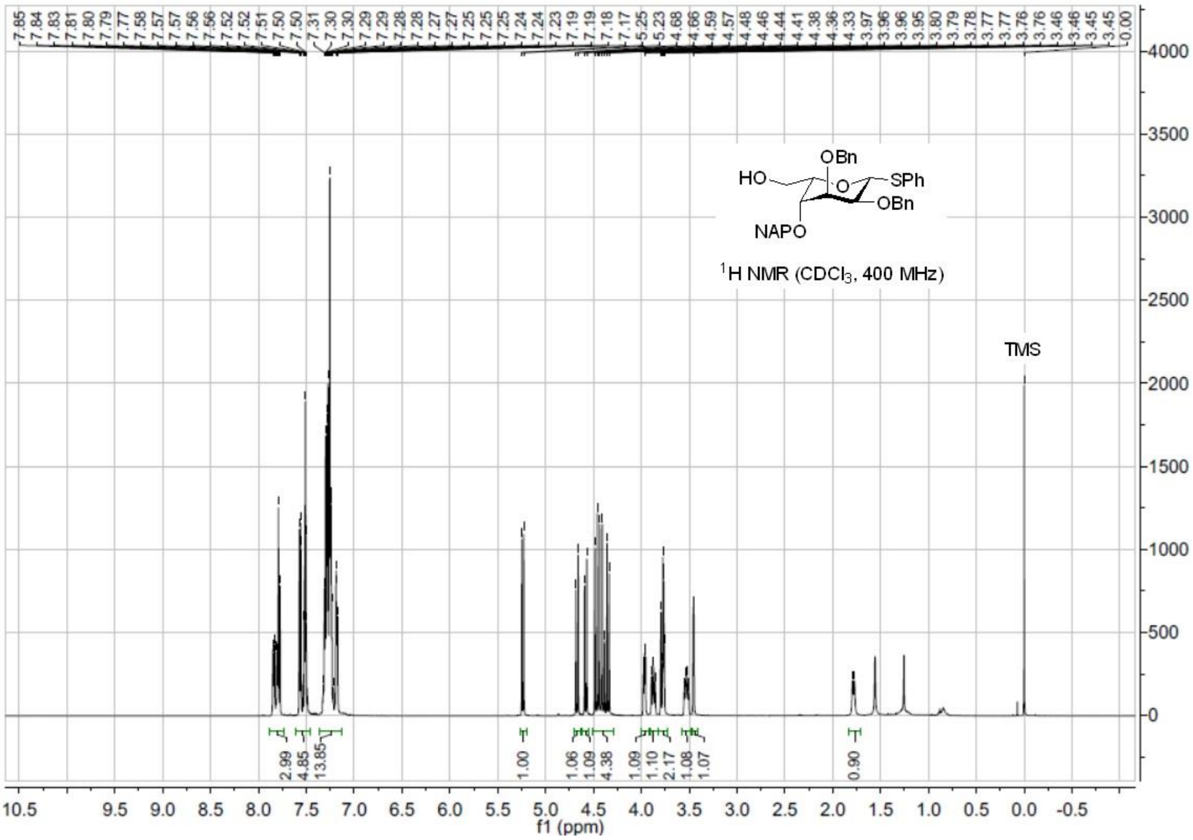

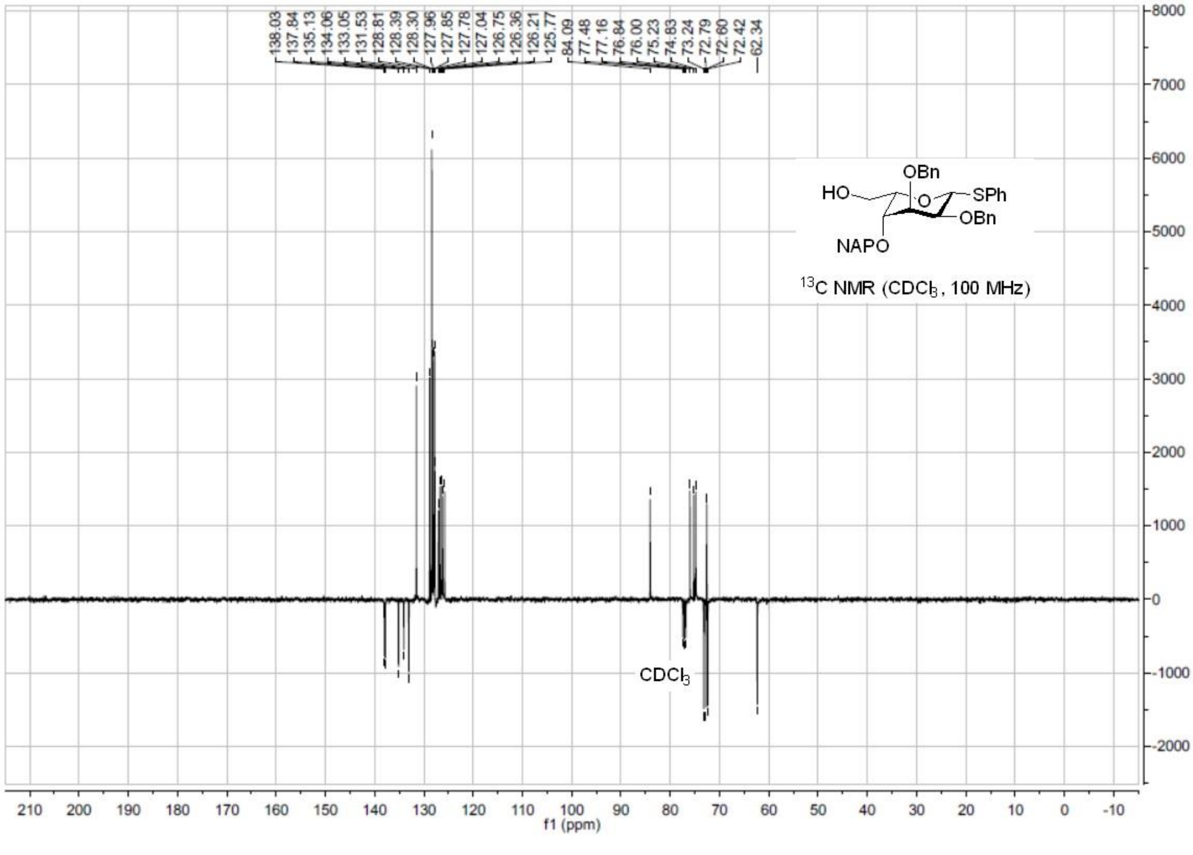

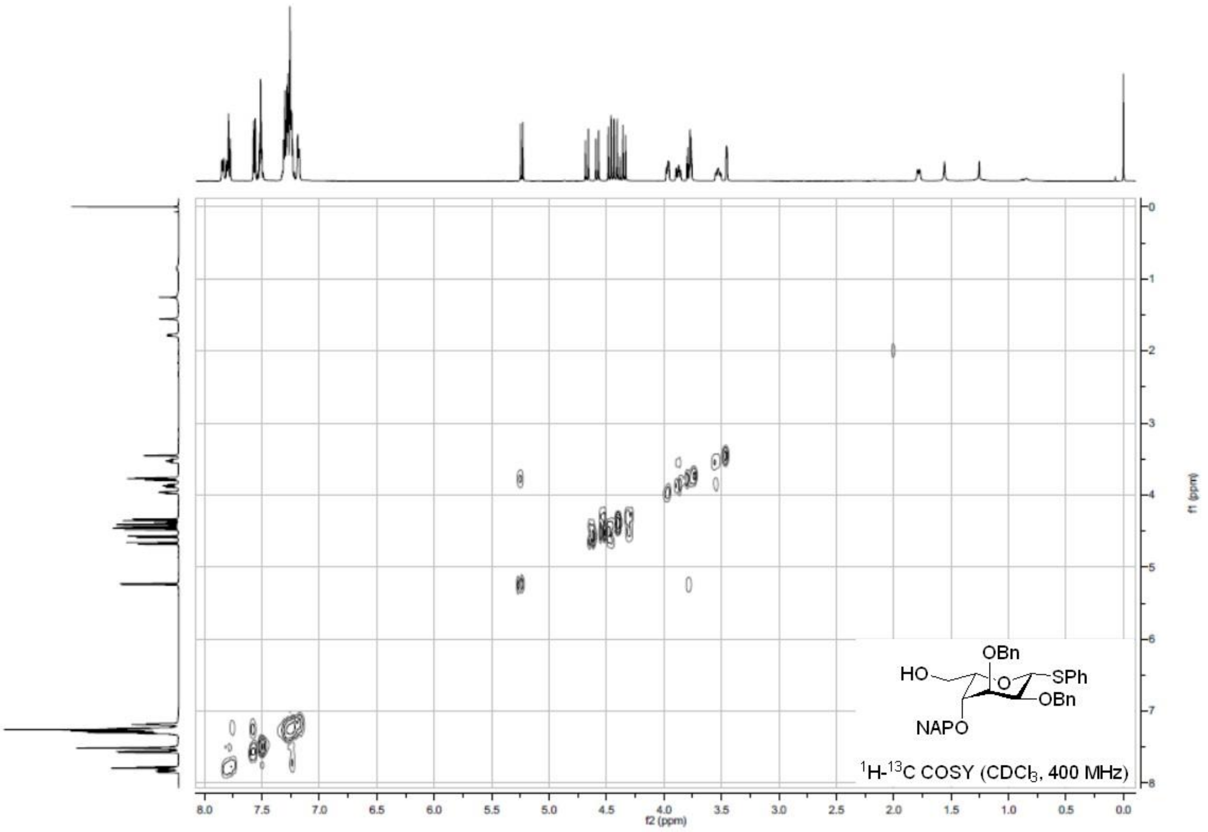
OBn
HO
O
SPh
OBn
NAPO
1H-13C COSY (CDCl3, 400 MHz)
f2 (ppm)
f1 (ppm)

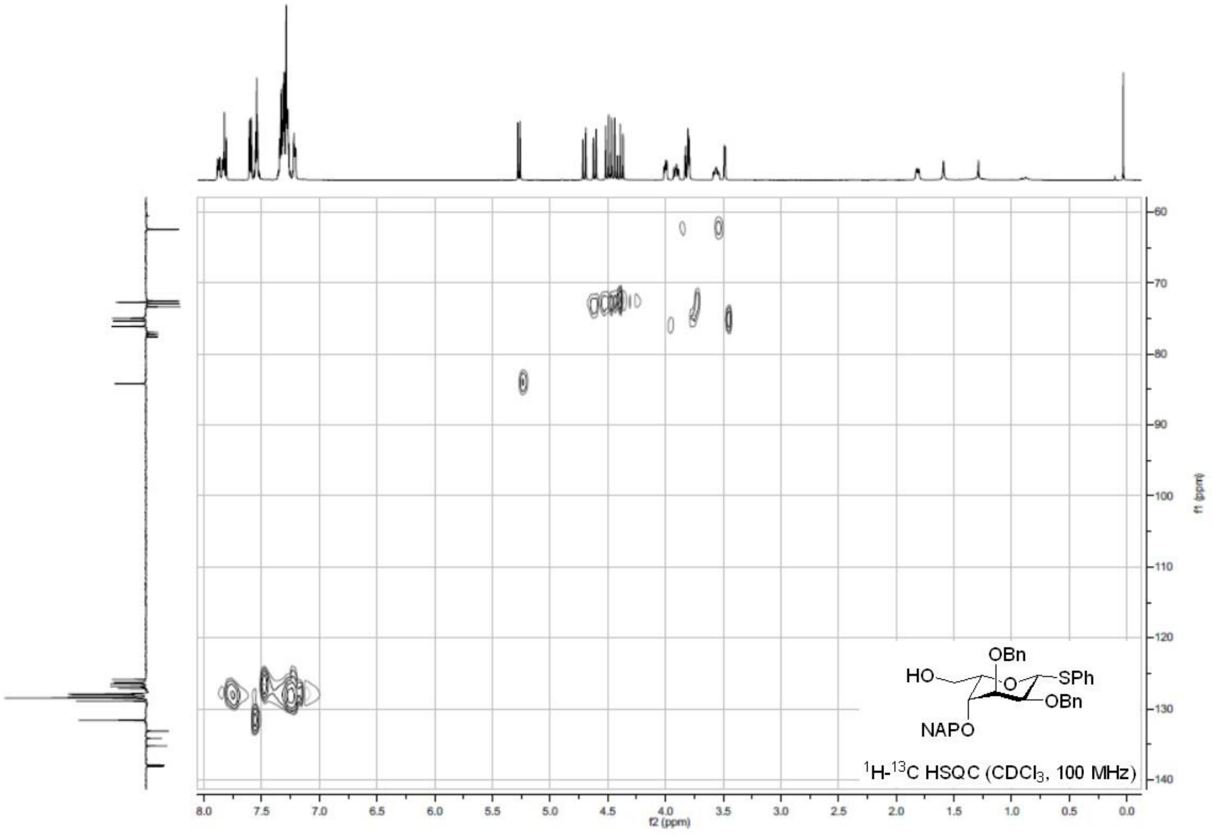
OBn
HO
O
SPh
OBn
NAPO
1H-13C HSQC (CDCl3, 100 MHz)
f2 (ppm)
f1 (ppm)

^{1}H and ^{13}C NMR spectra of compound **52**

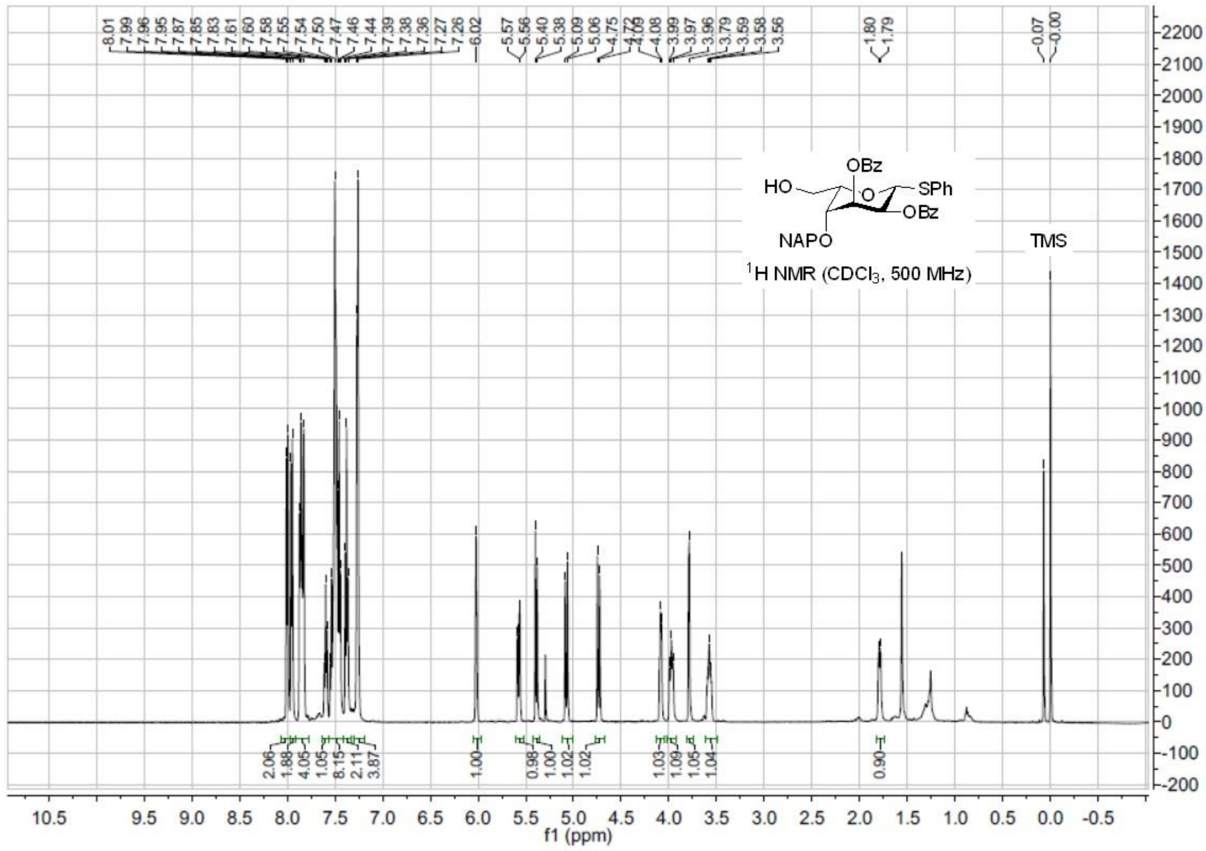

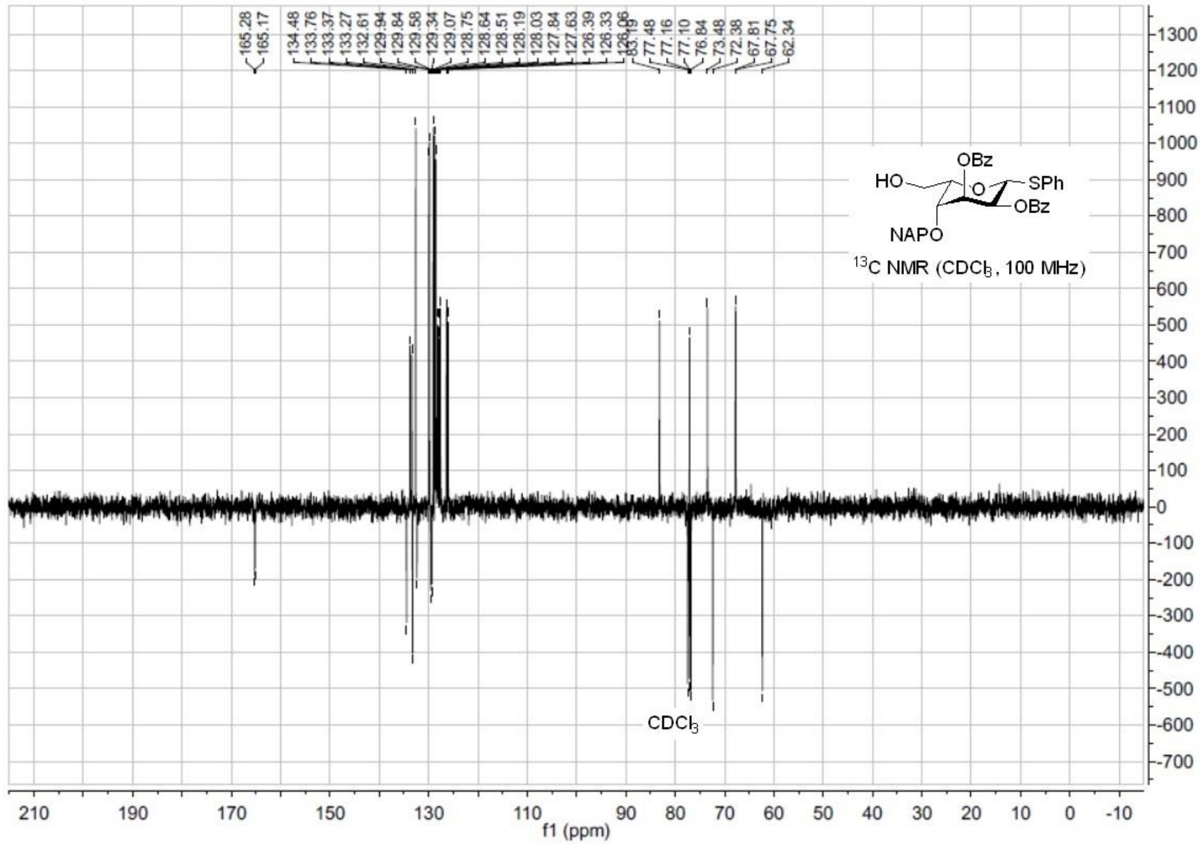

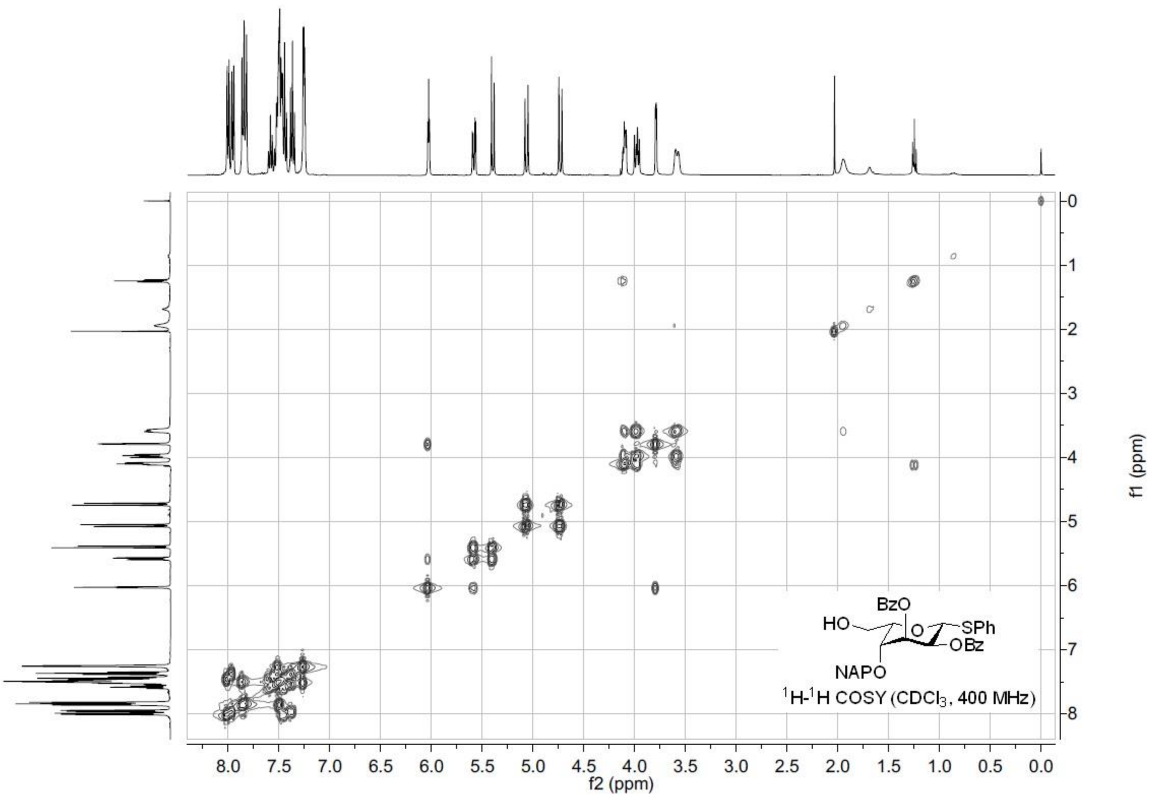
BzO
HO
O
SPh
OBz
NAPO
^{1}H-^{1}H COSY (CDCl$_3$, 400 MHz)
f1 (ppm)
f2 (ppm)

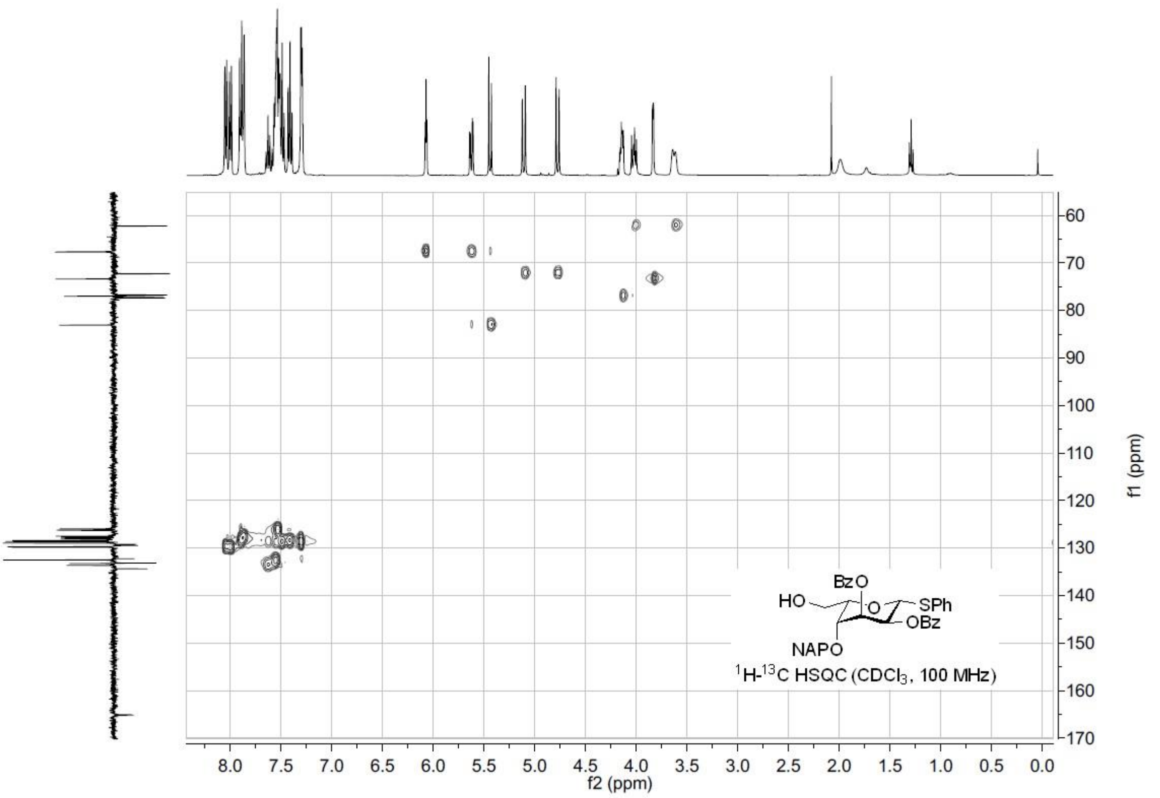
BzO
HO
O
SPh
OBz
NAPO
^{1}H-^{13}C HSQC (CDCl$_3$, 100 MHz)
f1 (ppm)
f2 (ppm)

^{1}H and ^{13}C NMR spectra of compound **53**

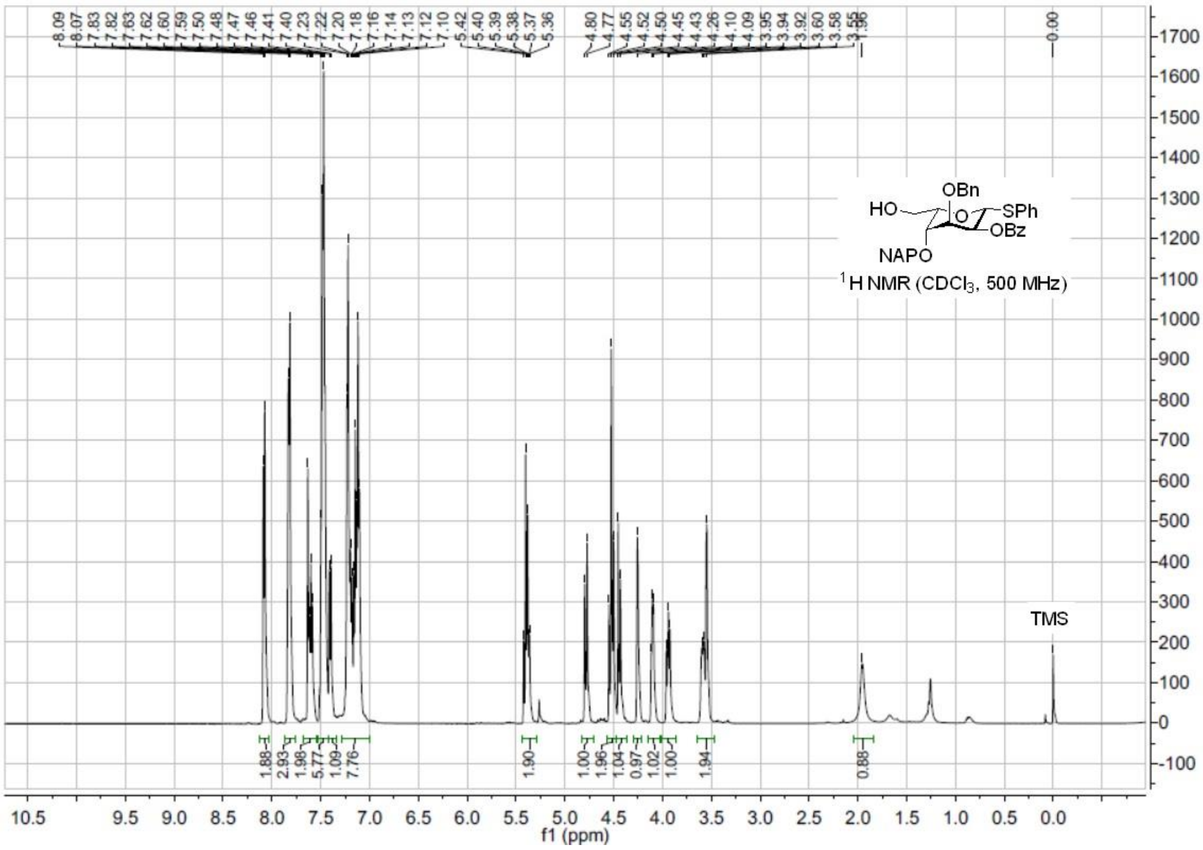

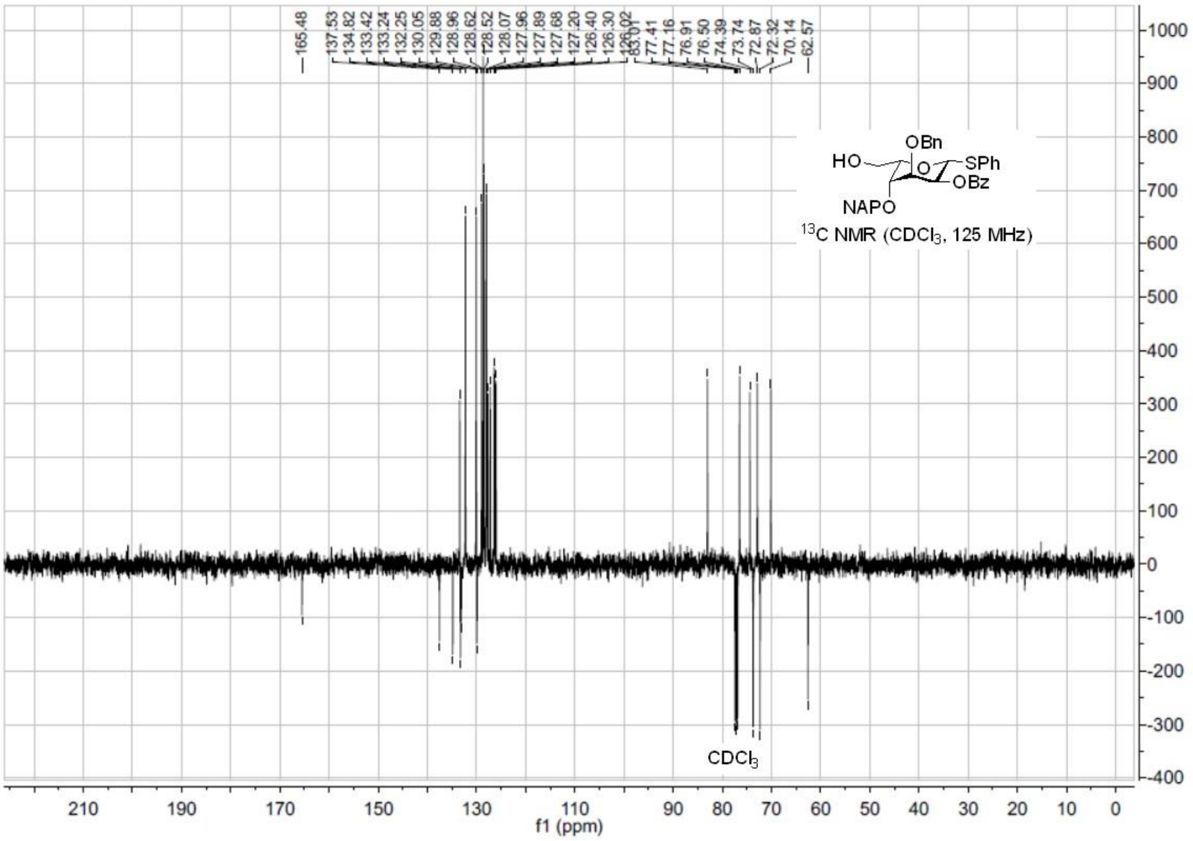

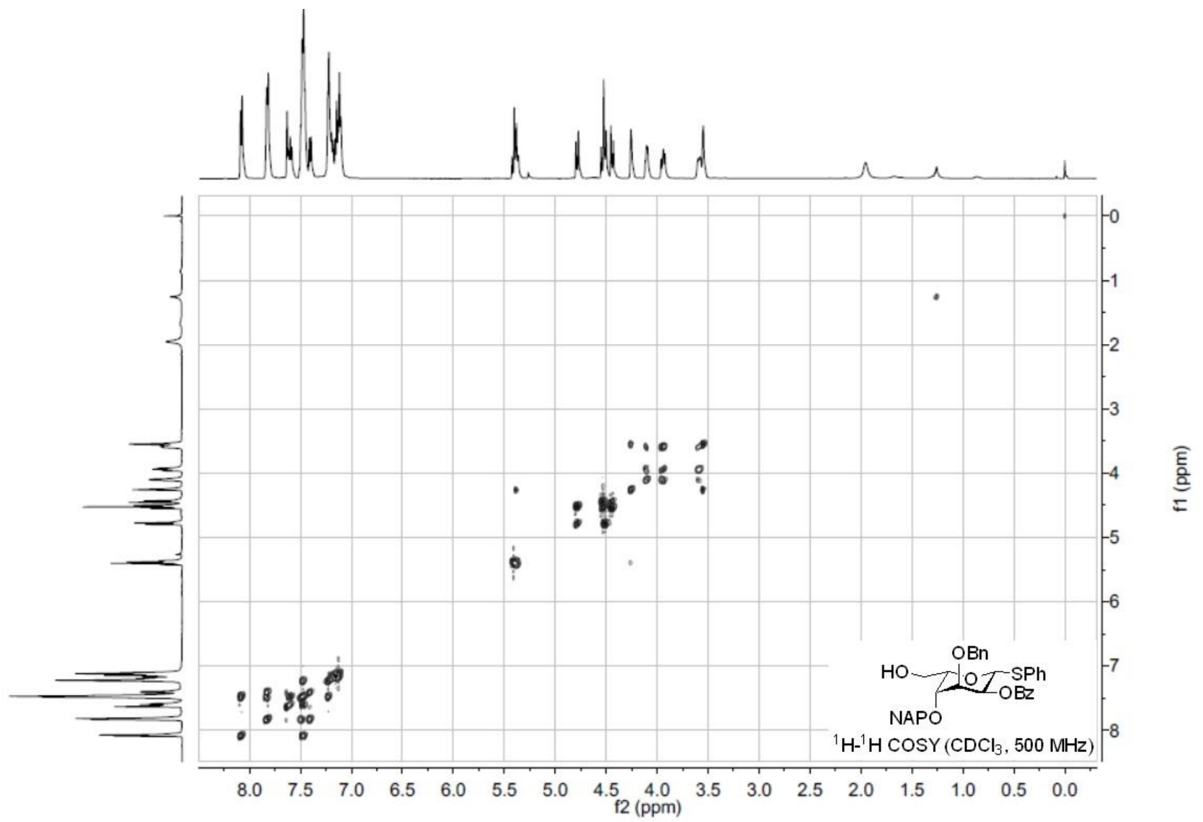

OBn
HO
O
SPh
OBz
NAPO
1H-1H COSY (CDCl3, 500 MHz)
f2 (ppm)
f1 (ppm)

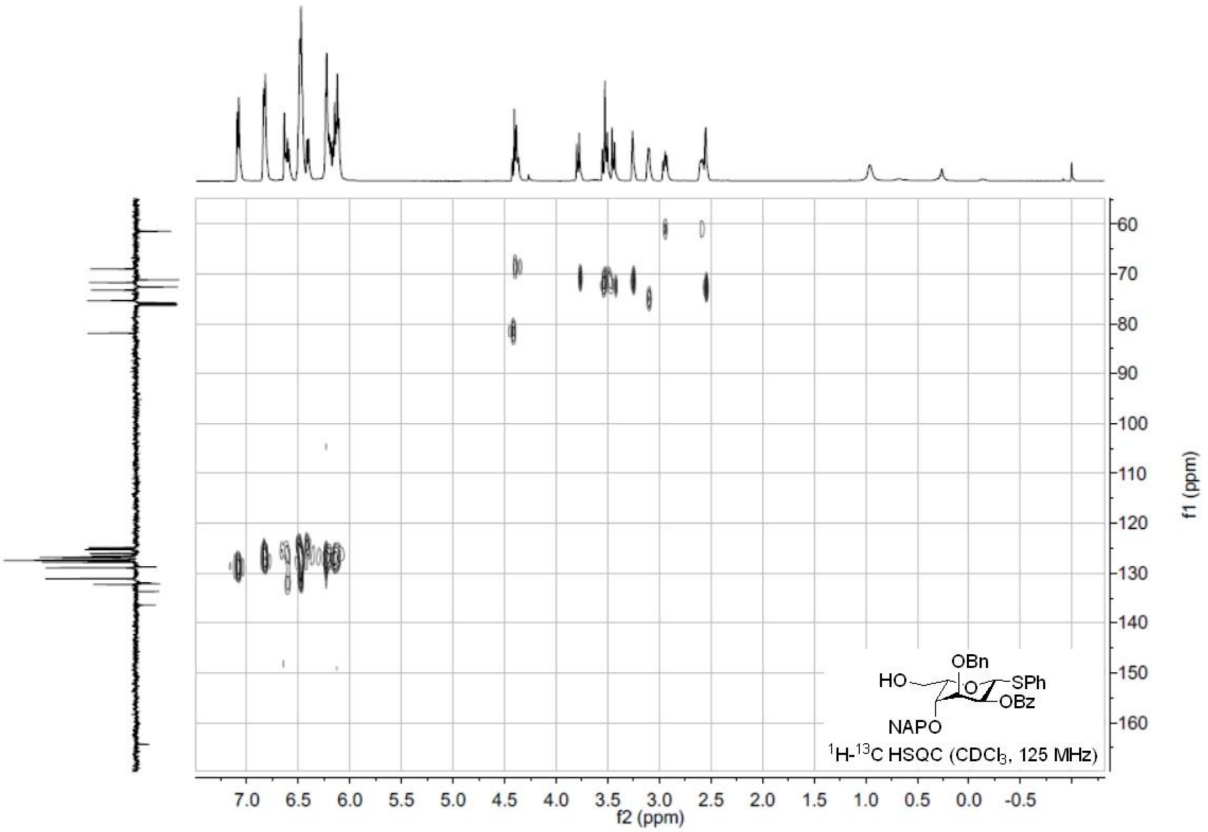

OBn
HO
O
SPh
OBz
NAPO
1H-13C HSQC (CDCl3, 125 MHz)
f2 (ppm)
f1 (ppm)

^{1}H and ^{13}C NMR spectra of compound **54**

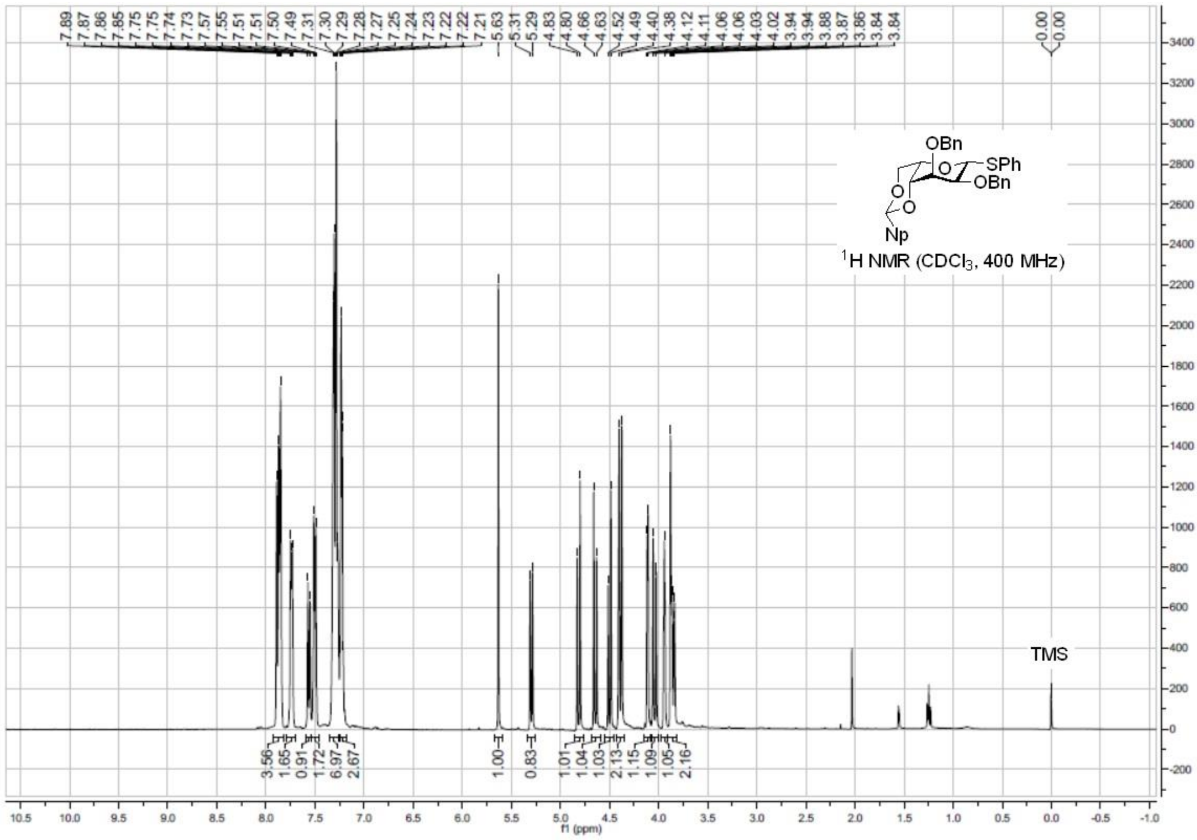

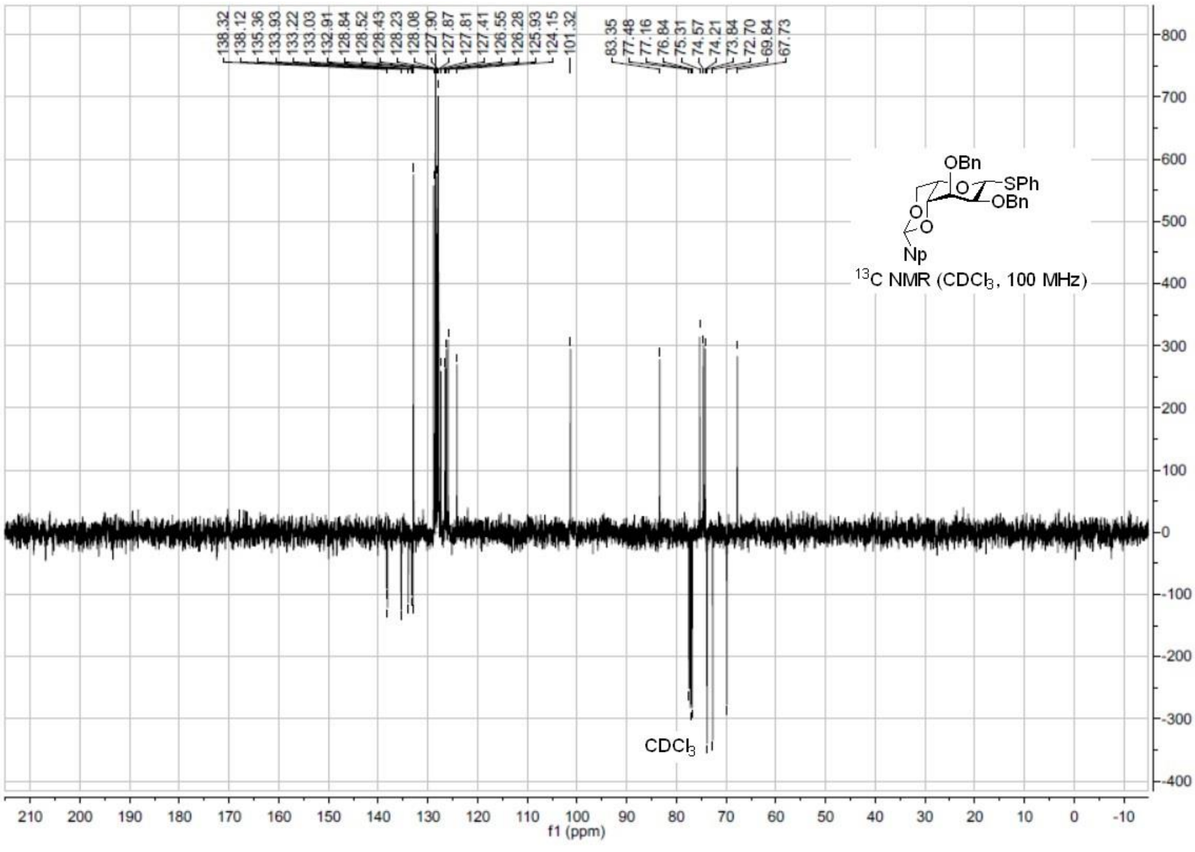

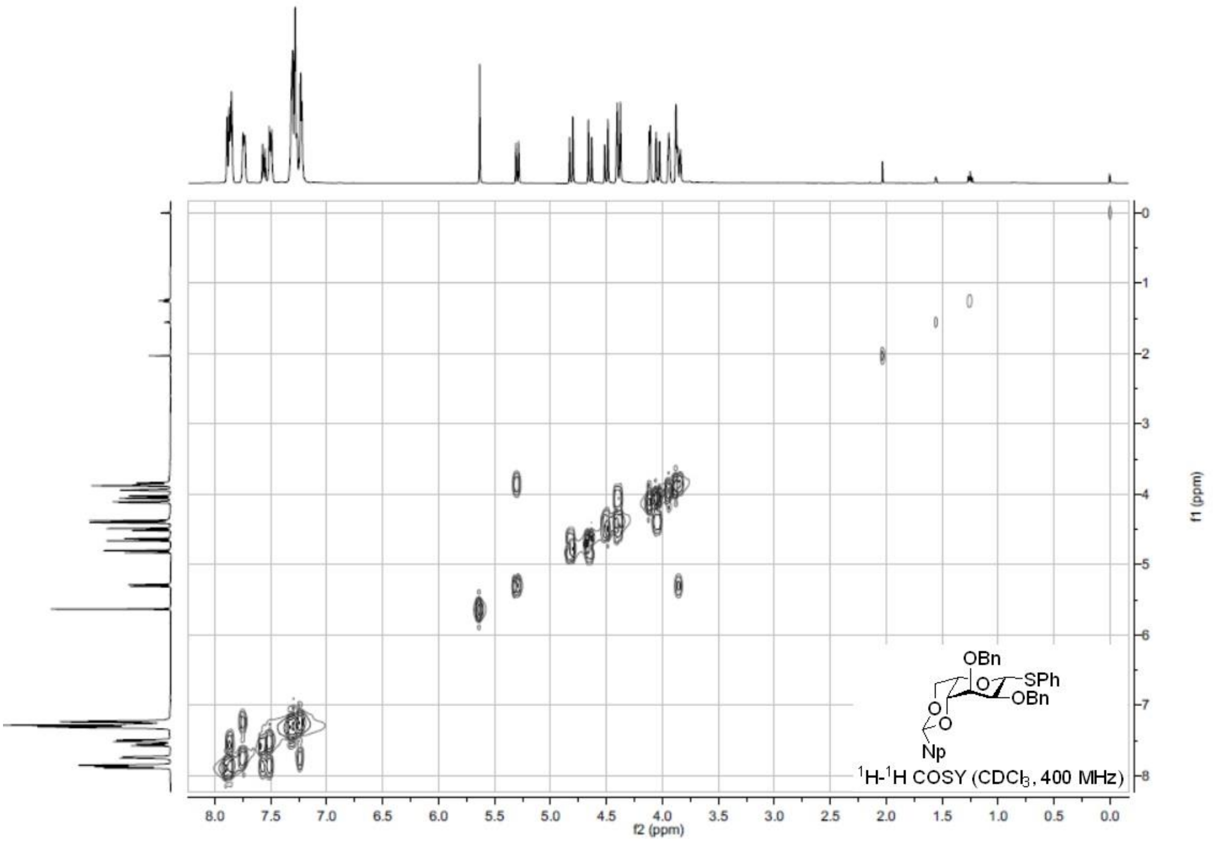

OBn
SPh
OBn
Np
$^1H-^1H$ COSY ($CDCl_3$, 400 MHz)
f2 (ppm)
f1 (ppm)

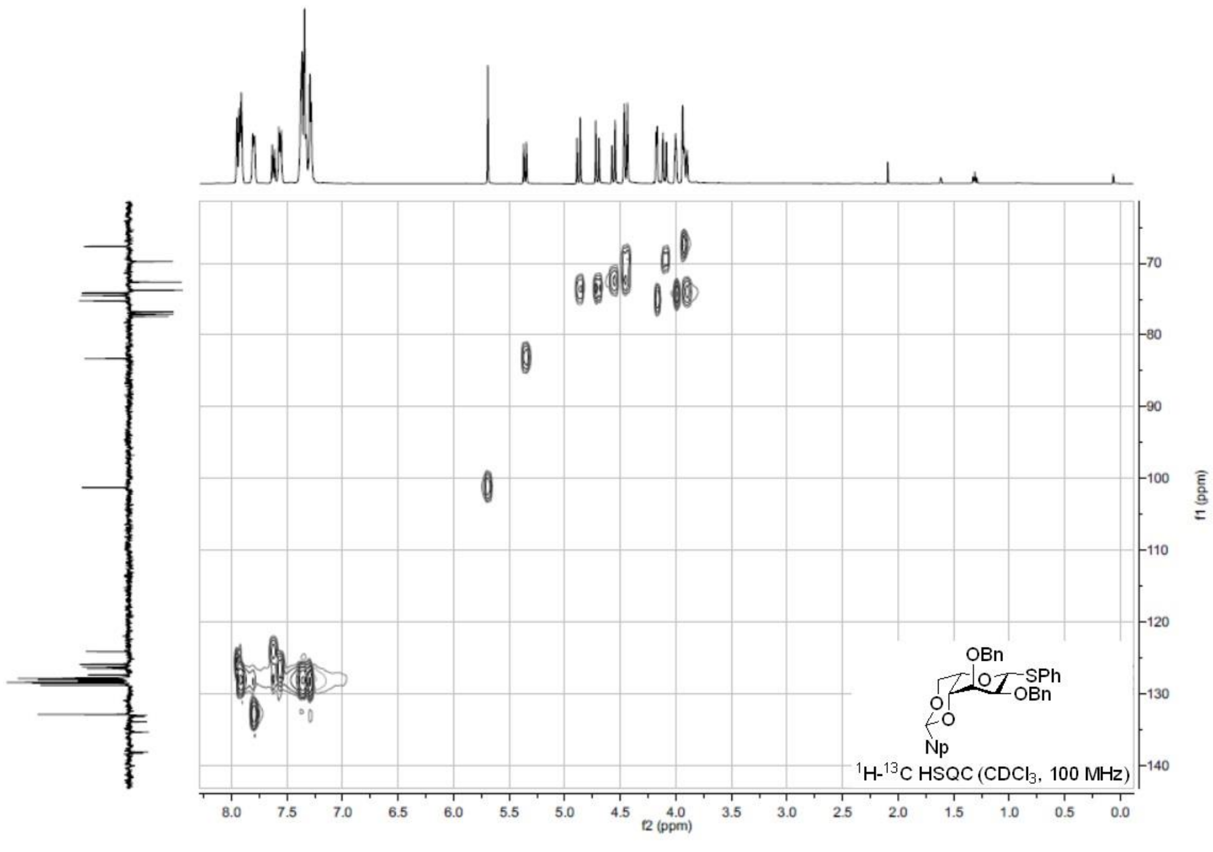

OBn
SPh
OBn
Np
$^1H-^{13}C$ HSQC ($CDCl_3$, 100 MHz)
f2 (ppm)
f1 (ppm)

^{1}H and ^{13}C NMR spectra of compound **55**

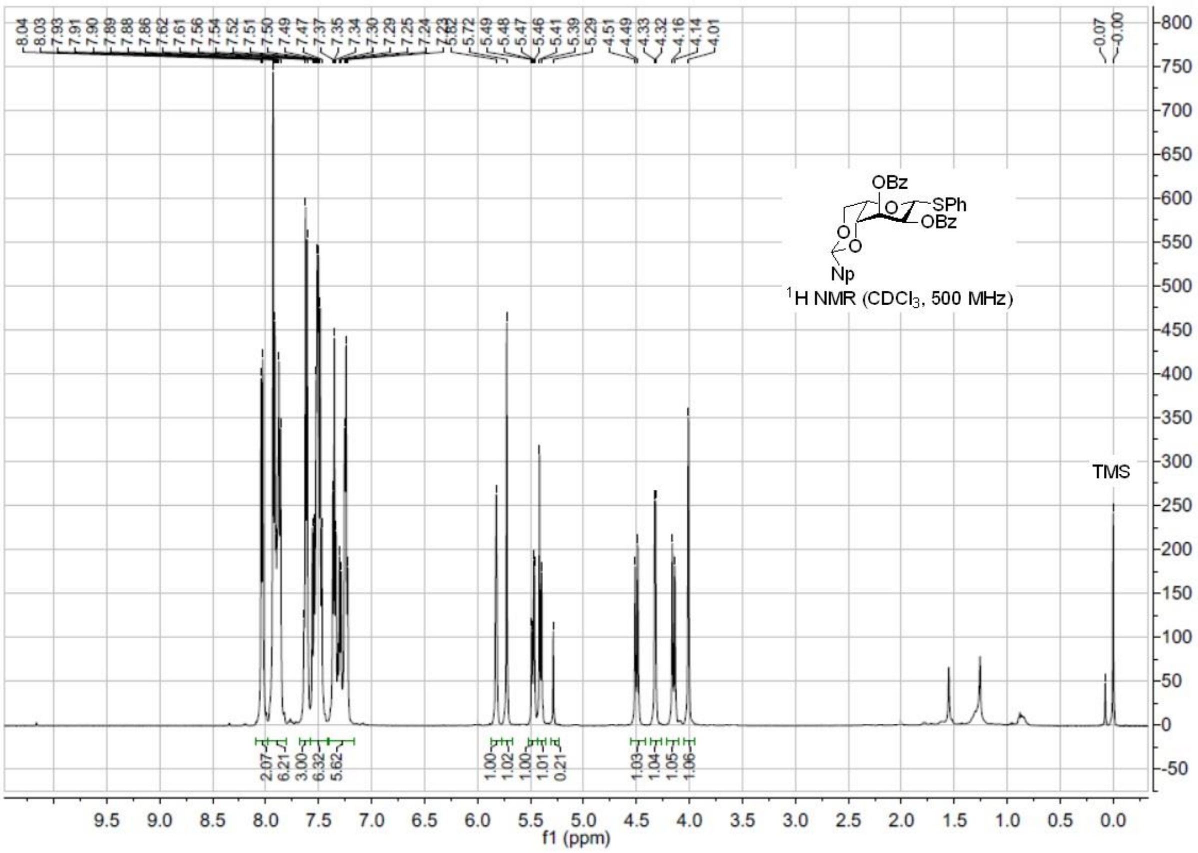

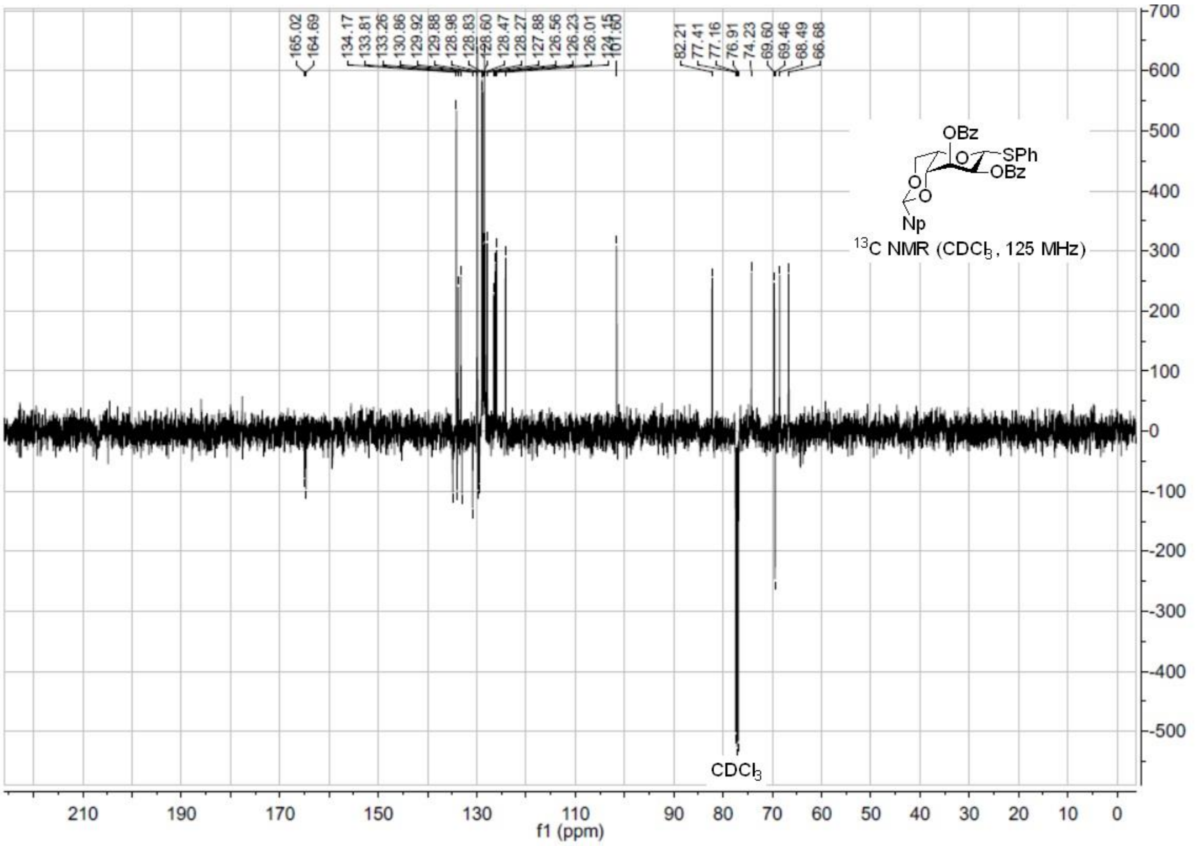

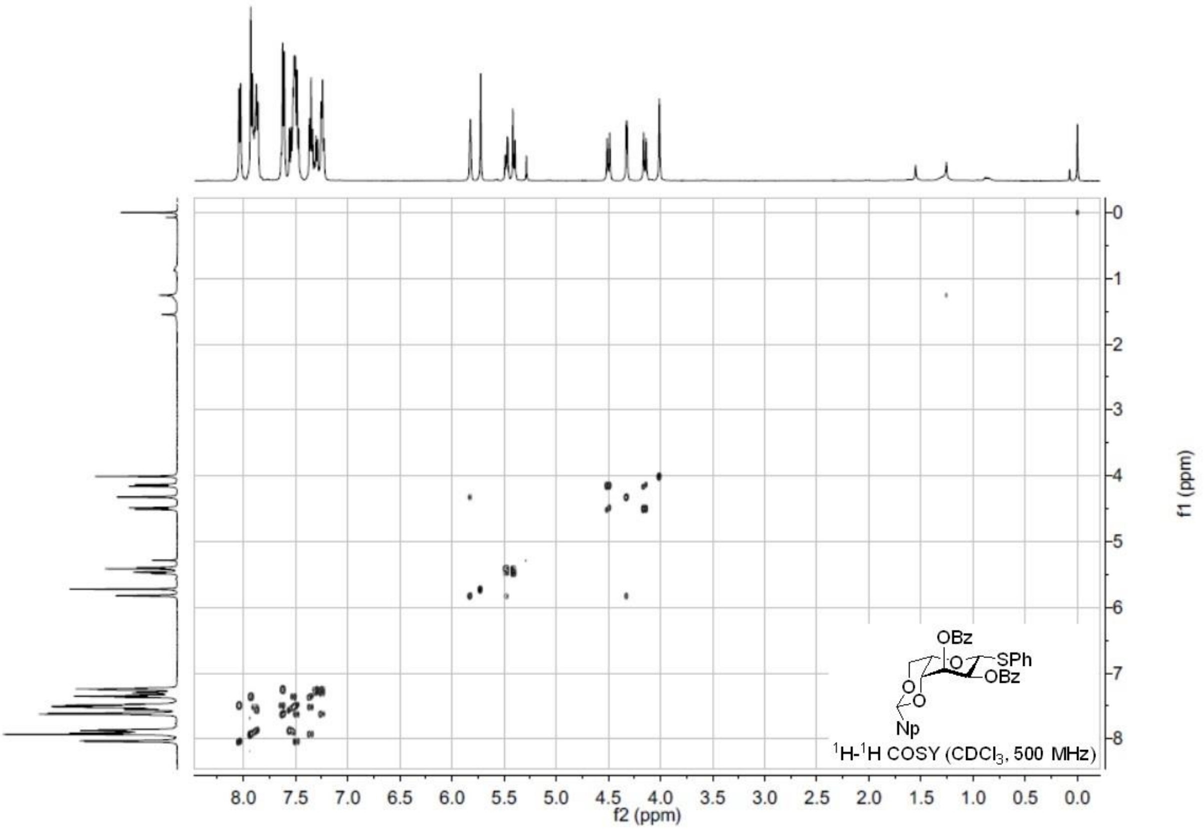
OBz
SPh
OBz
O
O
O
Np
1H-1H COSY (CDCl3, 500 MHz)
f2 (ppm)
f1 (ppm)

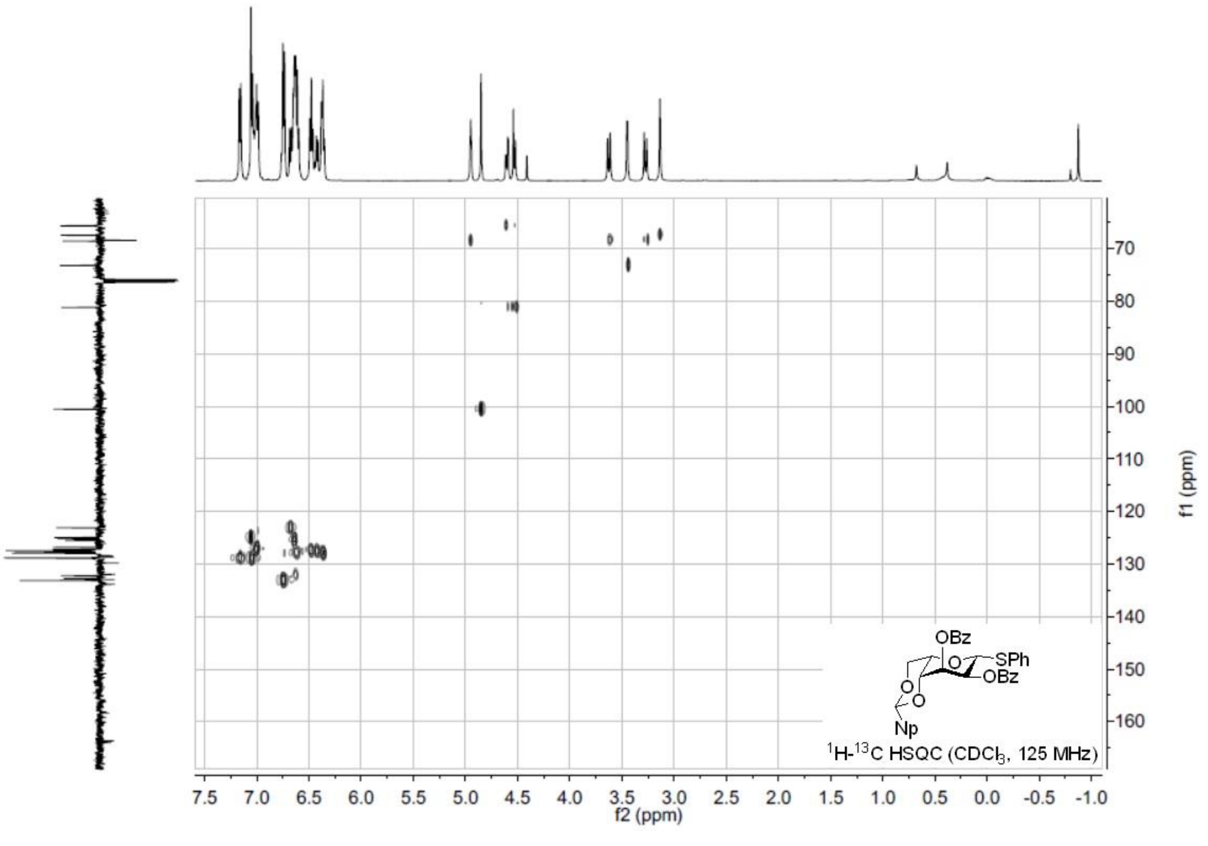
OBz
SPh
OBz
O
O
O
Np
1H-13C HSQC (CDCl3, 125 MHz)
f2 (ppm)
f1 (ppm)

^{1}H and ^{13}C NMR spectra of compound **56**

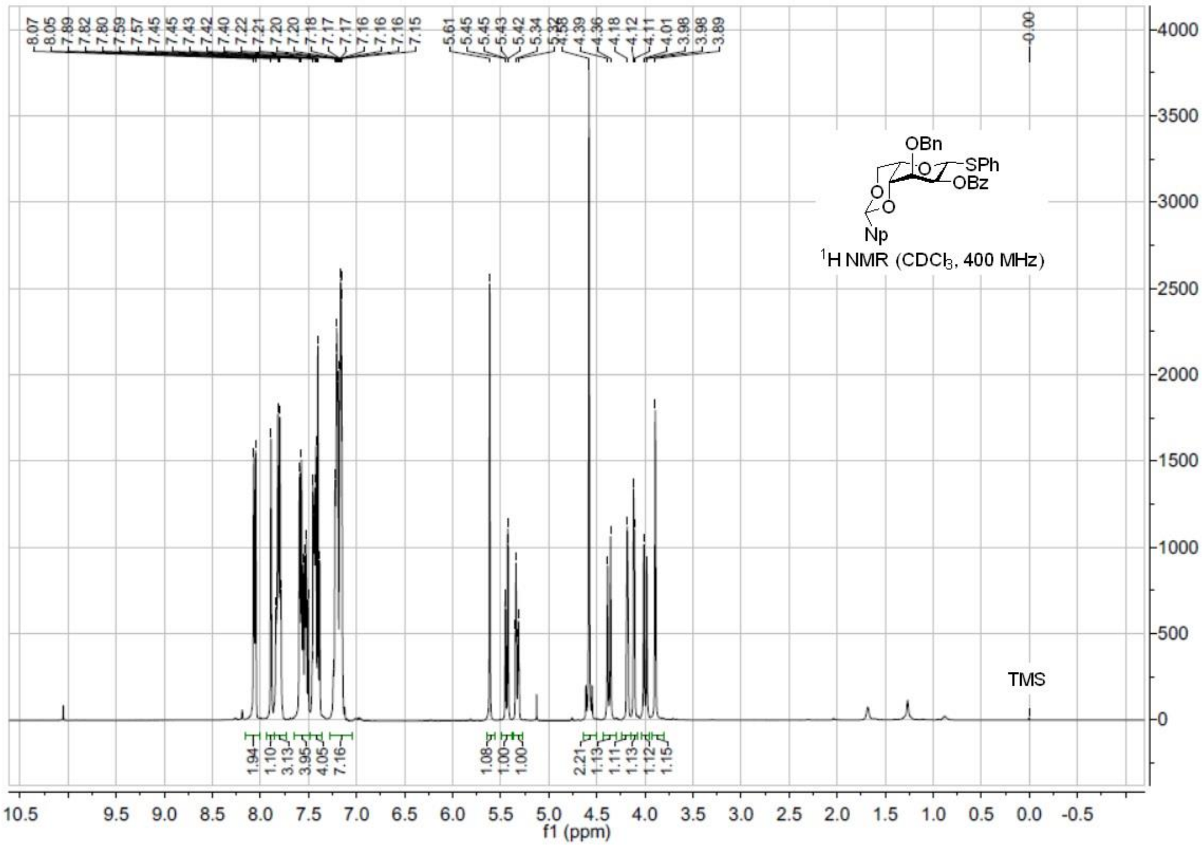

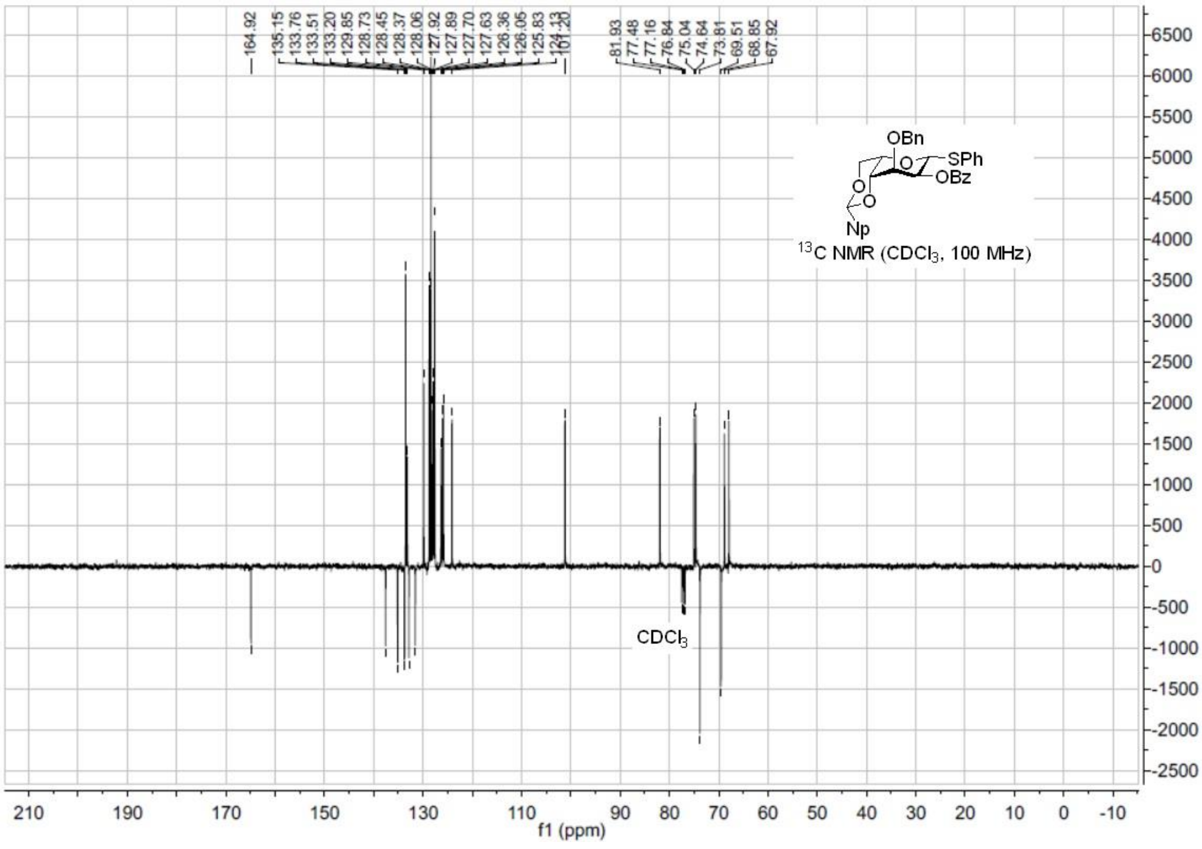

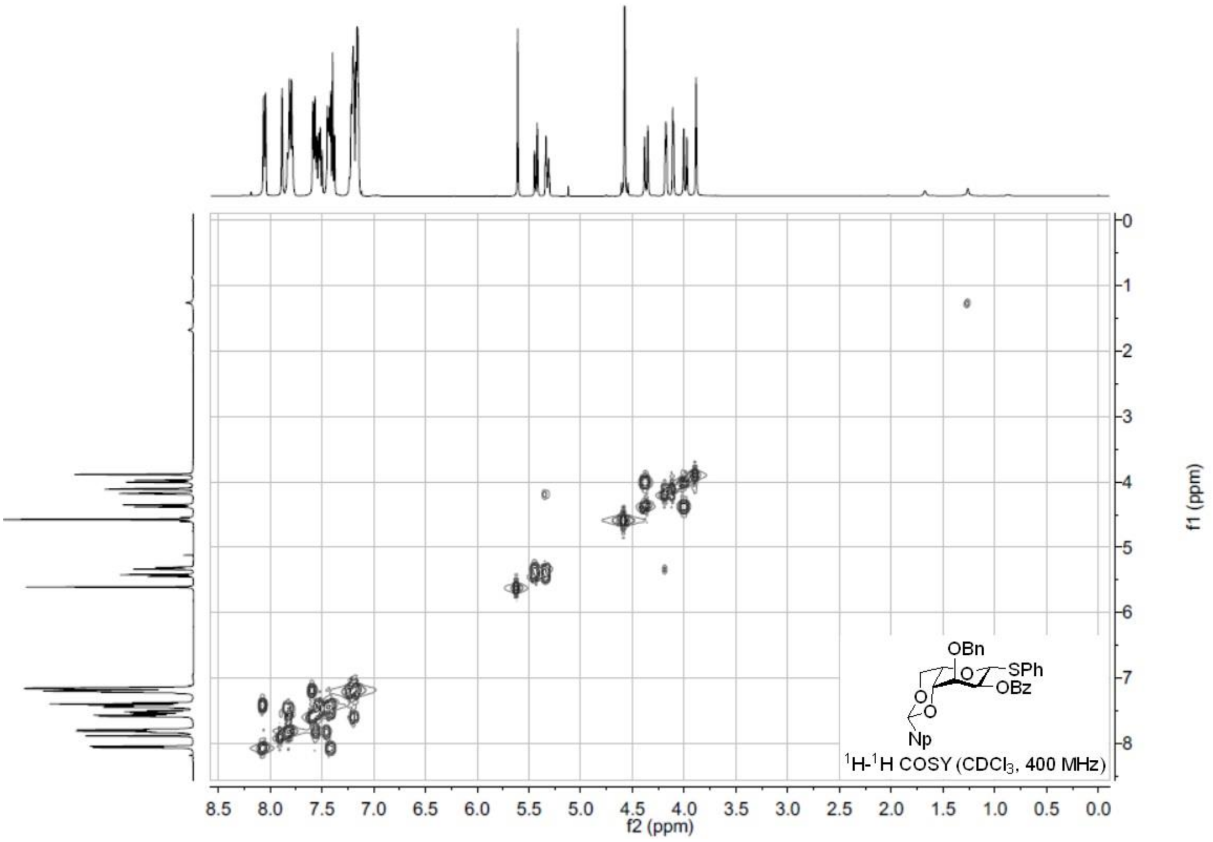
OBn
SPh
OBz
O
O
O
Np
^{1}H-^{1}H COSY (CDCl$_3$, 400 MHz)
f1 (ppm)
f2 (ppm)

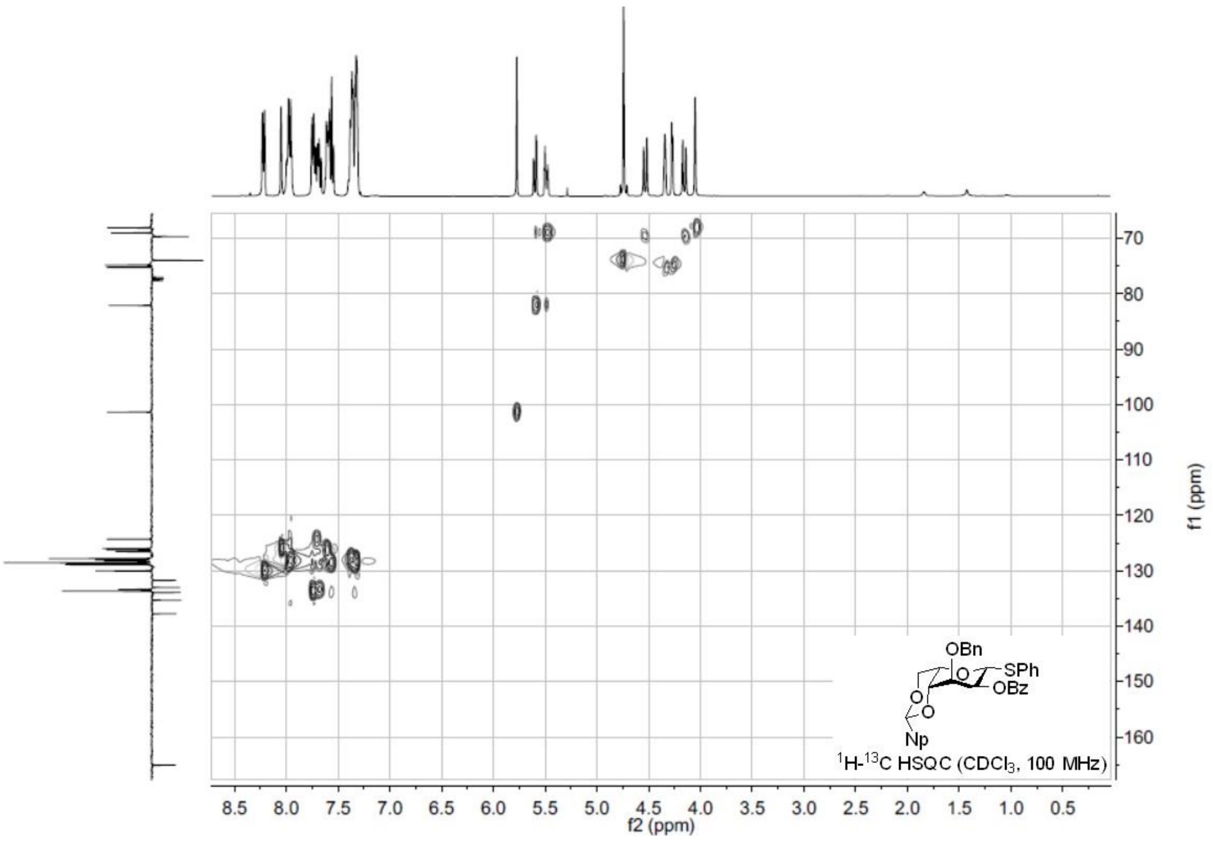
OBn
SPh
OBz
O
O
O
Np
^{1}H-^{13}C HSQC (CDCl$_3$, 100 MHz)
f1 (ppm)
f2 (ppm)

^{1}H and ^{13}C NMR spectra of compound **57**

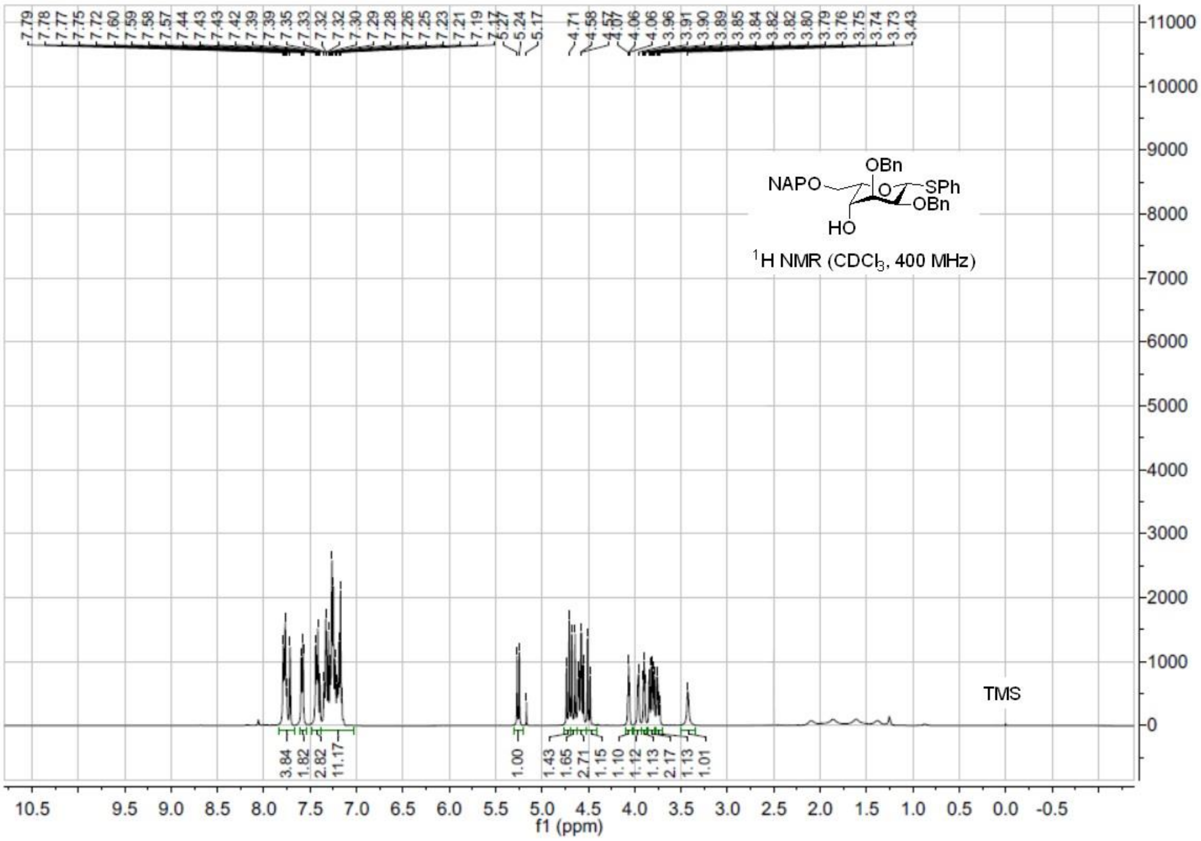

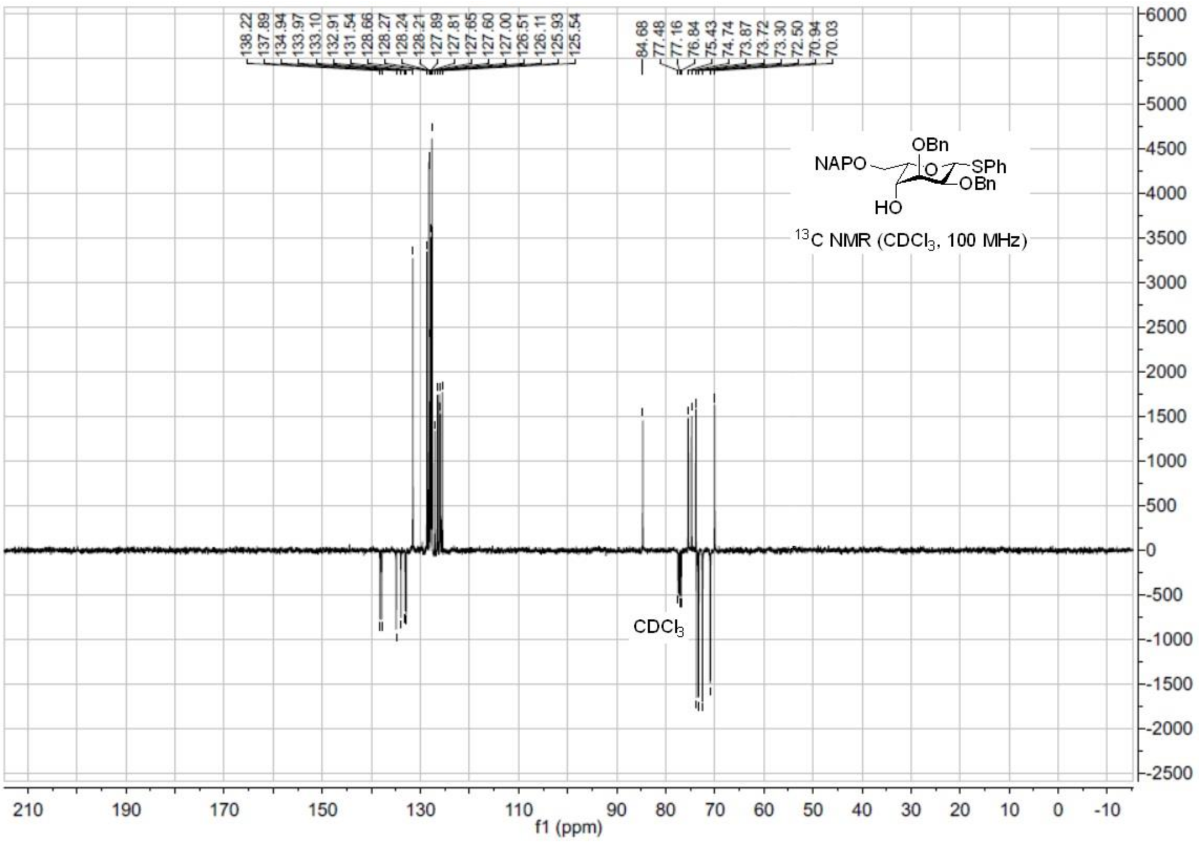

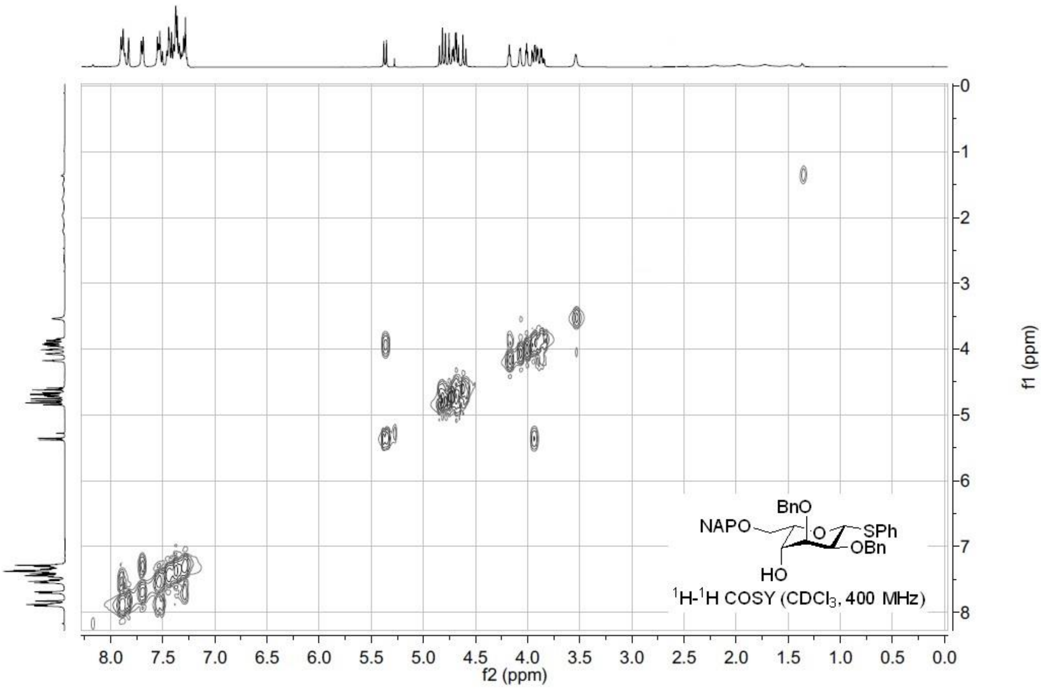
NAPO
BnO
O
SPh
OBn
HO
^{1}H-^{1}H COSY (CDCl$_3$, 400 MHz)
f2 (ppm)
f1 (ppm)

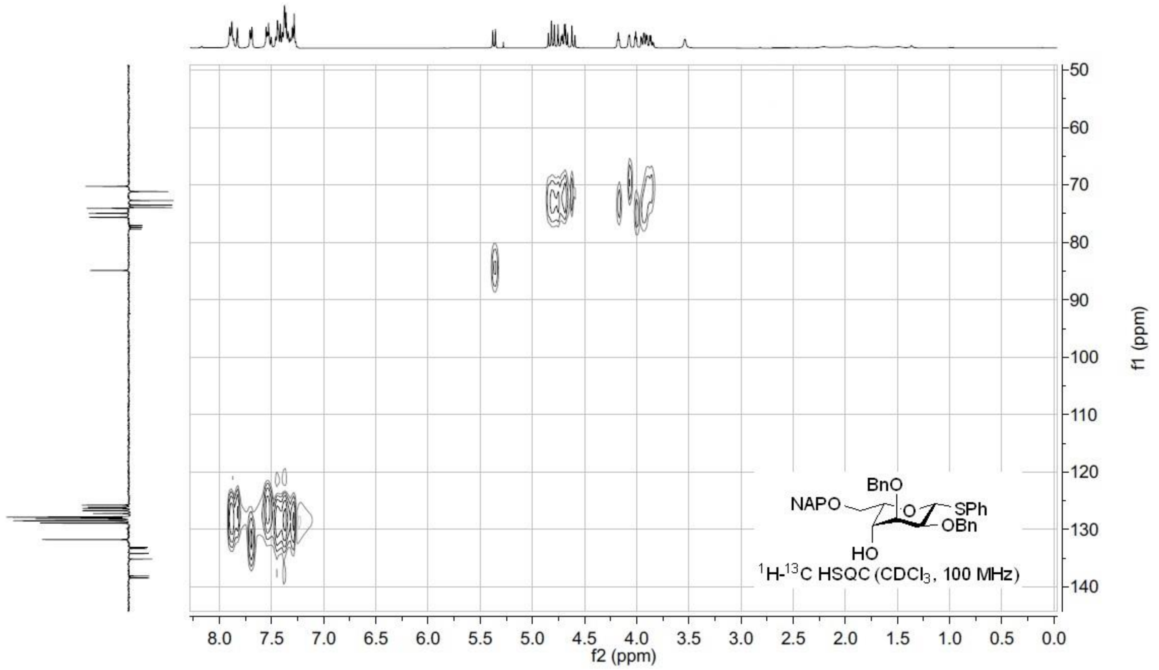
NAPO
BnO
O
SPh
OBn
HO
^{1}H-^{13}C HSQC (CDCl$_3$, 100 MHz)
f2 (ppm)
f1 (ppm)

^{1}H and ^{13}C NMR spectra of compound **58**

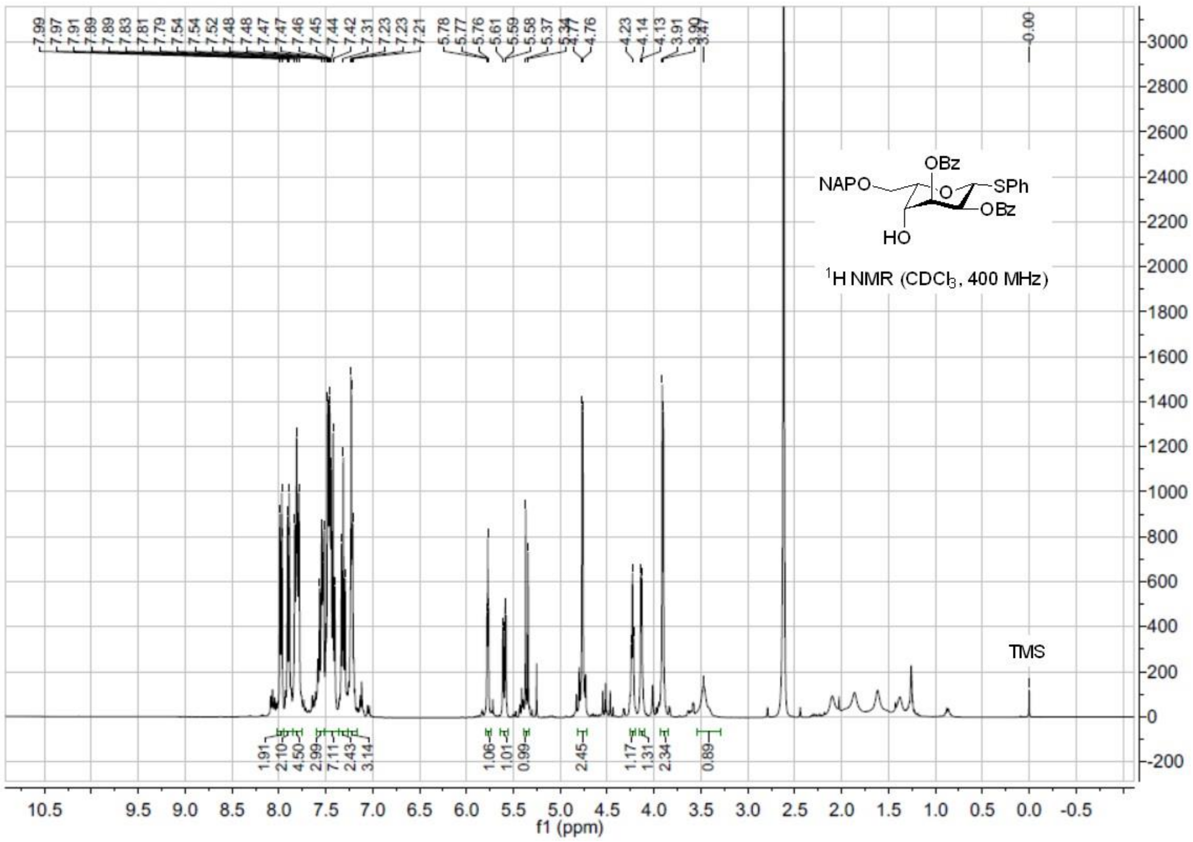

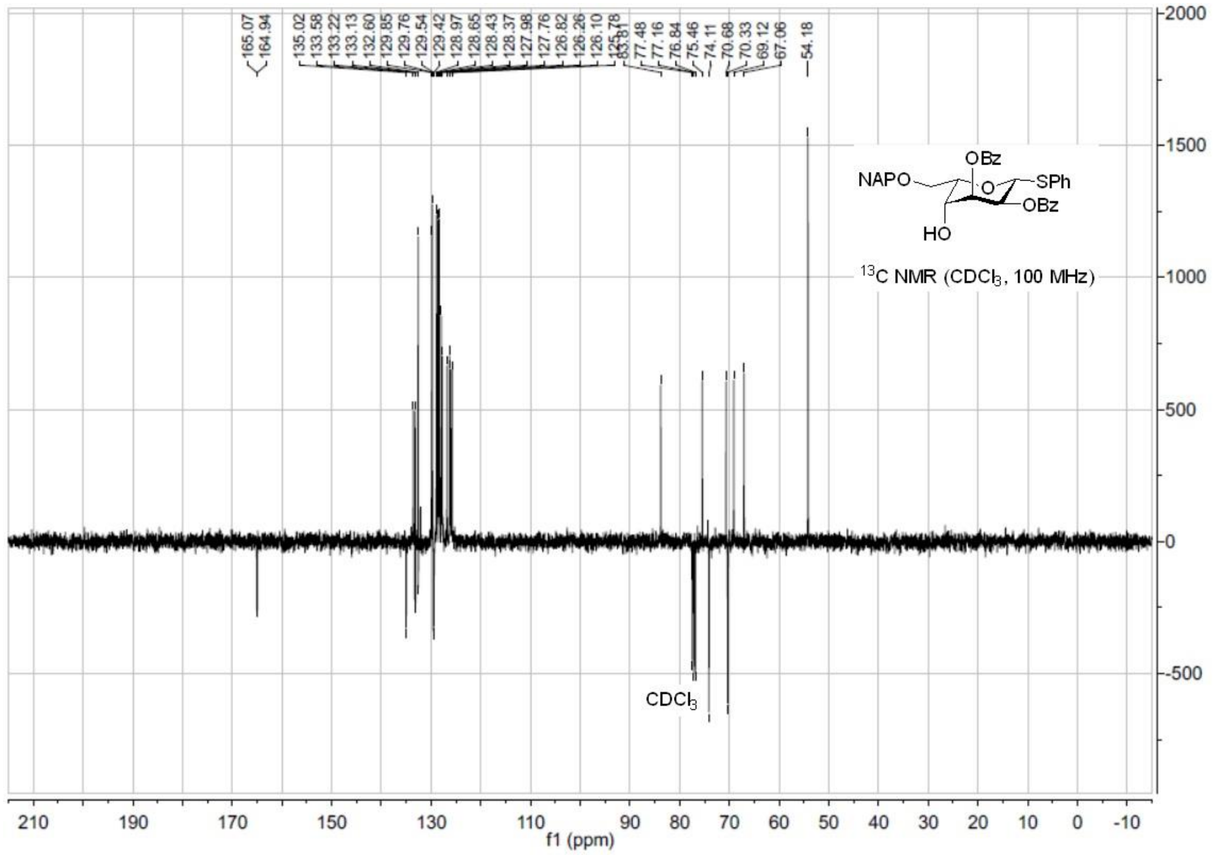

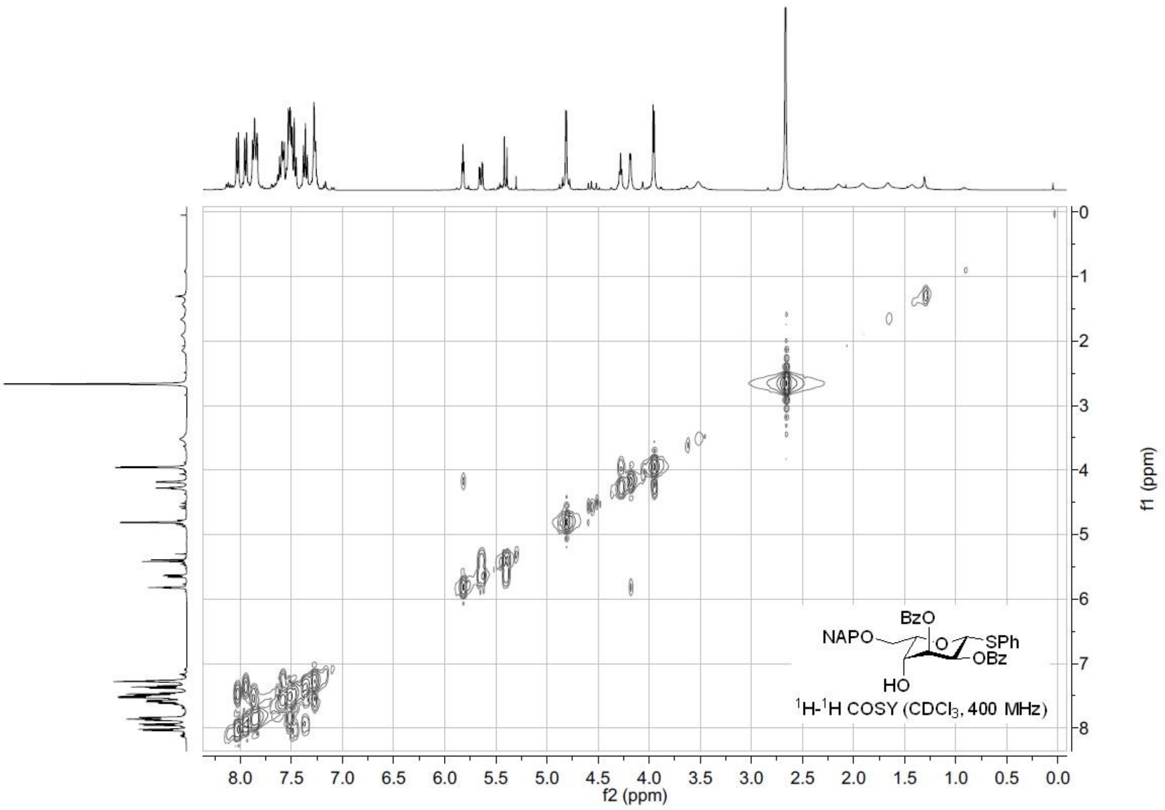
BzO
NAPO
O
SPh
OBz
HO
1H-1H COSY (CDCl3, 400 MHz)
f2 (ppm)
f1 (ppm)

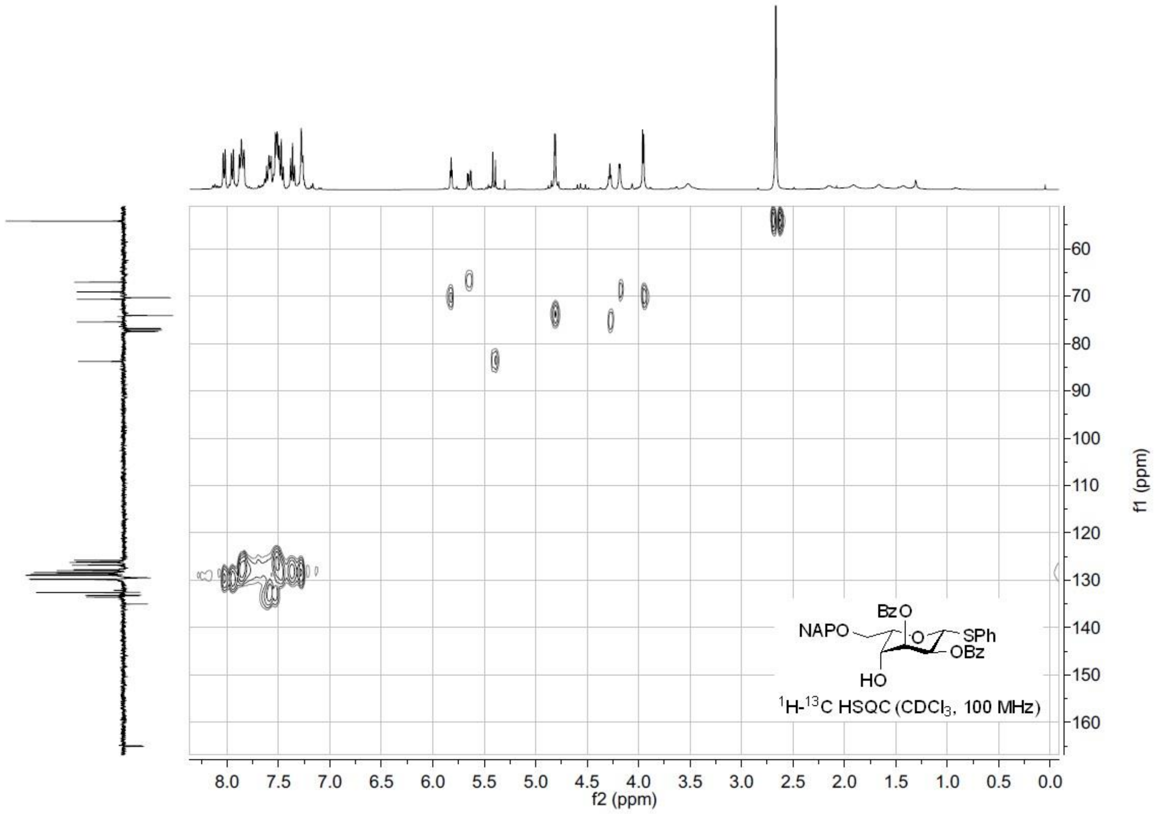
BzO
NAPO
O
SPh
OBz
HO
1H-13C HSQC (CDCl3, 100 MHz)
f2 (ppm)
f1 (ppm)

^{1}H and ^{13}C NMR spectra of compound **59**

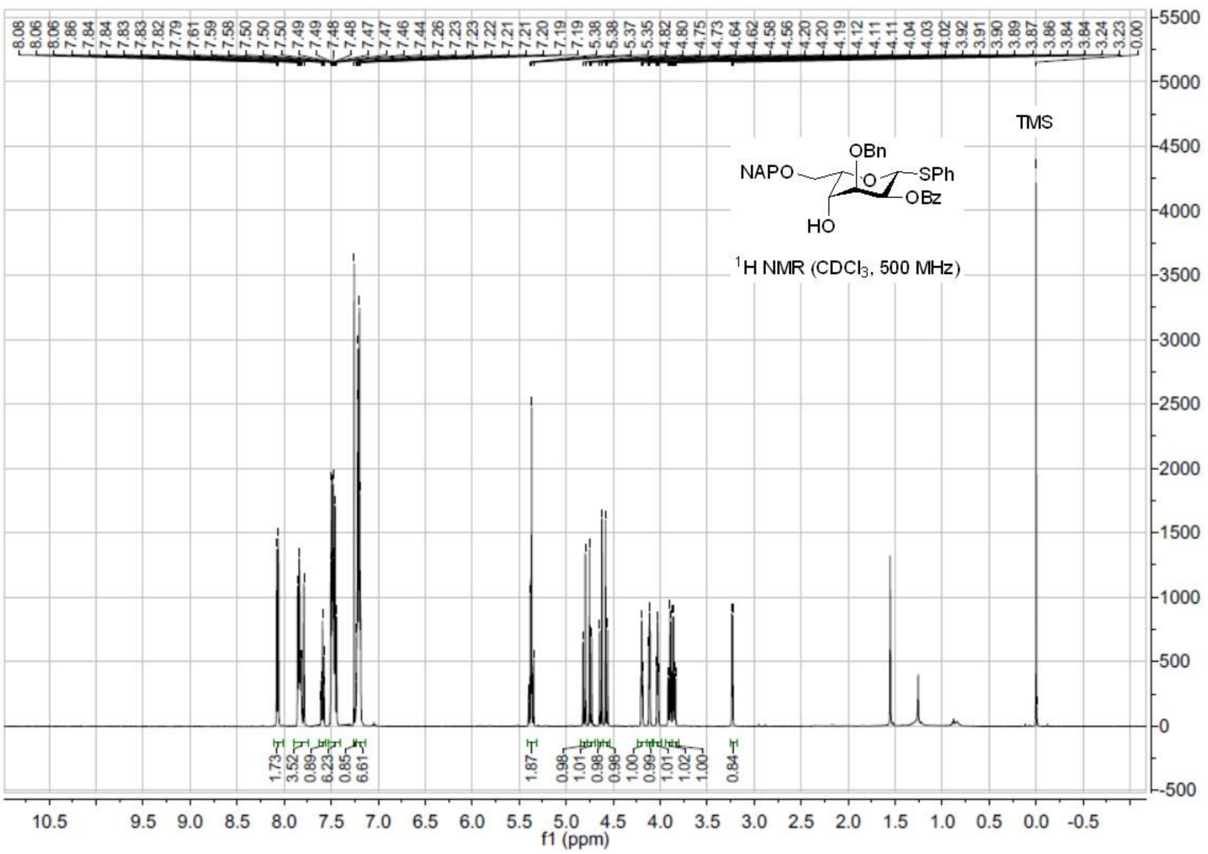

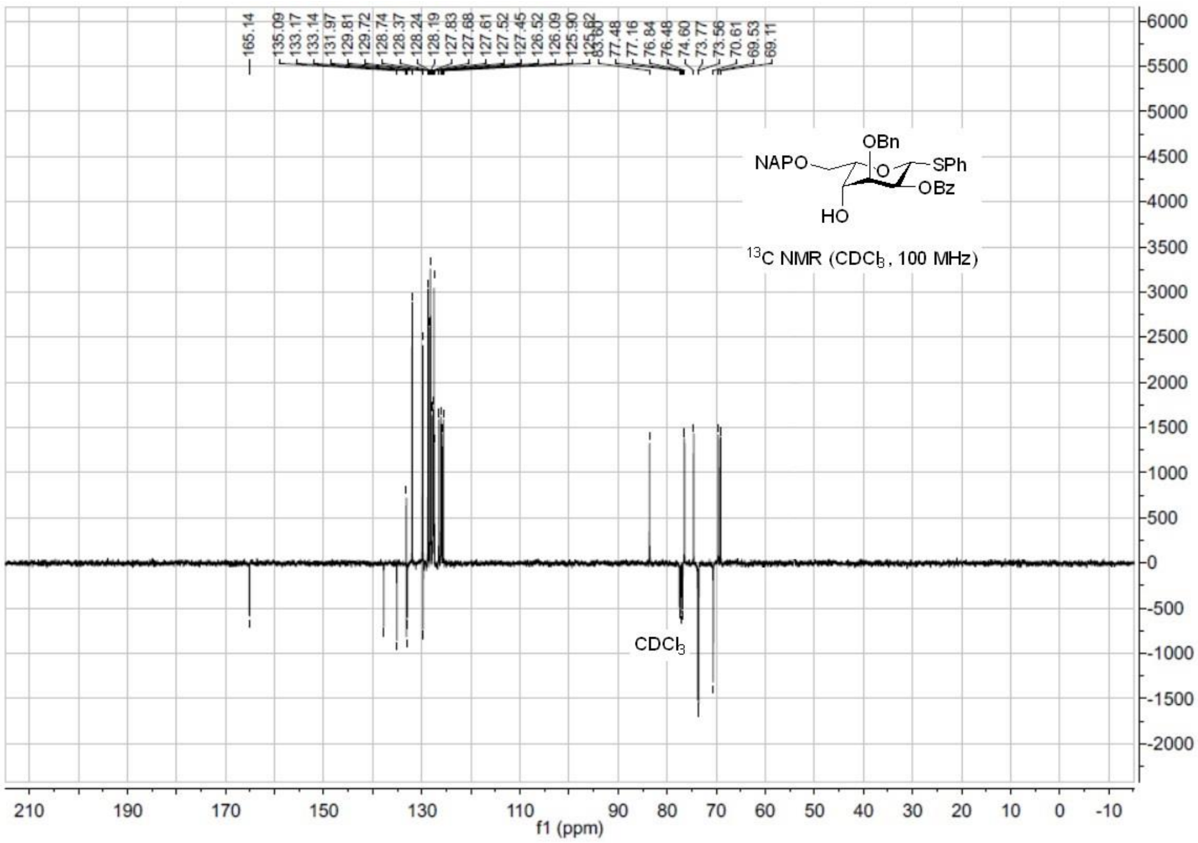

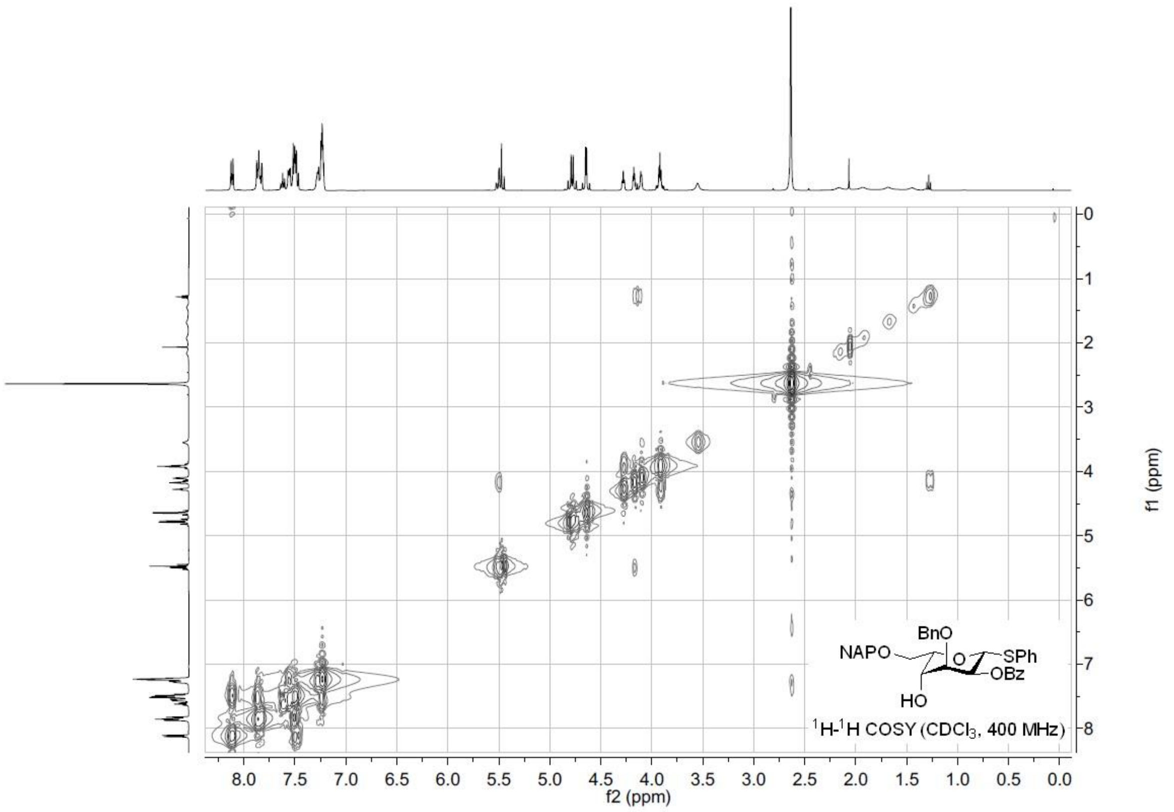
NAPO
BnO
O
SPh
OBz
HO
1H-1H COSY (CDCl3, 400 MHz)
f2 (ppm)
f1 (ppm)

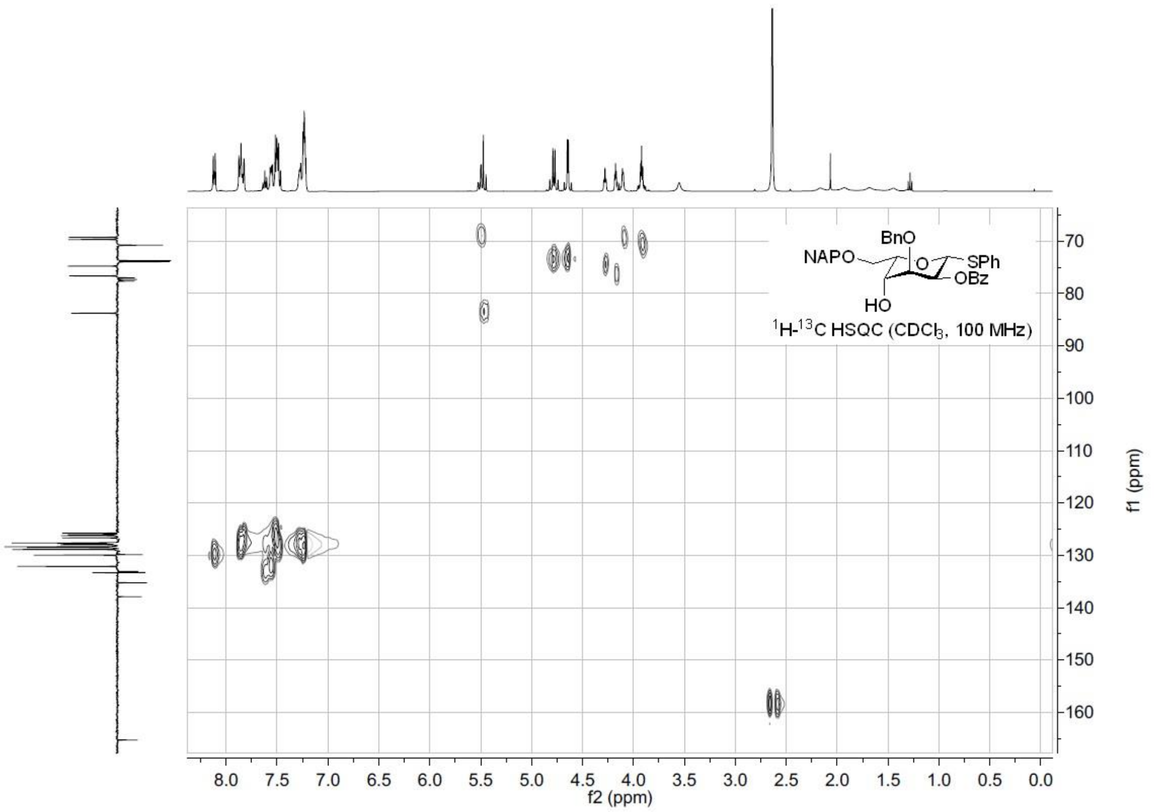
NAPO
BnO
O
SPh
OBz
HO
1H-13C HSQC (CDCl3, 100 MHz)
f2 (ppm)
f1 (ppm)

^{1}H and ^{13}C NMR spectra of compound **60**

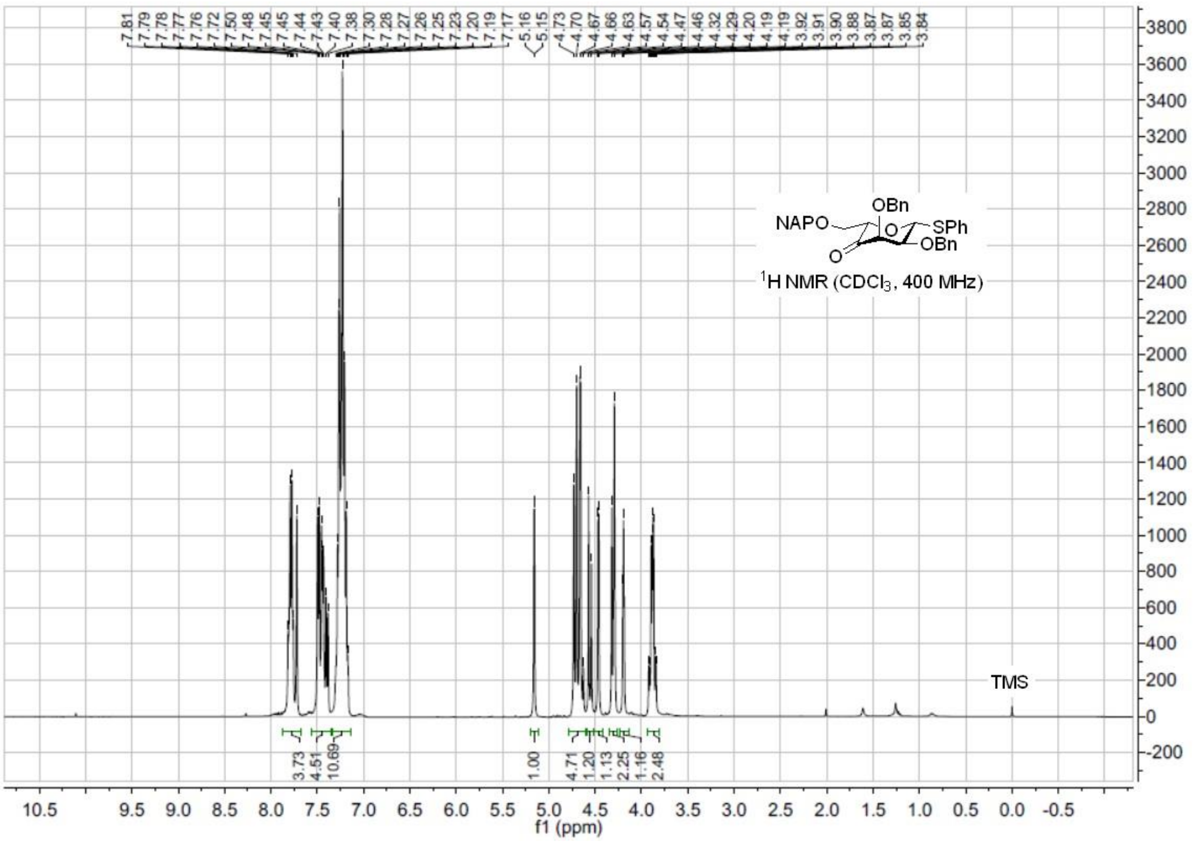

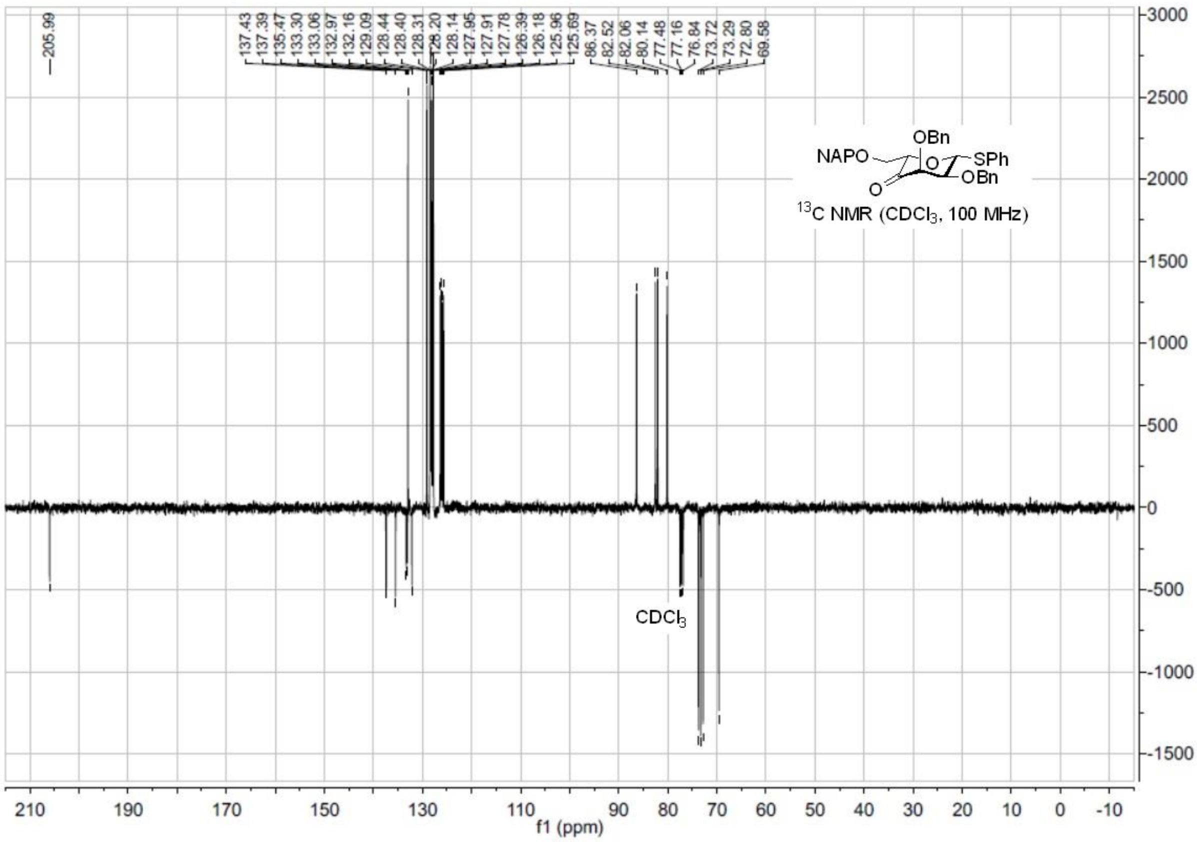

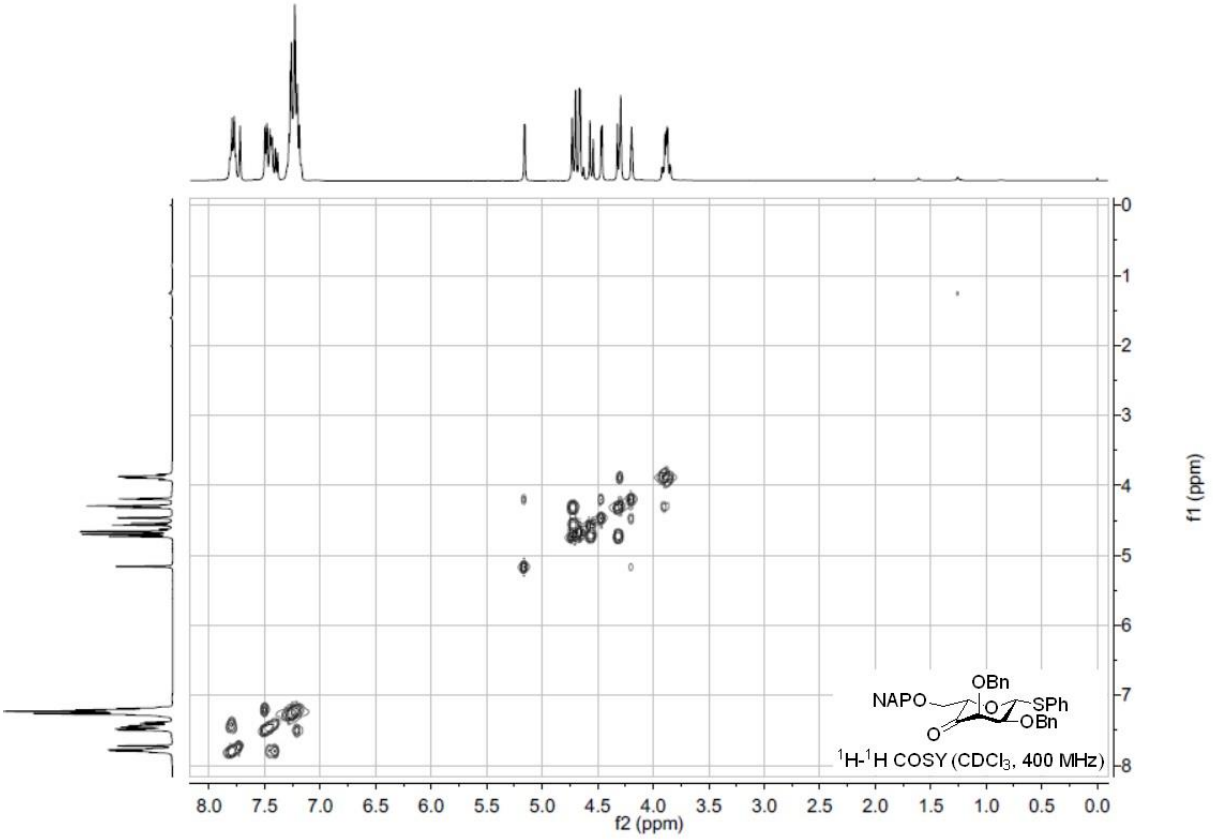

NAPO
OBn
O
SPh
OBn
O
^{1}H-^{1}H COSY (CDCl$_3$, 400 MHz)
f2 (ppm)
f1 (ppm)

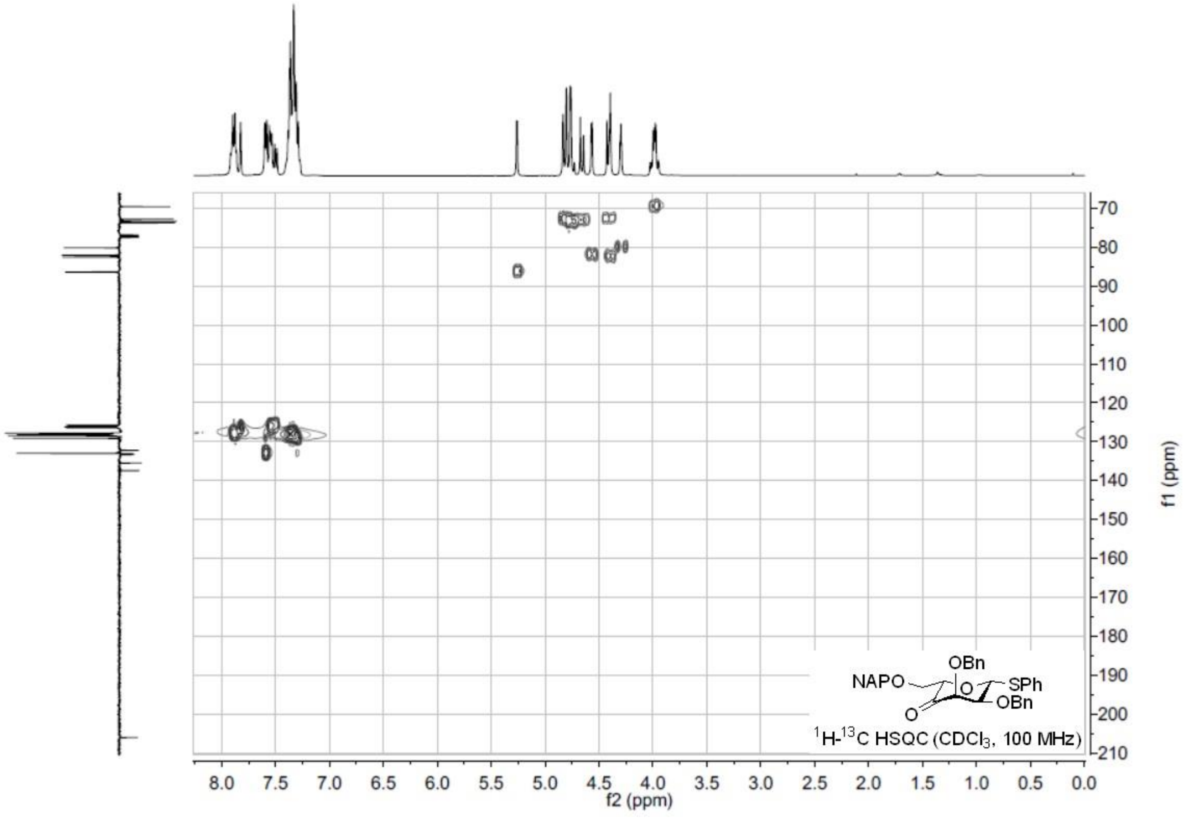

NAPO
OBn
O
SPh
OBn
O
^{1}H-^{13}C HSQC (CDCl$_3$, 100 MHz)
f2 (ppm)
f1 (ppm)

^{1}H and ^{13}C NMR spectra of compound **61**

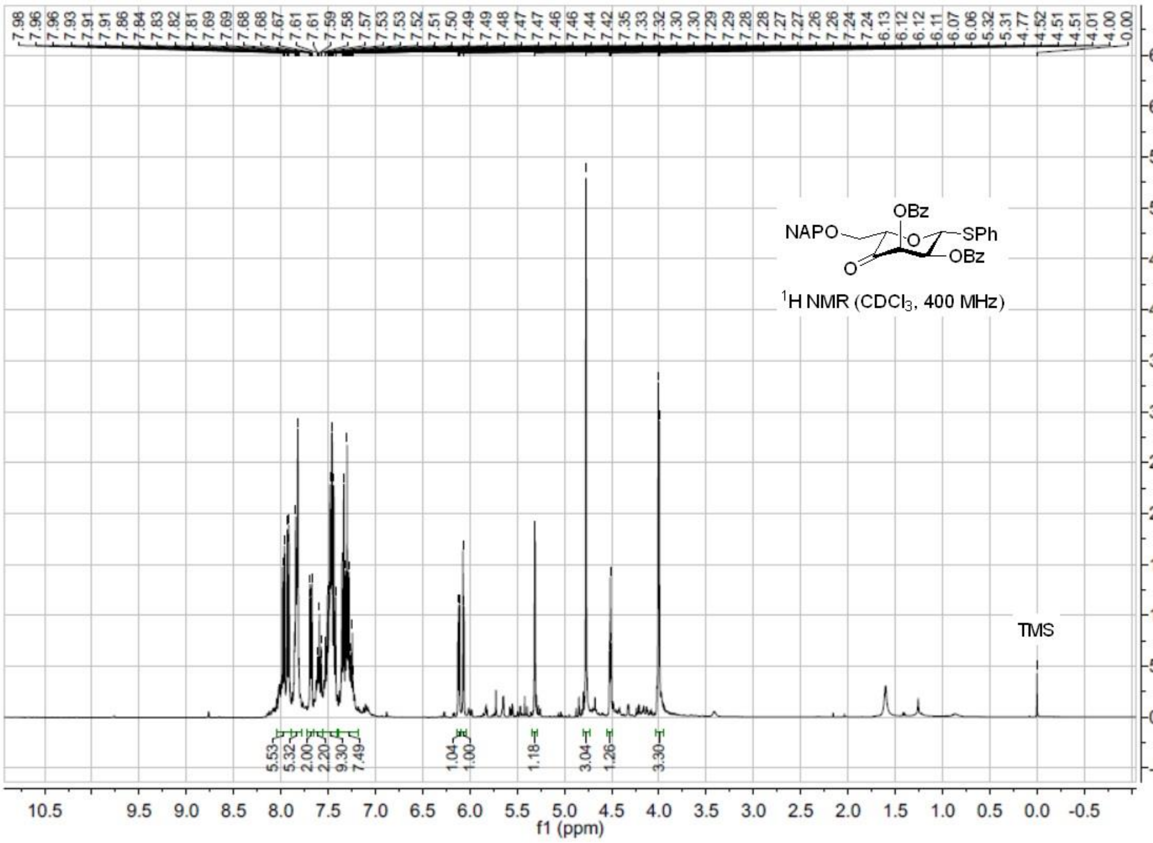

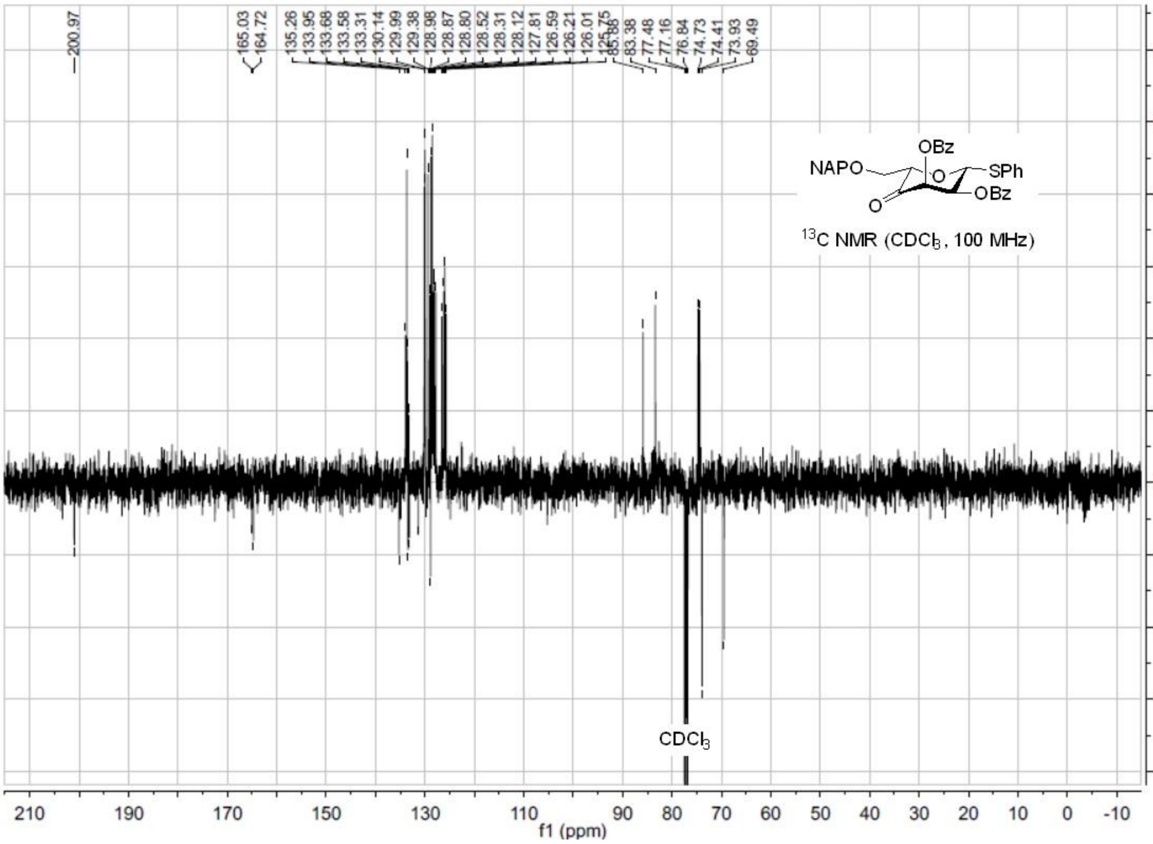

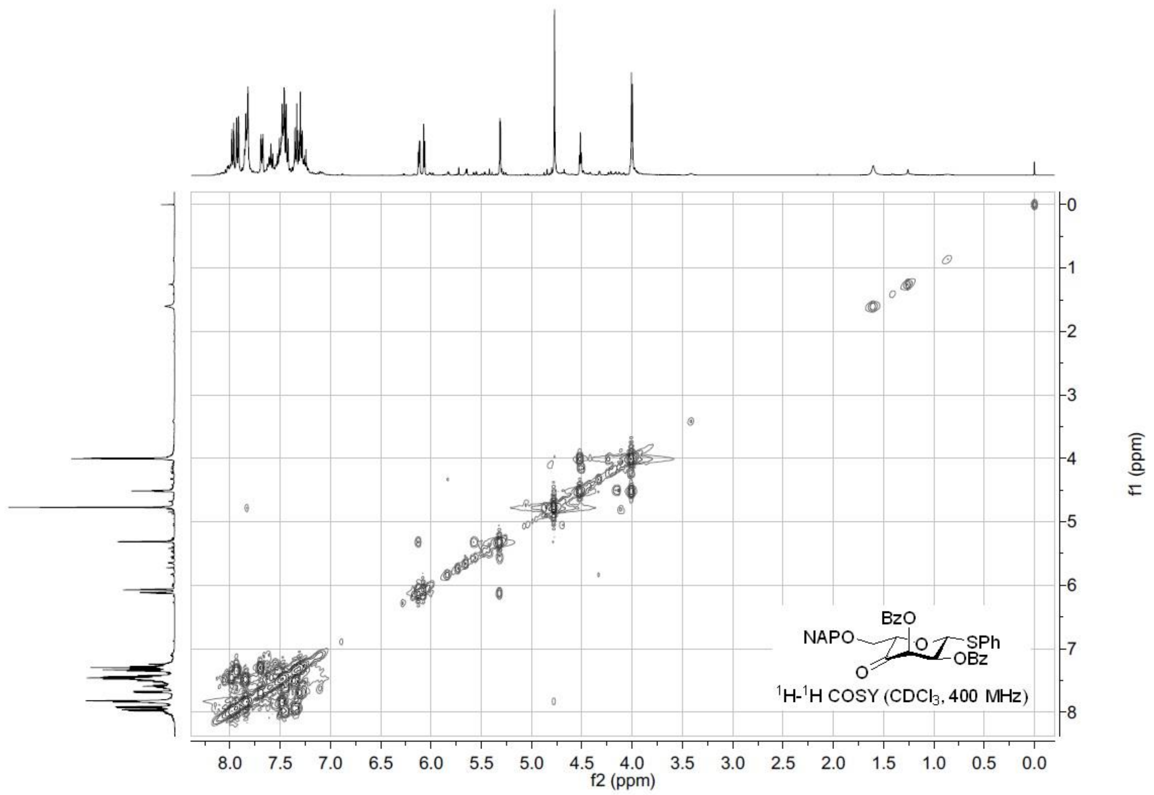
BzO
NAPO
O
SPh
OBz
O
^{1}H-^{1}H COSY (CDCl$_3$, 400 MHz)
f1 (ppm)
f2 (ppm)

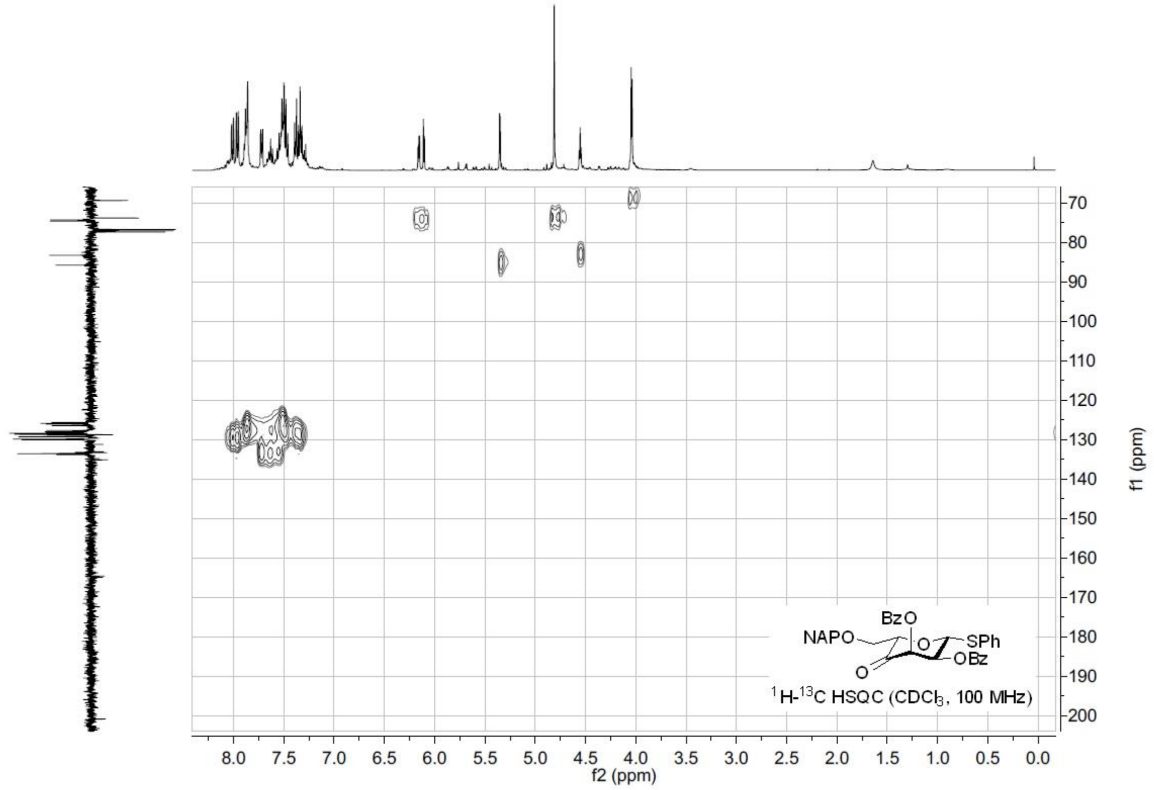
BzO
NAPO
O
SPh
OBz
O
^{1}H-^{13}C HSQC (CDCl$_3$, 100 MHz)
f1 (ppm)
f2 (ppm)

^{1}H and ^{13}C NMR spectra of compound **62**

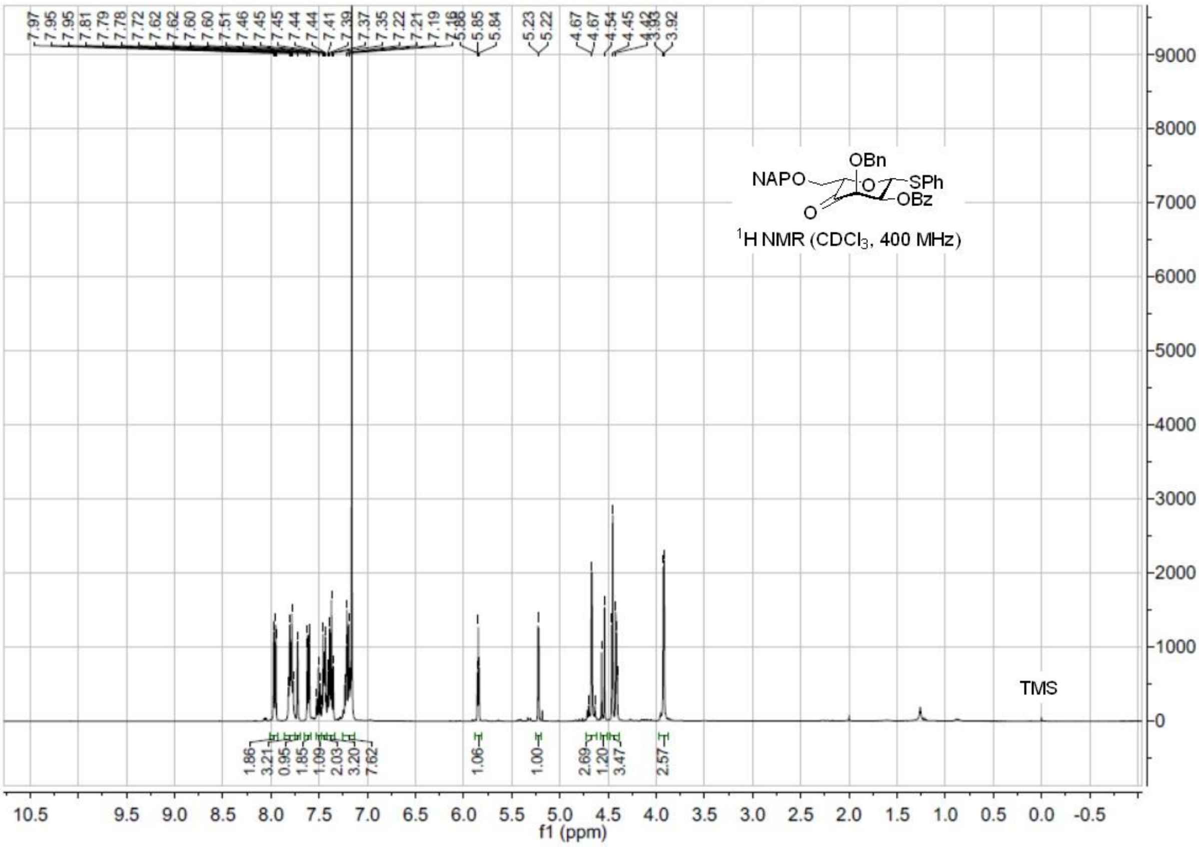

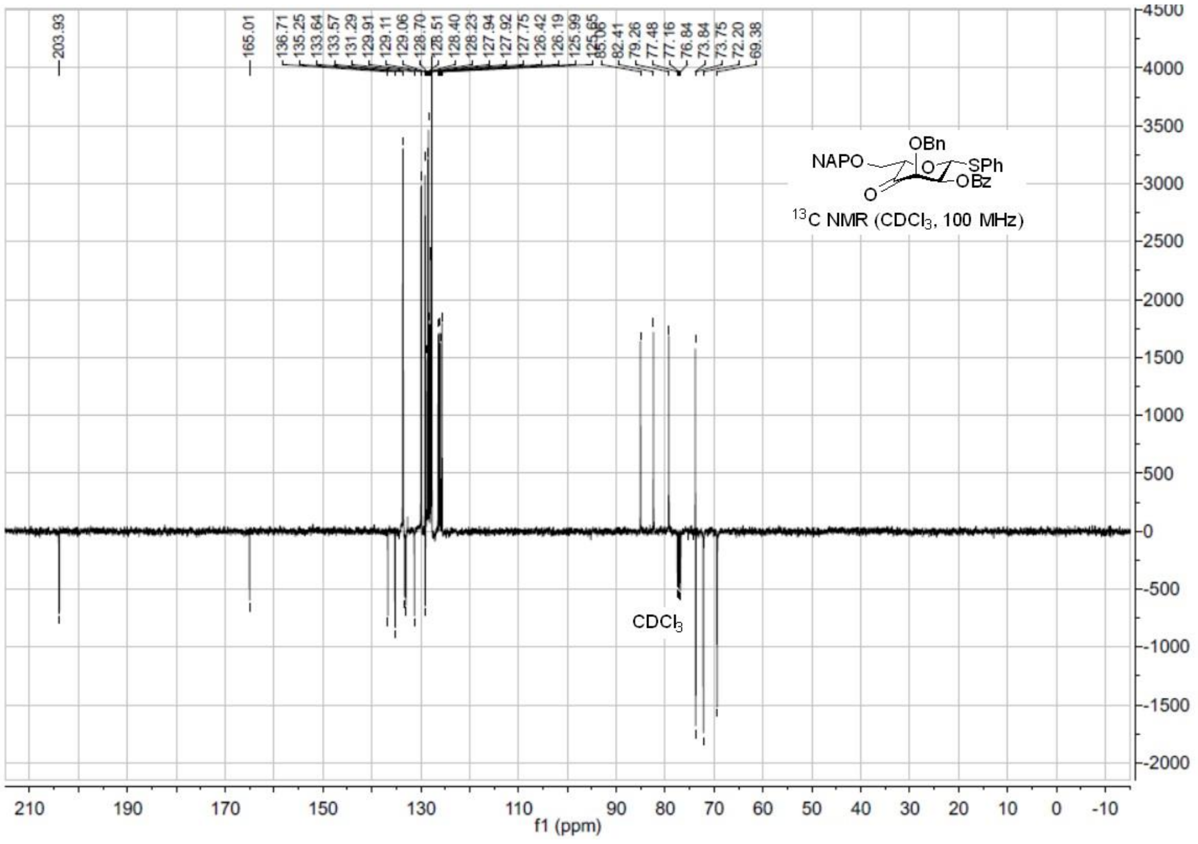

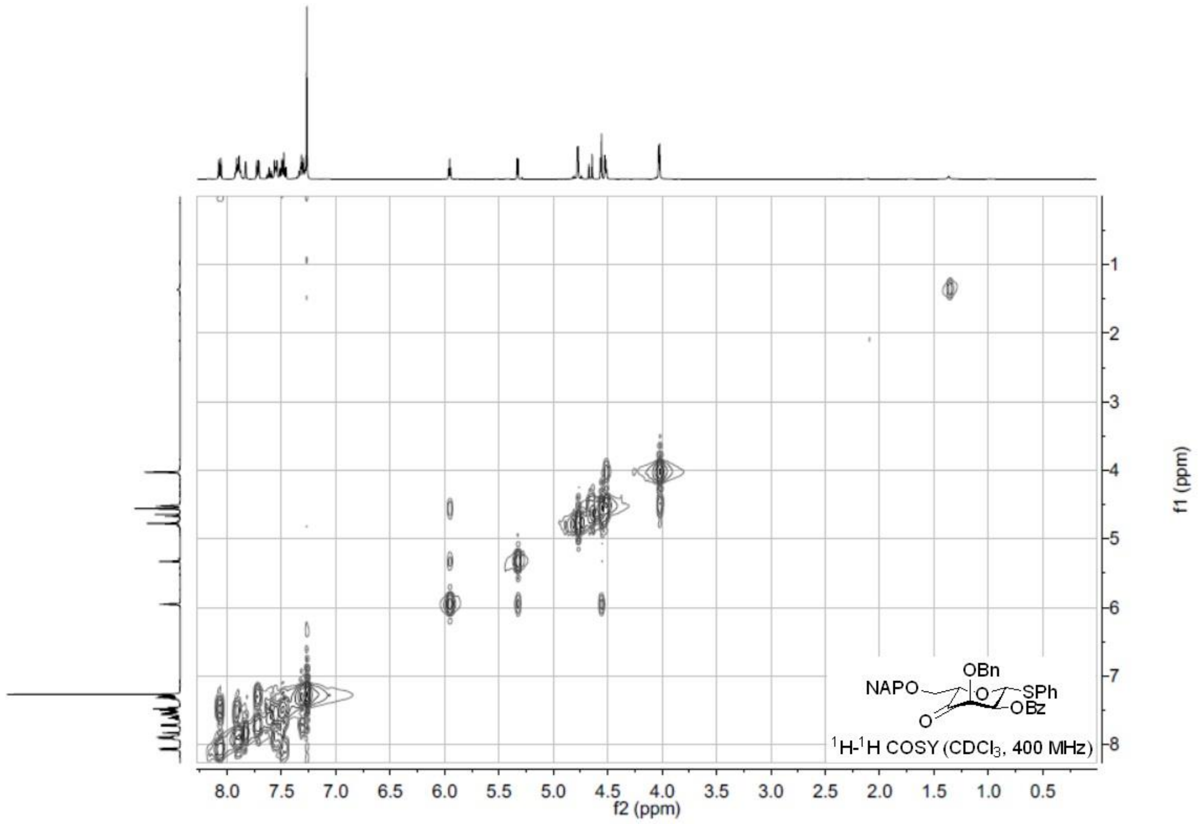
OBn
NAPO
O
SPh
OBz
O
1H-1H COSY (CDCl3, 400 MHz)
f2 (ppm)
f1 (ppm)

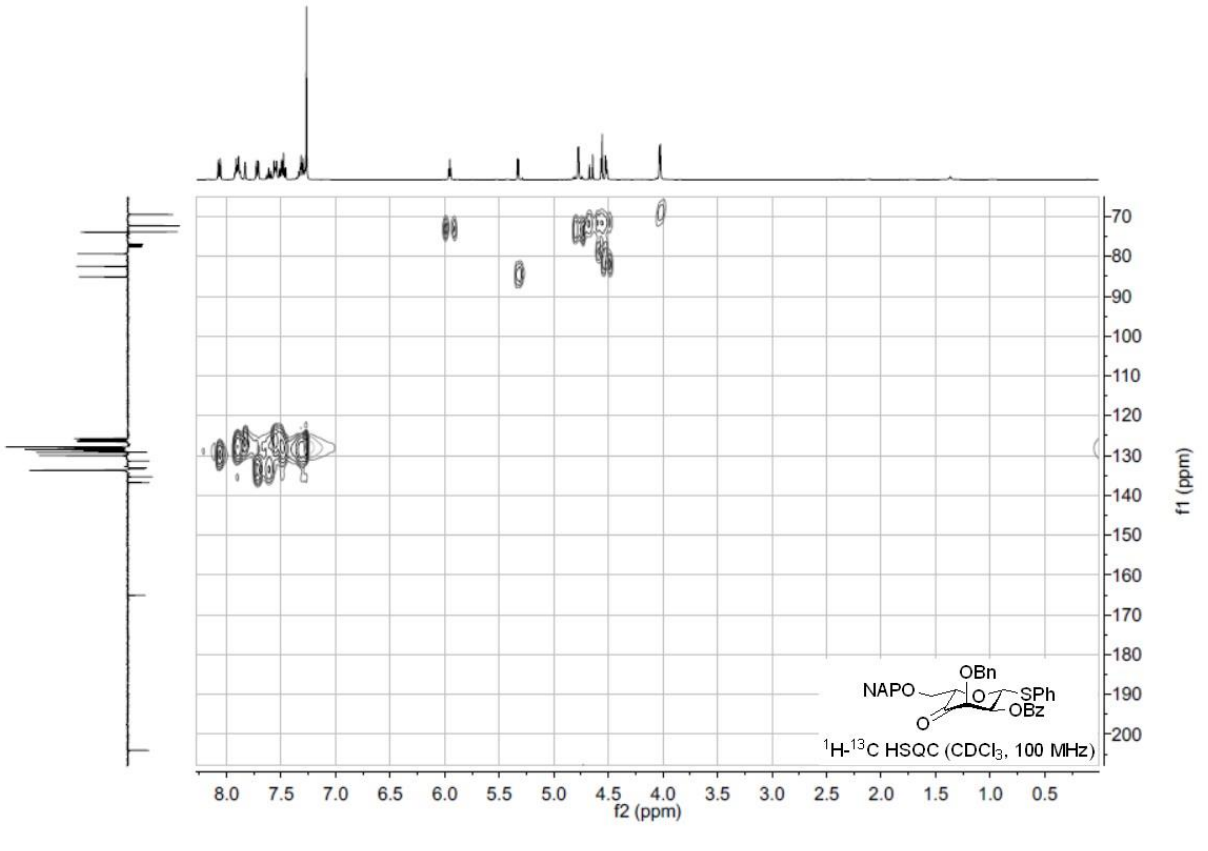
OBn
NAPO
O
SPh
OBz
O
1H-13C HSQC (CDCl3, 100 MHz)
f2 (ppm)
f1 (ppm)

^{1}H and ^{13}C NMR spectra of compound **63**

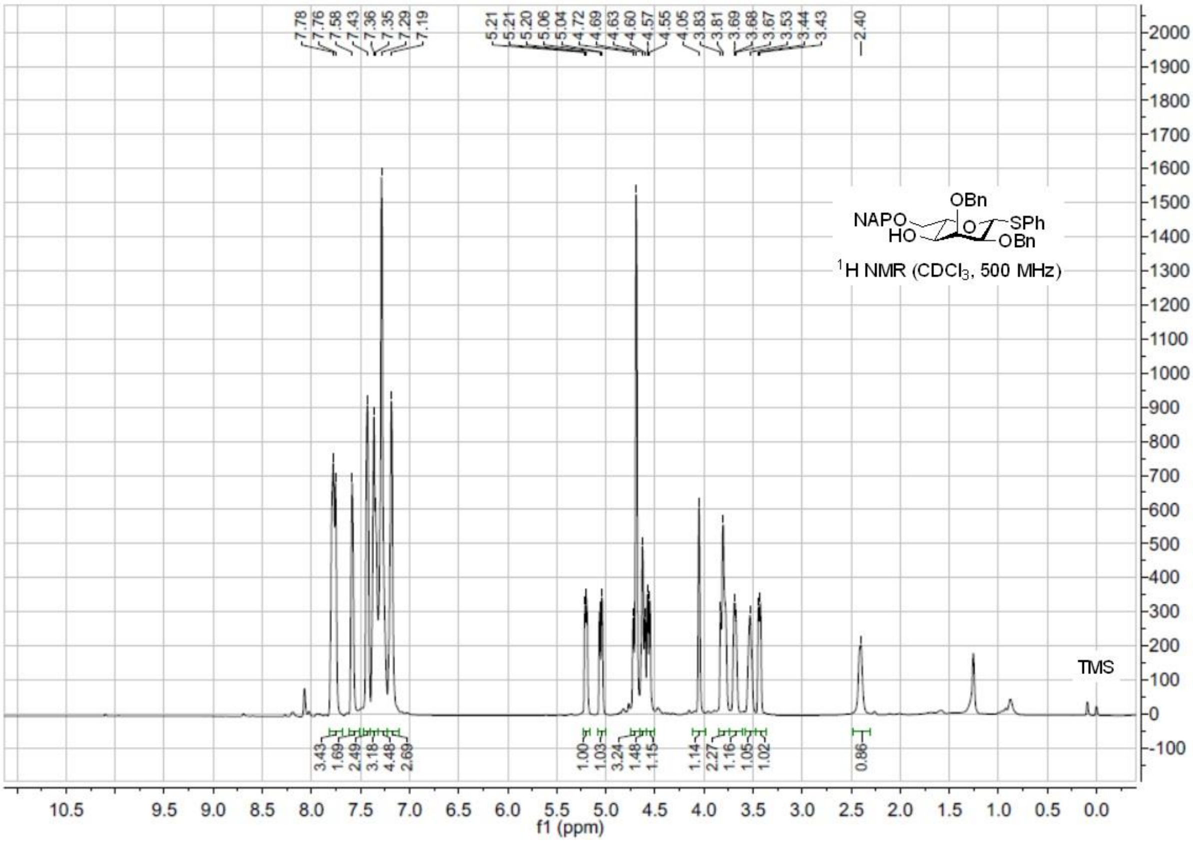

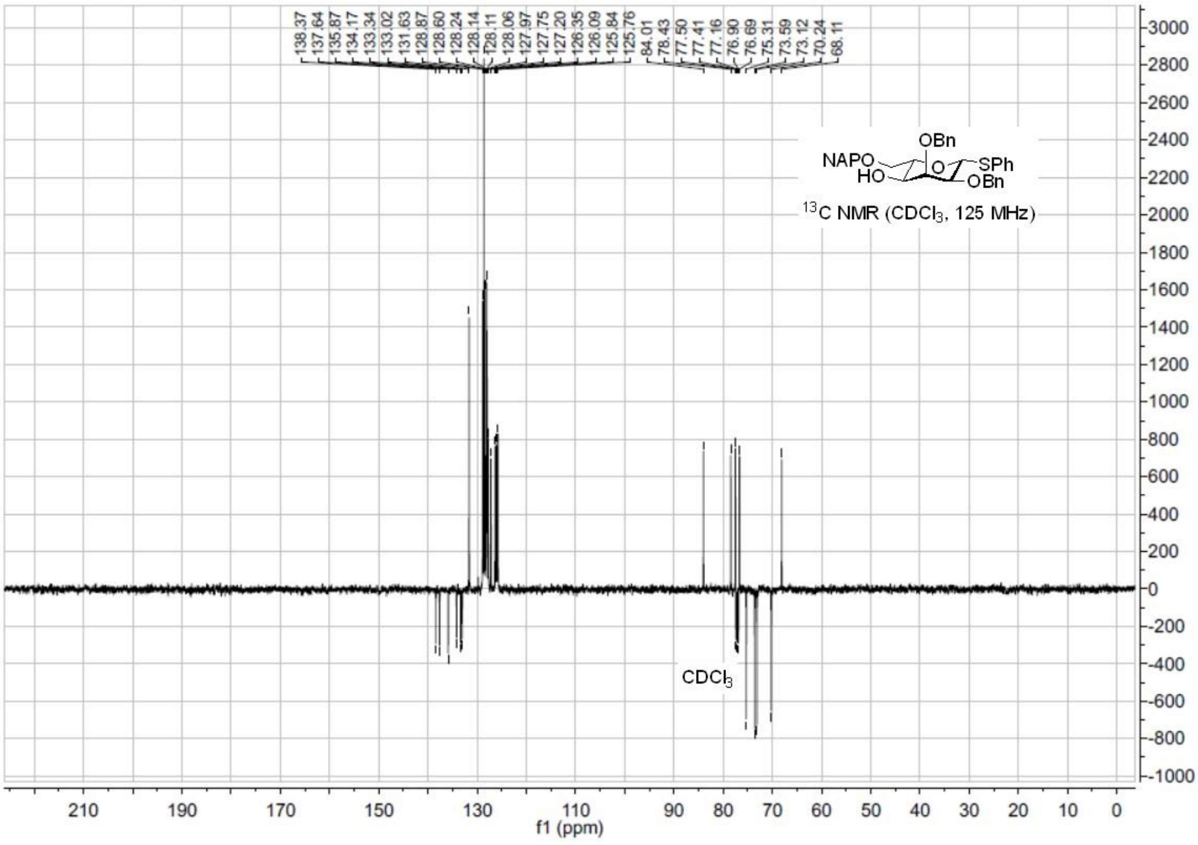

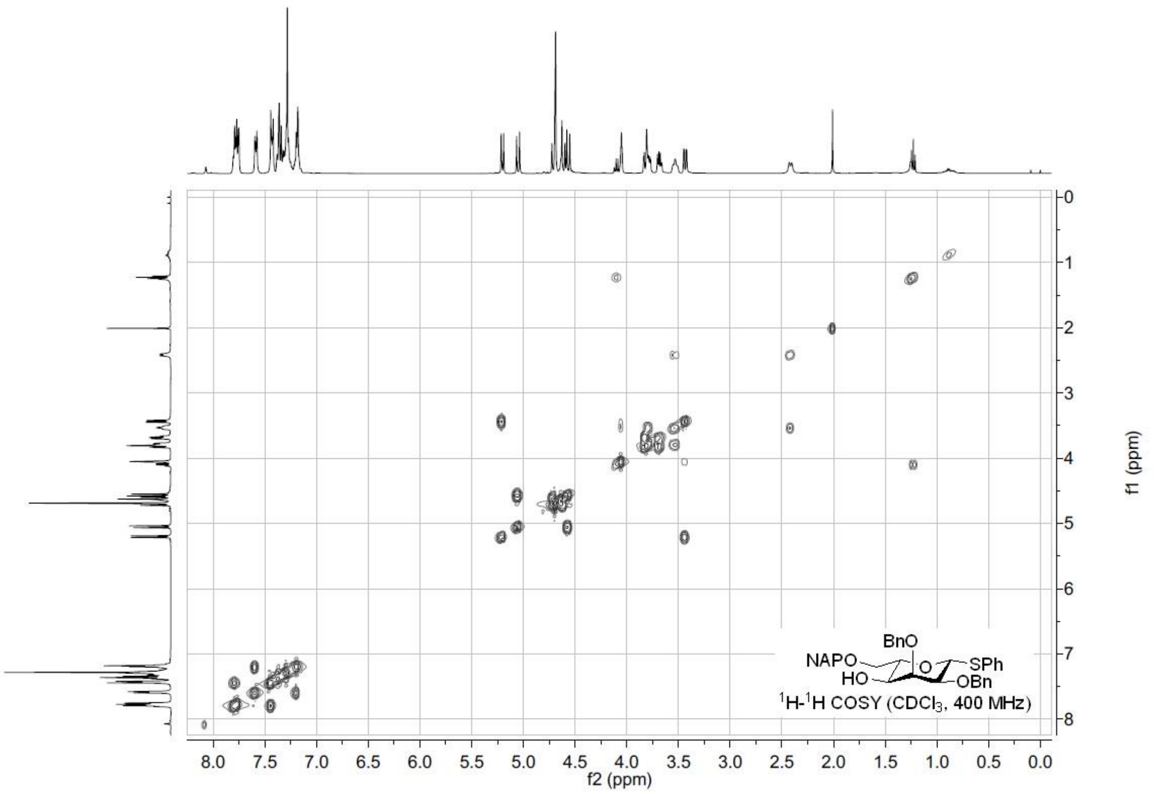
BnO
NAPO
HO
O
SPh
OBn
^{1}H-^{1}H COSY (CDCl$_3$, 400 MHz)
f2 (ppm)
f1 (ppm)

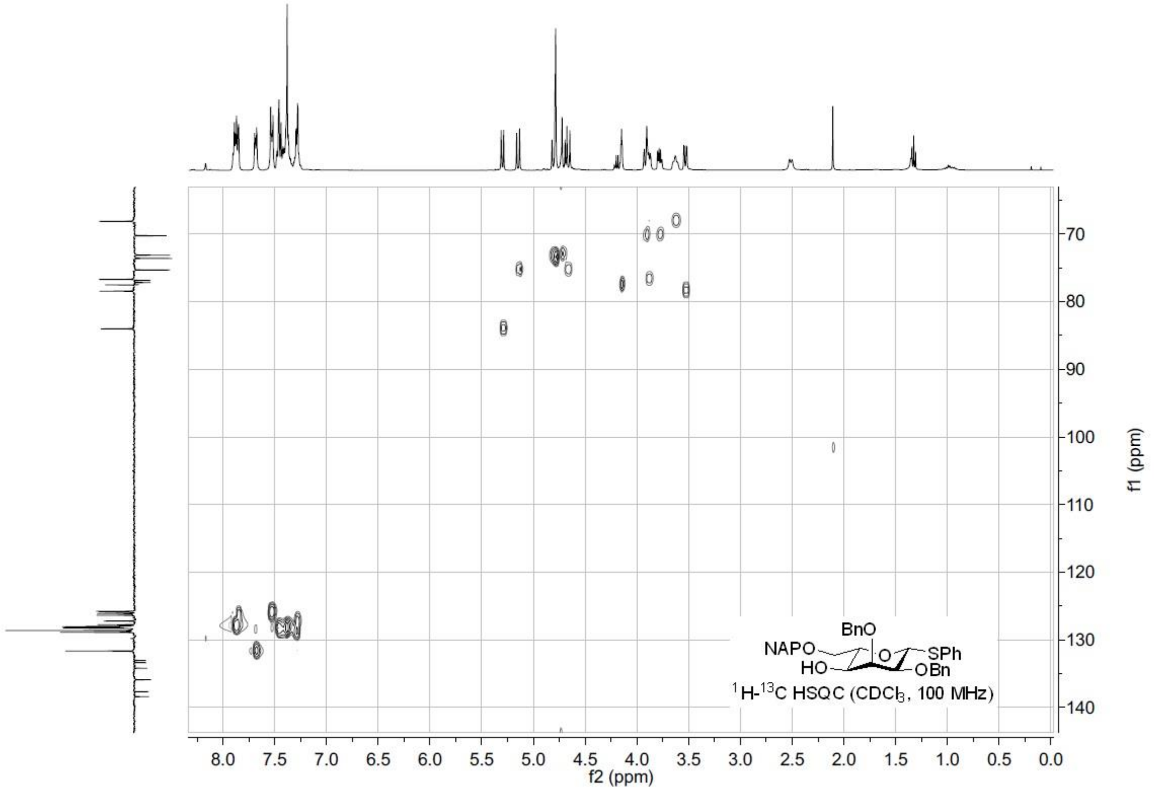
BnO
NAPO
HO
O
SPh
OBn
^{1}H-^{13}C HSQC (CDCl$_3$, 100 MHz)
f2 (ppm)
f1 (ppm)

^{1}H and ^{13}C NMR spectra of the mixture of compound **64** and **58**

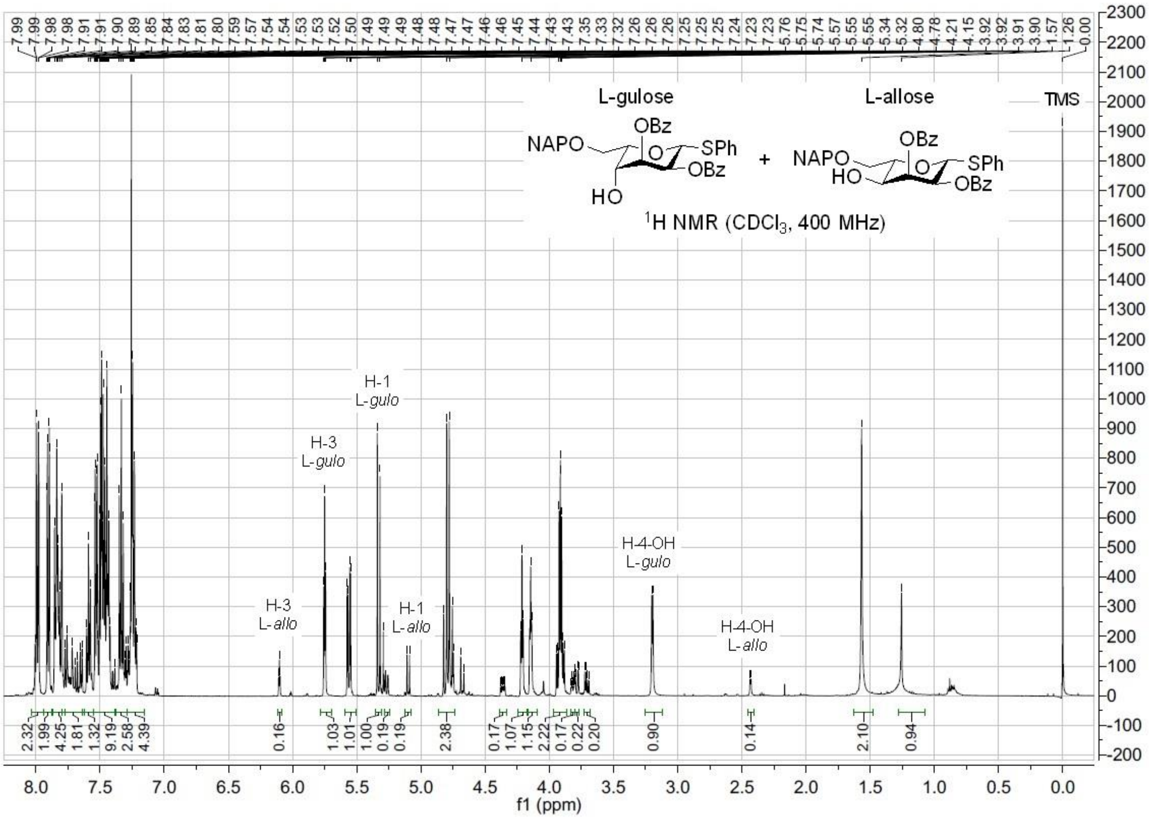

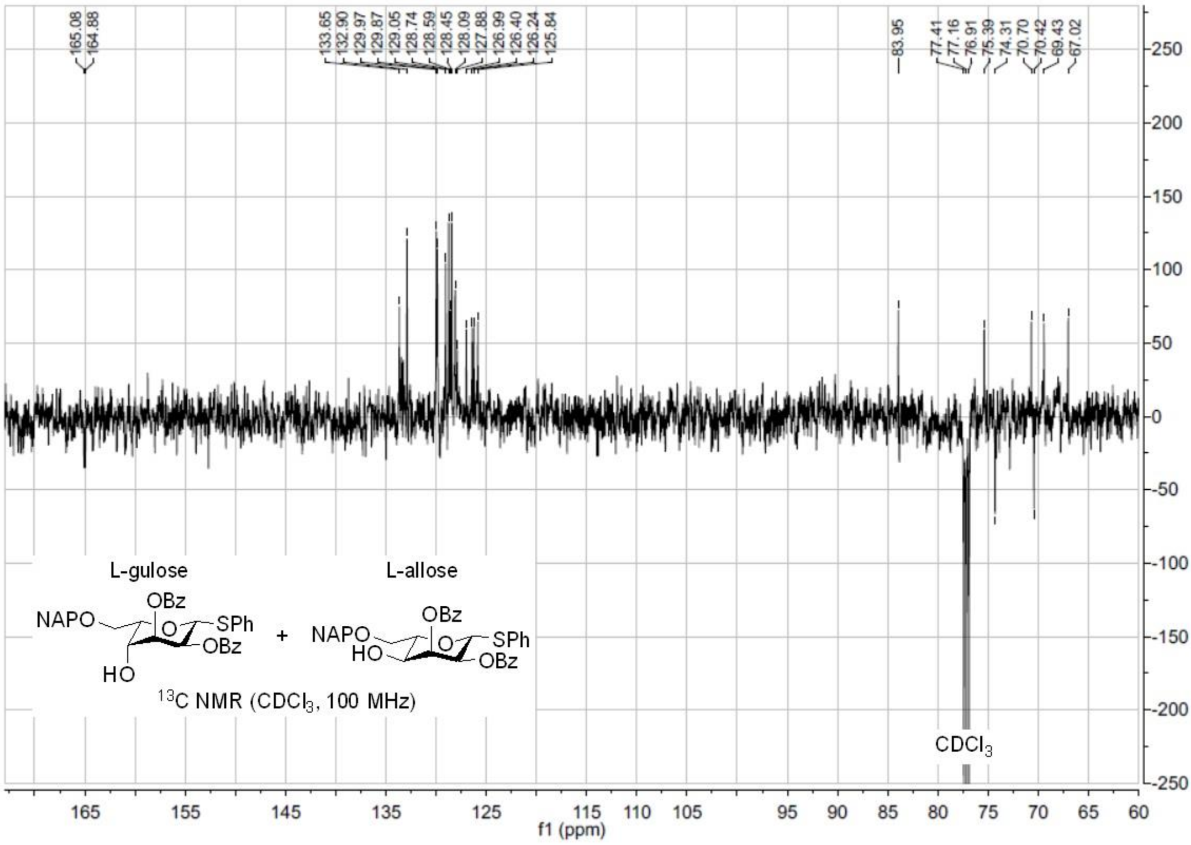

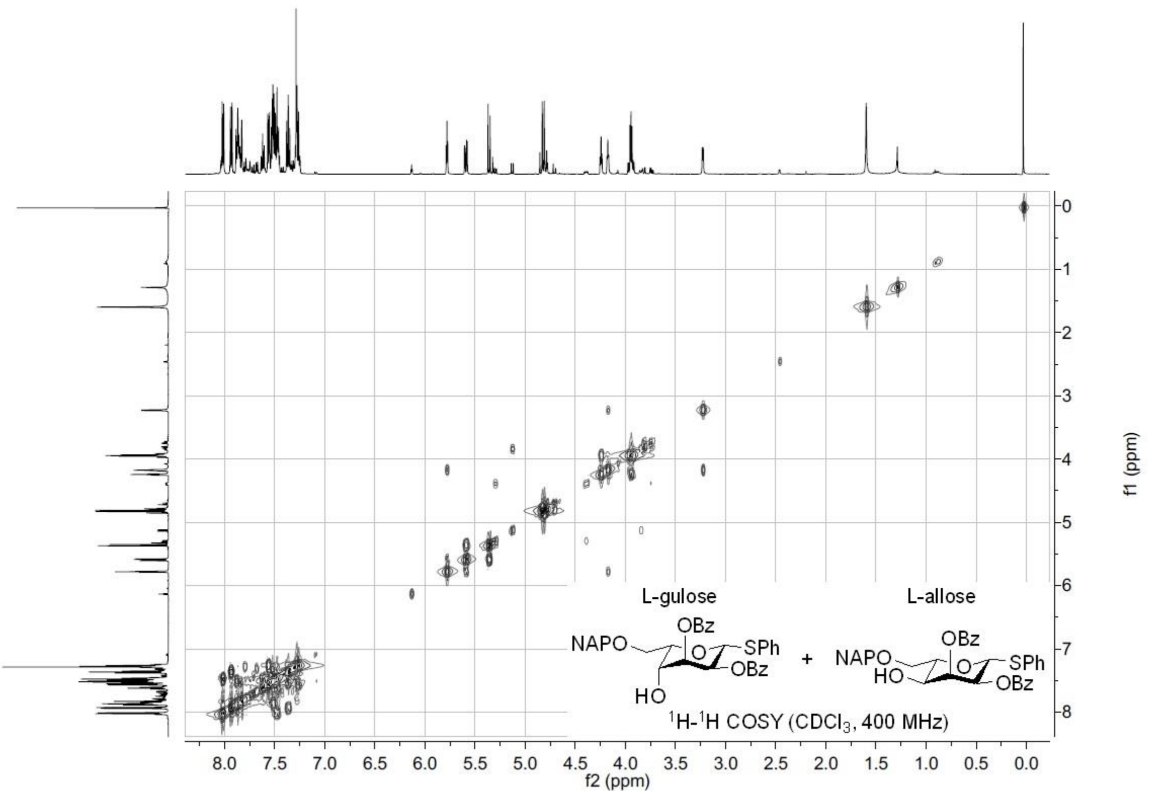

L-gulose
L-allose
NAPO
OBz
SPh
OBz
HO
+
¹H-¹H COSY (CDCl₃, 400 MHz)
f2 (ppm)
f1 (ppm)

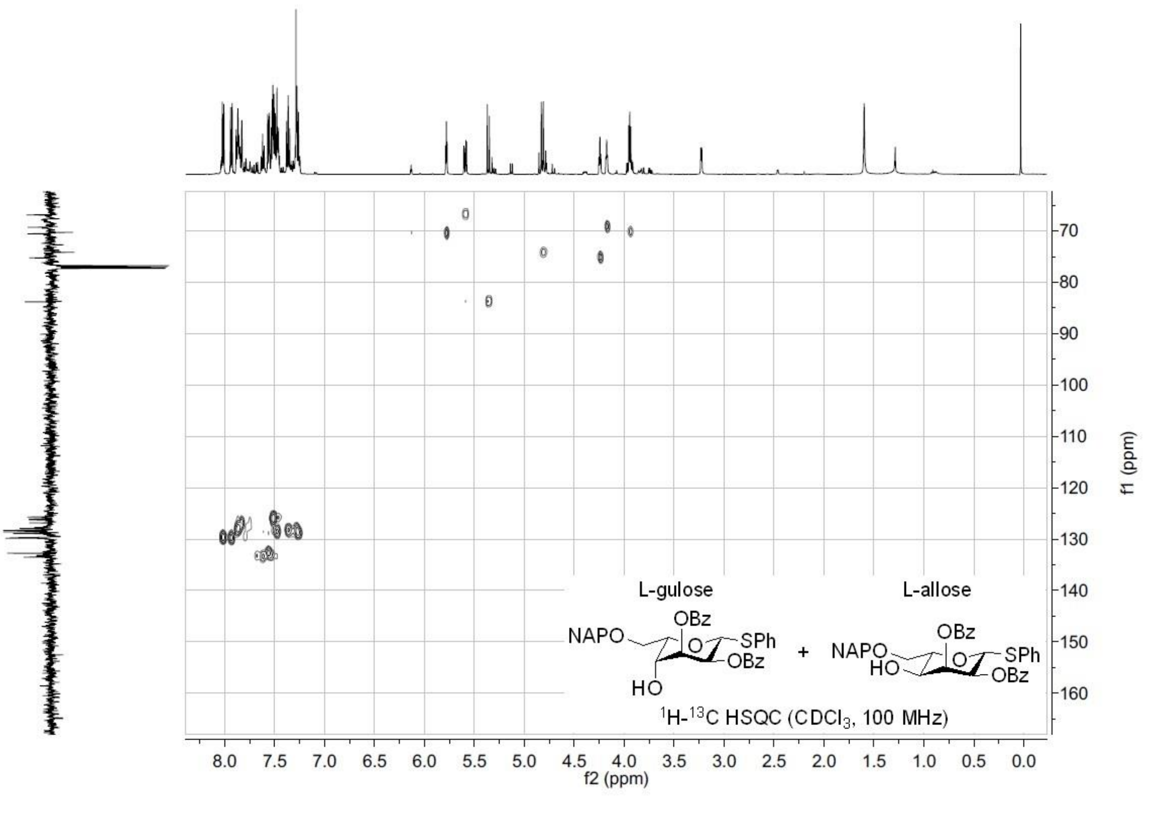

L-gulose
L-allose
NAPO
OBz
SPh
OBz
HO
+
¹H-¹³C HSQC (CDCl₃, 100 MHz)
f2 (ppm)
f1 (ppm)

^{1}H and ^{13}C NMR spectra of compound **65**

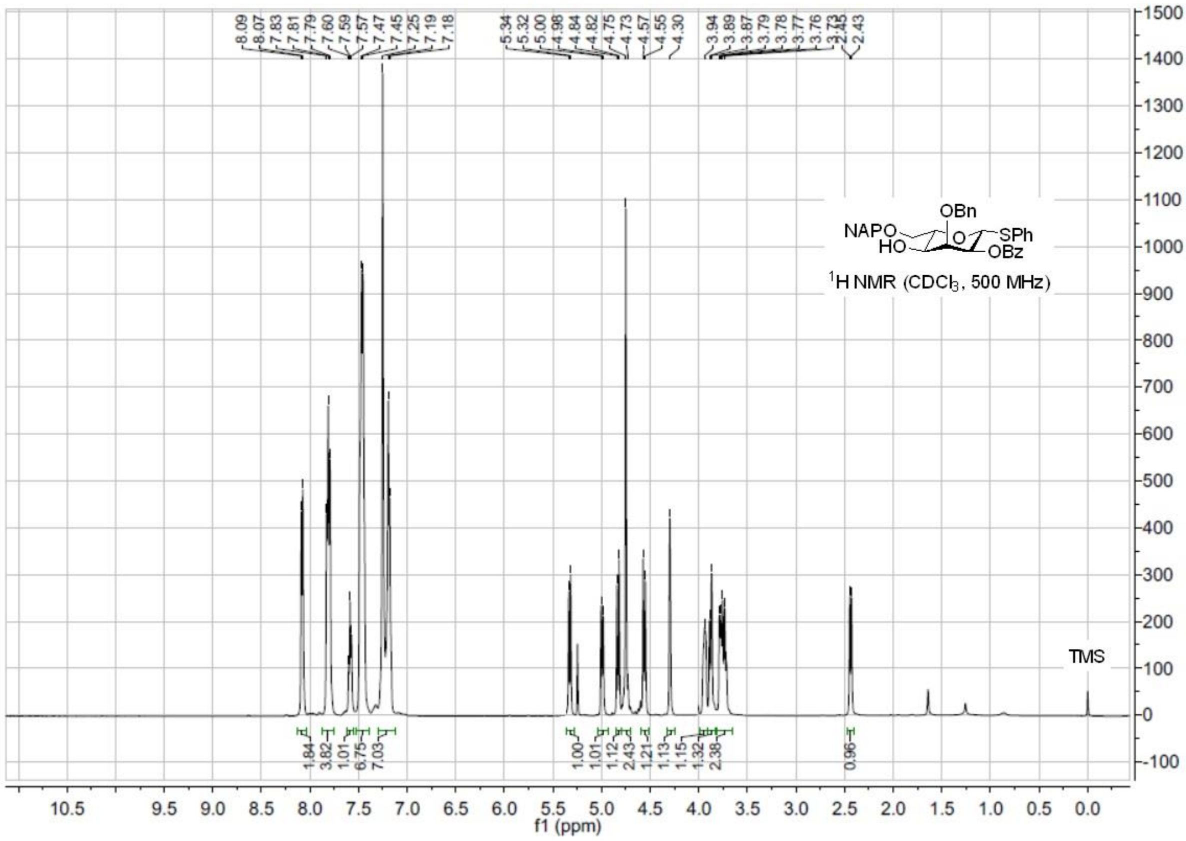

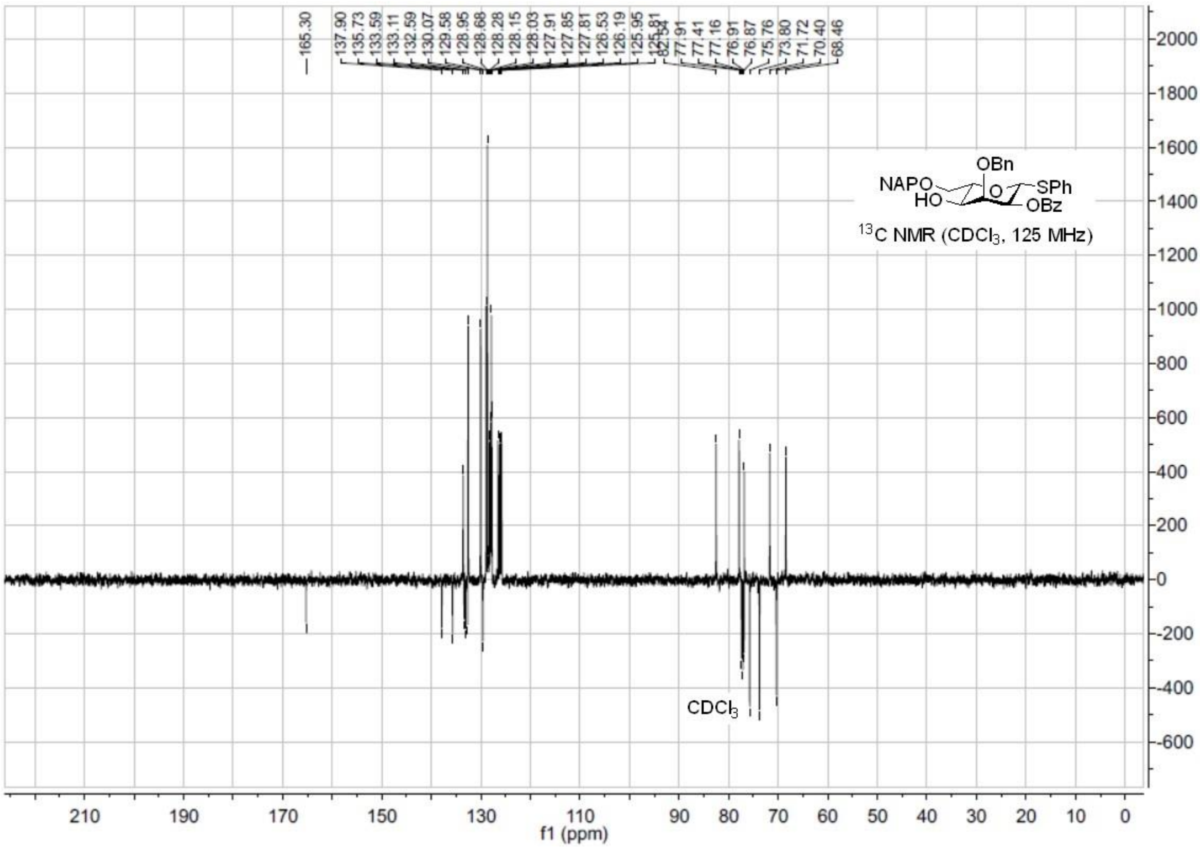

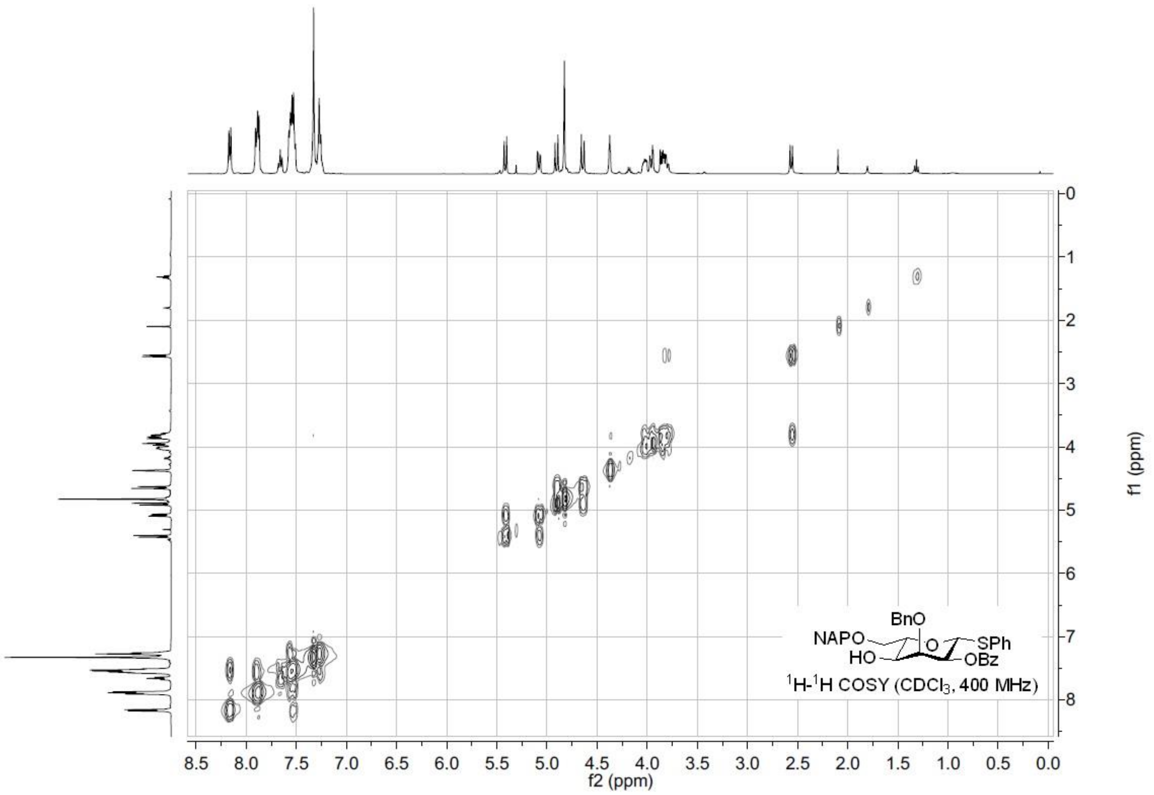
BnO
NAPO
HO
O
SPh
OBz
^{1}H-^{1}H COSY (CDCl$_3$, 400 MHz)
f1 (ppm)
f2 (ppm)

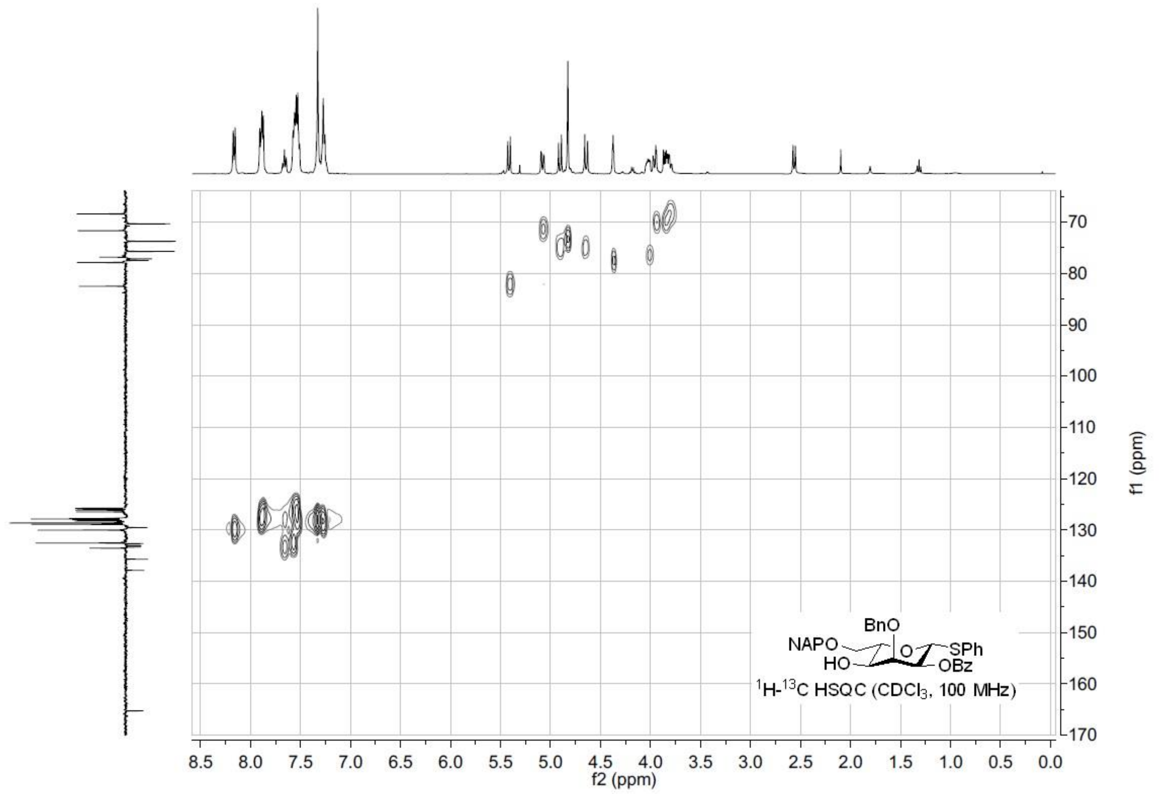
BnO
NAPO
HO
O
SPh
OBz
^{1}H-^{13}C HSQC (CDCl$_3$, 100 MHz)
f1 (ppm)
f2 (ppm)

^{1}H and ^{13}C NMR spectra of compound **66**

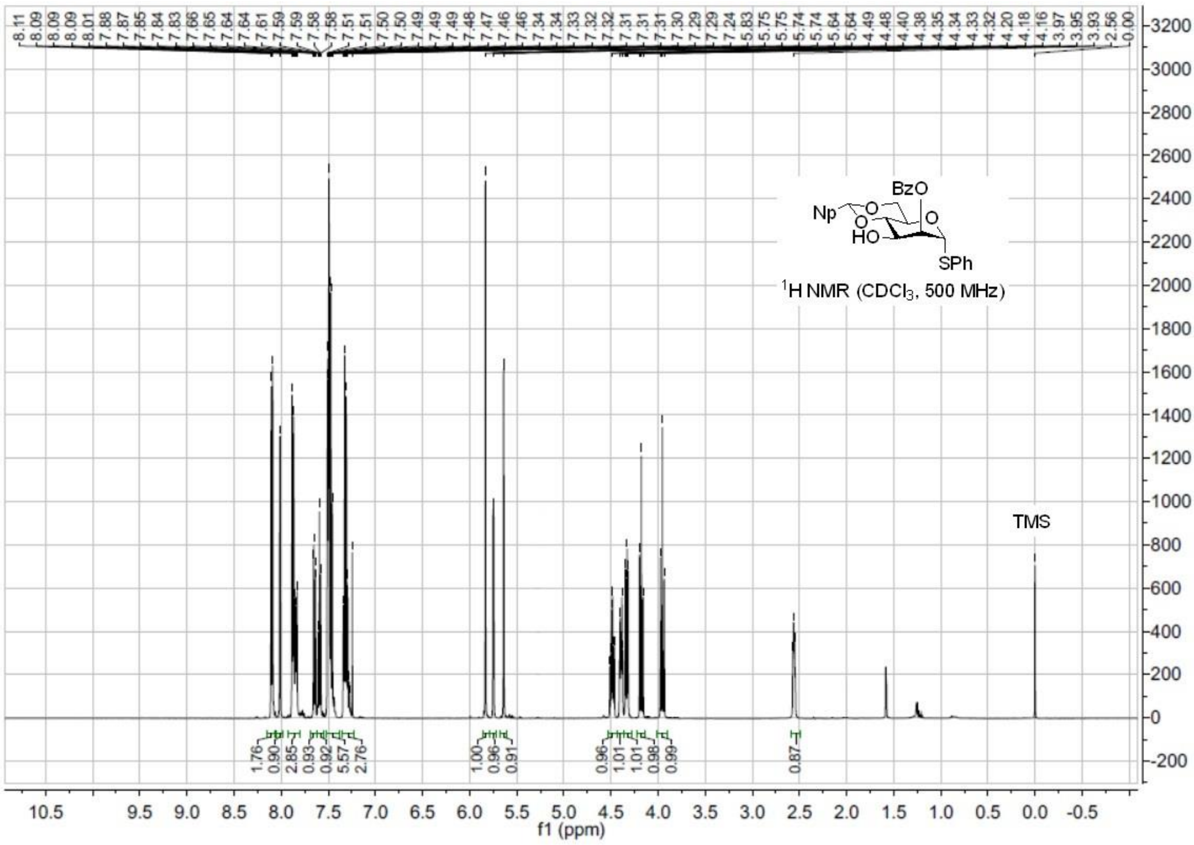

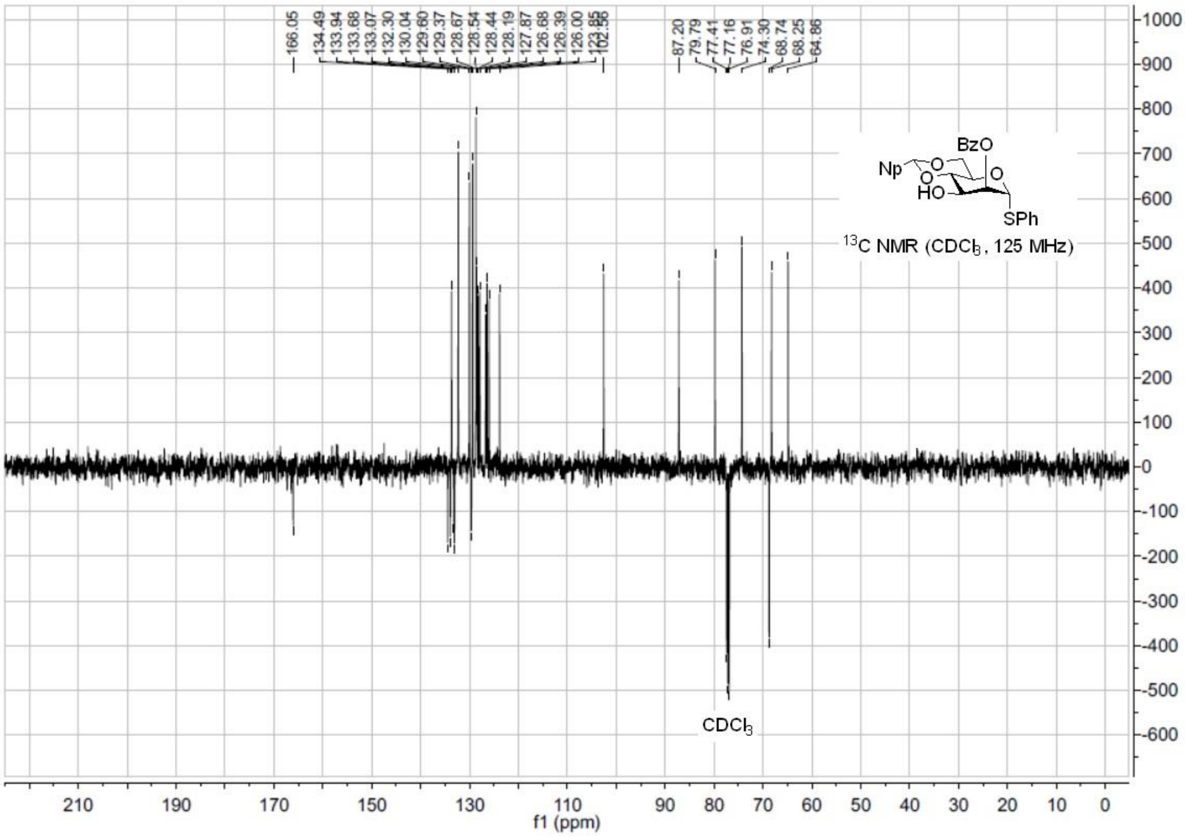

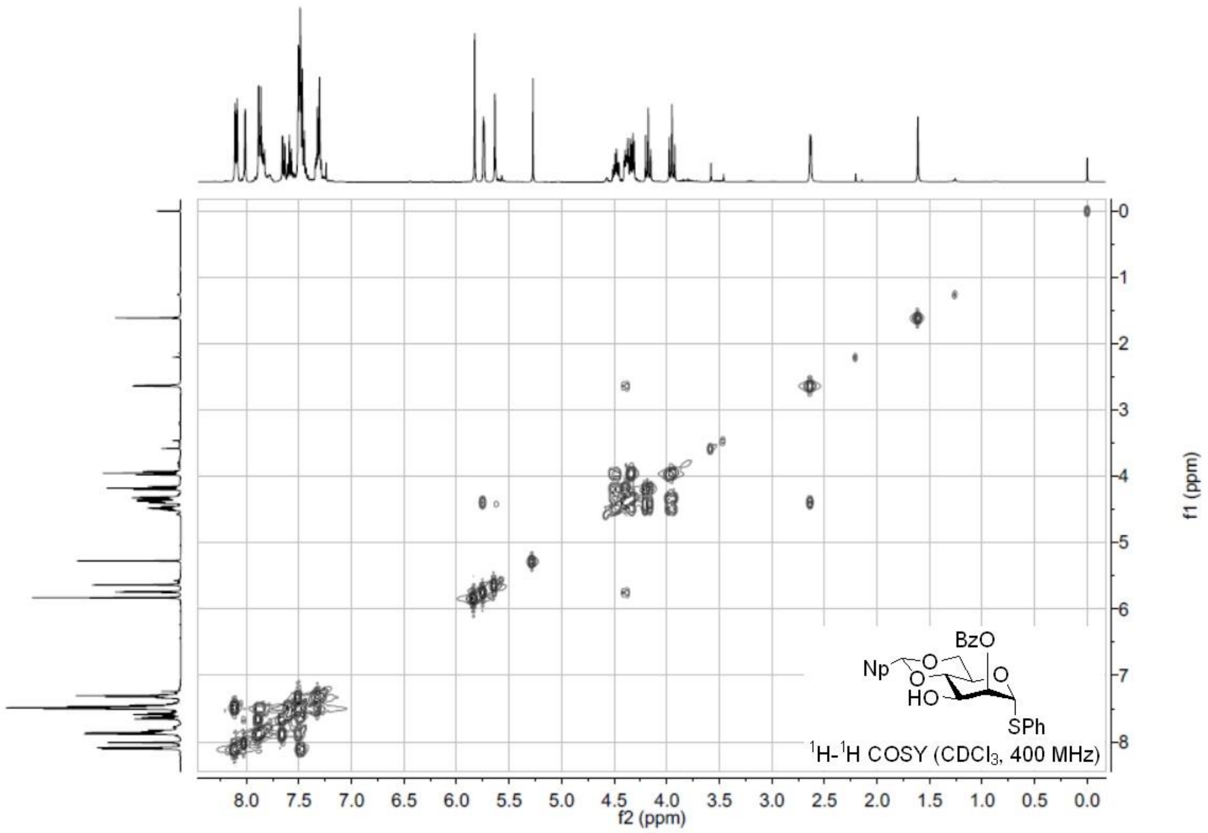
BzO
Np
O
O
O
HO
SPh
^{1}H-^{1}H COSY (CDCl$_3$, 400 MHz)
f2 (ppm)
f1 (ppm)

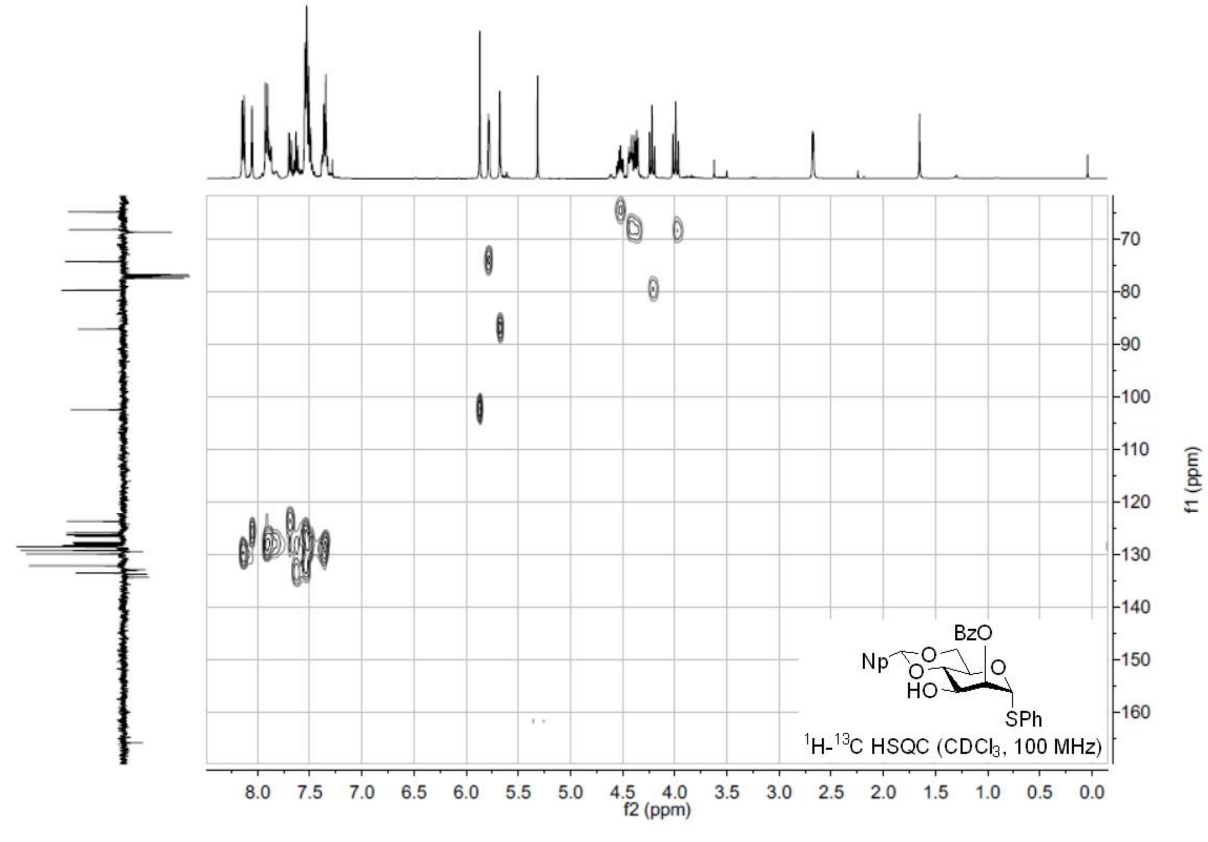
BzO
Np
O
O
O
HO
SPh
^{1}H-^{13}C HSQC (CDCl$_3$, 100 MHz)
f2 (ppm)
f1 (ppm)

^{1}H and ^{13}C NMR spectra of **3-ulose**

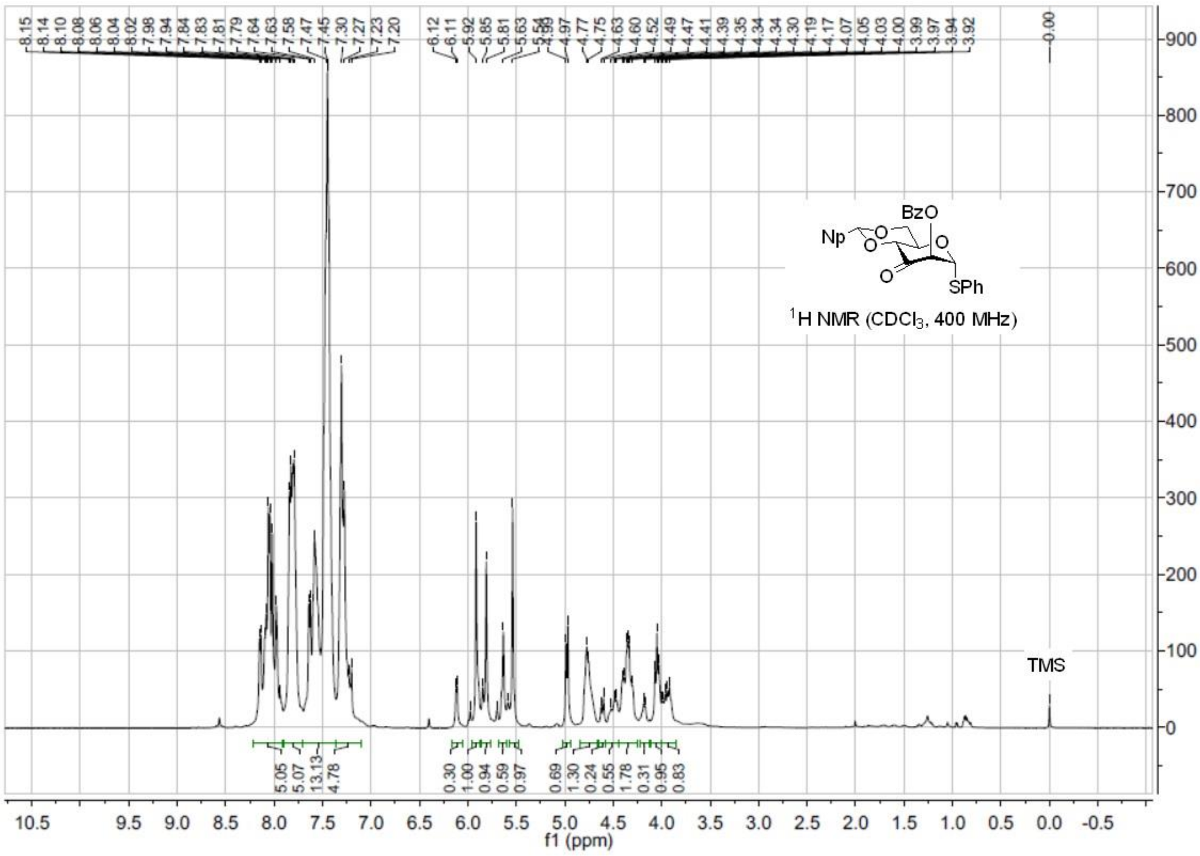

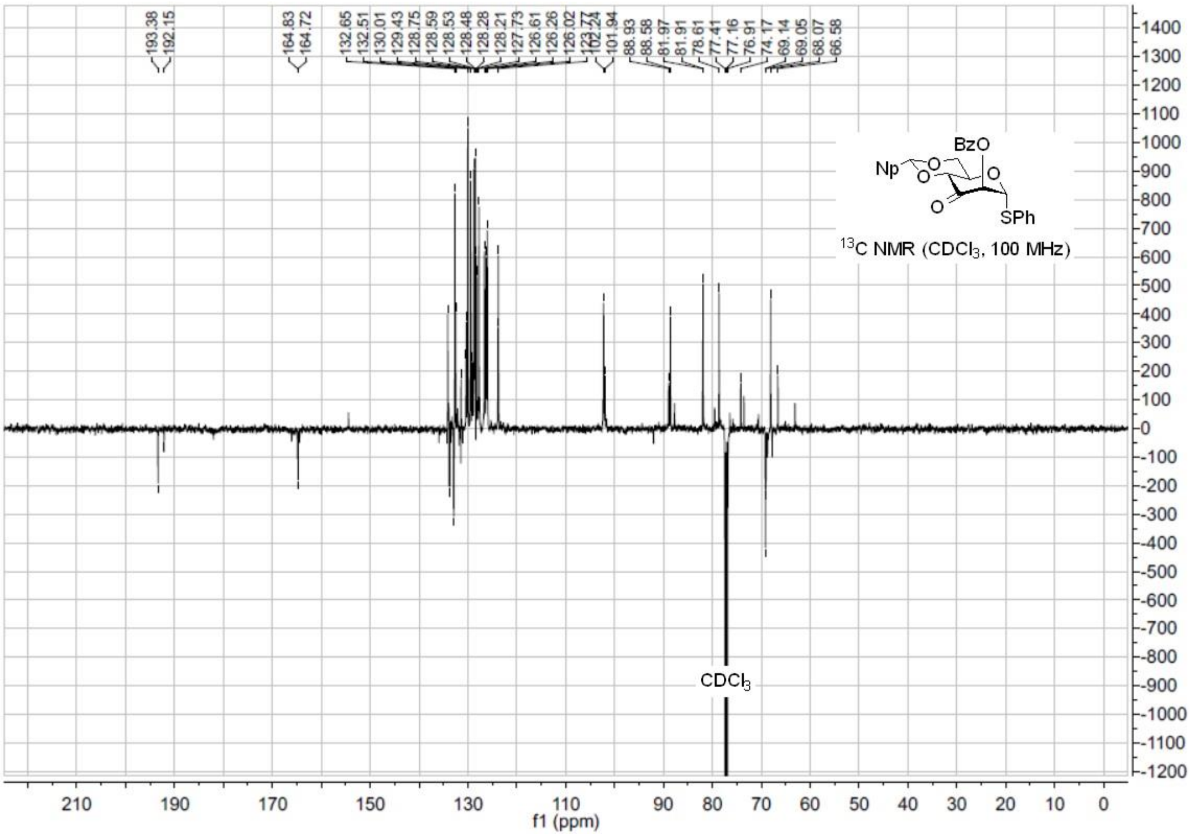

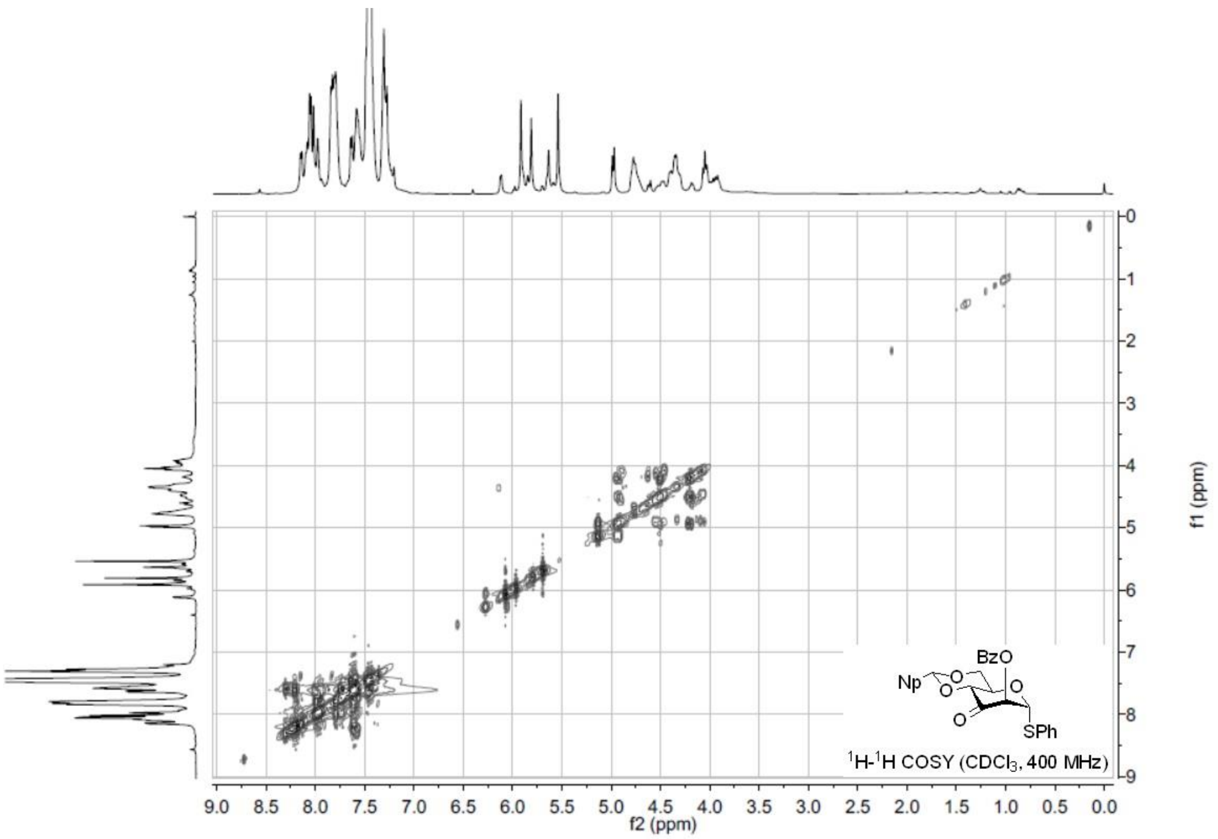

BzO
Np
O
SPh
^{1}H-^{1}H COSY (CDCl$_3$, 400 MHz)
f2 (ppm)
f1 (ppm)

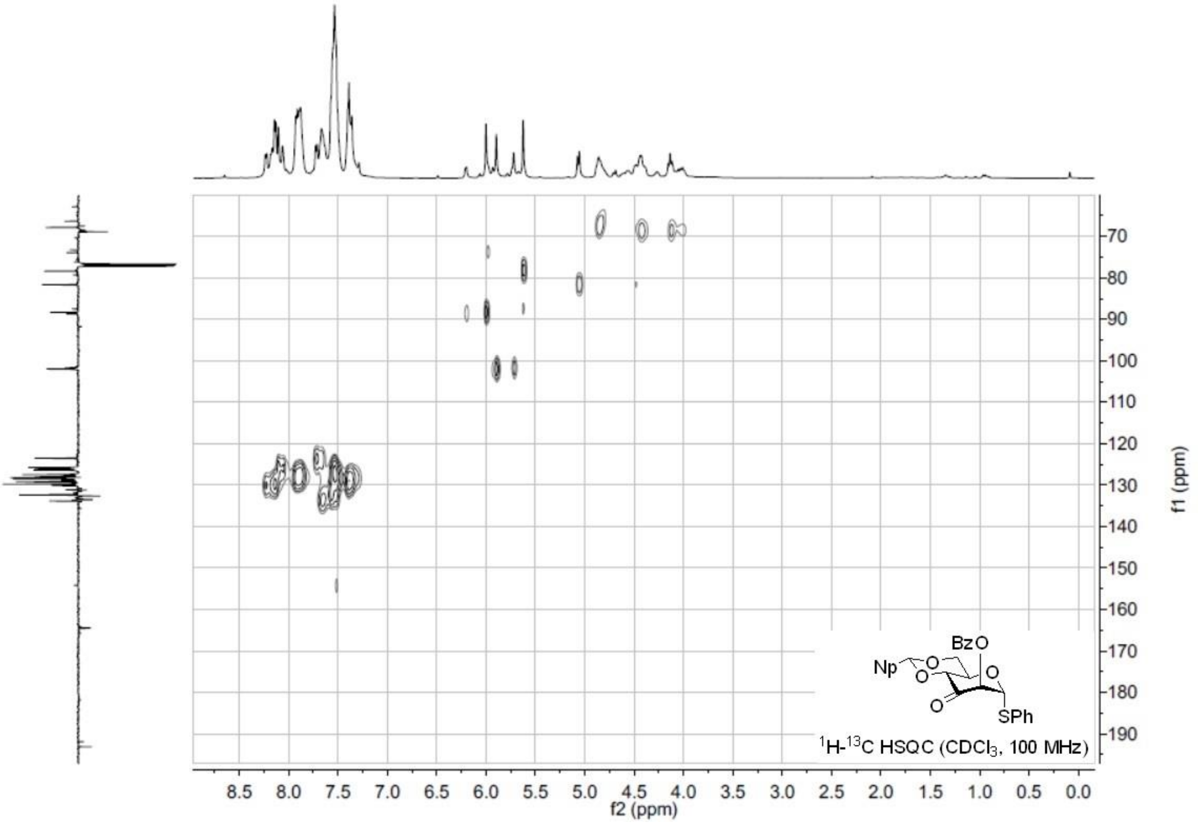

BzO
Np
O
SPh
^{1}H-^{13}C HSQC (CDCl$_3$, 100 MHz)
f2 (ppm)
f1 (ppm)

^{1}H and ^{13}C NMR spectra of compound **67**

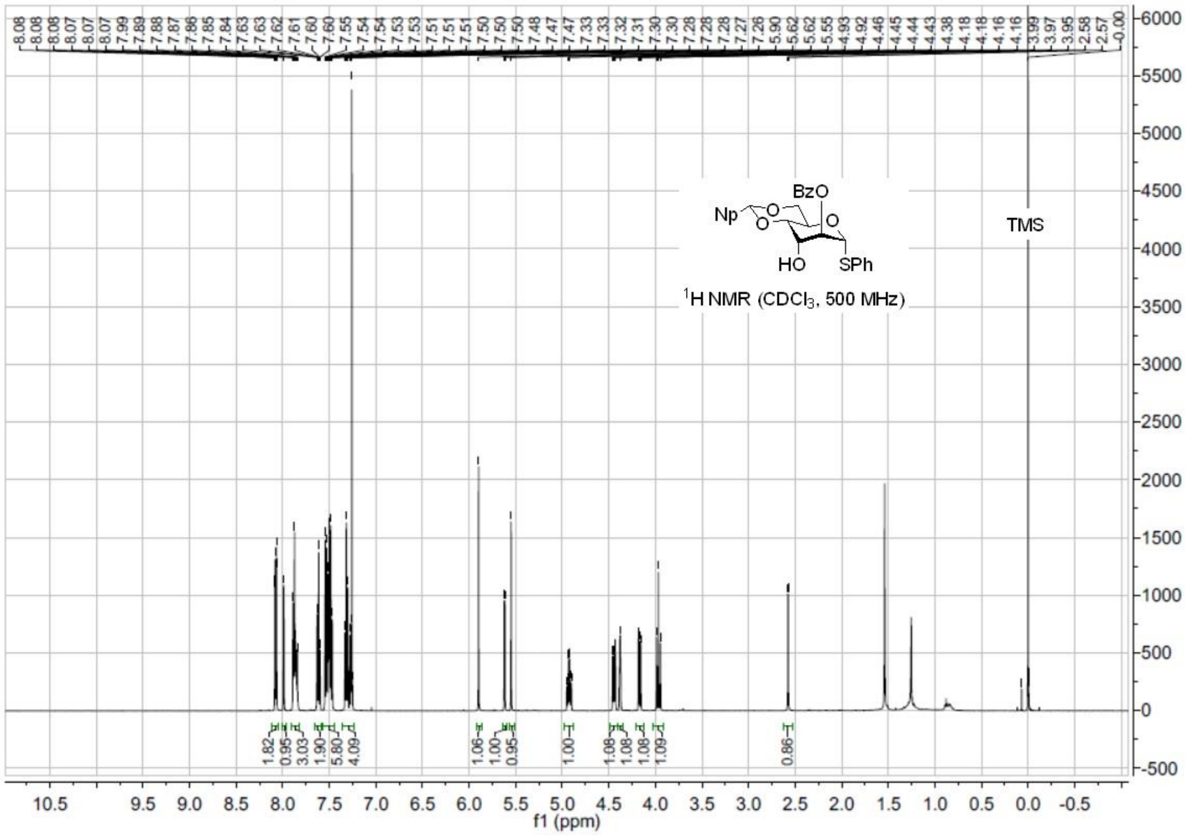

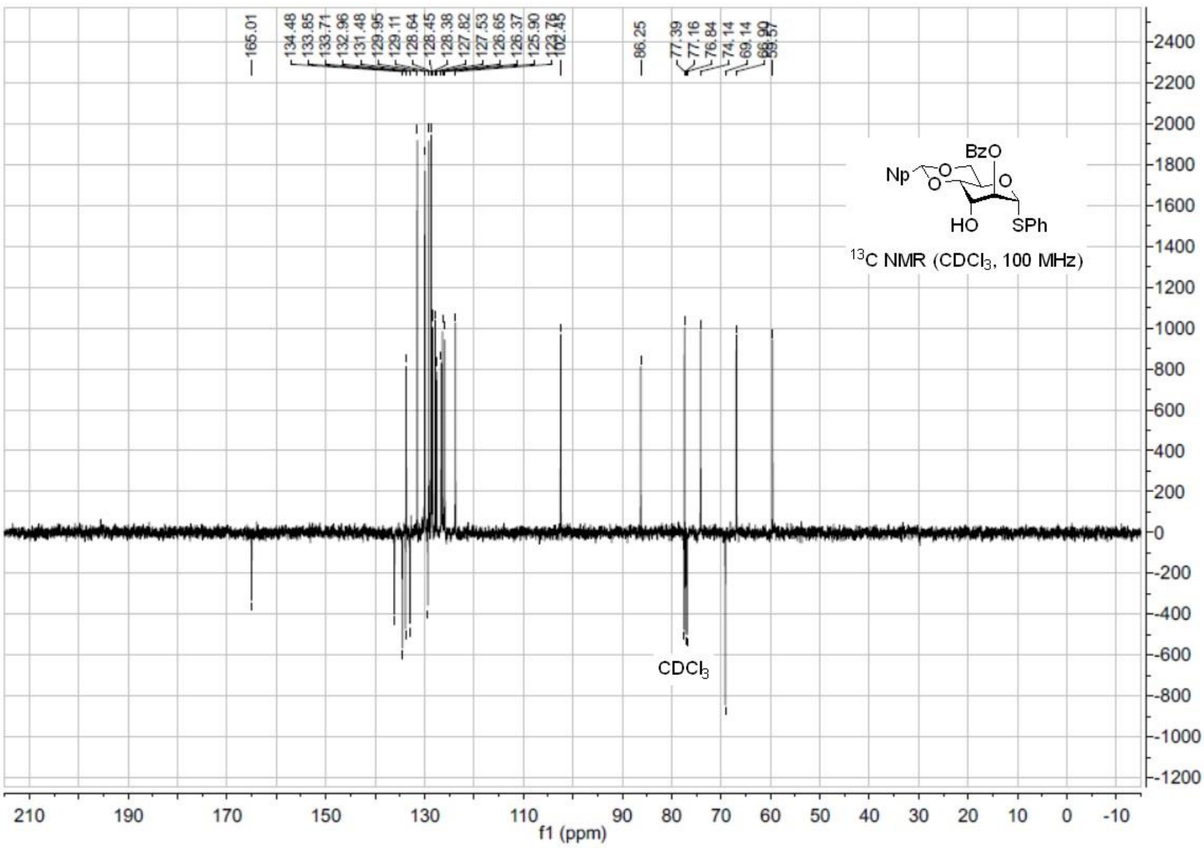

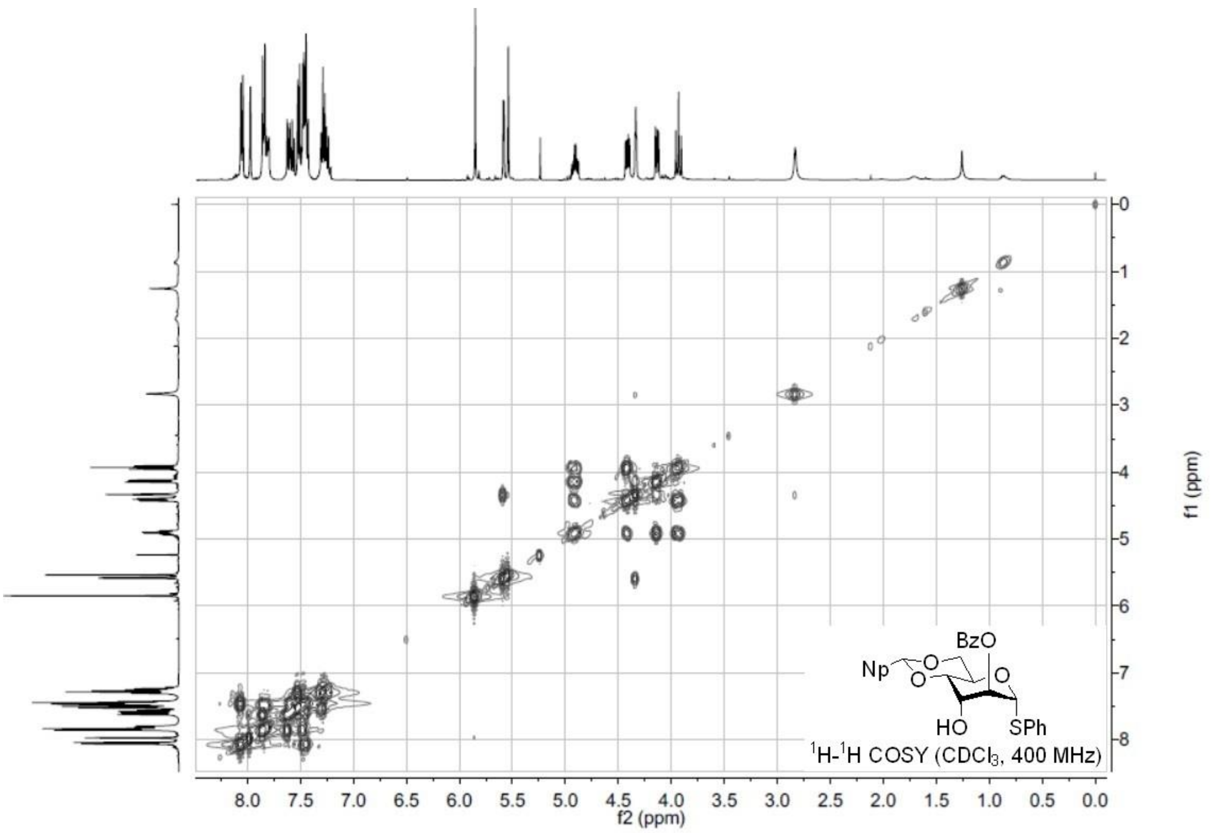
BzO
Np
O
O
O
HO
SPh
^{1}H-^{1}H COSY (CDCl$_3$, 400 MHz)
f2 (ppm)
f1 (ppm)

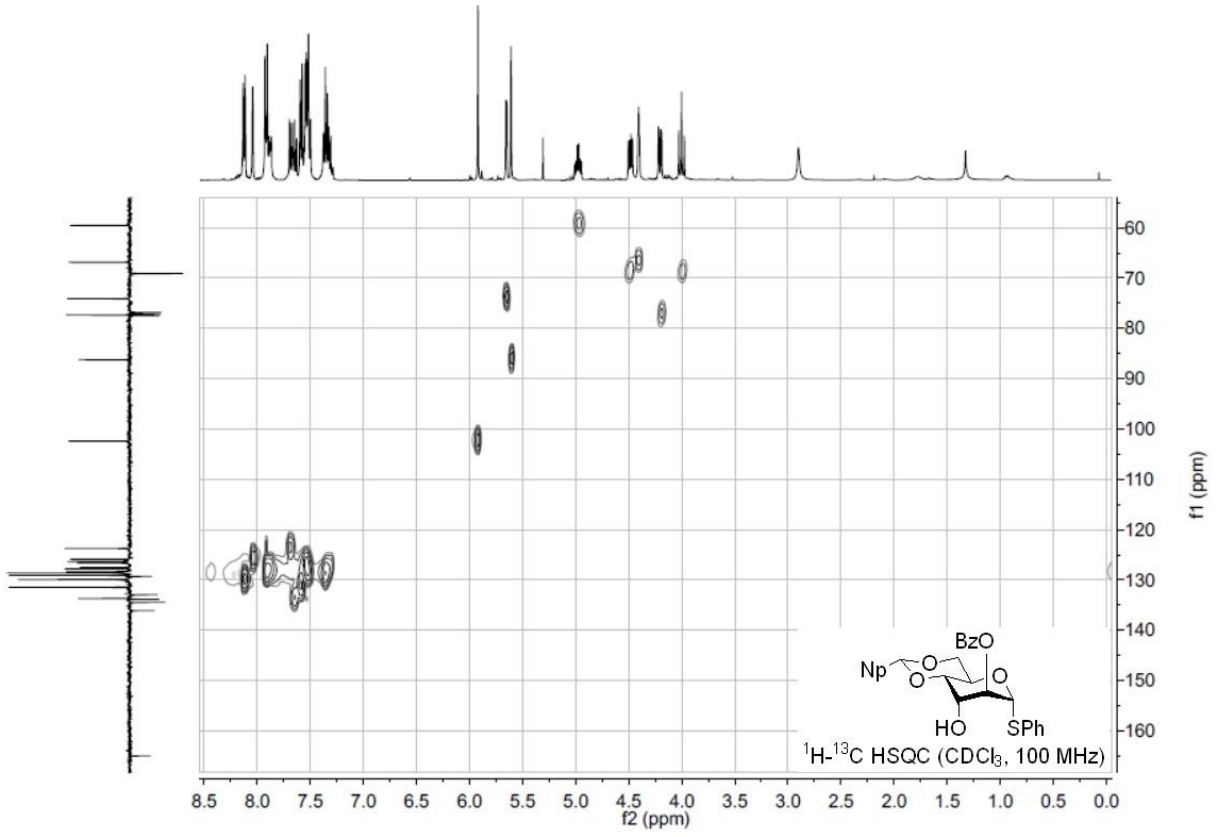
BzO
Np
O
O
O
HO
SPh
^{1}H-^{13}C HSQC (CDCl$_3$, 100 MHz)
f2 (ppm)
f1 (ppm)

^{1}H and ^{13}C NMR spectra of compound **68**

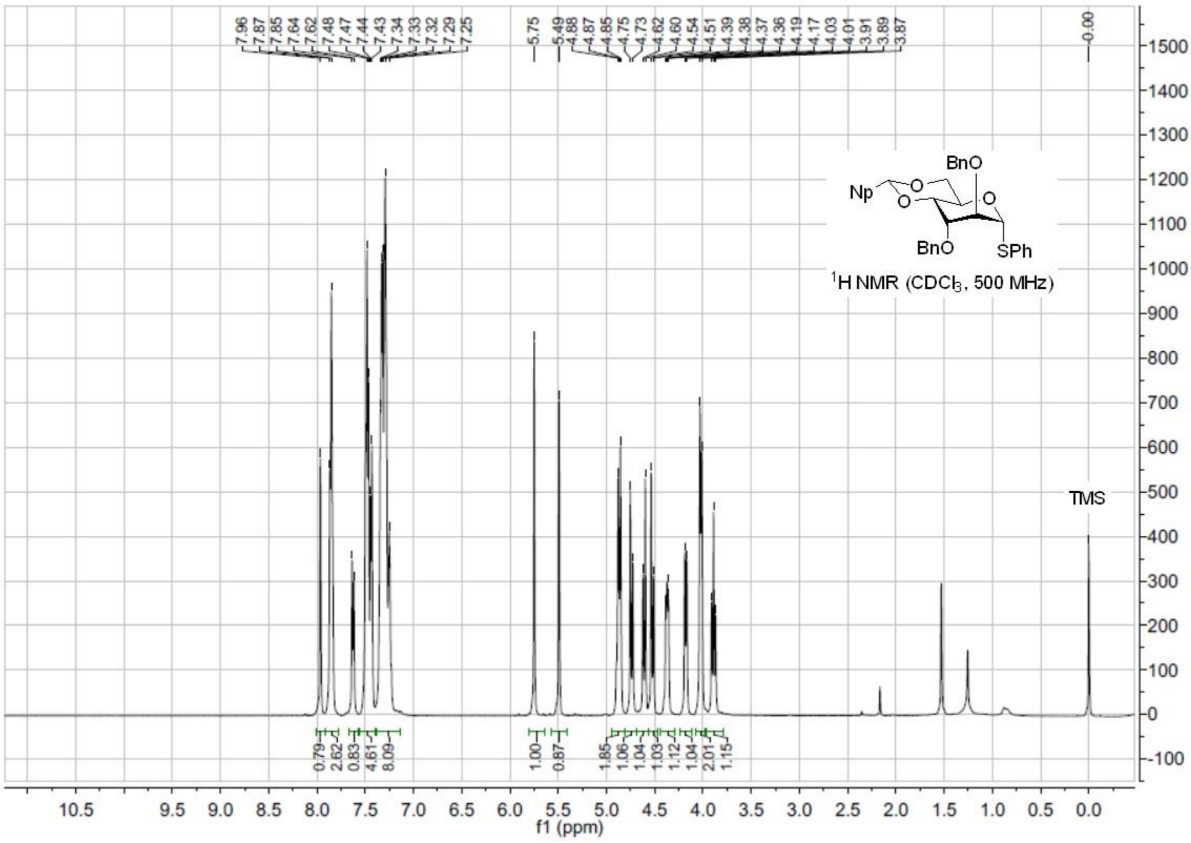

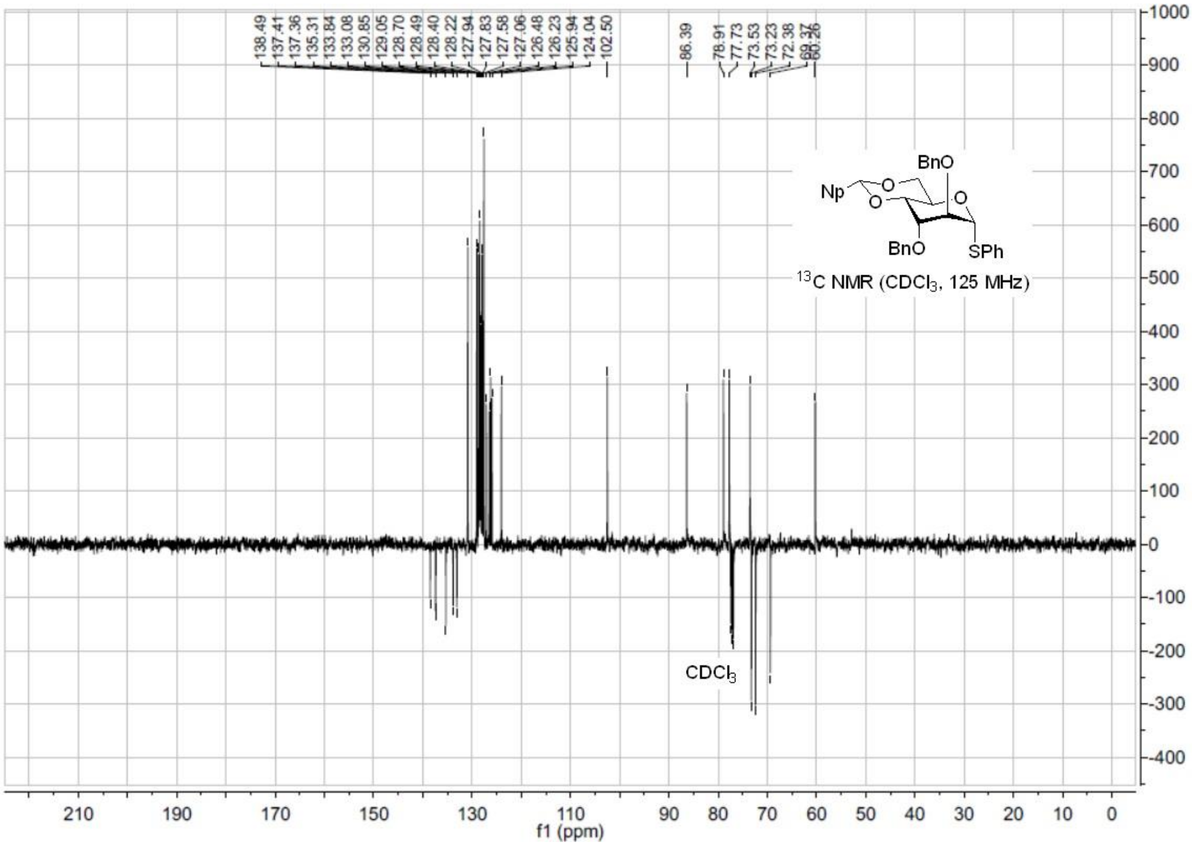

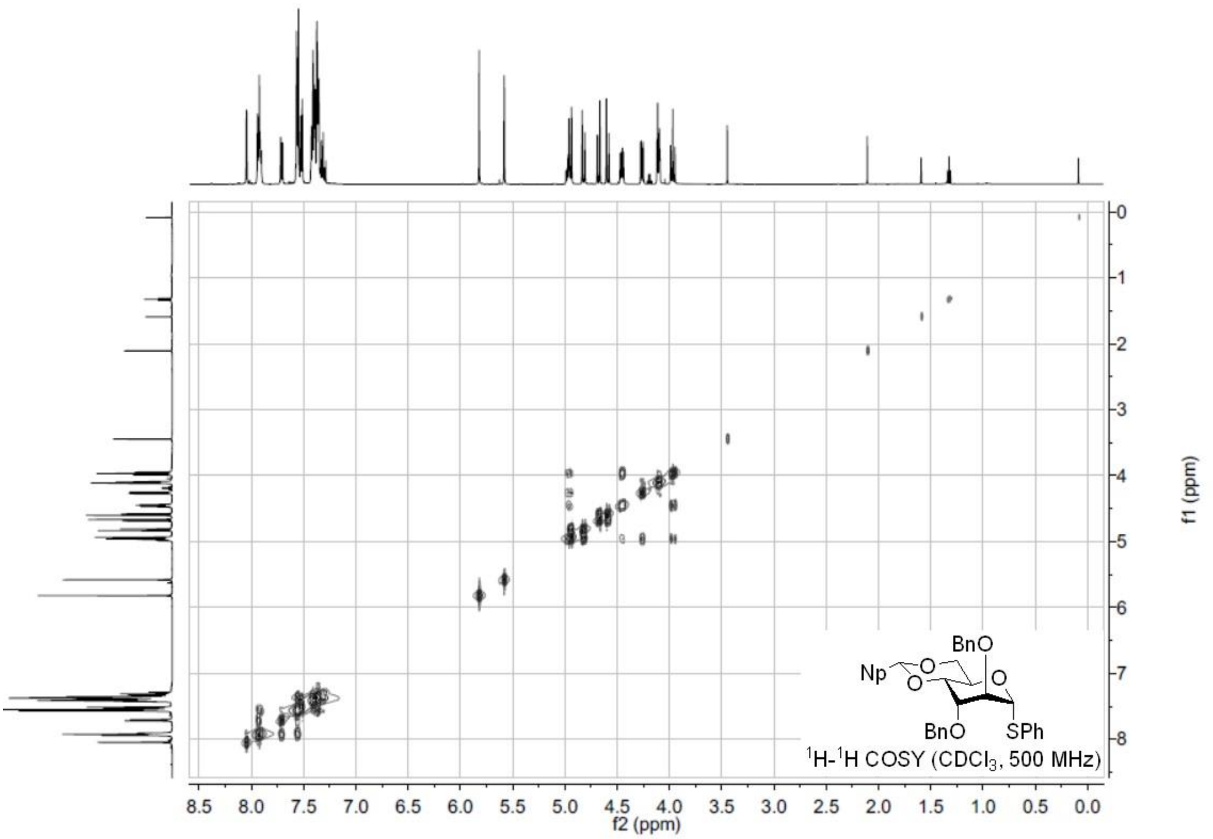
BnO
Np
O
O
O
BnO
SPh
1H-1H COSY (CDCl3, 500 MHz)
f1 (ppm)
f2 (ppm)

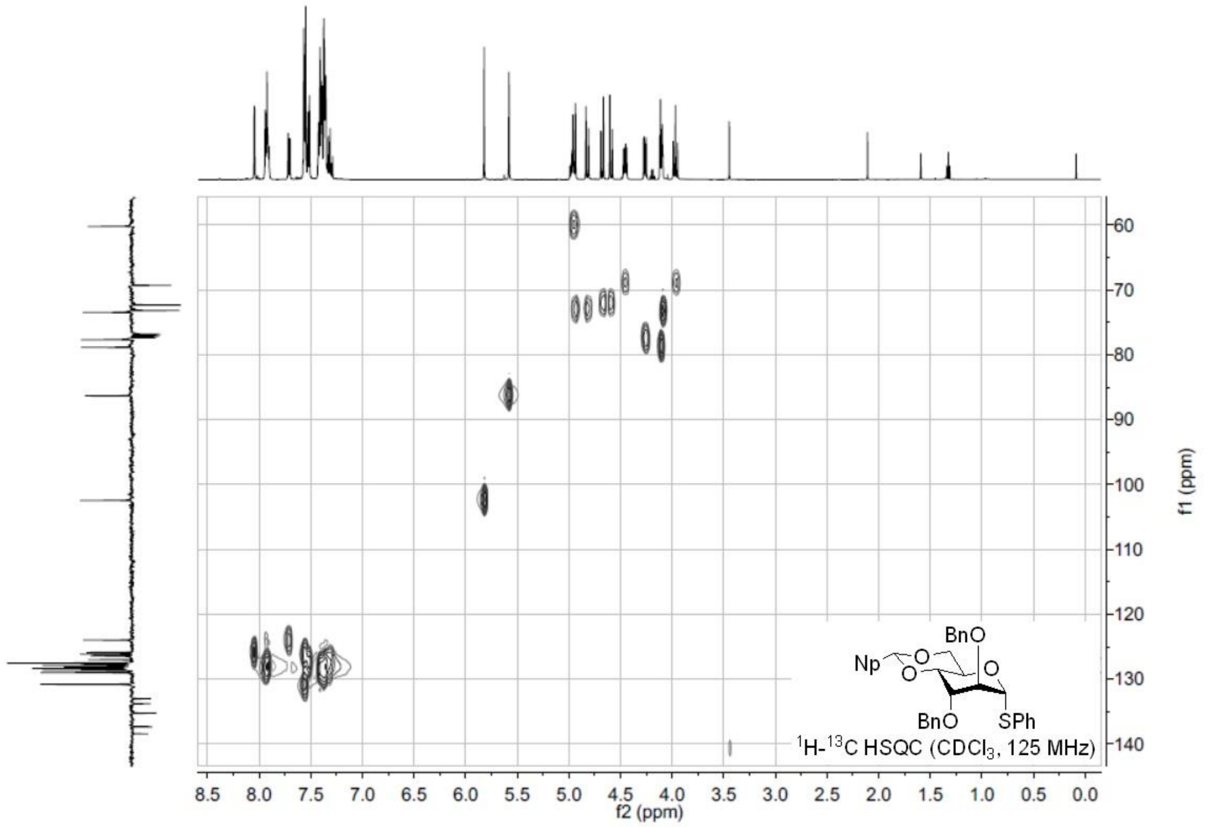
BnO
Np
O
O
O
BnO
SPh
1H-13C HSQC (CDCl3, 125 MHz)
f1 (ppm)
f2 (ppm)

^{1}H and ^{13}C NMR spectra of compound **69**

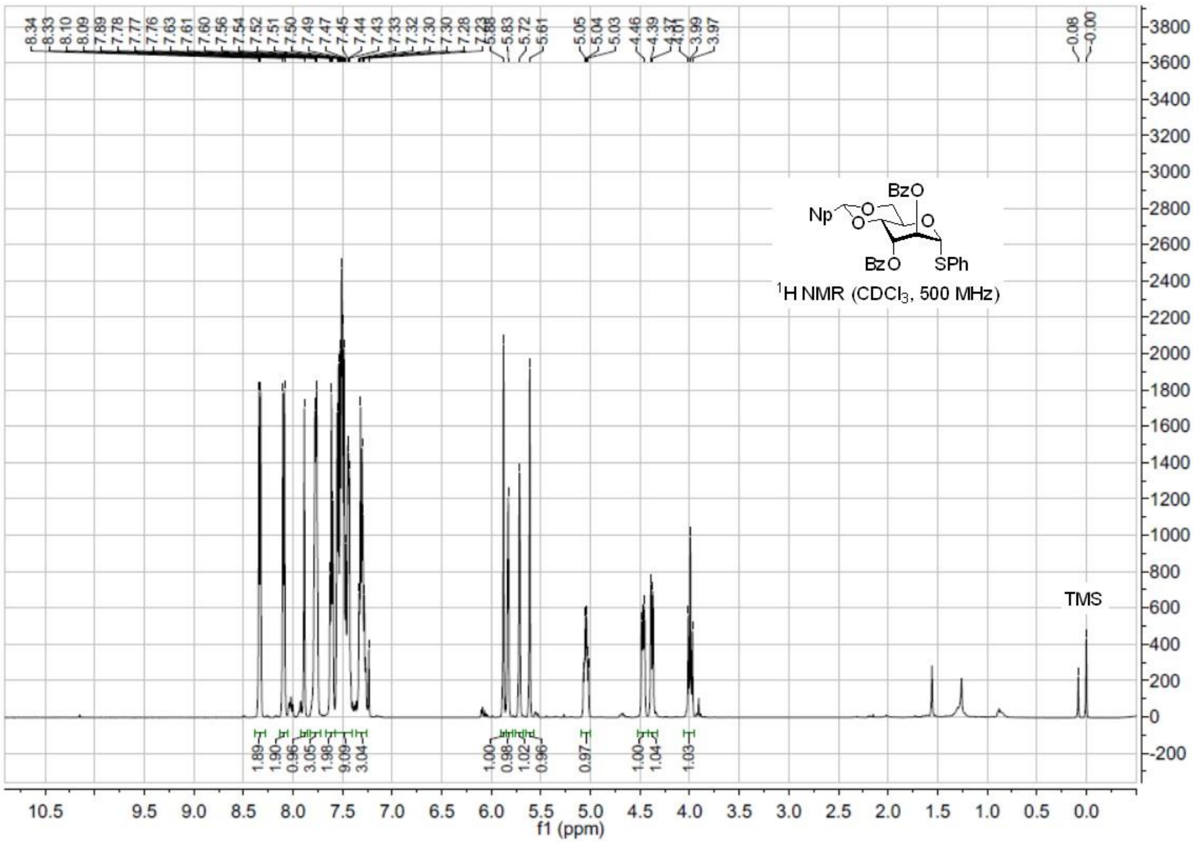

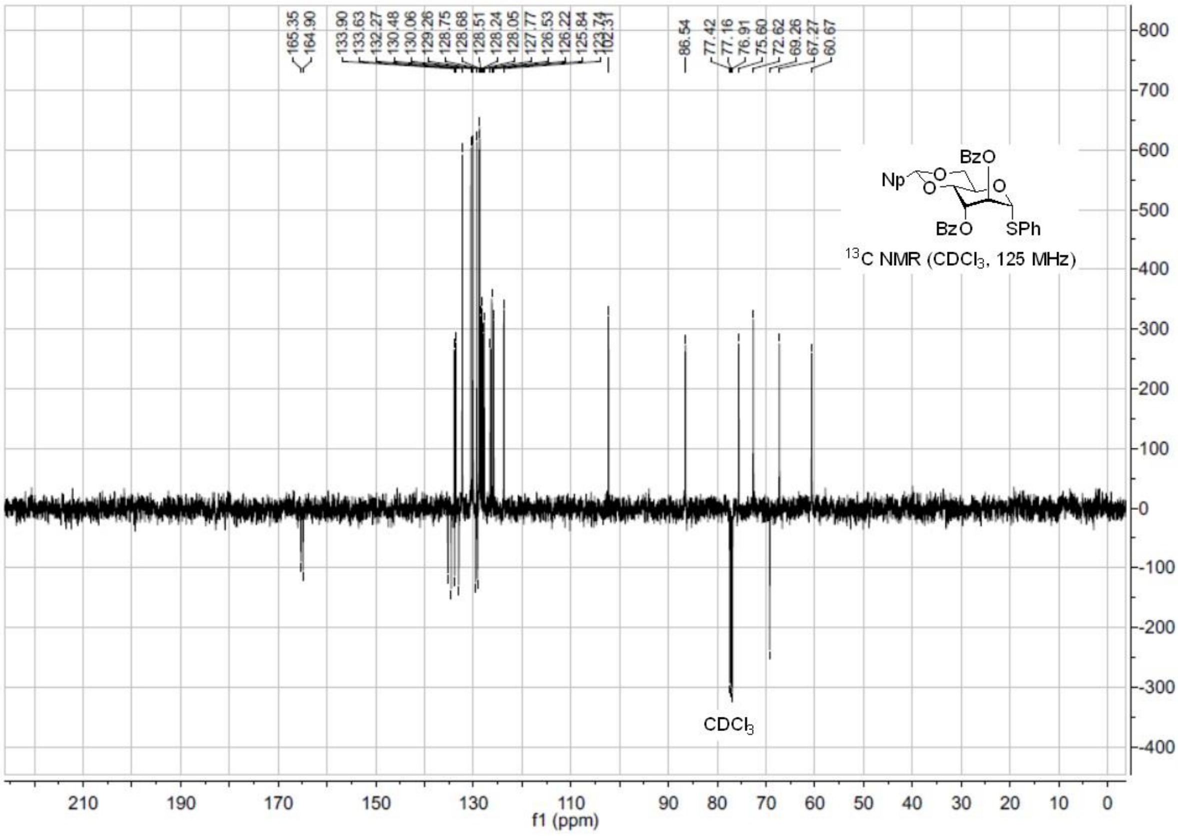

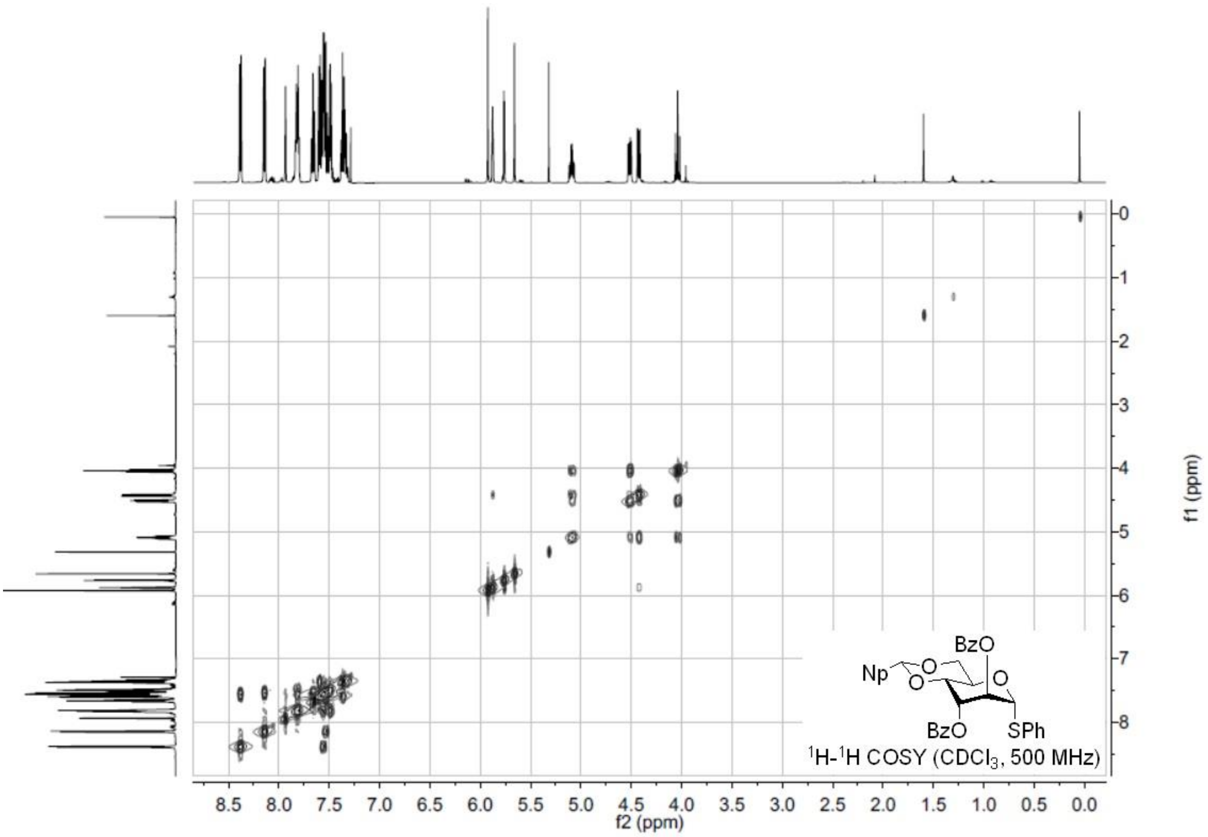

BzO
Np
O
O
O
BzO
SPh
^{1}H-^{1}H COSY (CDCl$_3$, 500 MHz)
f2 (ppm)
f1 (ppm)

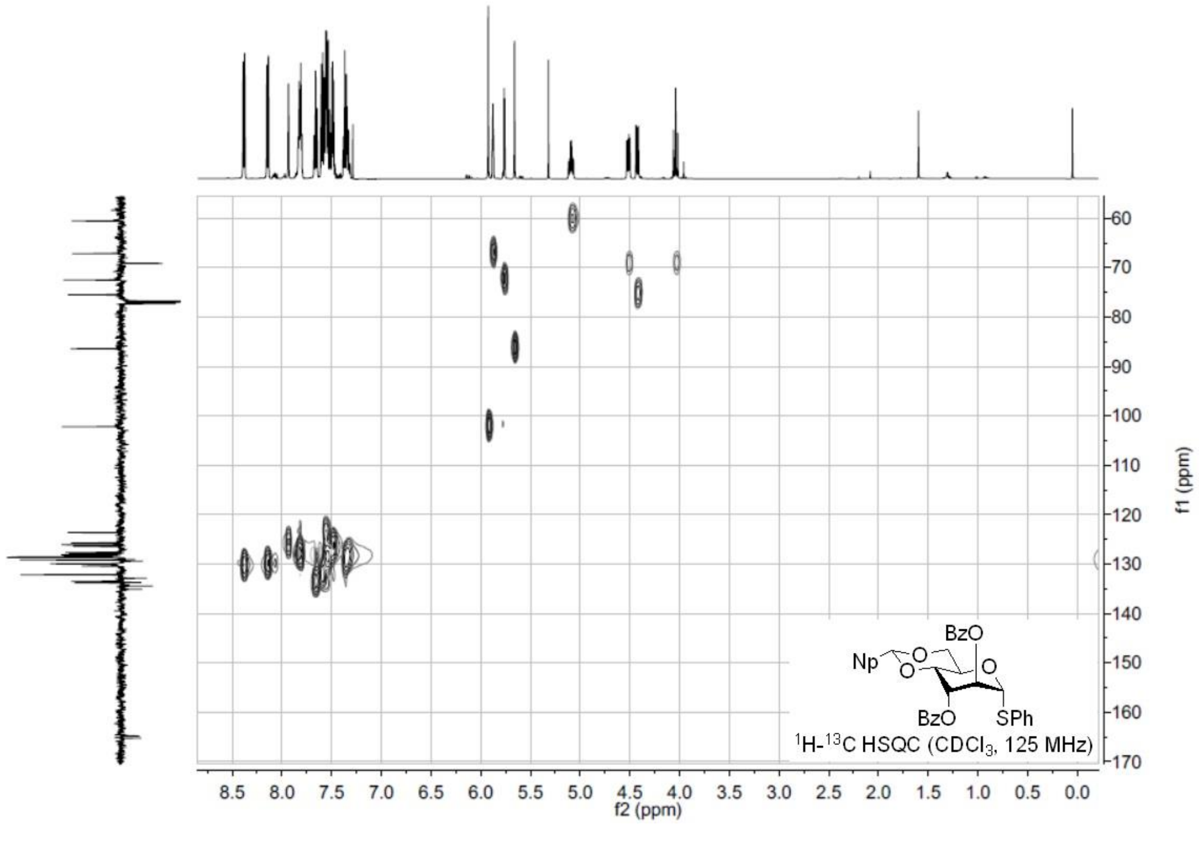

BzO
Np
O
O
O
BzO
SPh
^{1}H-^{13}C HSQC (CDCl$_3$, 125 MHz)
f2 (ppm)
f1 (ppm)

^1H and ^{13}C NMR spectra of compound **70**

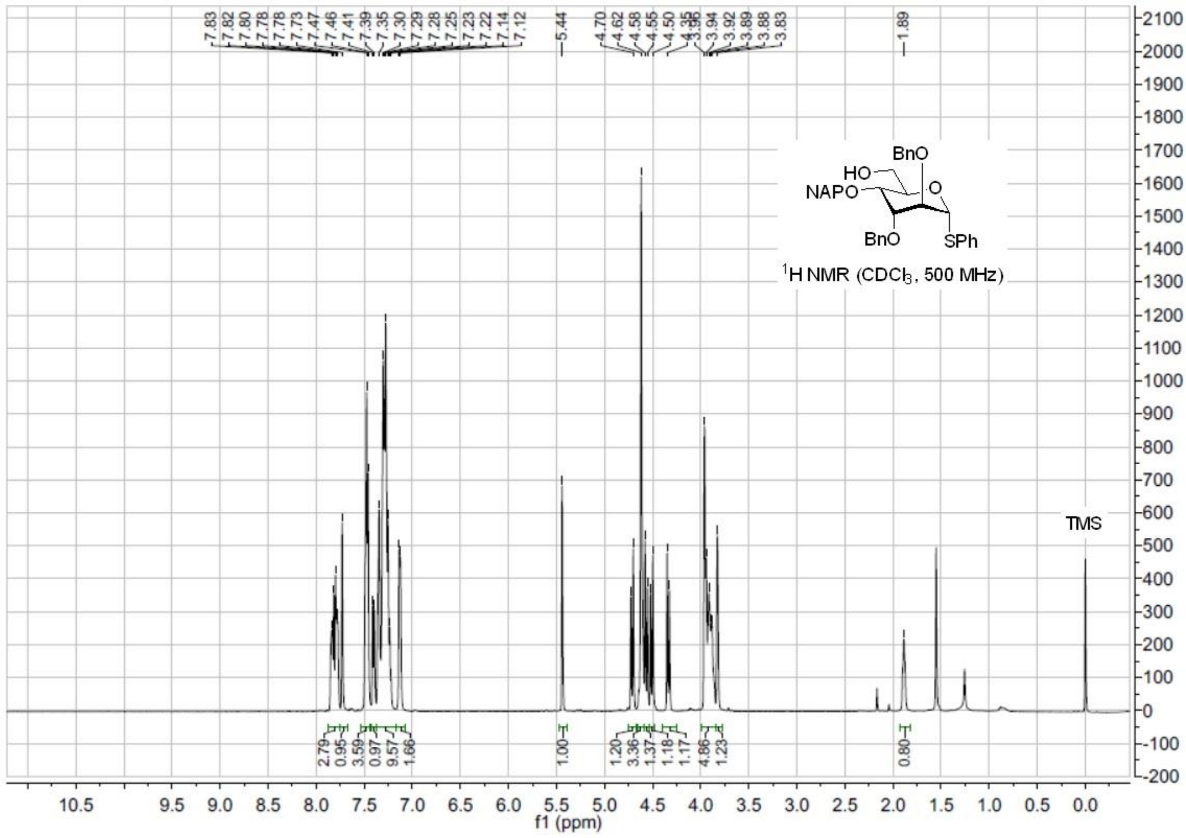

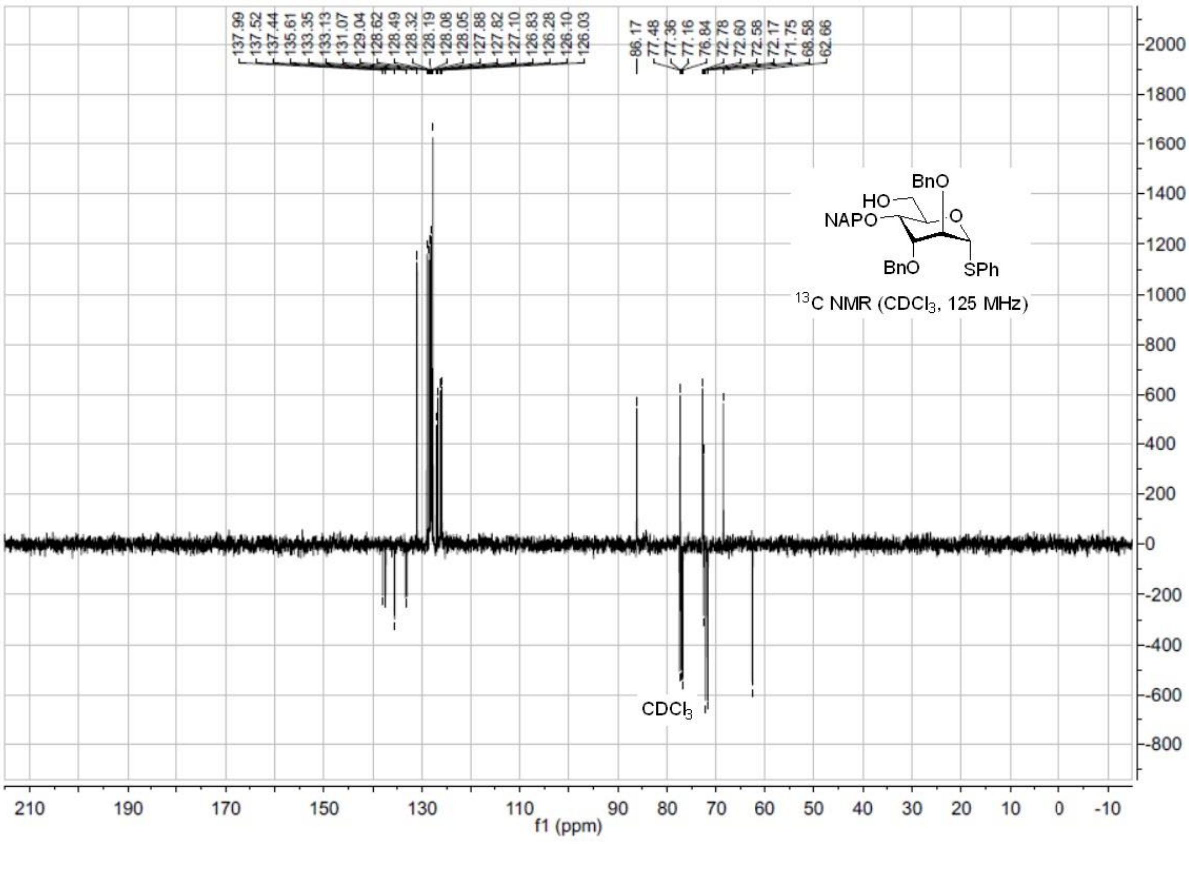

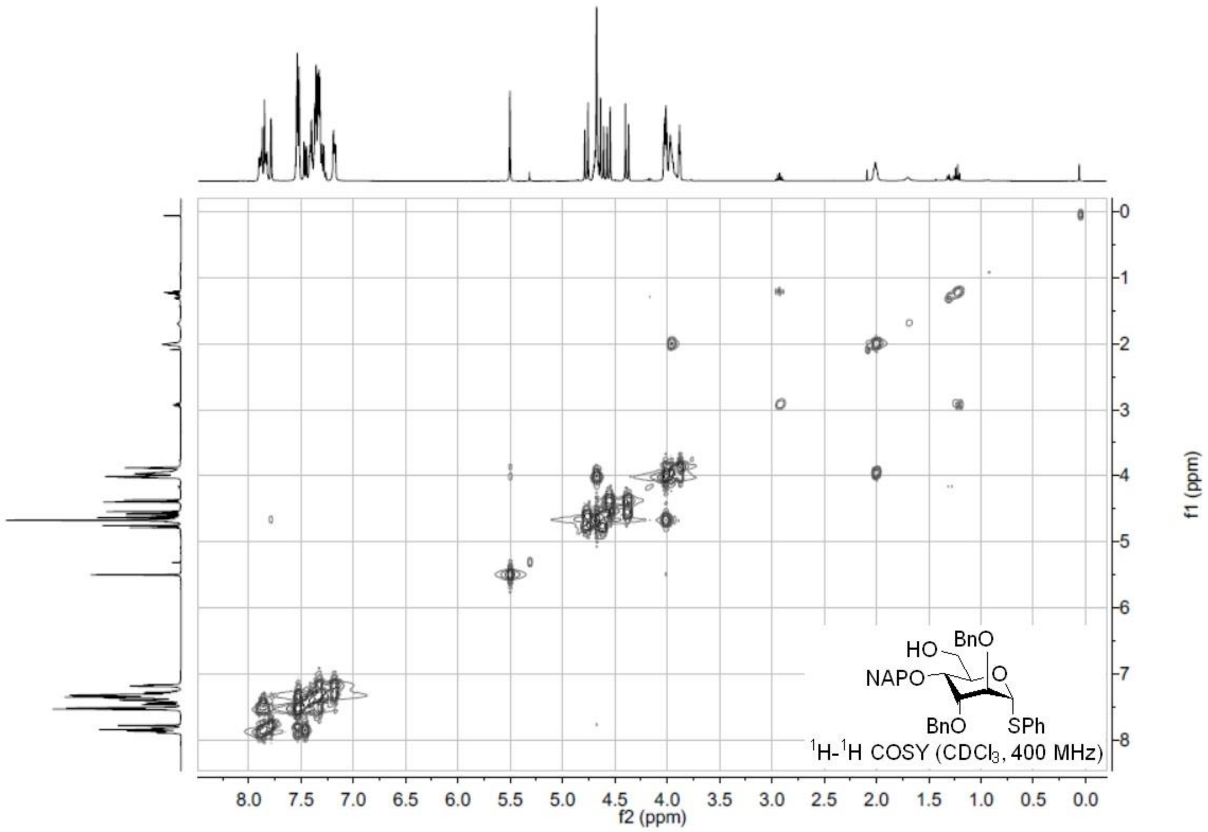
HO
BnO
NAPO
O
BnO
SPh
^{1}H-^{1}H COSY (CDCl$_3$, 400 MHz)
f1 (ppm)
f2 (ppm)

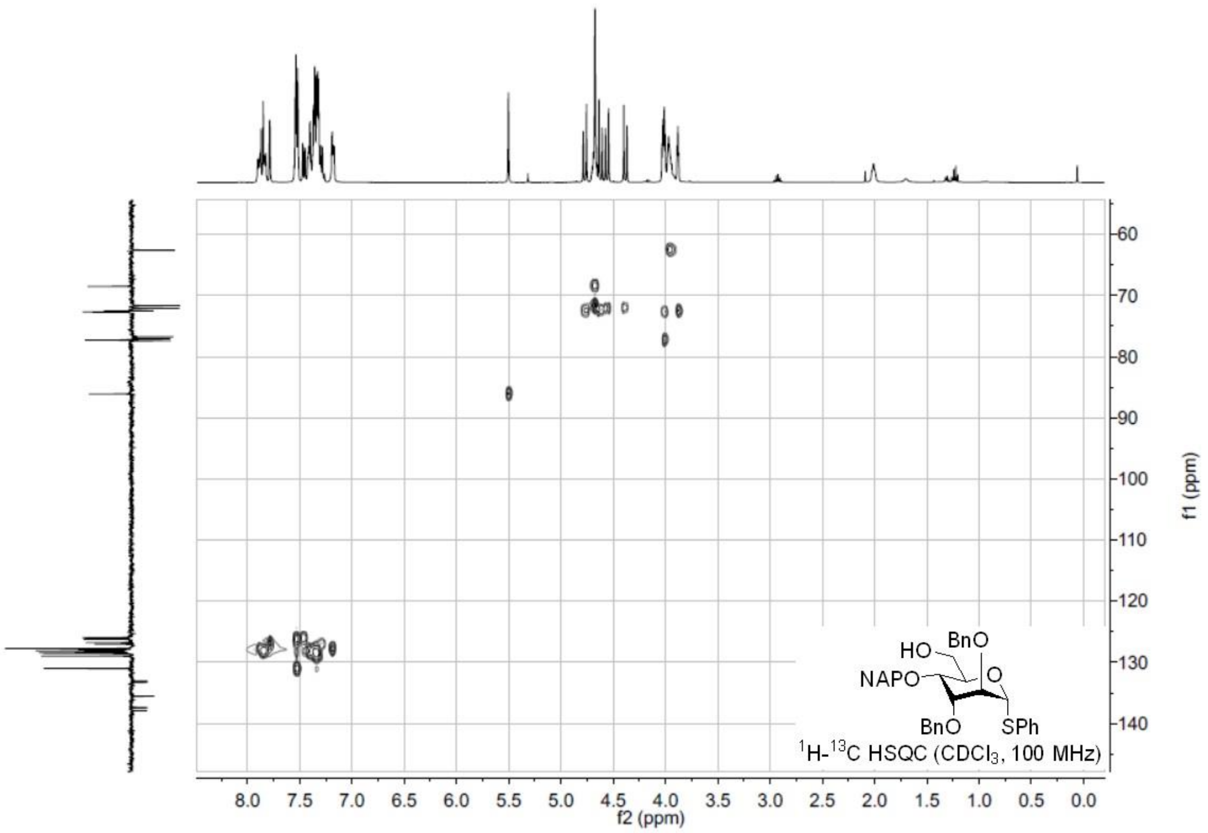
HO
BnO
NAPO
O
BnO
SPh
^{1}H-^{13}C HSQC (CDCl$_3$, 100 MHz)
f1 (ppm)
f2 (ppm)

^{1}H and ^{13}C NMR spectra of compound **71**

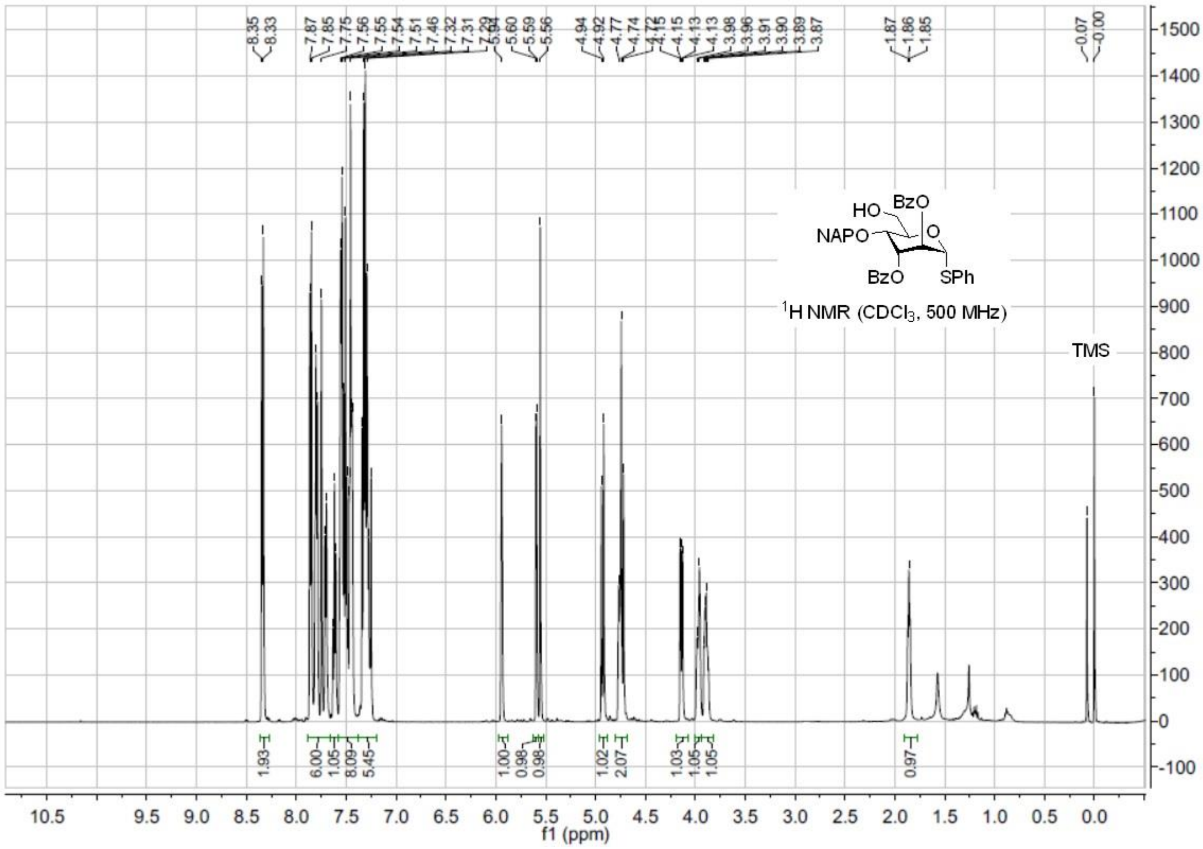

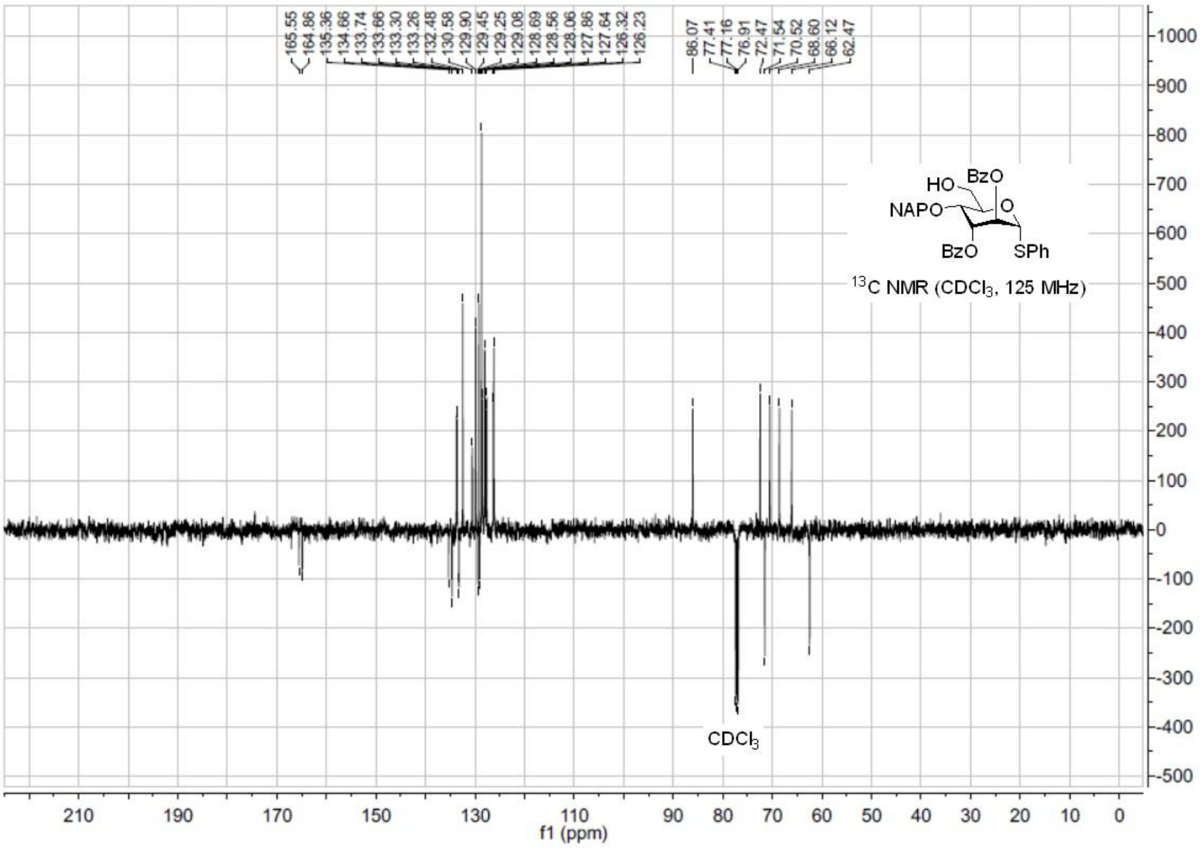

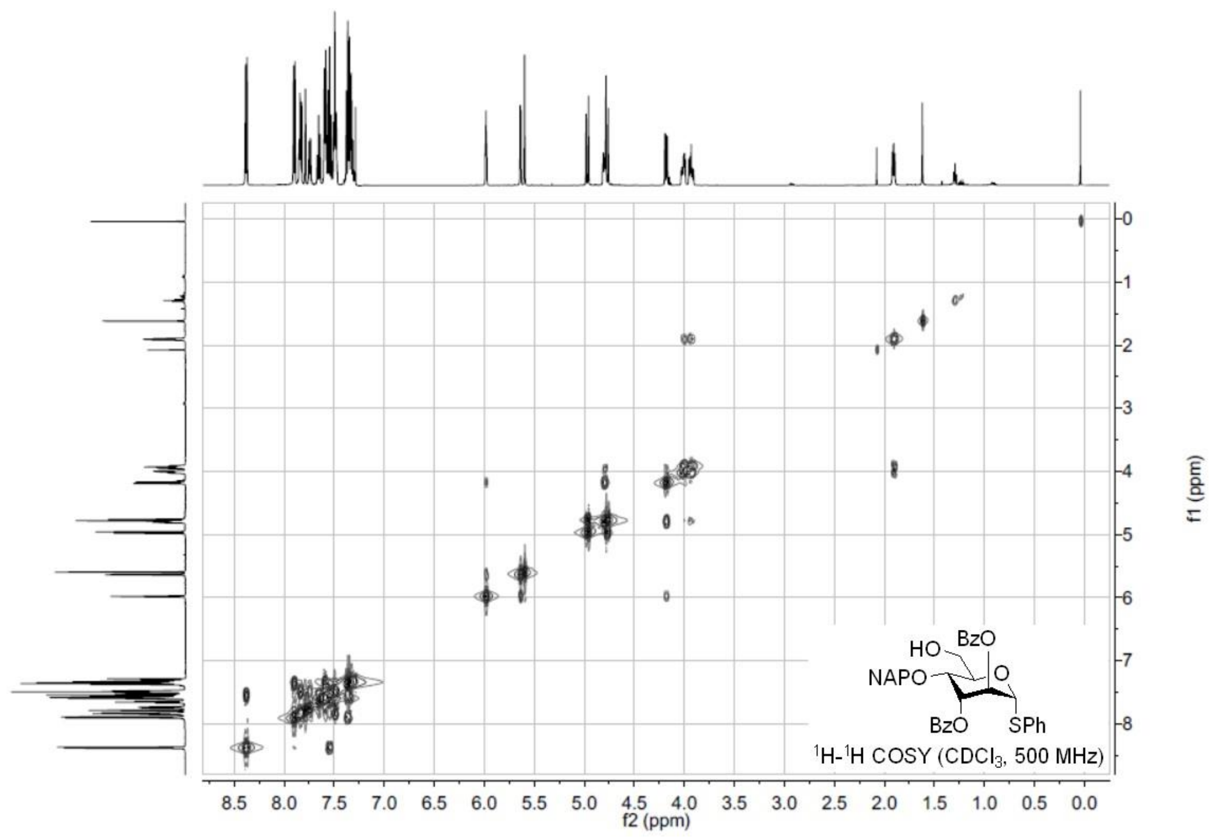
HO
BzO
NAPO
O
BzO
SPh
1H-1H COSY (CDCl3, 500 MHz)
f2 (ppm)
f1 (ppm)

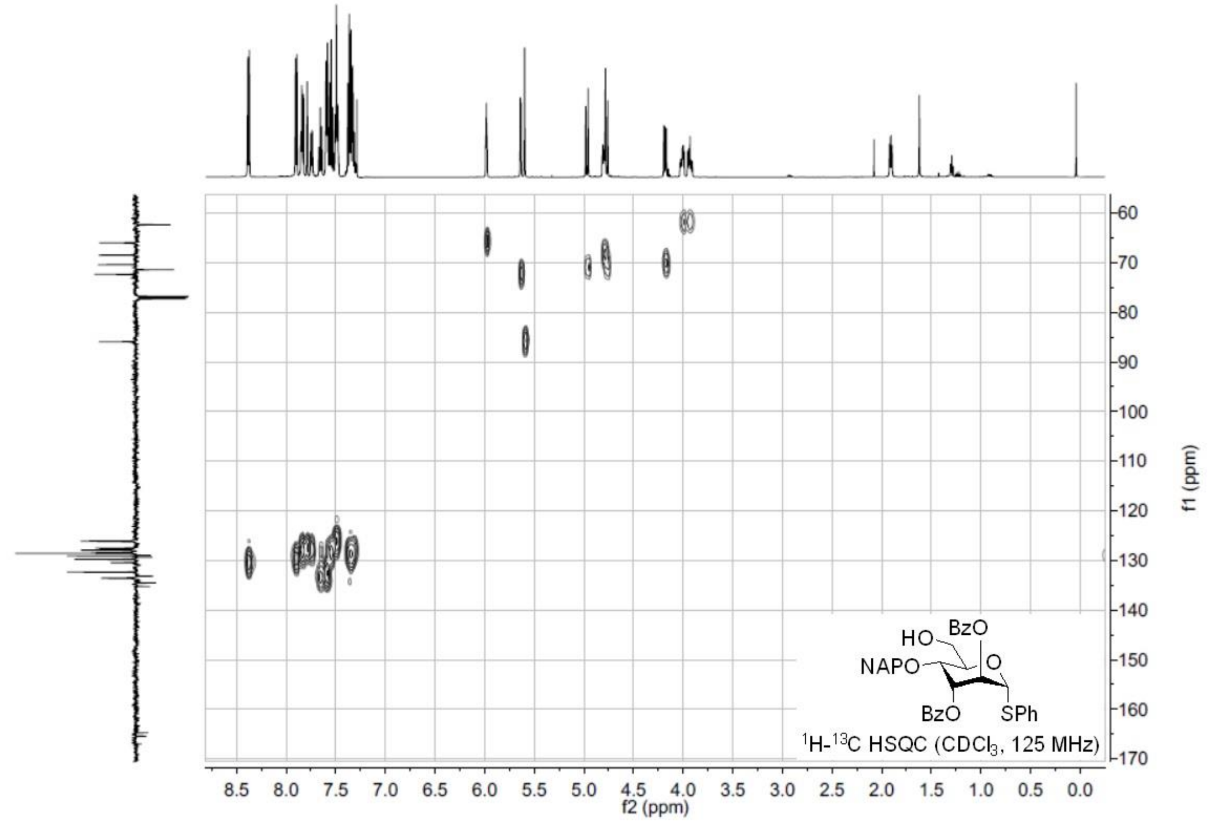
HO
BzO
NAPO
O
BzO
SPh
1H-13C HSQC (CDCl3, 125 MHz)
f2 (ppm)
f1 (ppm)

^{1}H and ^{13}C NMR spectra of compound **72**

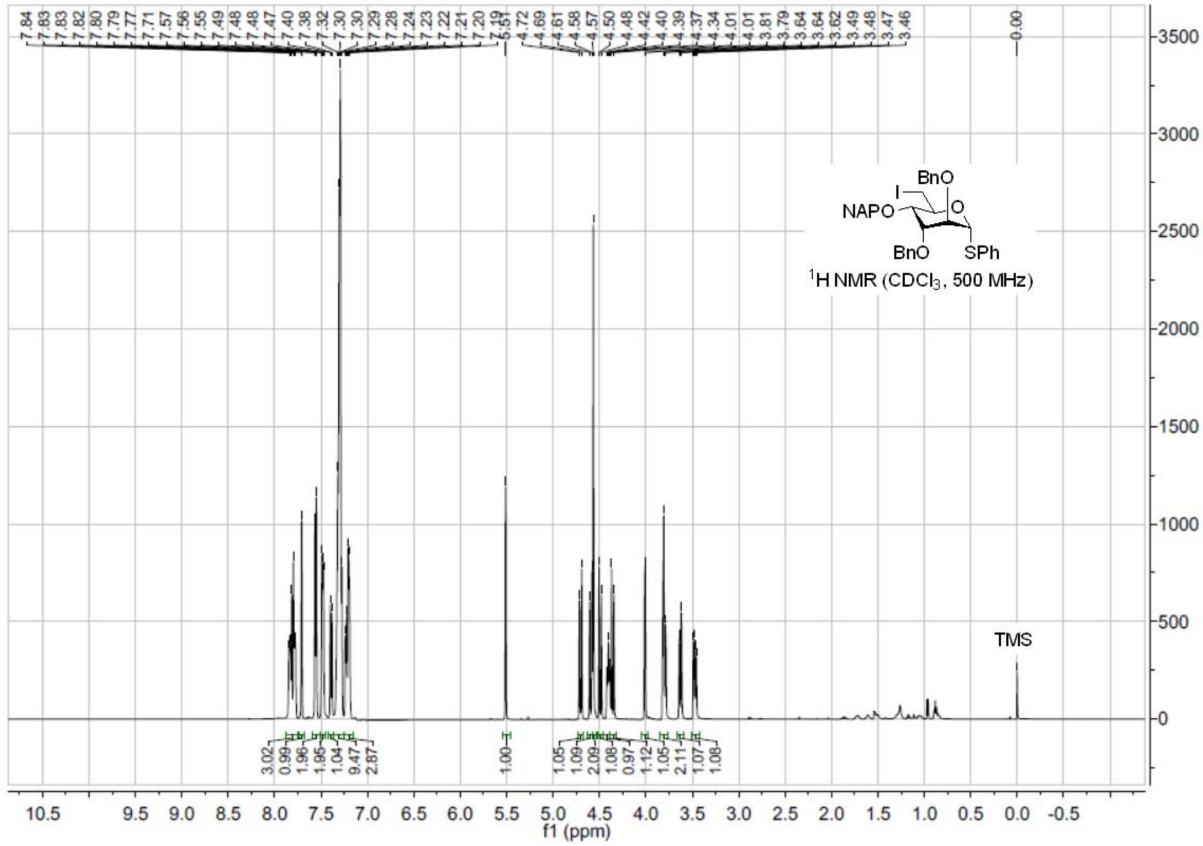

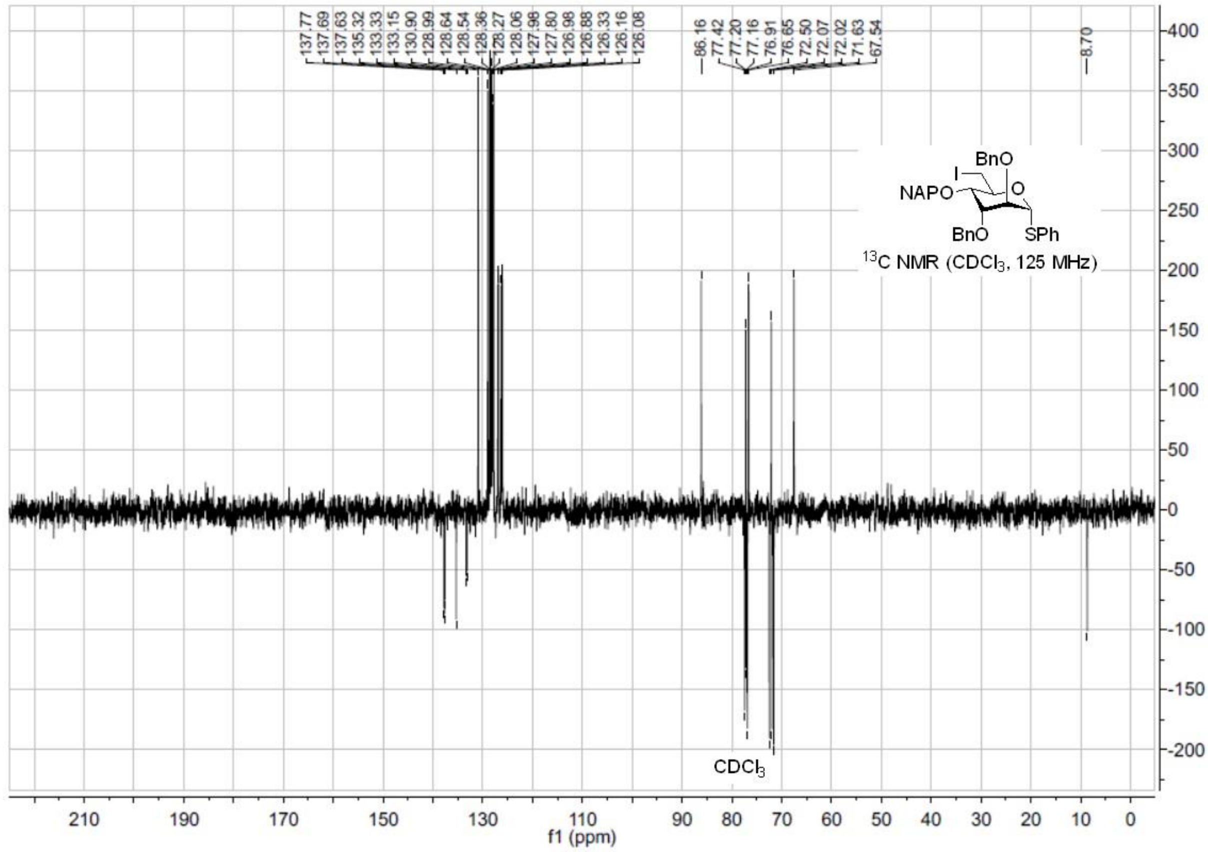

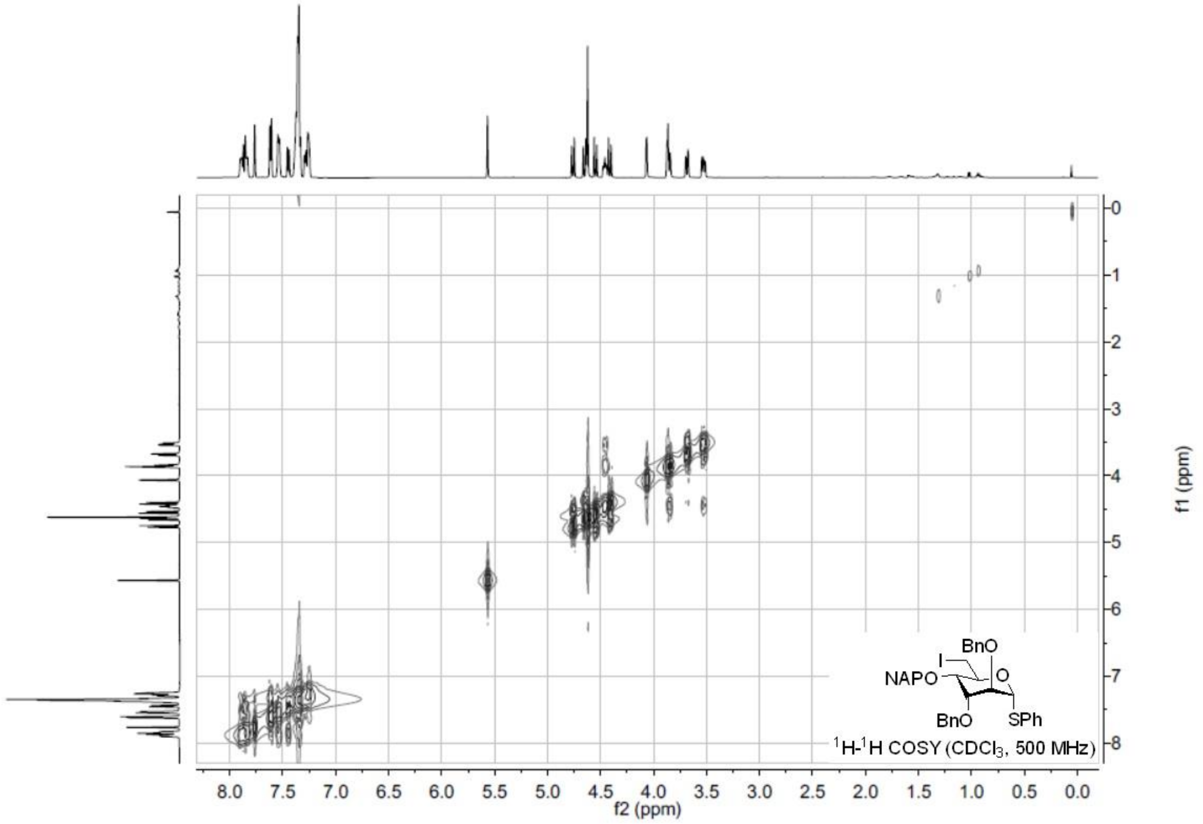
BnO
I
NAPO
O
BnO
SPh
^{1}H-^{1}H COSY (CDCl$_3$, 500 MHz)
f2 (ppm)
f1 (ppm)

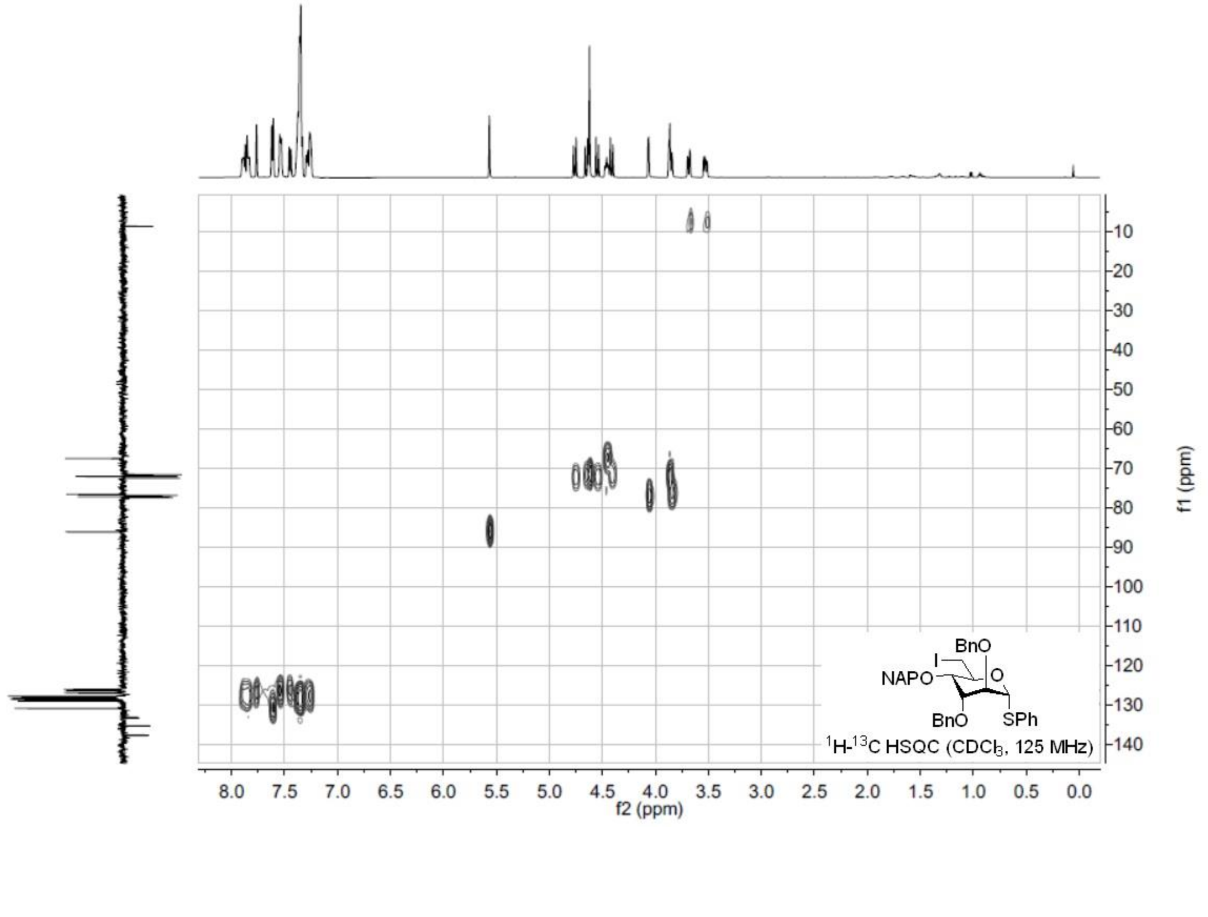
BnO
I
NAPO
O
BnO
SPh
^{1}H-^{13}C HSQC (CDCl$_3$, 125 MHz)
f2 (ppm)
f1 (ppm)

^{1}H and ^{13}C NMR spectra of compound **73**

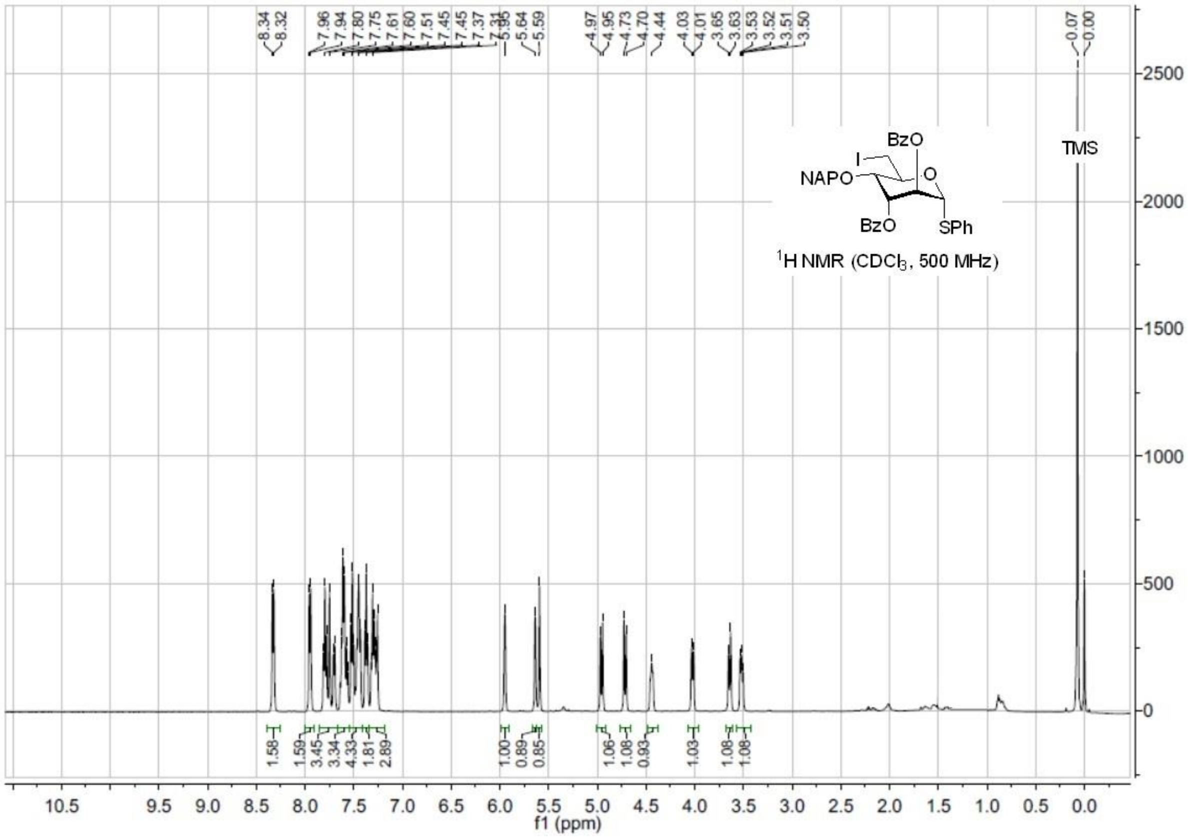

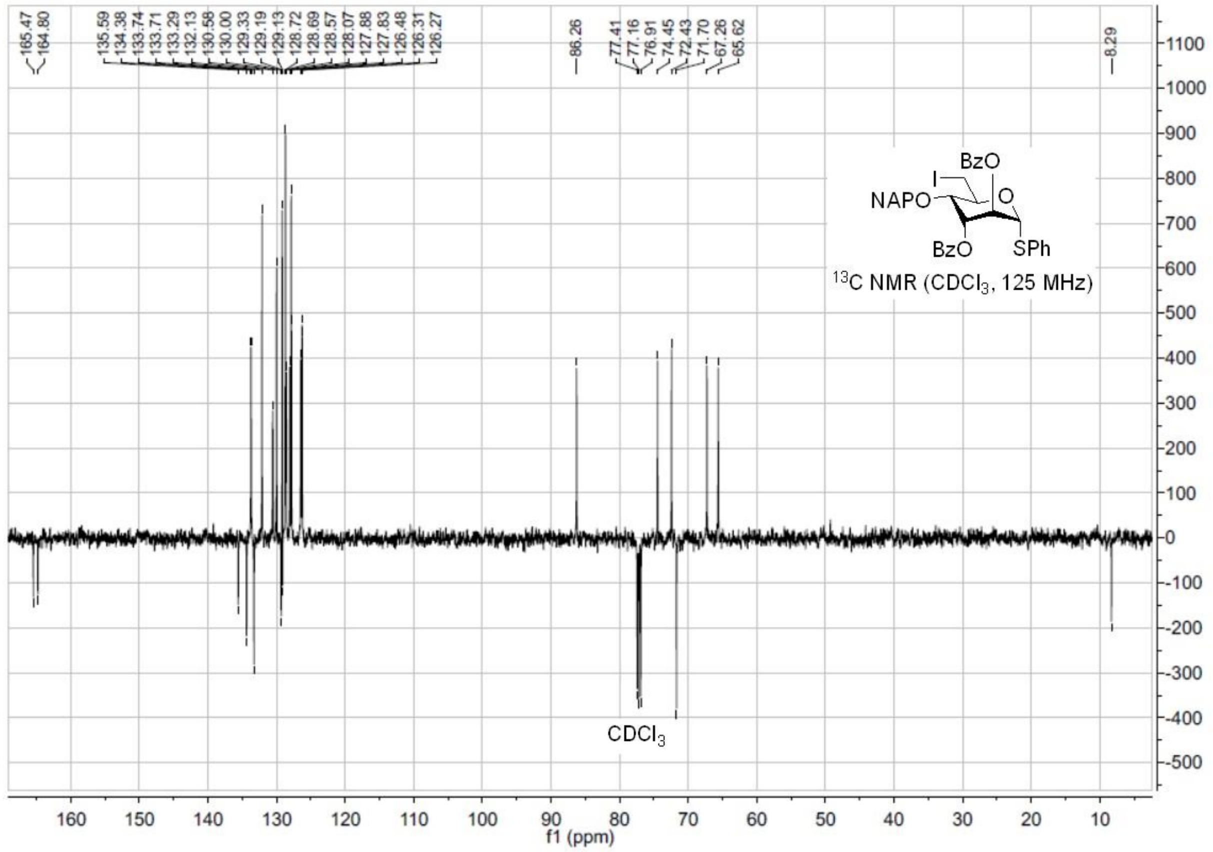

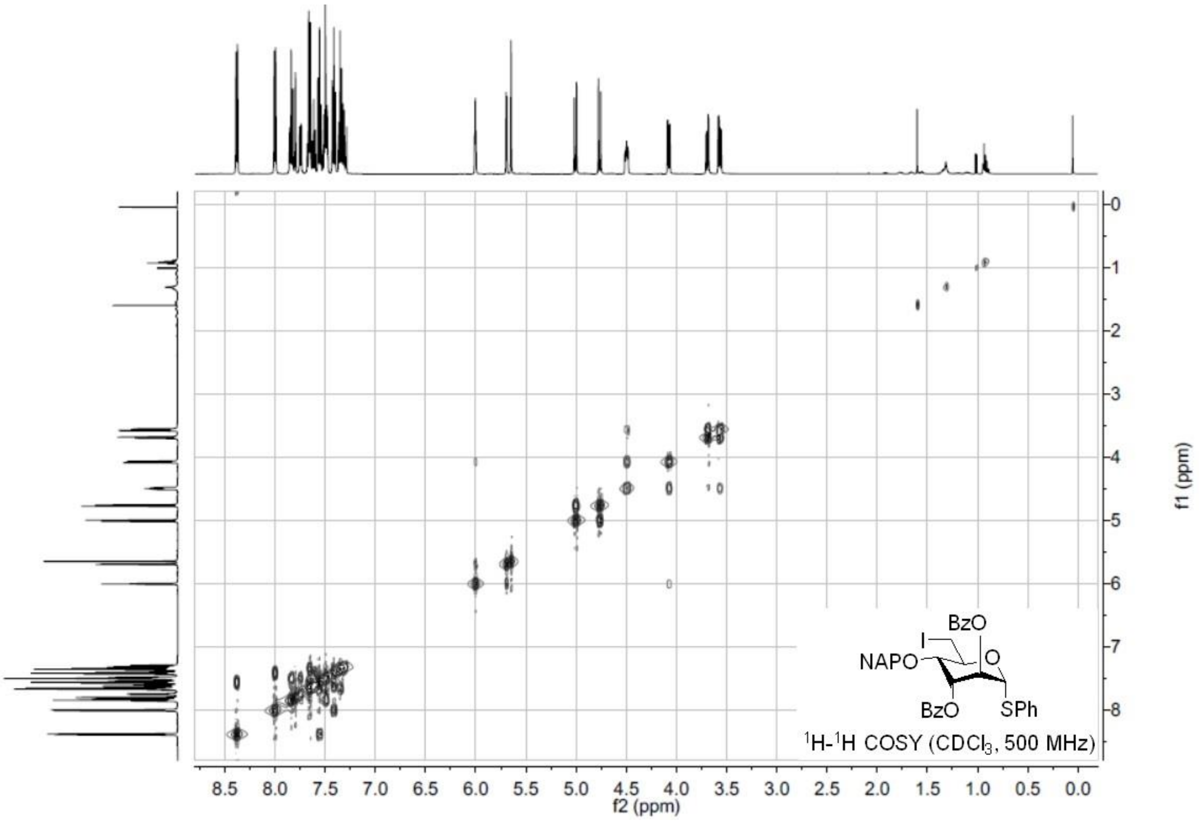
BzO
I
NAPO
O
BzO
SPh
^{1}H-^{1}H COSY (CDCl$_3$, 500 MHz)
f2 (ppm)
f1 (ppm)

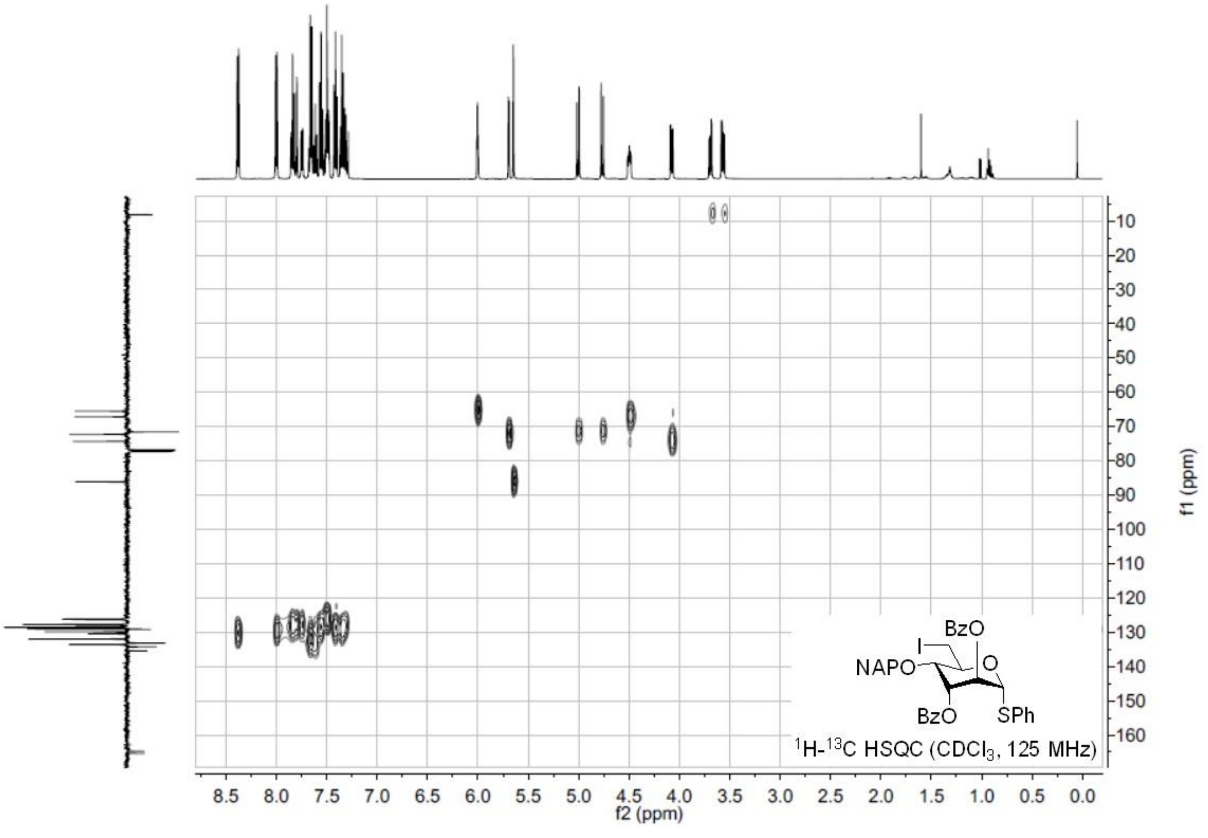
BzO
I
NAPO
O
BzO
SPh
^{1}H-^{13}C HSQC (CDCl$_3$, 125 MHz)
f2 (ppm)
f1 (ppm)

^{1}H and ^{13}C NMR spectra of compound **74**

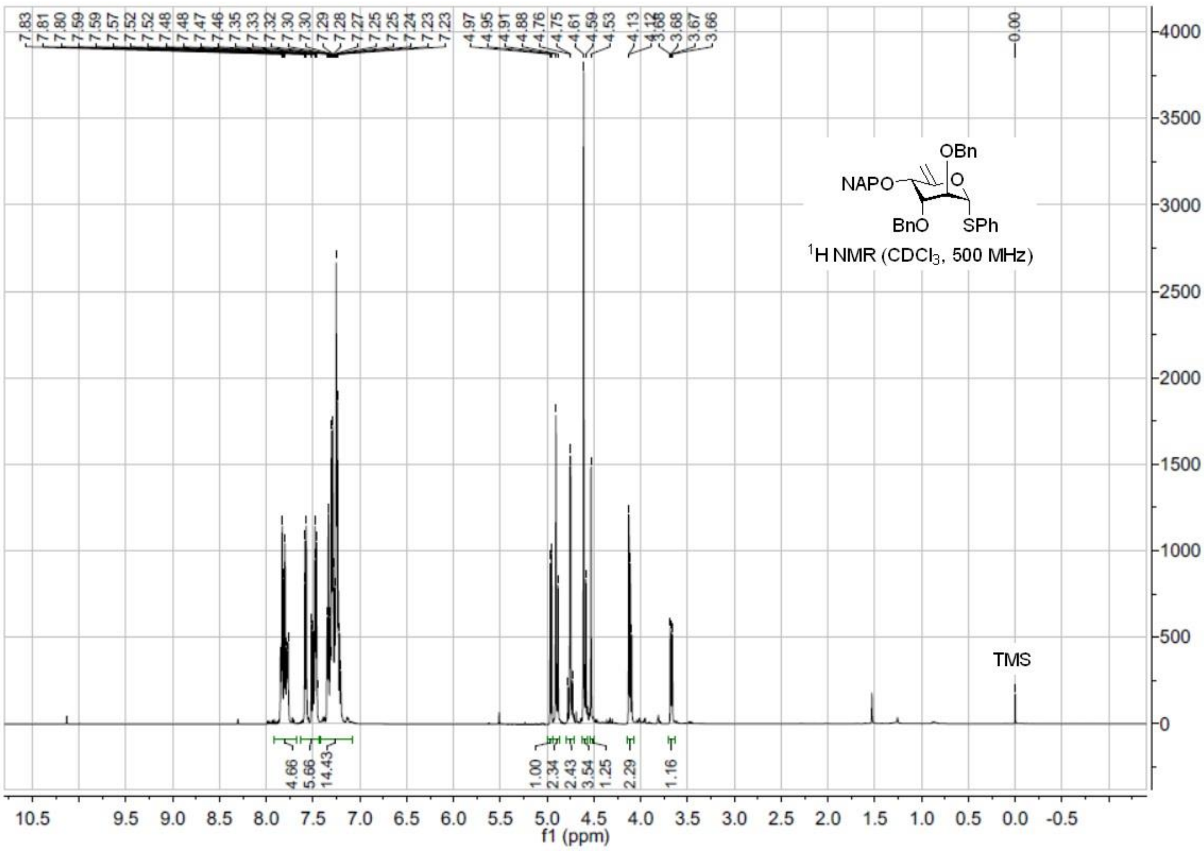

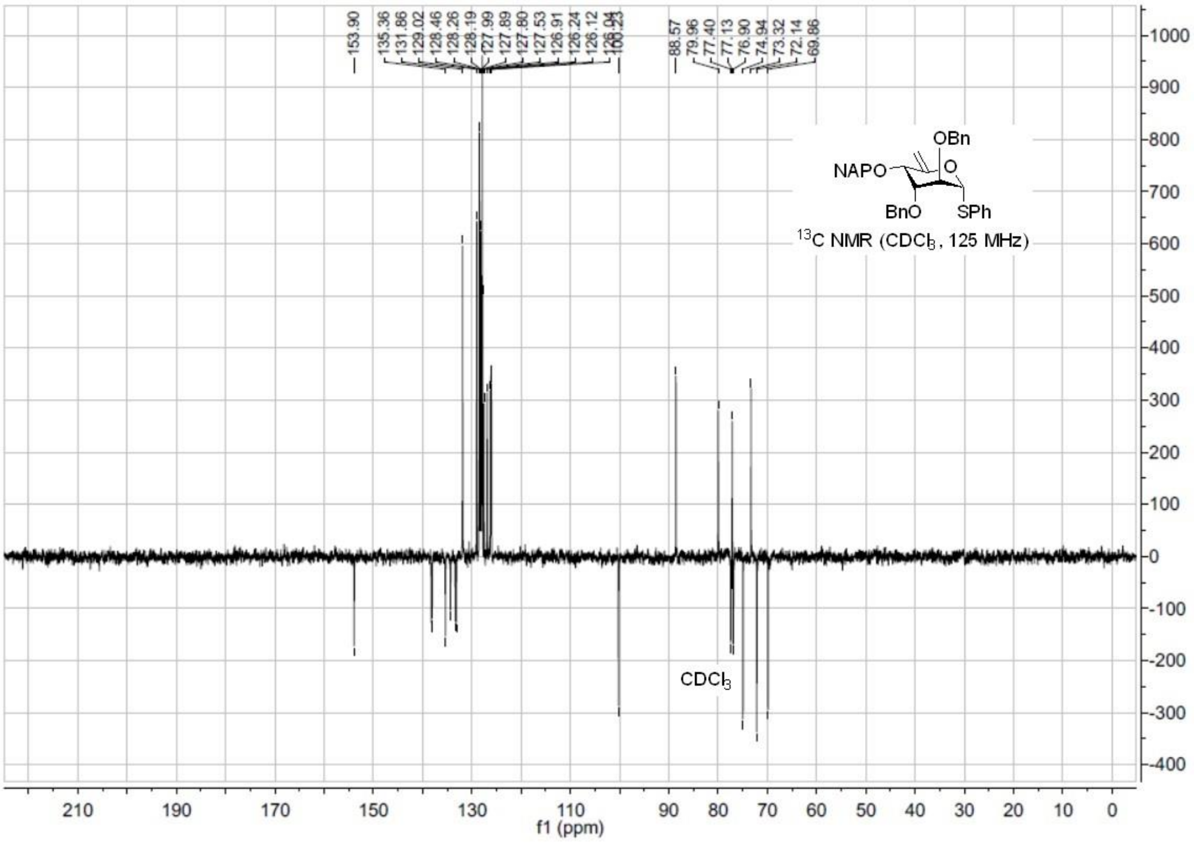

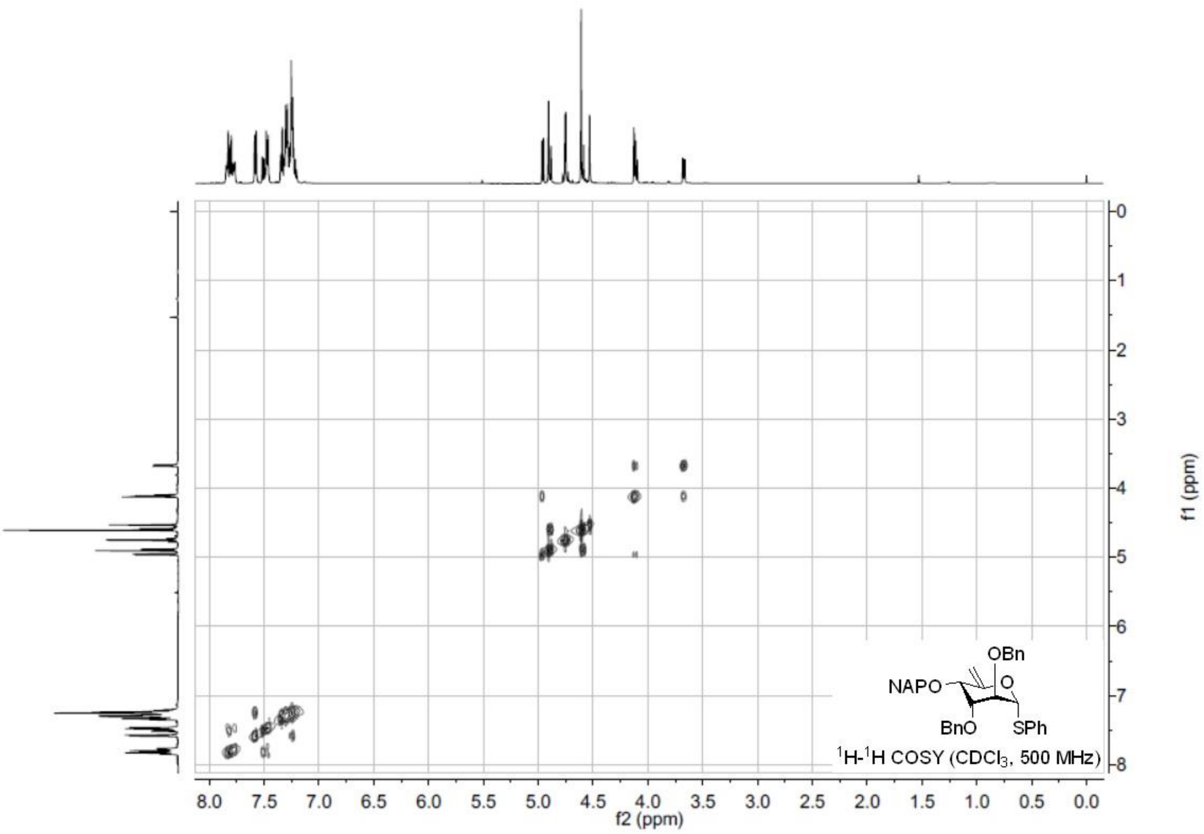
OBn
NAPO
O
BnO
SPh
^{1}H-^{1}H COSY (CDCl$_3$, 500 MHz)
f2 (ppm)
f1 (ppm)

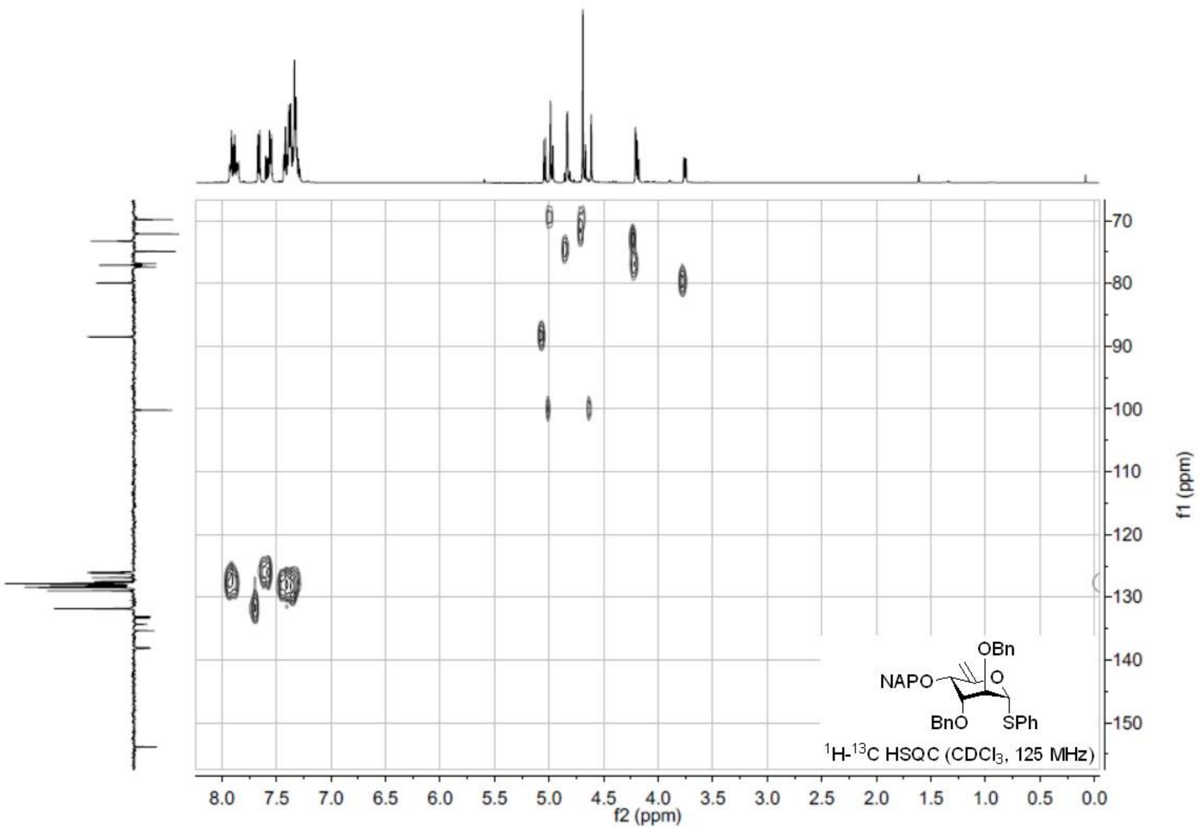
OBn
NAPO
O
BnO
SPh
^{1}H-^{13}C HSQC (CDCl$_3$, 125 MHz)
f2 (ppm)
f1 (ppm)

^{1}H and ^{13}C NMR spectra of compound **75**

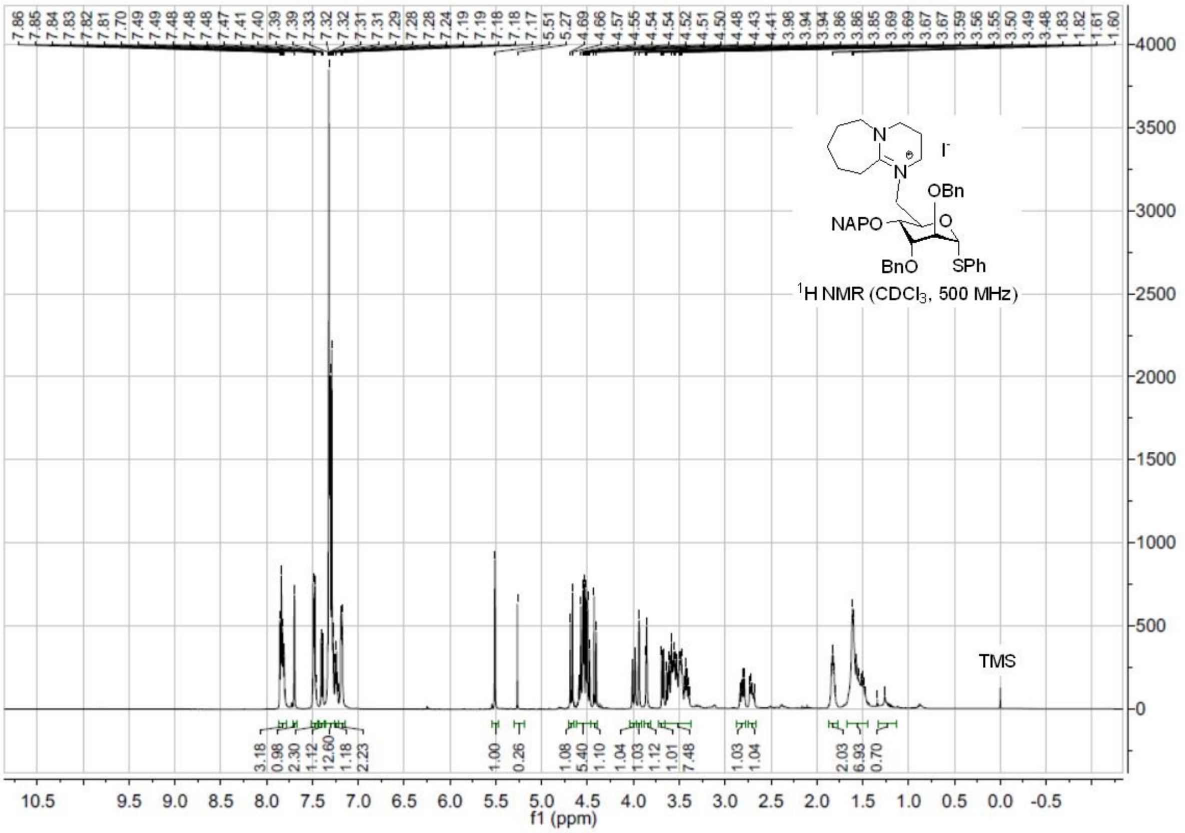

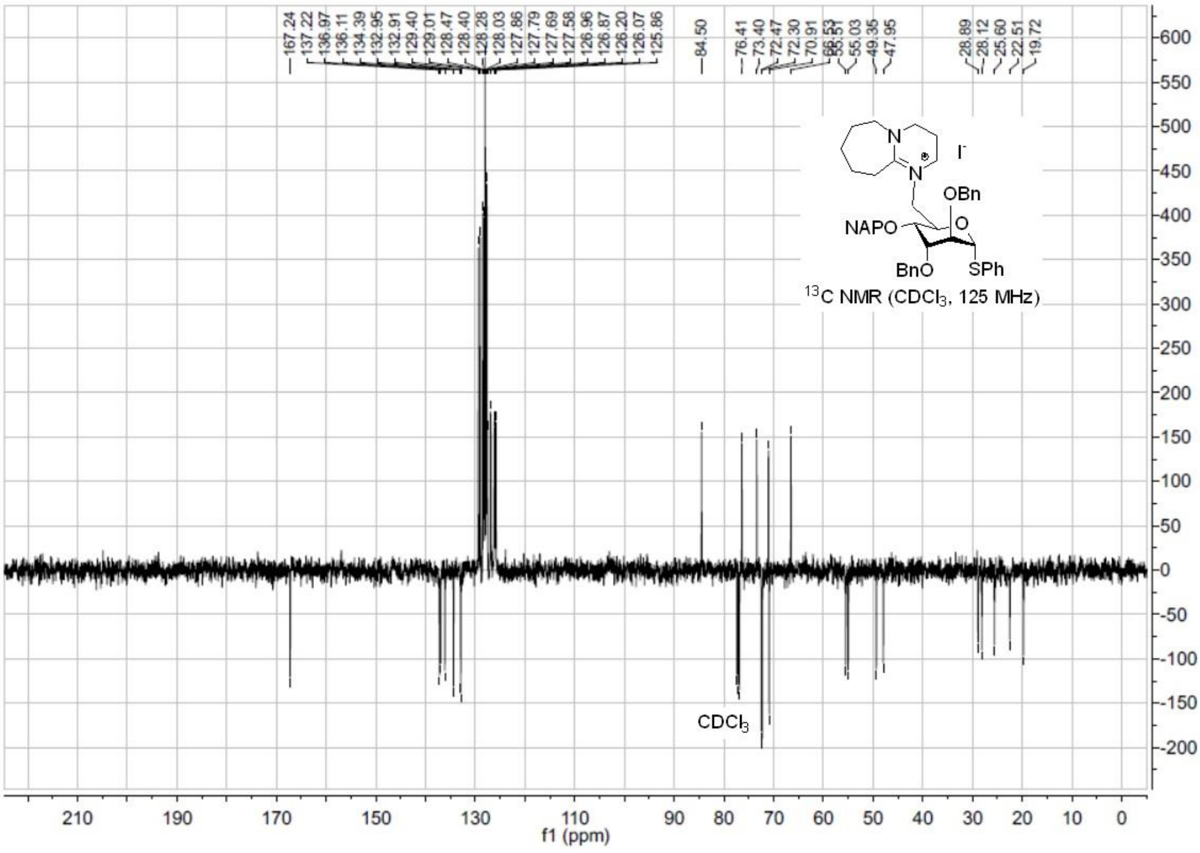

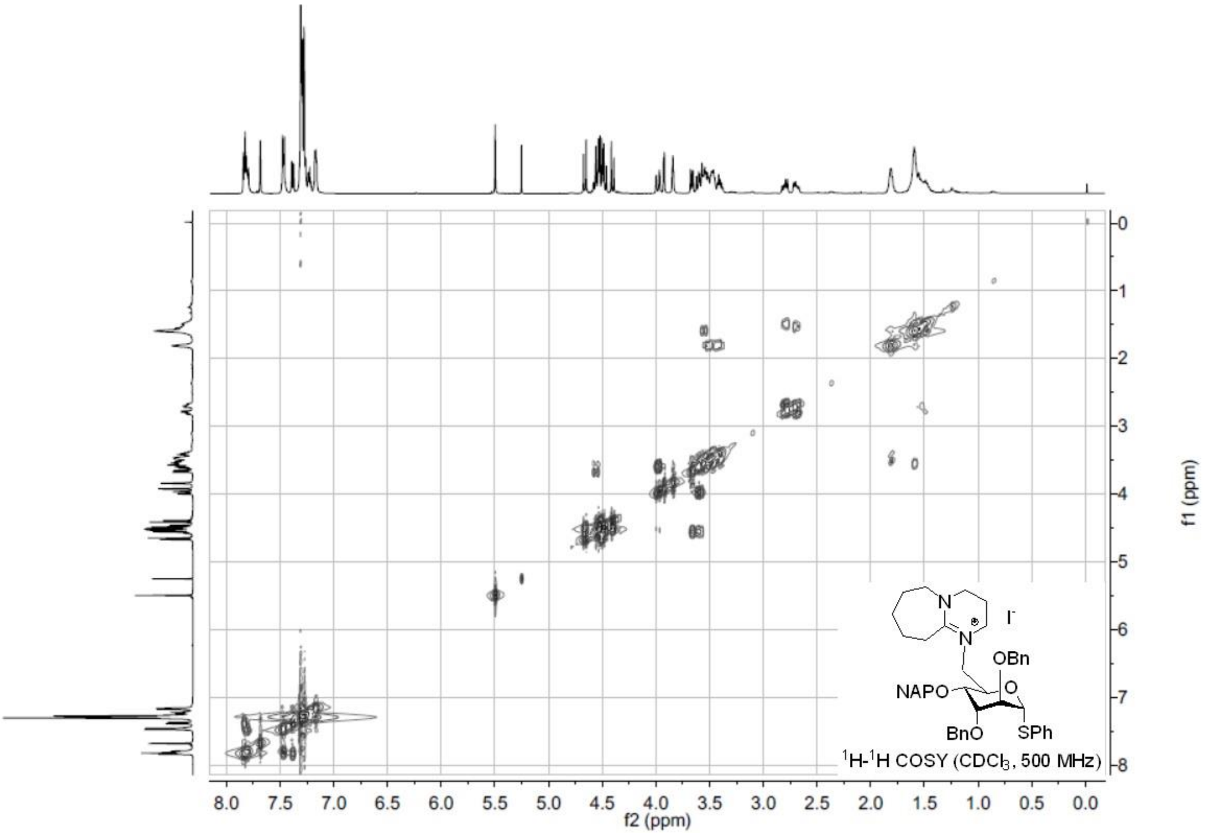

I⁻
OBn
NAPO
BnO
SPh
^{1}H-^{1}H COSY (CDCl$_3$, 500 MHz)
f1 (ppm)
f2 (ppm)

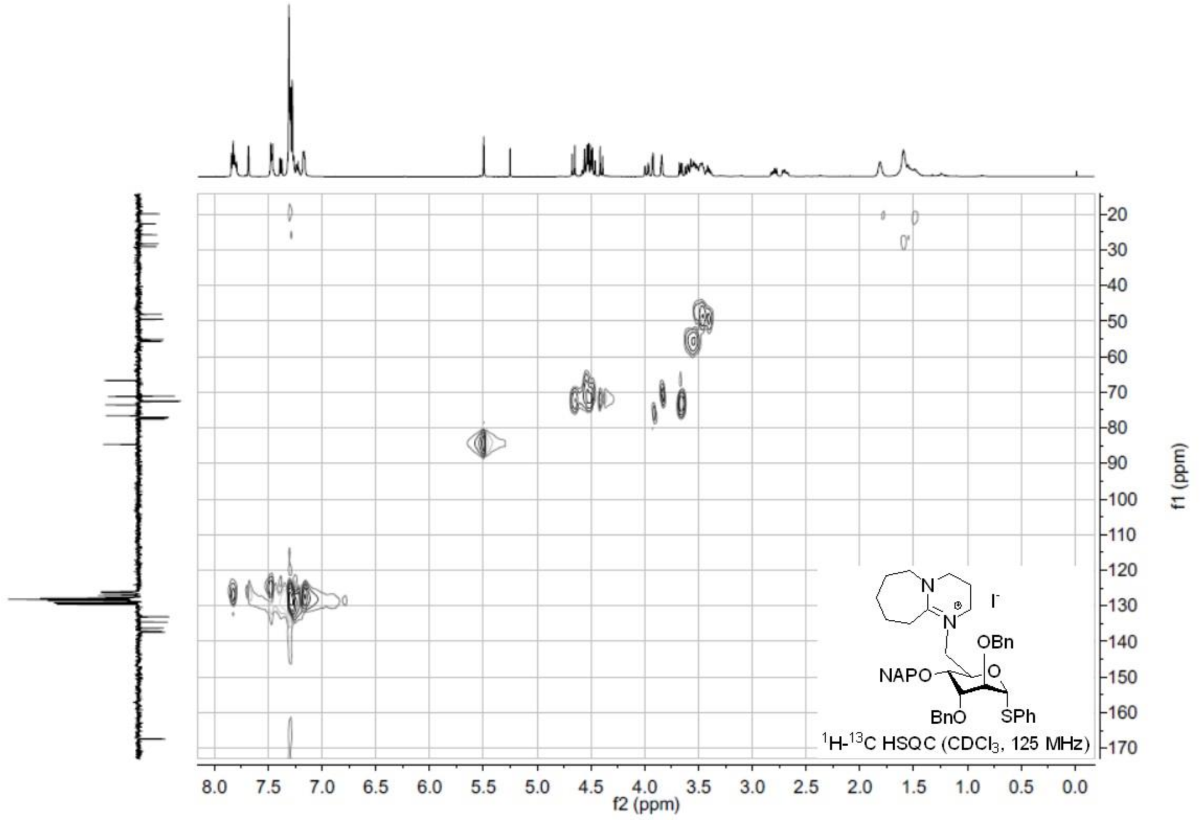

I⁻
OBn
NAPO
BnO
SPh
^{1}H-^{13}C HSQC (CDCl$_3$, 125 MHz)
f1 (ppm)
f2 (ppm)

^{1}H and ^{13}C NMR spectra of compound **76**

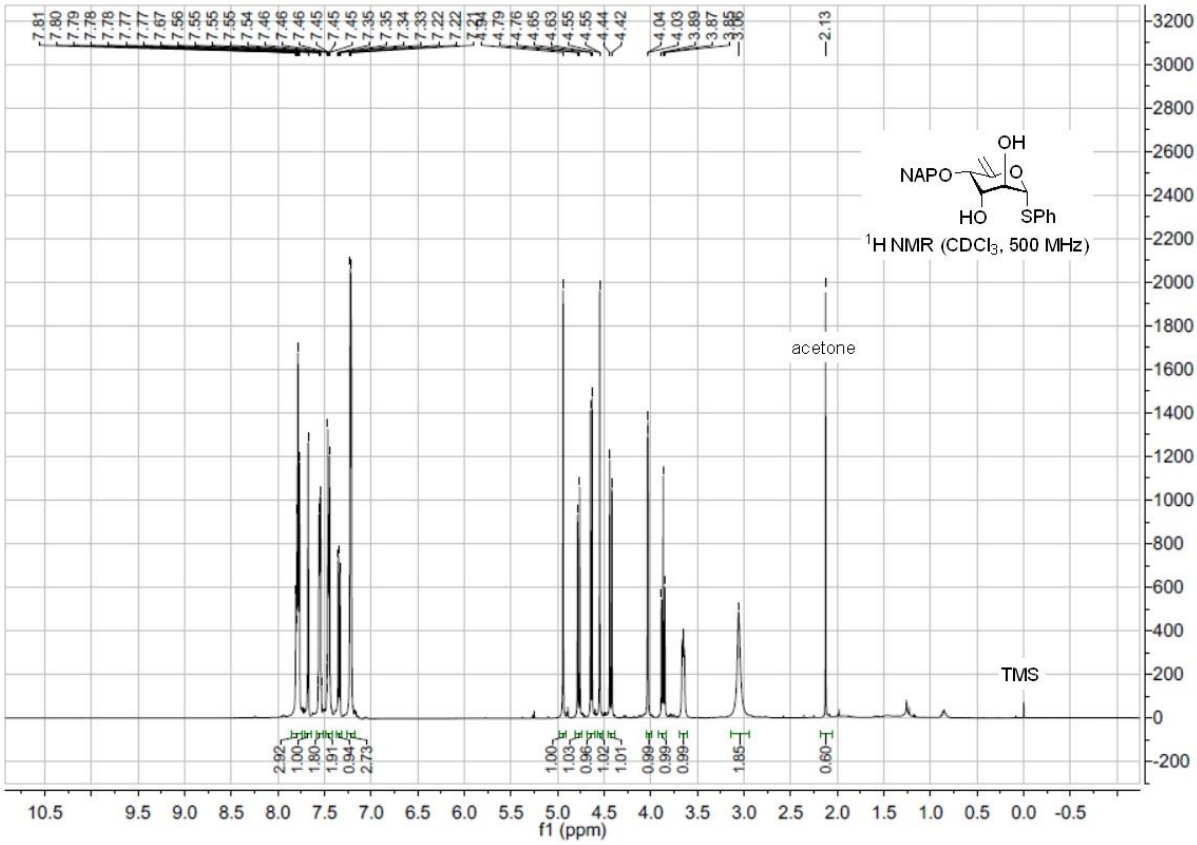

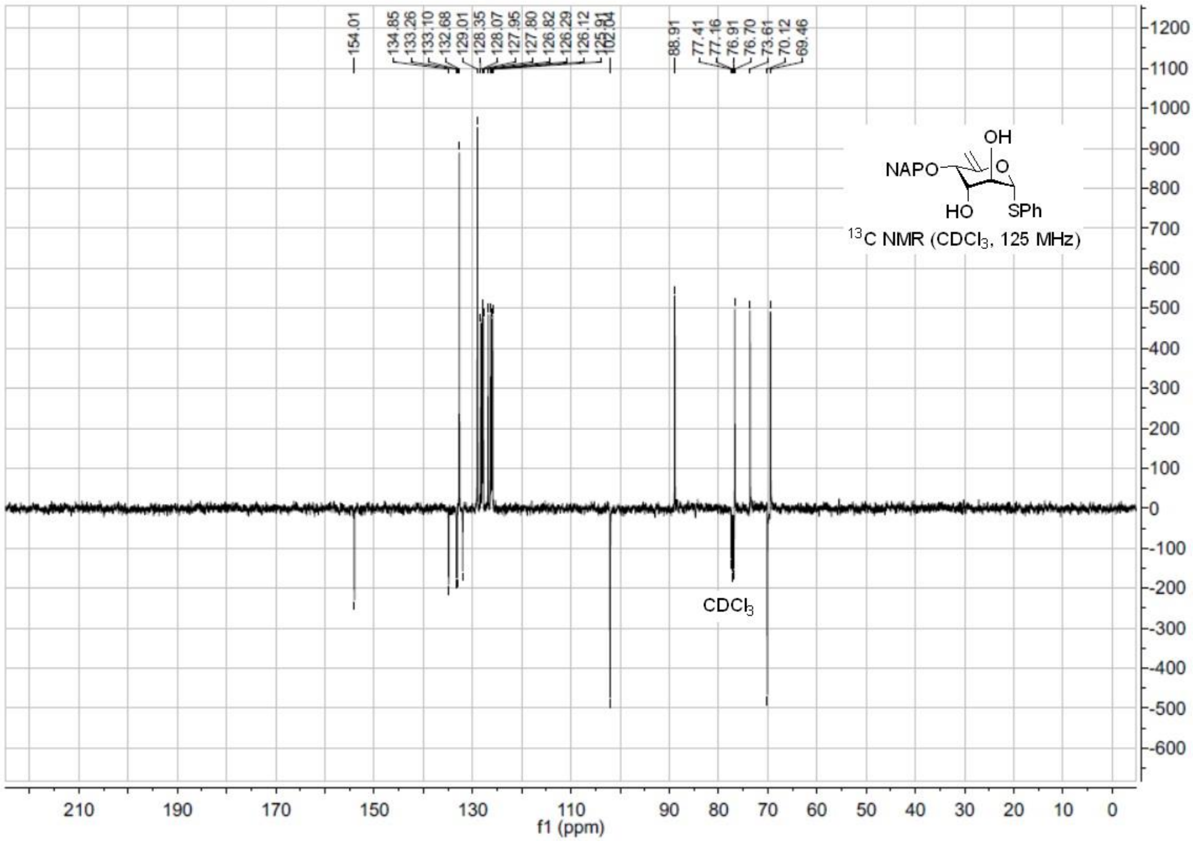

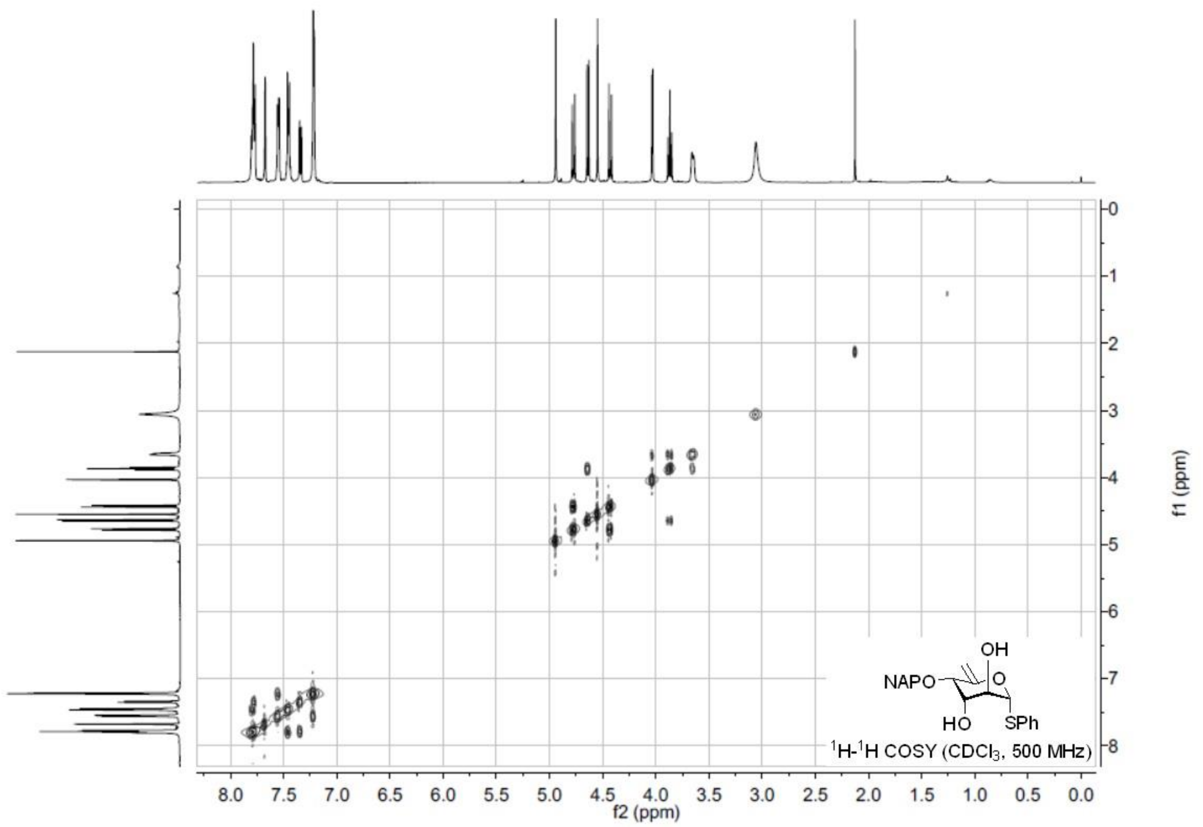
OH
NAPO
O
HO
SPh
1H-1H COSY (CDCl3, 500 MHz)
f2 (ppm)
f1 (ppm)

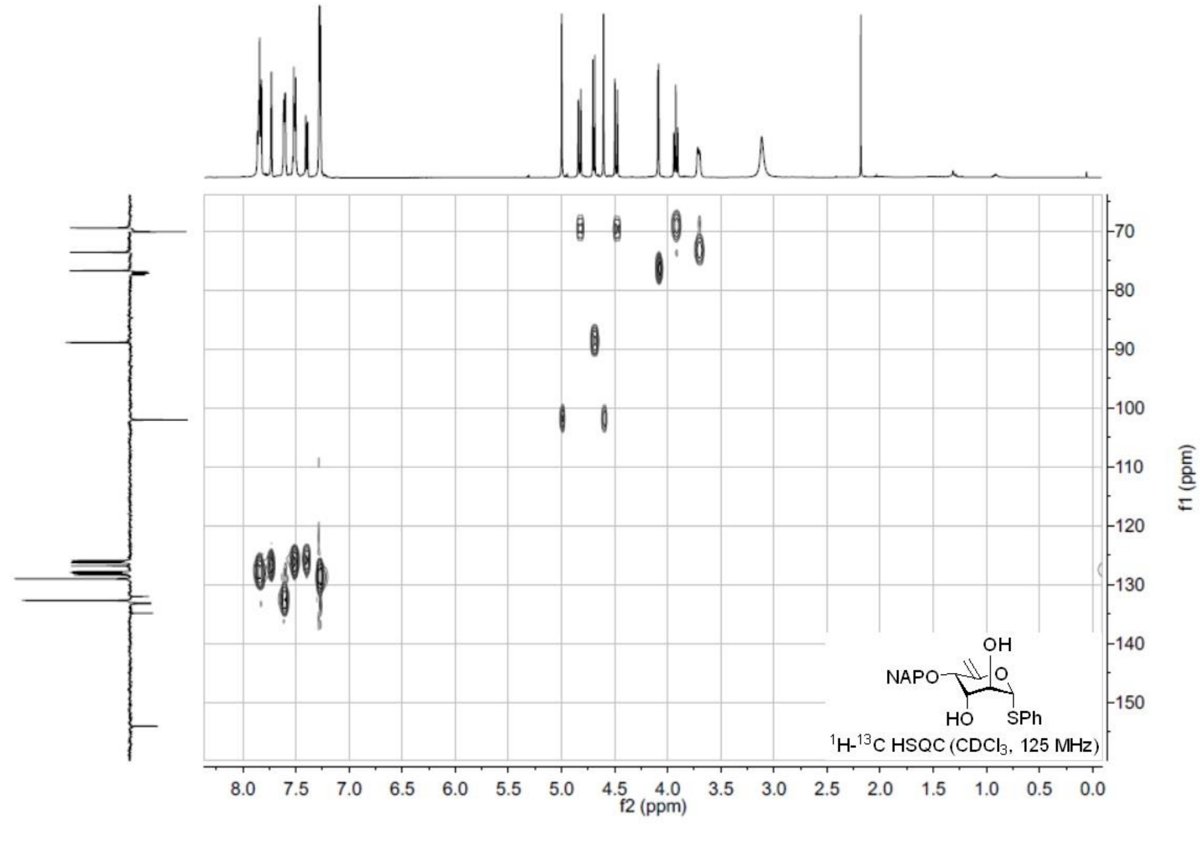
OH
NAPO
O
HO
SPh
1H-13C HSQC (CDCl3, 125 MHz)
f2 (ppm)
f1 (ppm)

^{1}H and ^{13}C NMR spectra of compound **77**

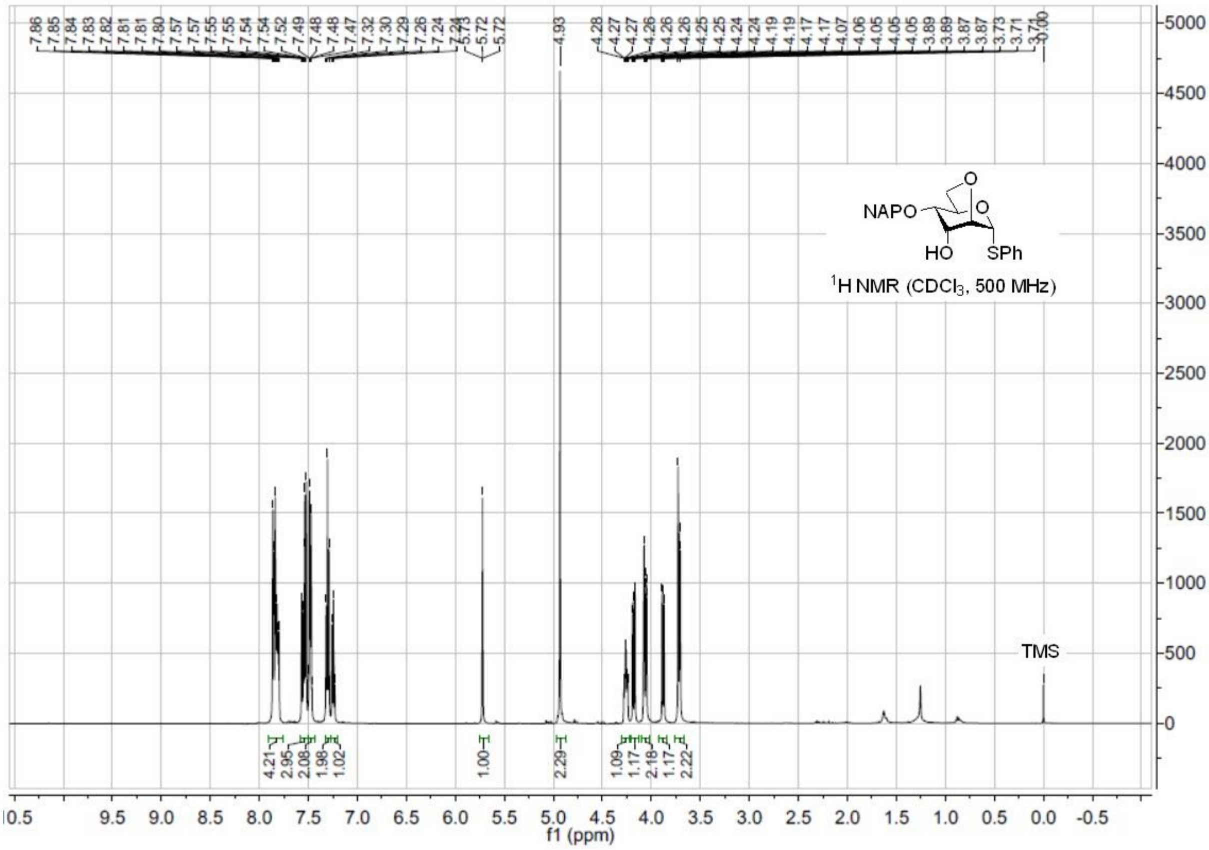

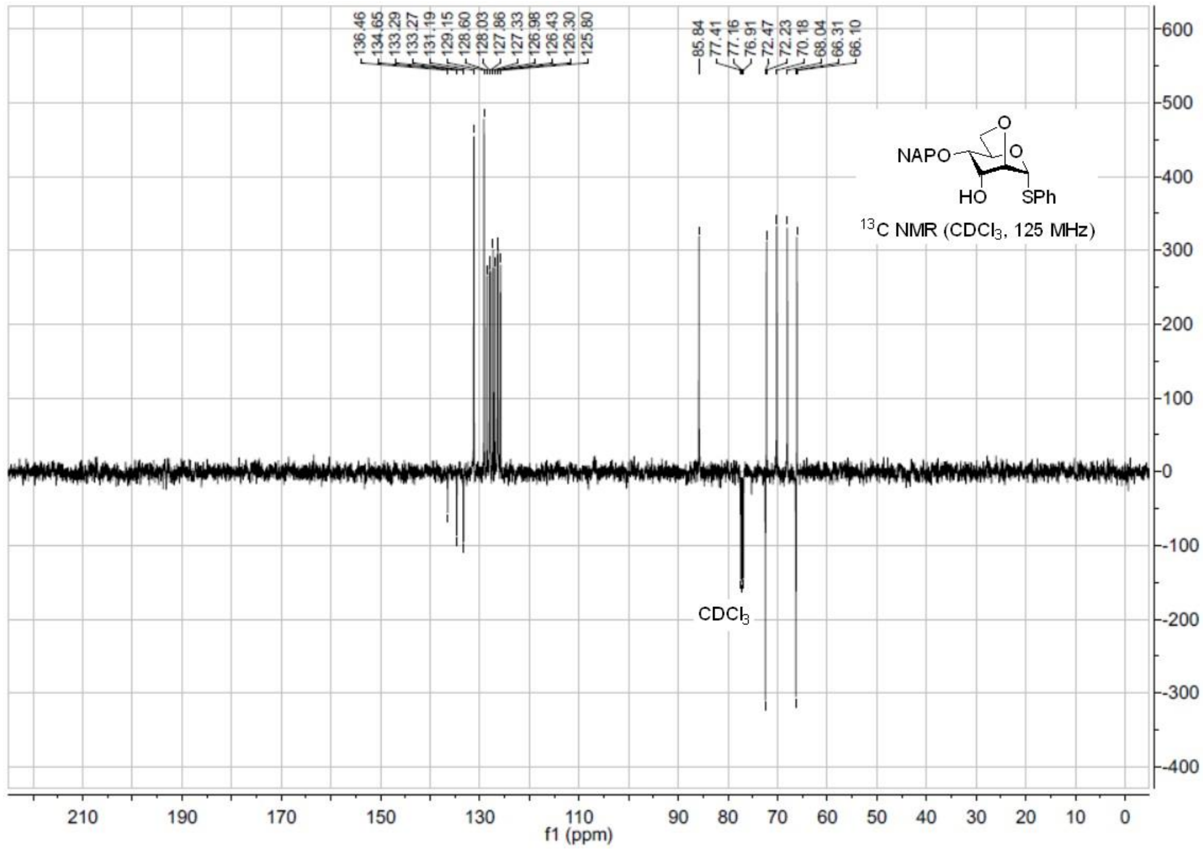

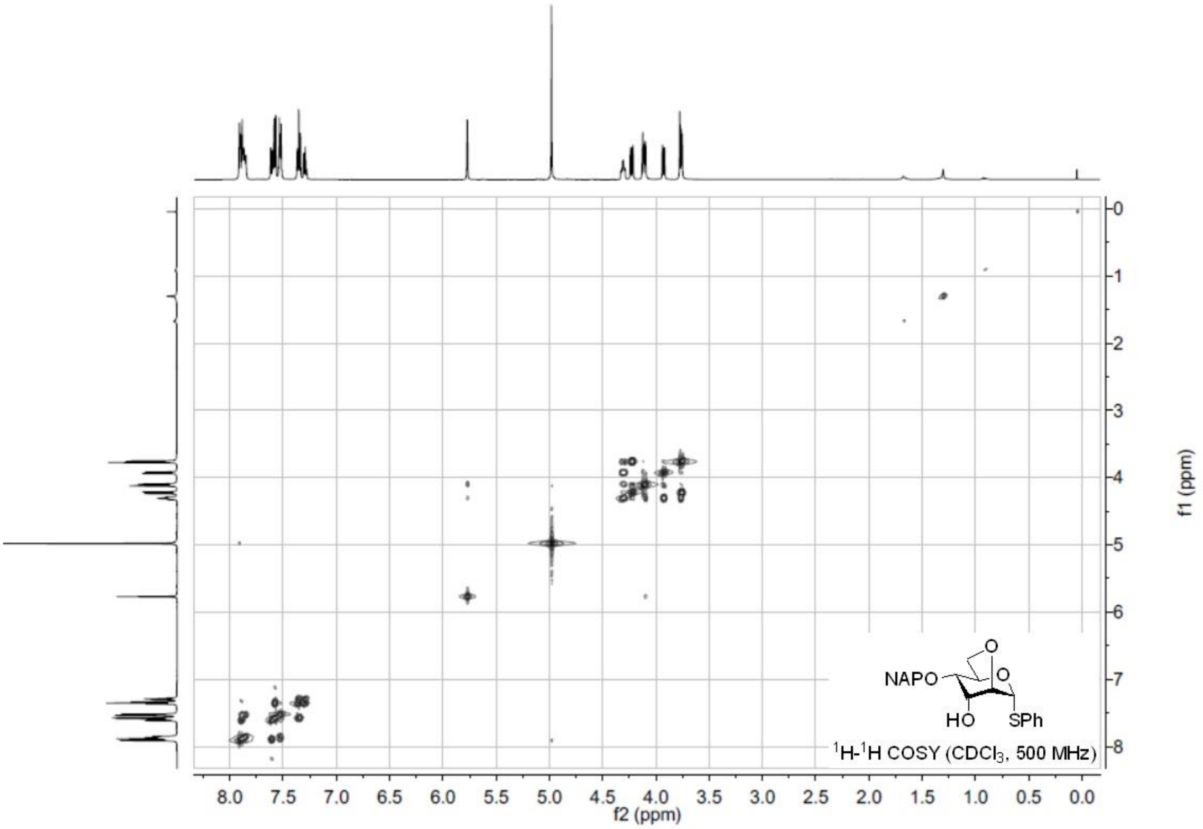
NAPO
O
O
HO
SPh
^{1}H-^{1}H COSY (CDCl$_3$, 500 MHz)
f2 (ppm)
f1 (ppm)

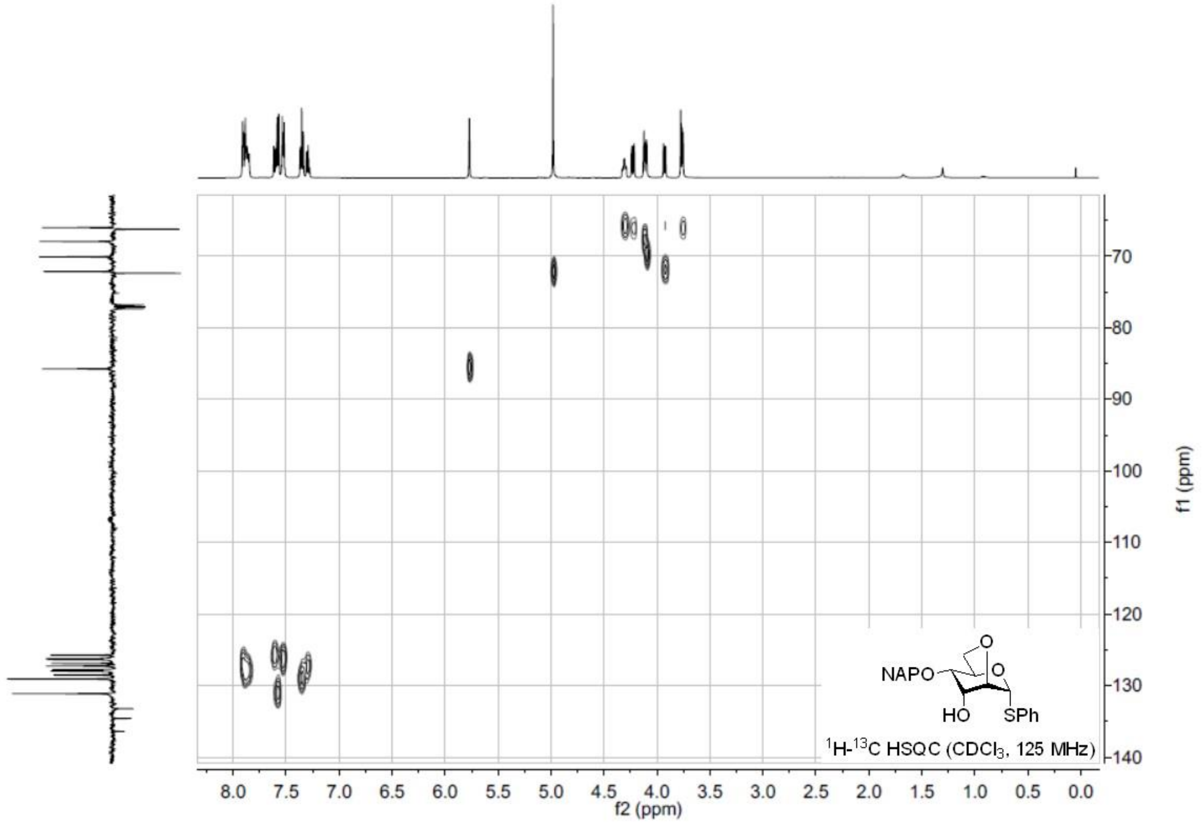
NAPO
O
O
HO
SPh
^{1}H-^{13}C HSQC (CDCl$_3$, 125 MHz)
f2 (ppm)
f1 (ppm)

^{1}H and ^{13}C NMR spectra of compound **78**

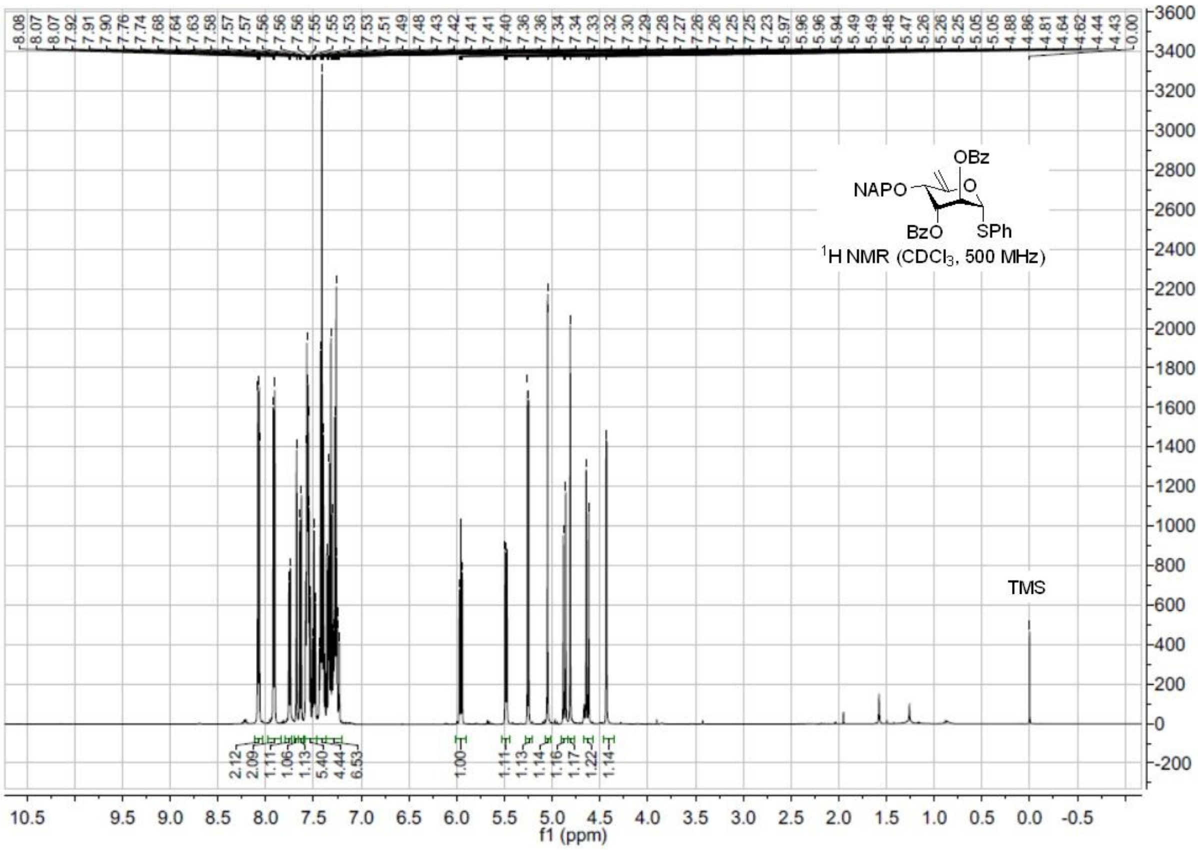

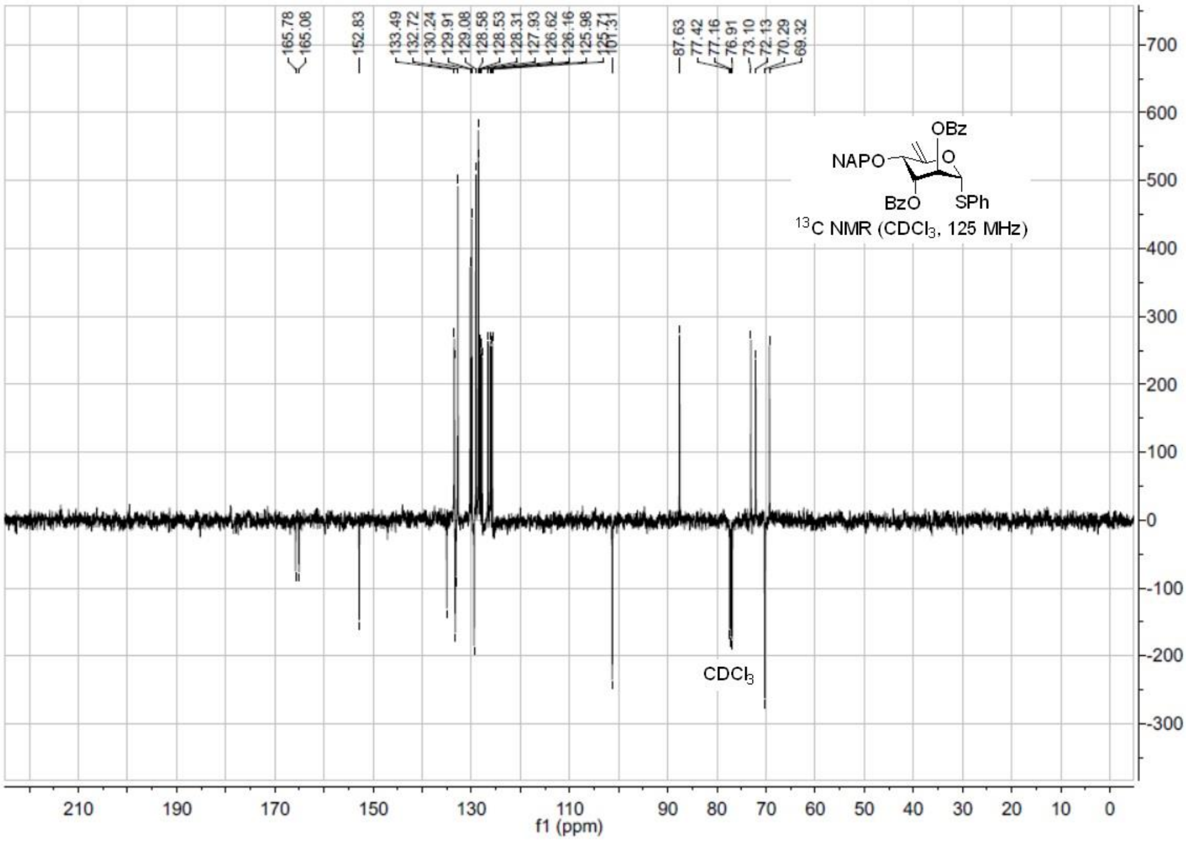

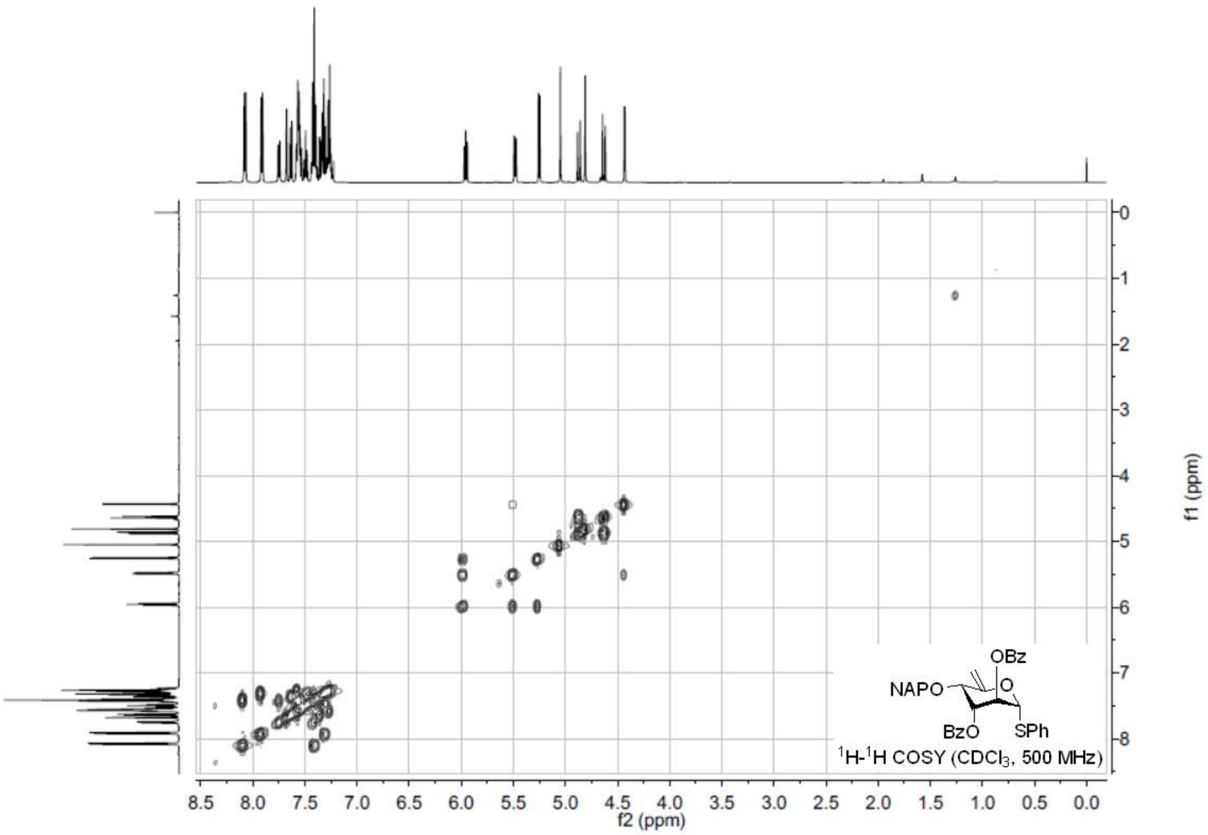
OBz
NAPO
O
BzO
SPh
1H-1H COSY (CDCl3, 500 MHz)
f1 (ppm)
f2 (ppm)

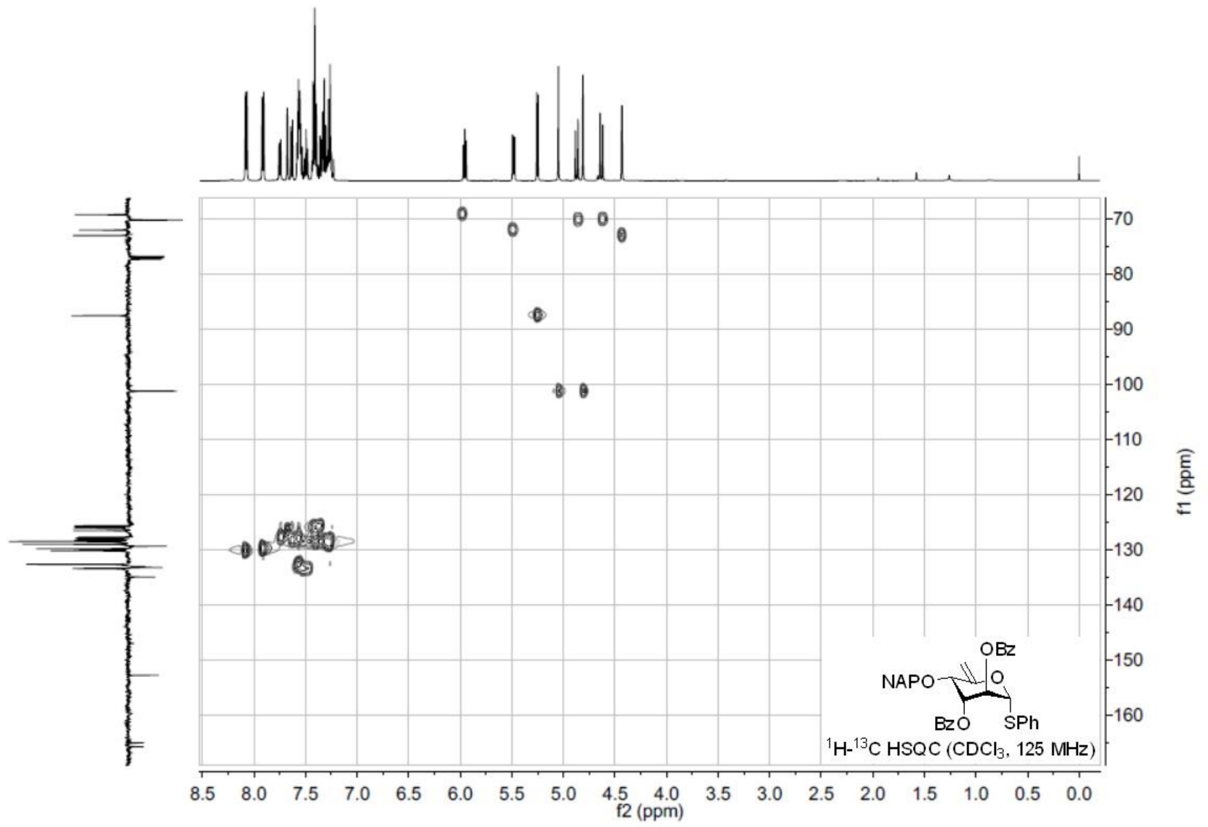
OBz
NAPO
O
BzO
SPh
1H-13C HSQC (CDCl3, 125 MHz)
f1 (ppm)
f2 (ppm)

^{1}H and ^{13}C NMR spectra of compound **79**

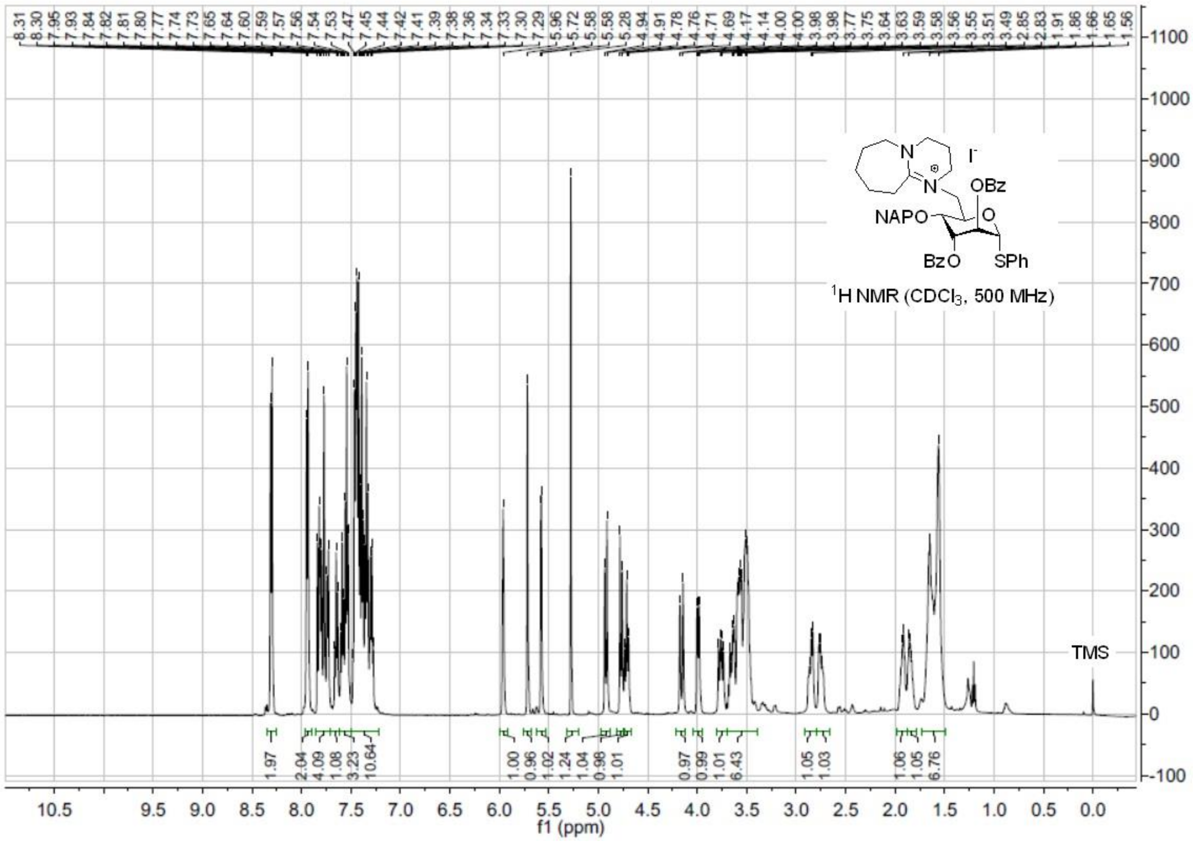

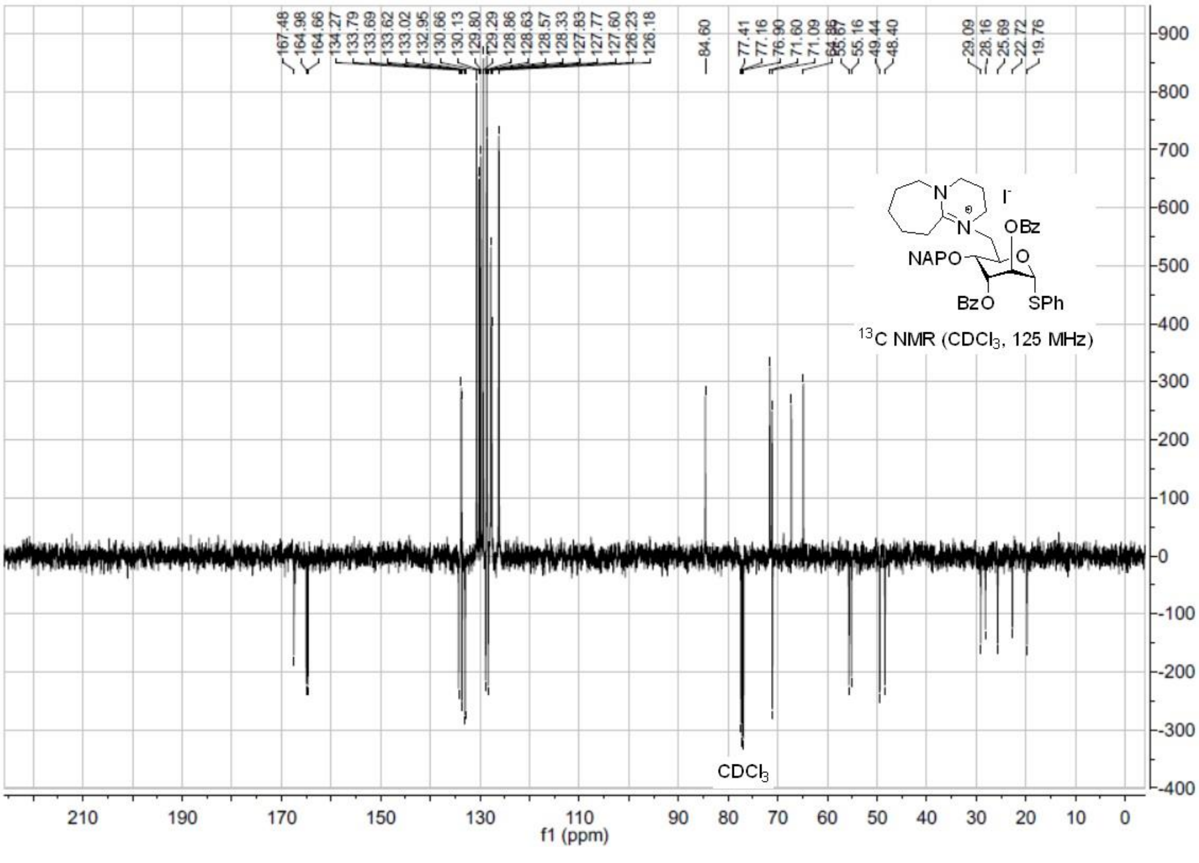

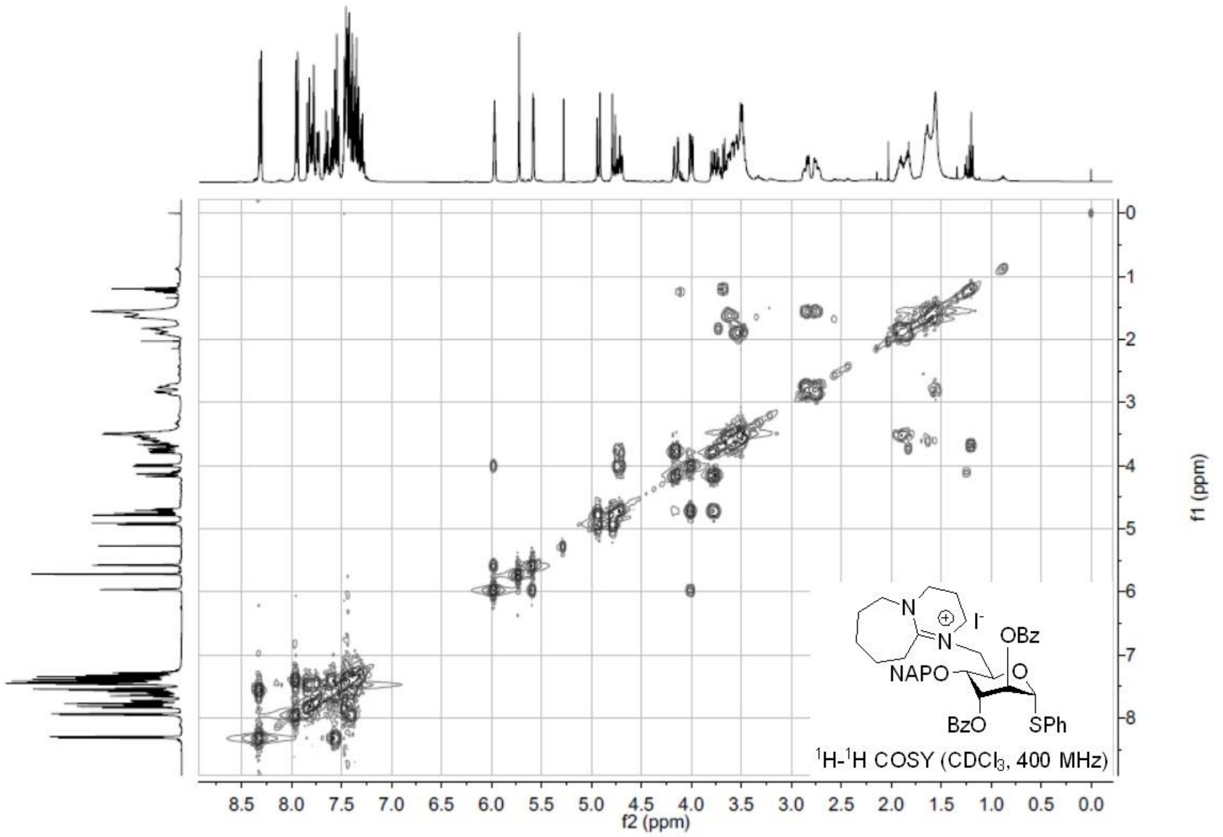

f1 (ppm)
f2 (ppm)
NAPO
BzO
OBz
SPh
I⁻
^{1}H-^{1}H COSY ($CDCl_3$, 400 MHz)

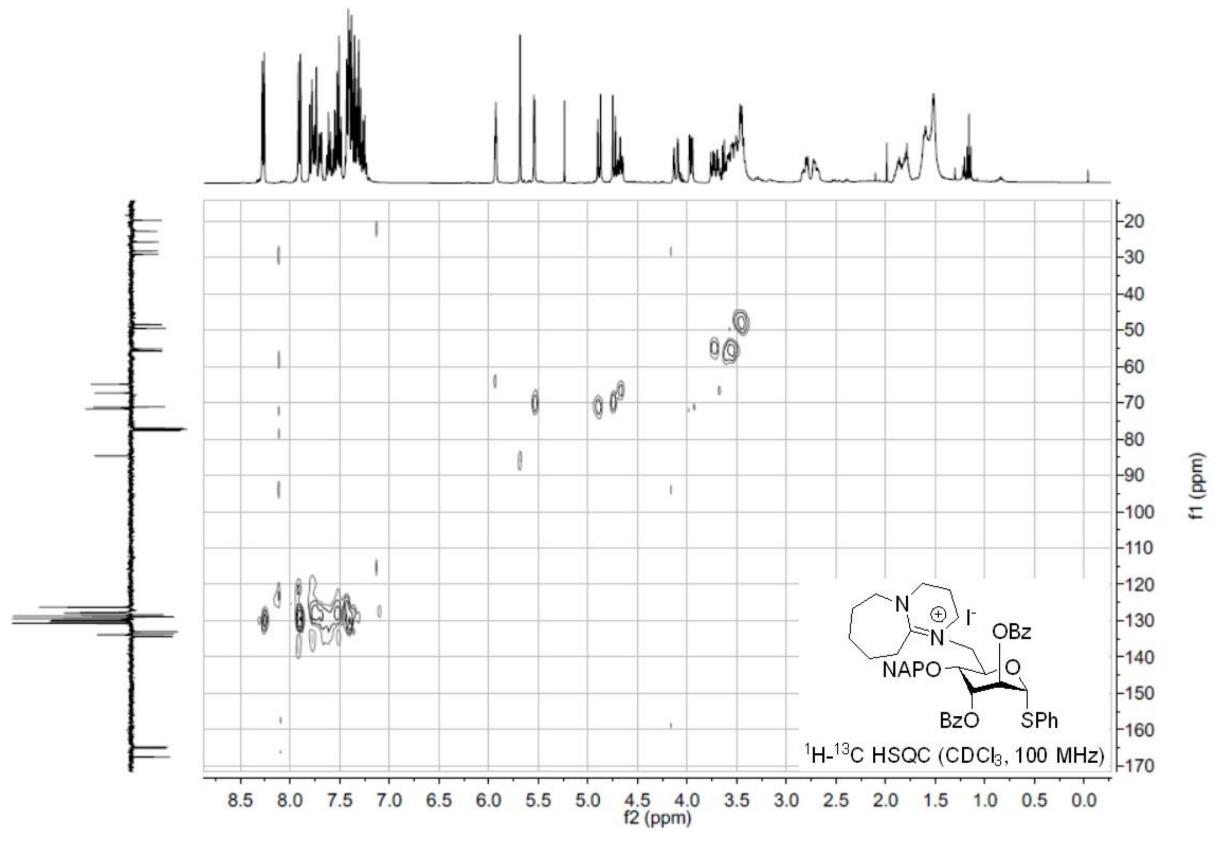

f1 (ppm)
f2 (ppm)
NAPO
BzO
OBz
SPh
I⁻
^{1}H-^{13}C HSQC ($CDCl_3$, 100 MHz)

^{1}H and ^{13}C NMR spectra of compound **80**

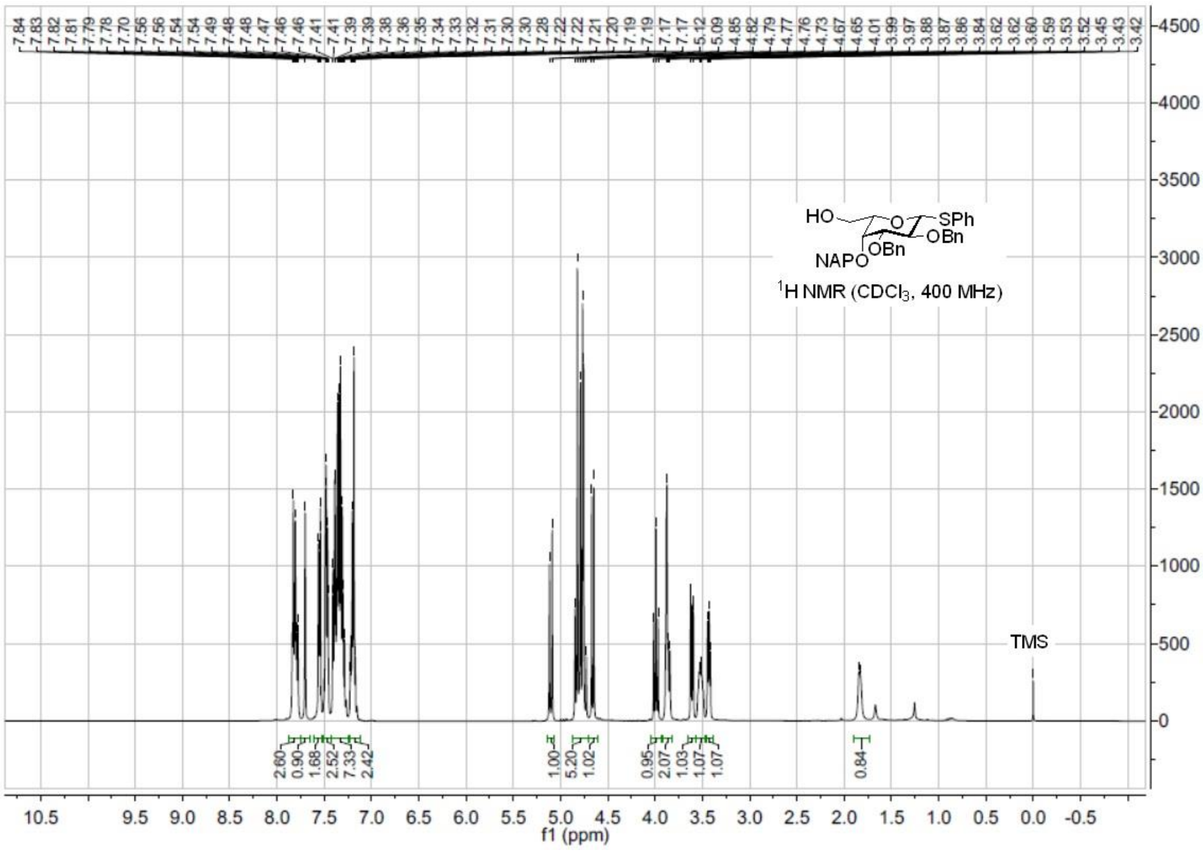

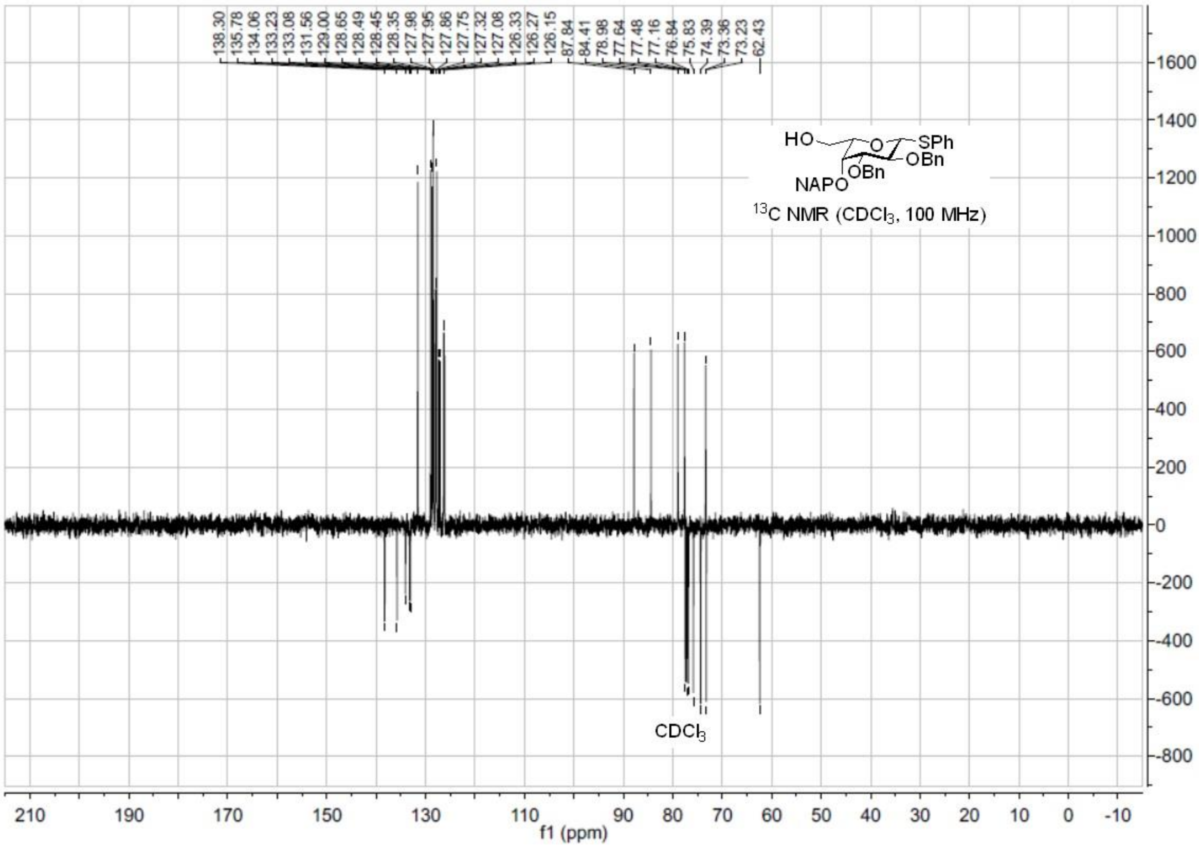

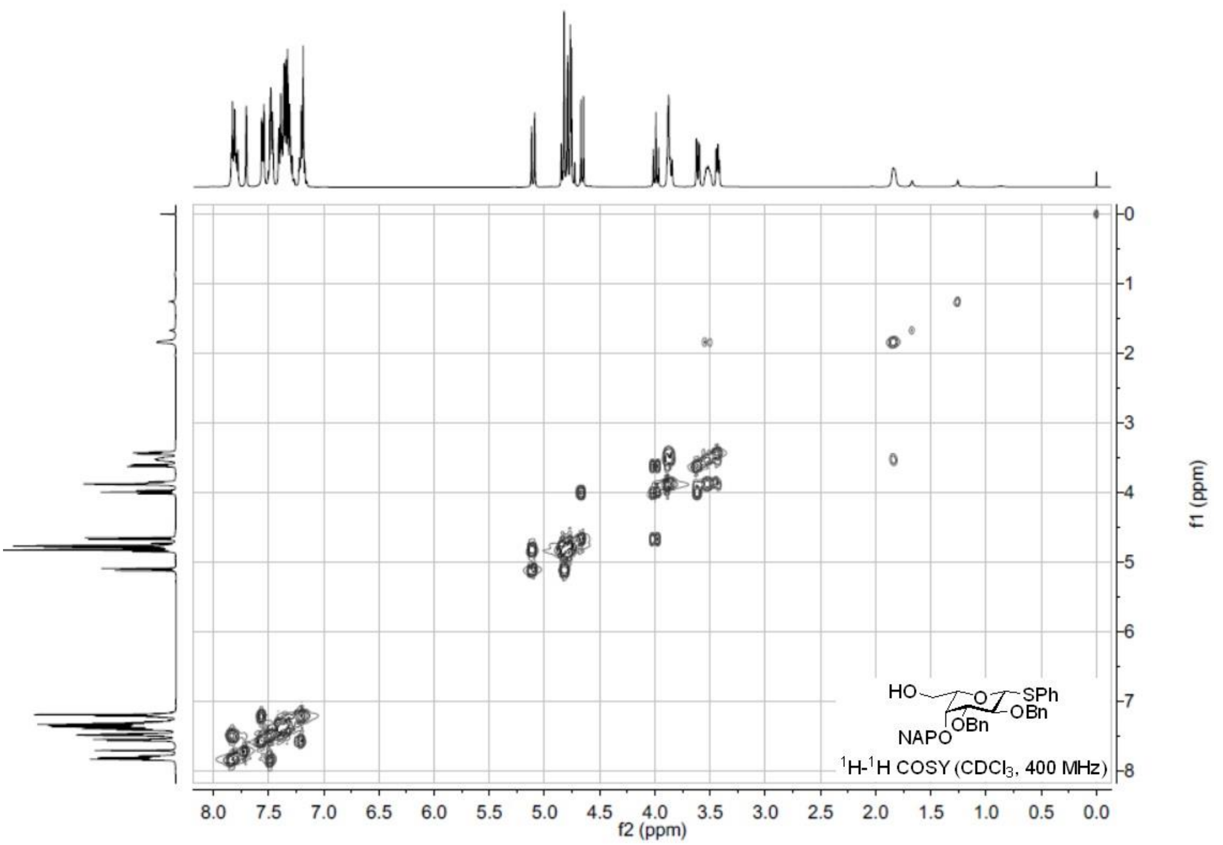
HO
O
SPh
OBn
OBn
NAPO
^{1}H-^{1}H COSY (CDCl$_3$, 400 MHz)
f2 (ppm)
f1 (ppm)

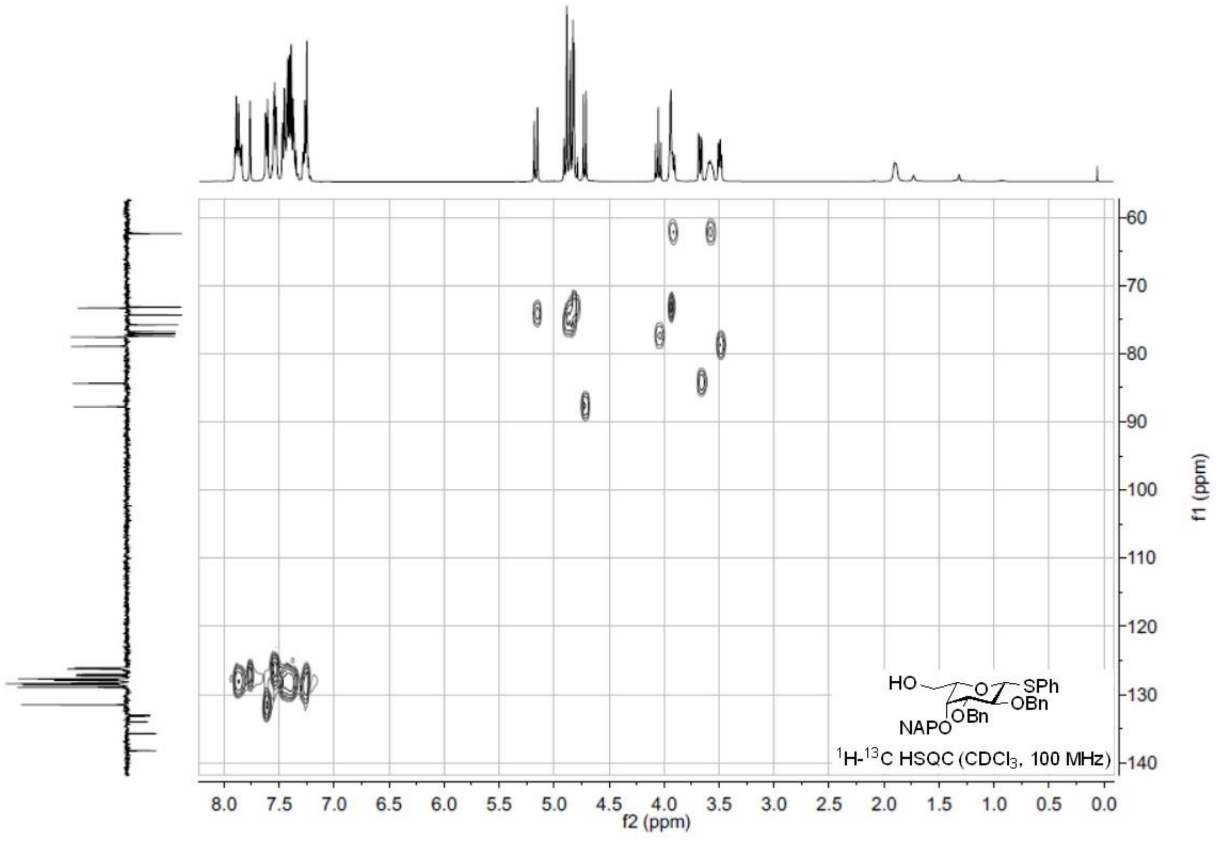
HO
O
SPh
OBn
OBn
NAPO
^{1}H-^{13}C HSQC (CDCl$_3$, 100 MHz)
f2 (ppm)
f1 (ppm)

^{1}H and ^{13}C NMR spectra of compound **81**

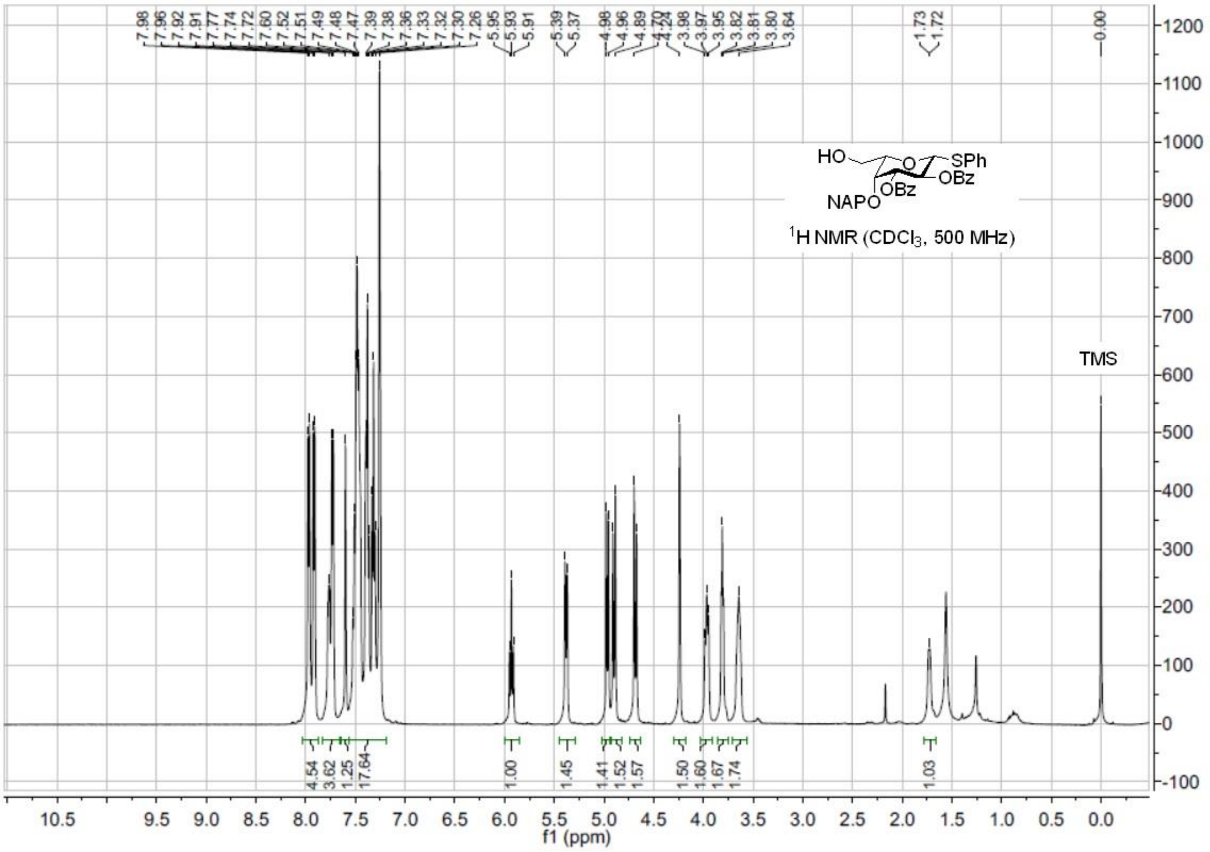

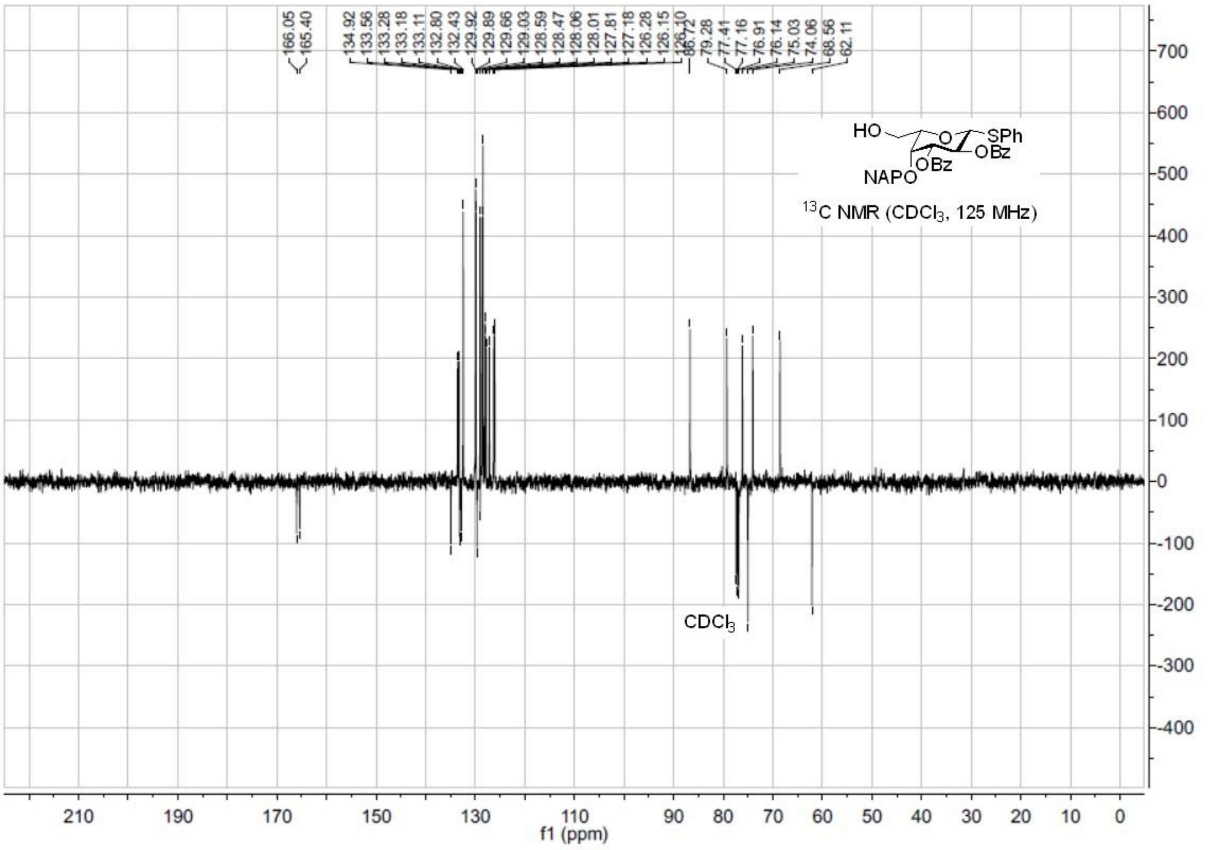

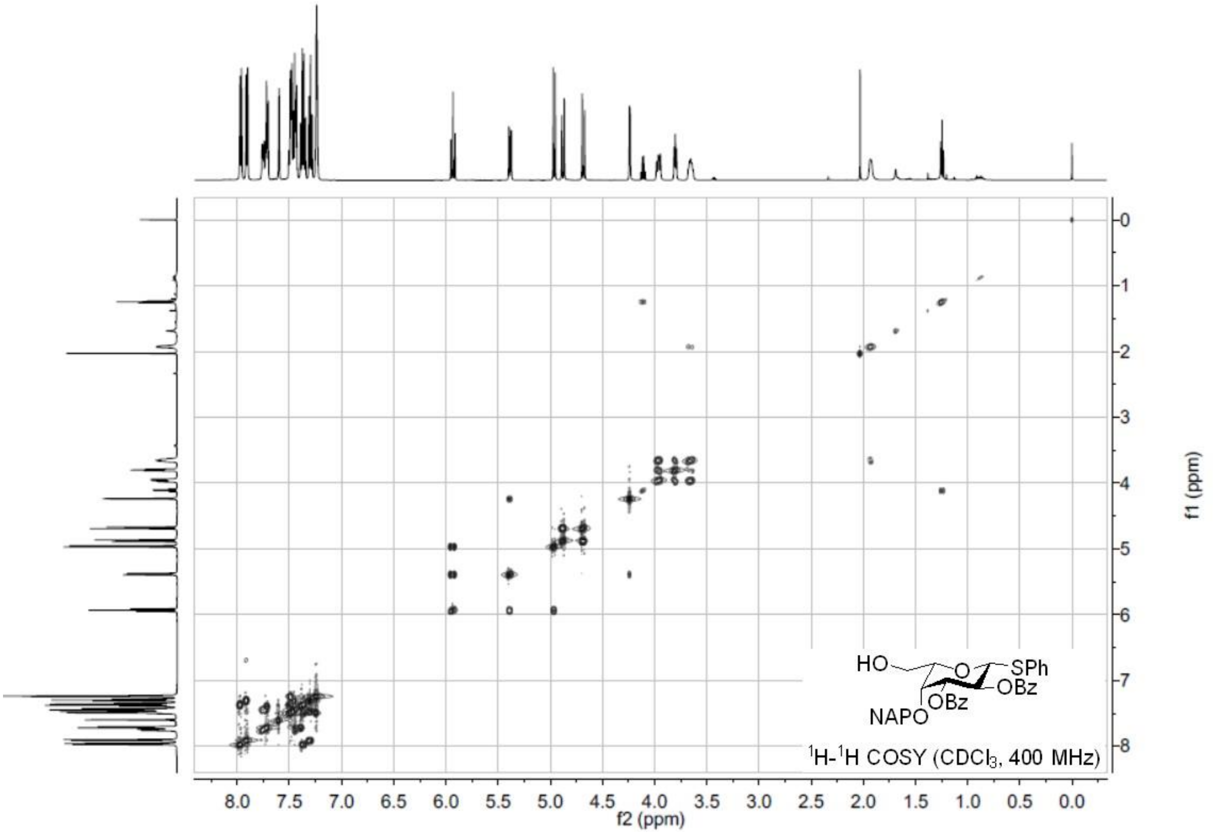
HO
O
SPh
OBz
OBz
NAPO
^{1}H-^{1}H COSY (CDCl$_3$, 400 MHz)
f2 (ppm)
f1 (ppm)

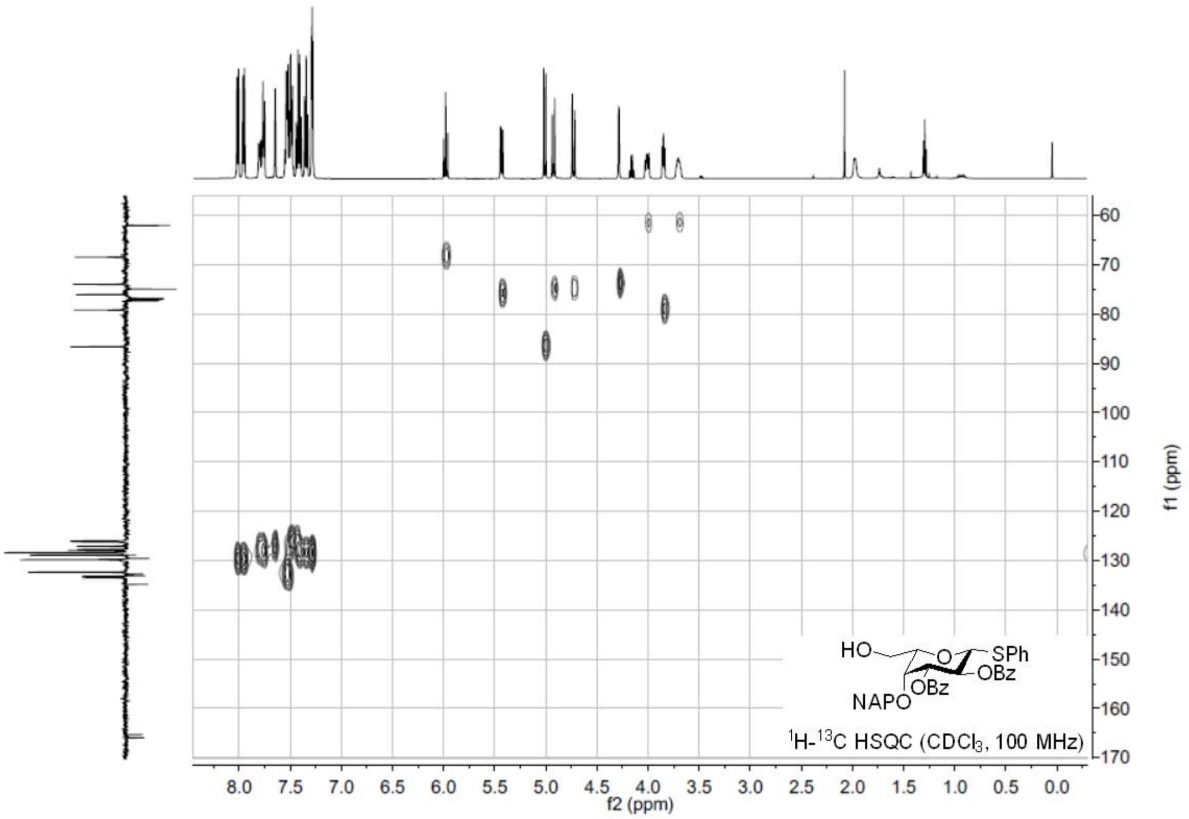
HO
O
SPh
OBz
OBz
NAPO
^{1}H-^{13}C HSQC (CDCl$_3$, 100 MHz)
f2 (ppm)
f1 (ppm)

^{1}H and ^{13}C NMR spectra of compound **82**

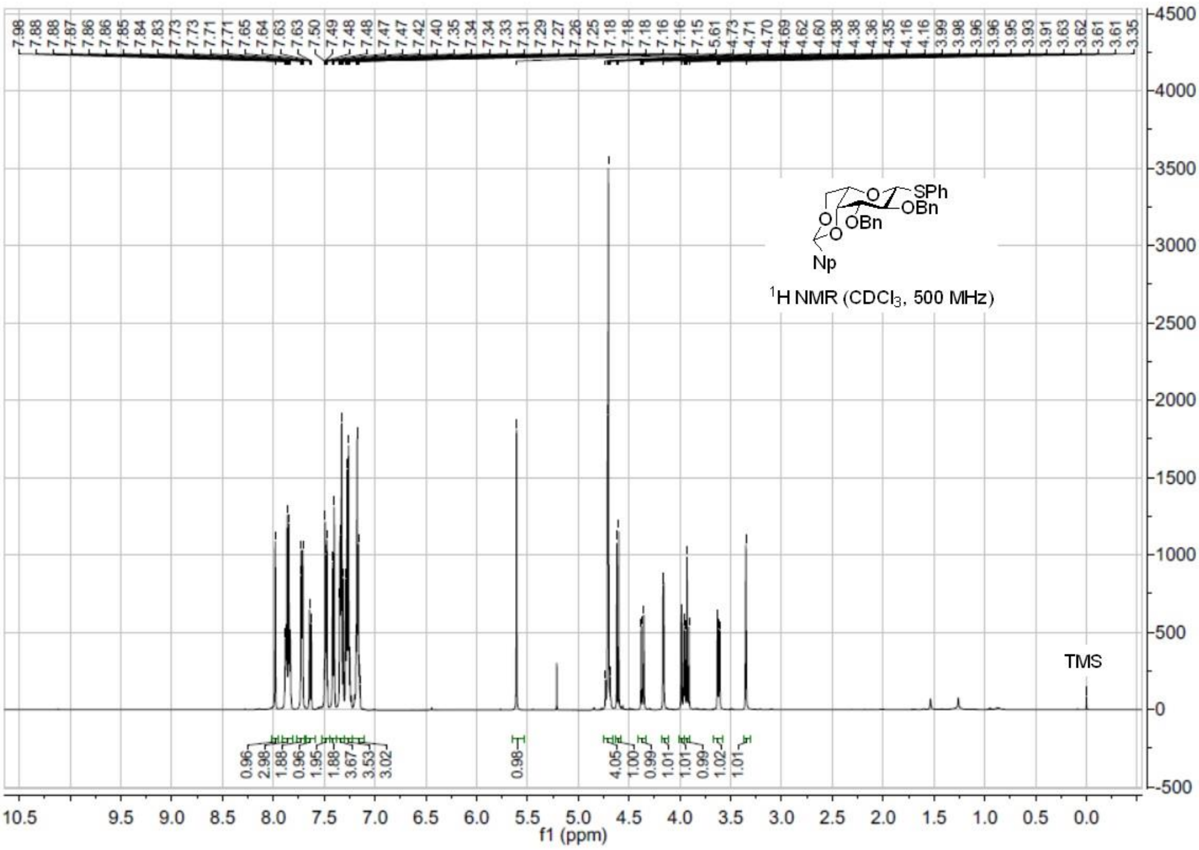

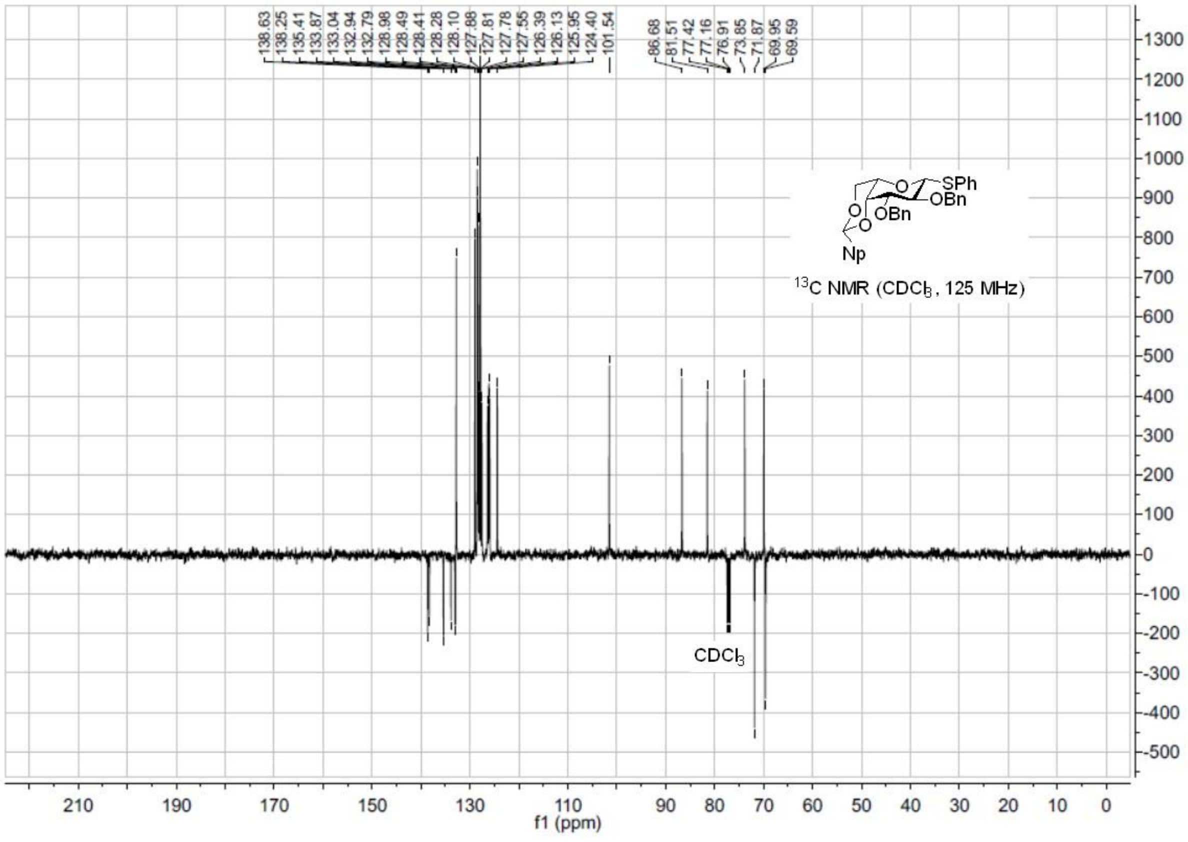

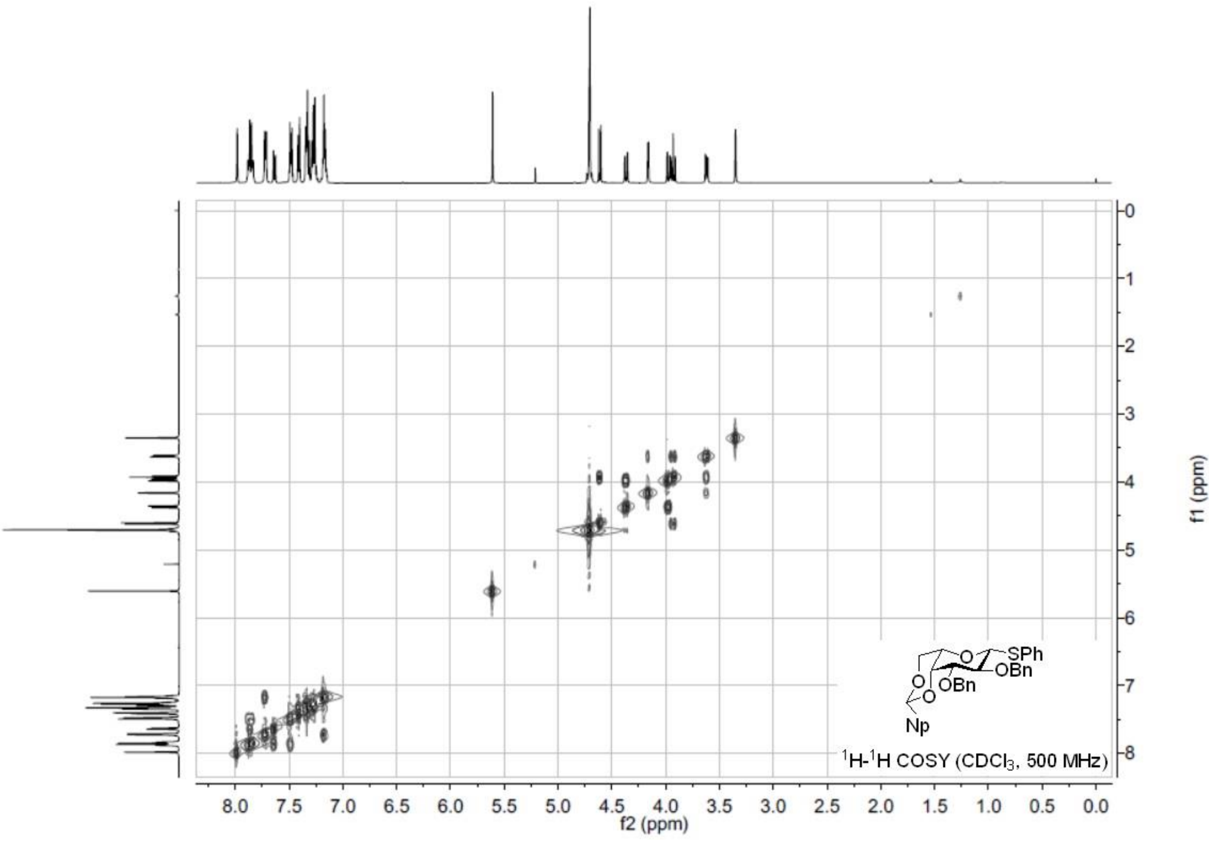
SPh
OBn
OBn
Np
1H-1H COSY (CDCl3, 500 MHz)
f2 (ppm)
f1 (ppm)

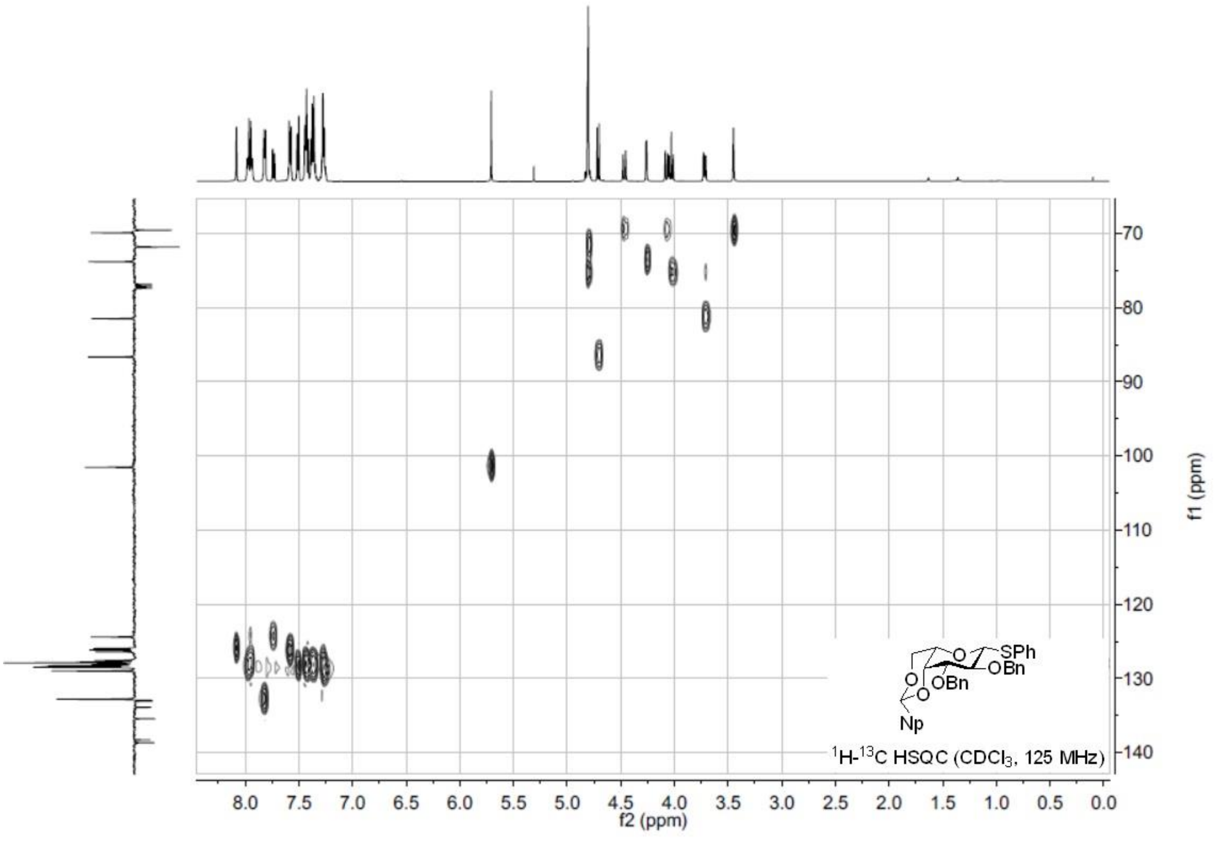
SPh
OBn
OBn
Np
1H-13C HSQC (CDCl3, 125 MHz)
f2 (ppm)
f1 (ppm)

^{1}H and ^{13}C NMR spectra of compound **83**

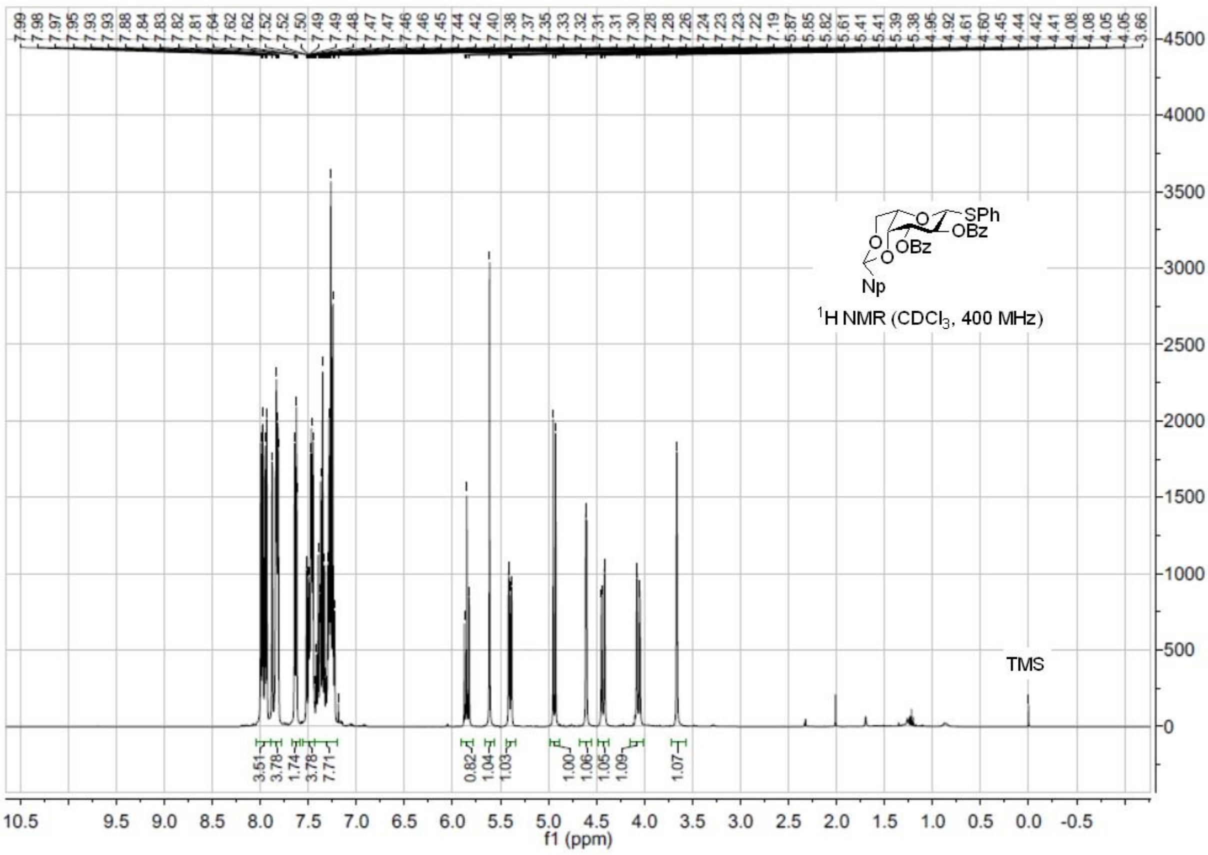

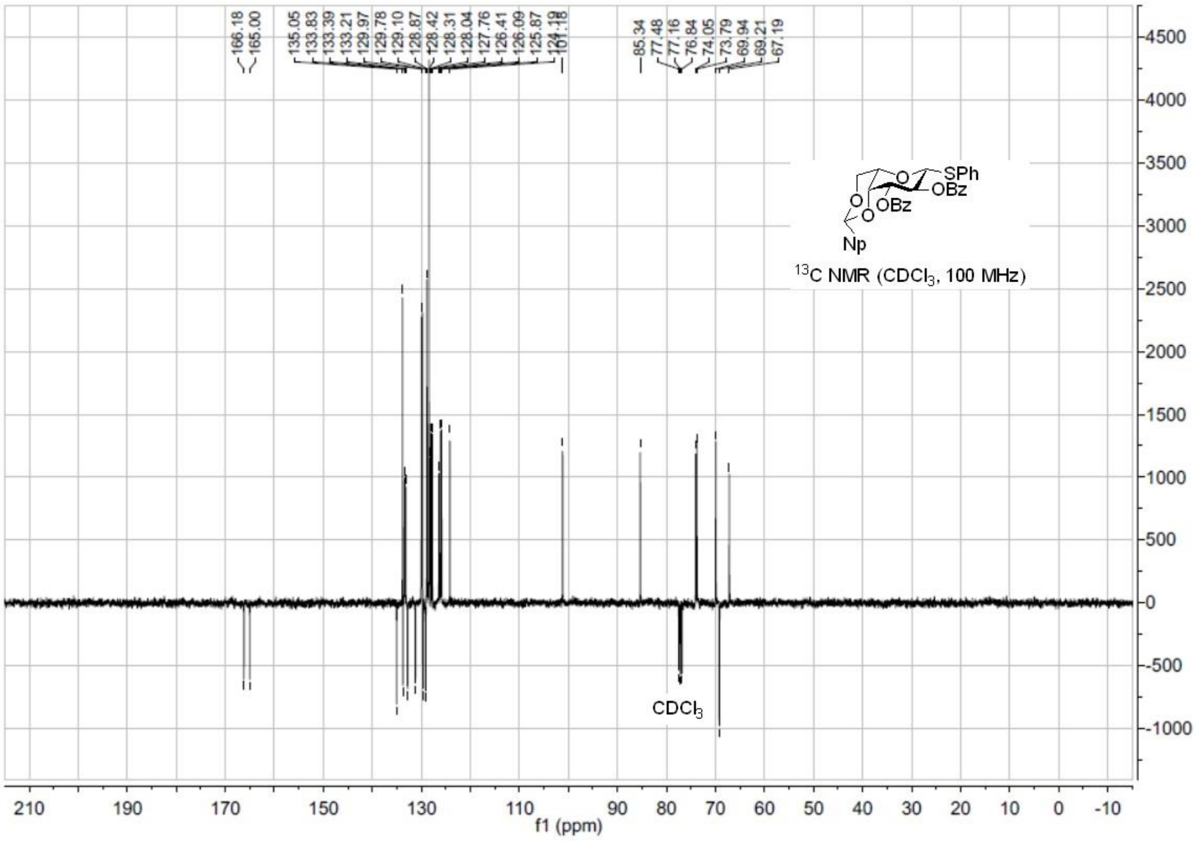

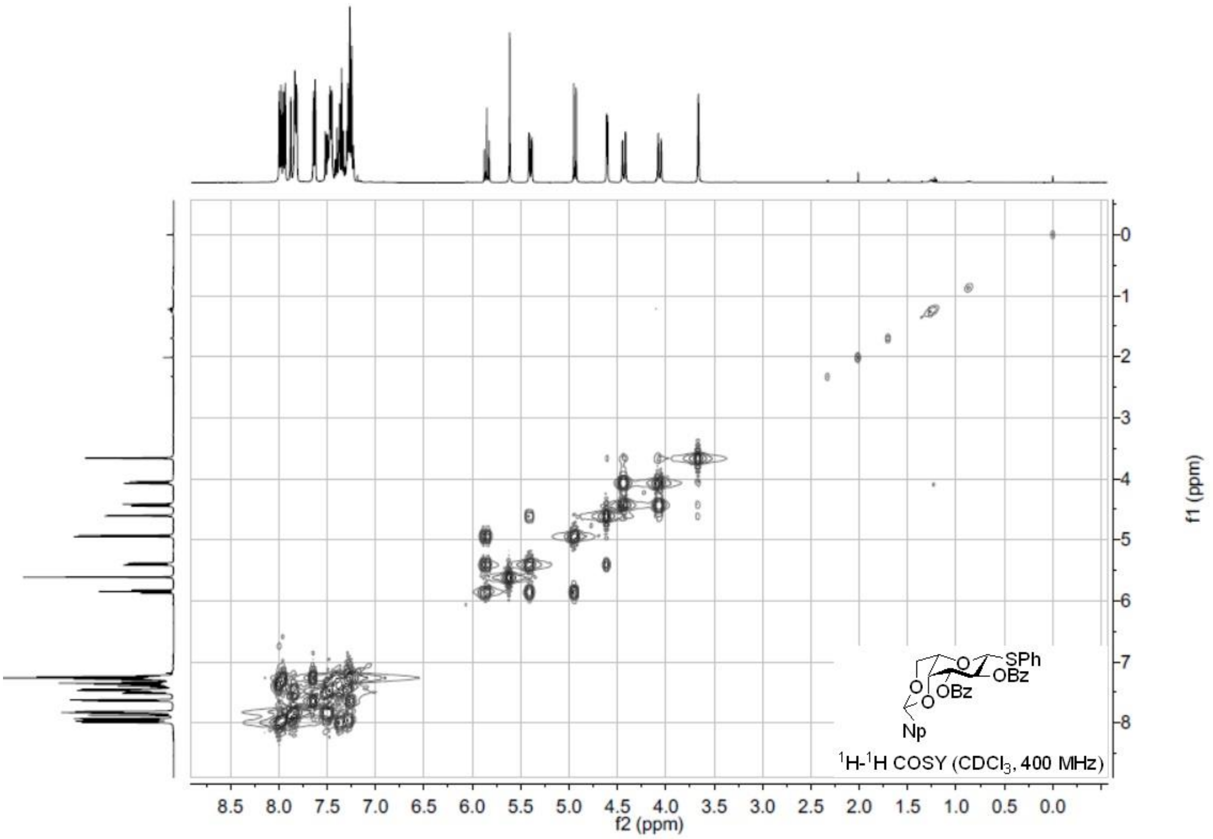
SPh
OBz
OBz
O
O
O
Np
^{1}H-^{1}H COSY (CDCl$_3$, 400 MHz)
f2 (ppm)
f1 (ppm)

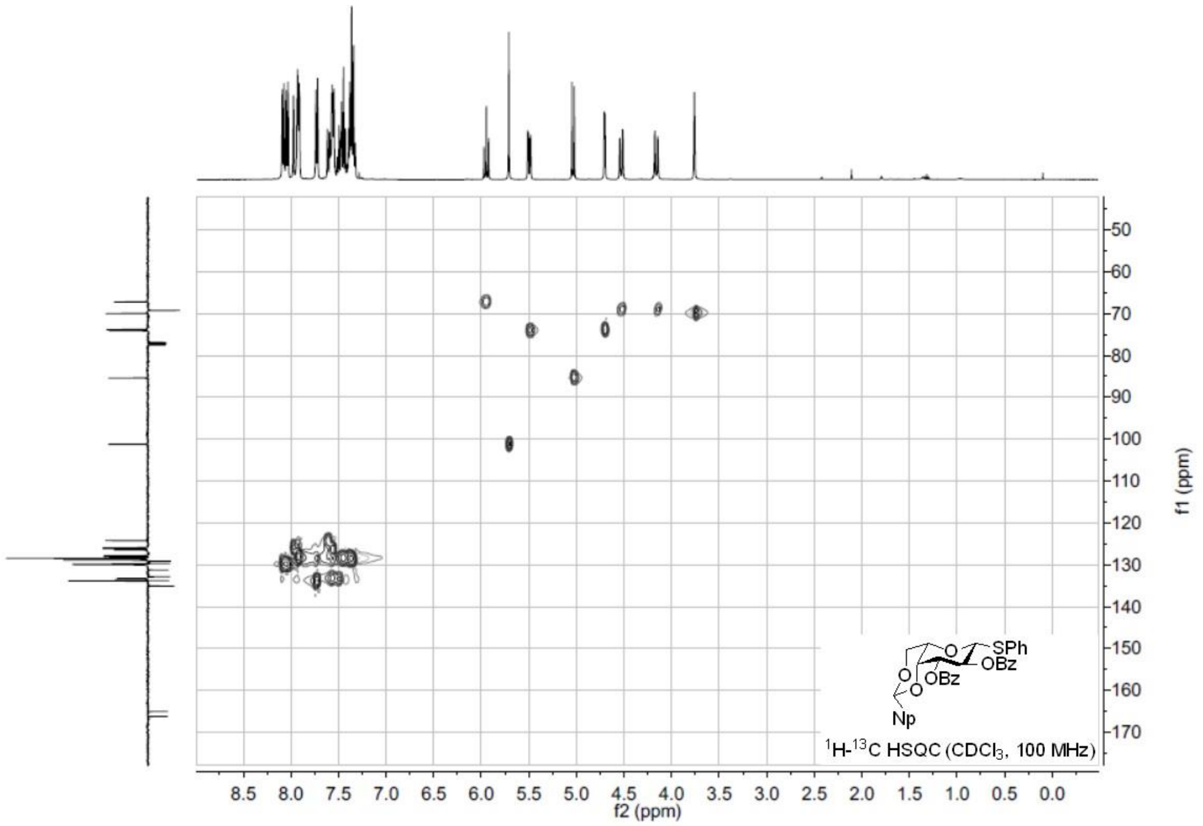
SPh
OBz
OBz
O
O
O
Np
^{1}H-^{13}C HSQC (CDCl$_3$, 100 MHz)
f2 (ppm)
f1 (ppm)

^{1}H and ^{13}C NMR spectra of compound **84**

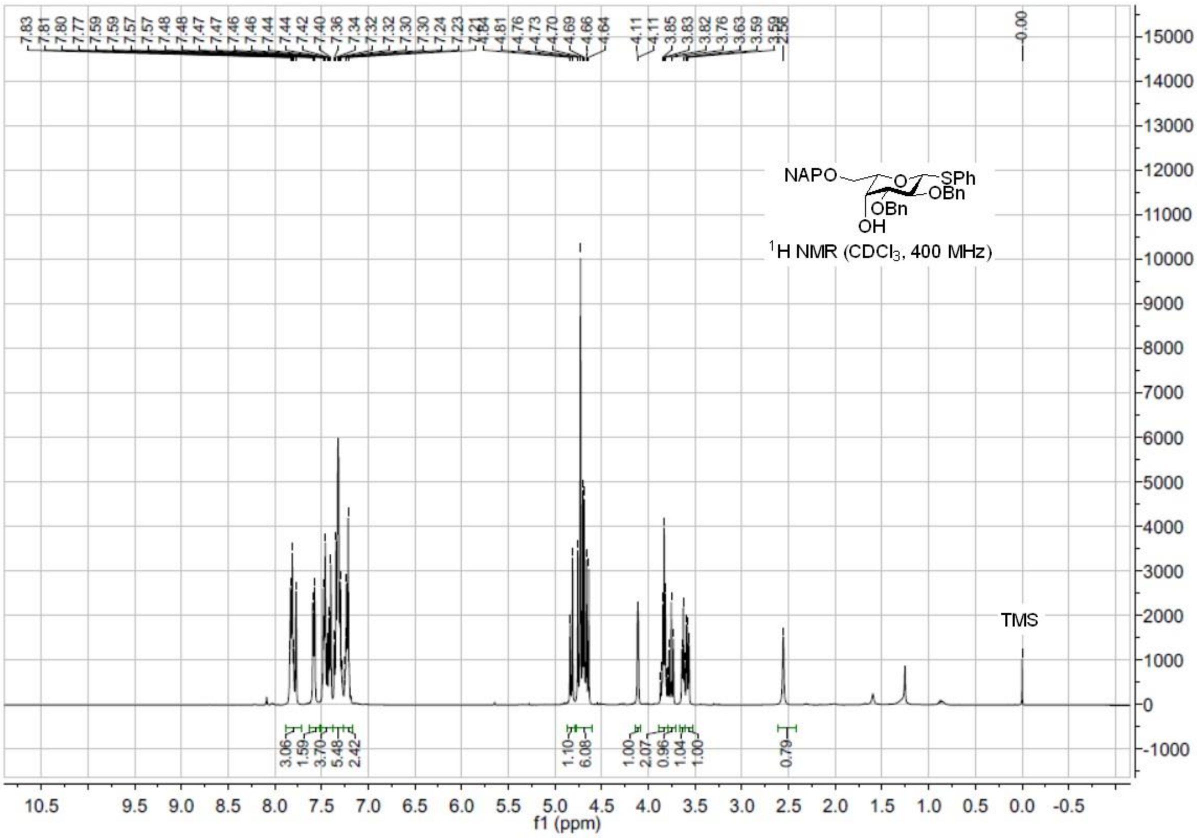

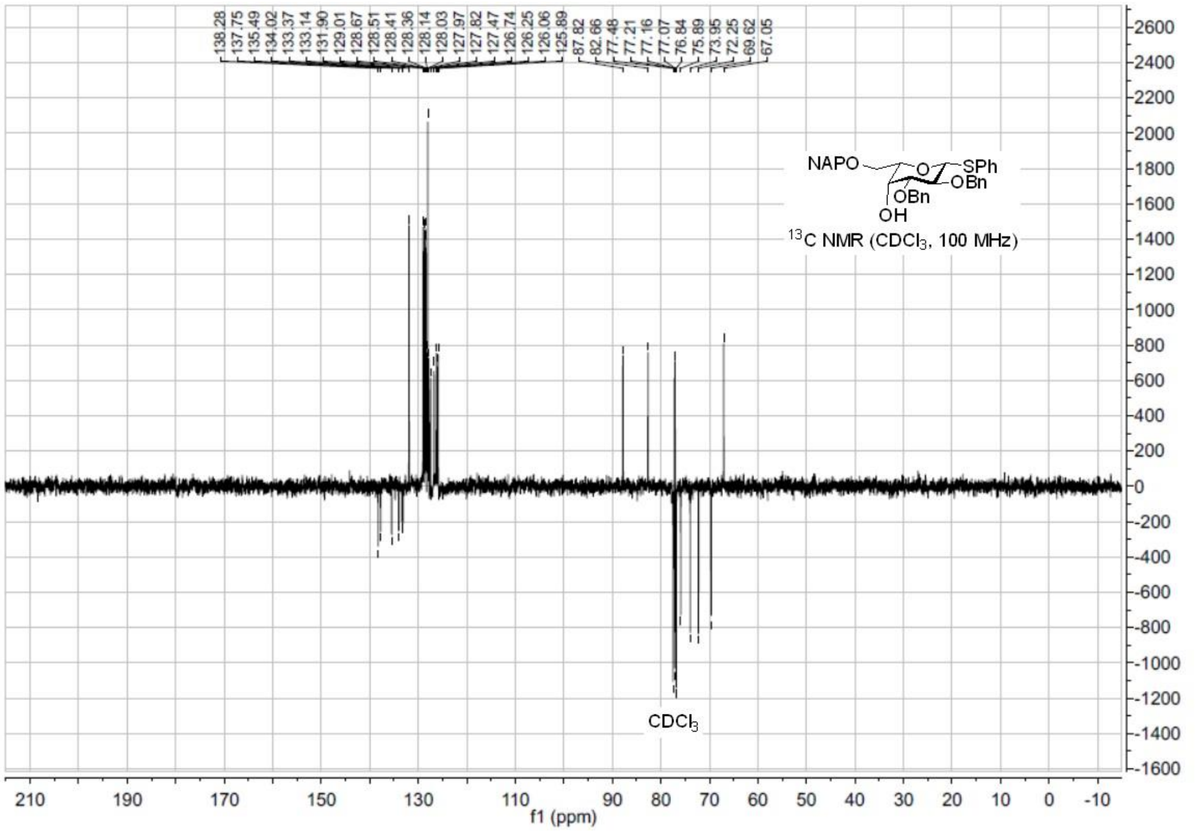

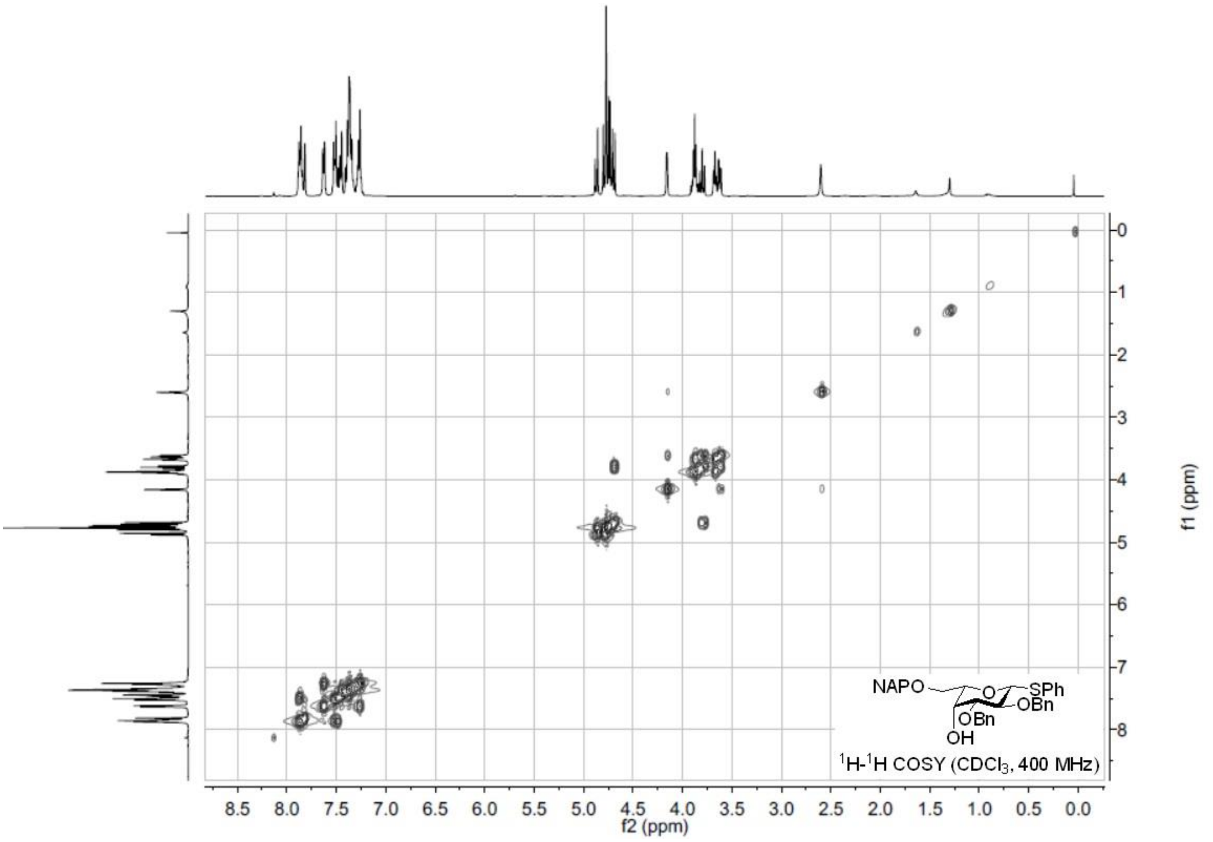
NAPO
SPh
OBn
OBn
OH
^{1}H-^{1}H COSY (CDCl$_3$, 400 MHz)
f2 (ppm)
f1 (ppm)

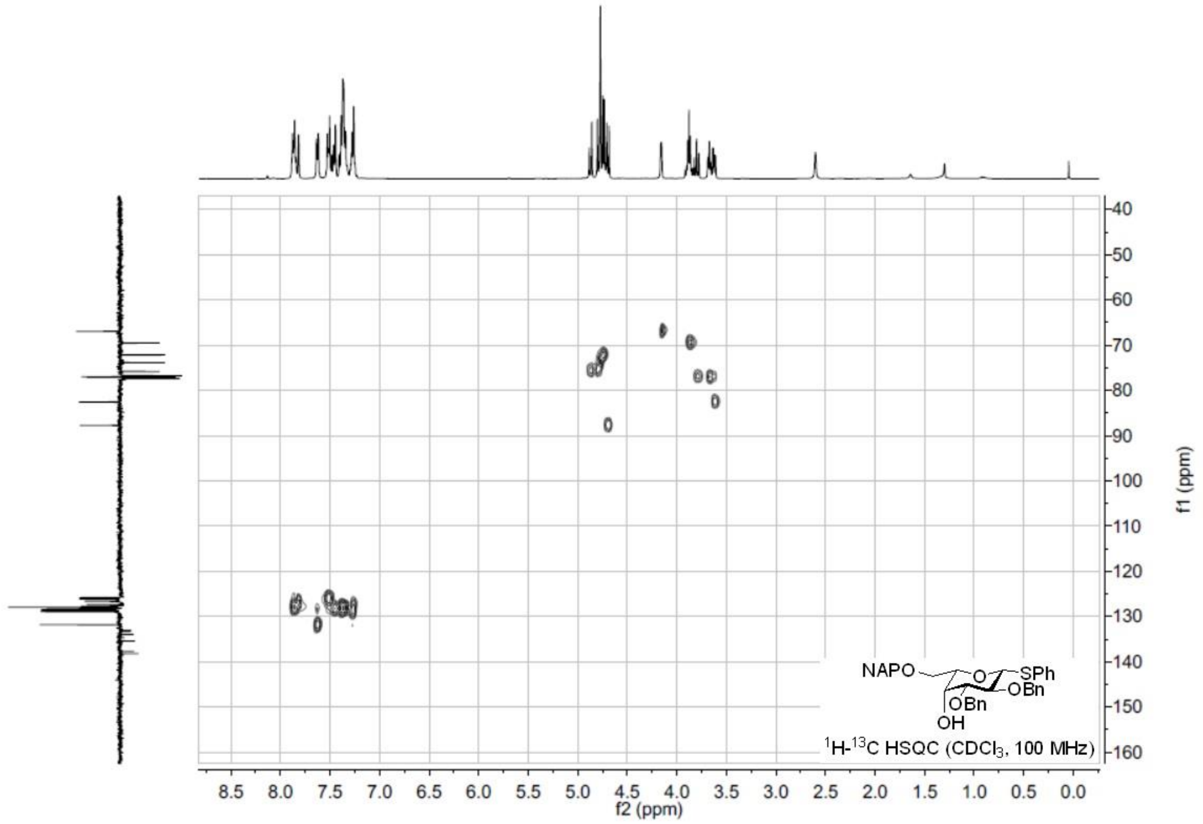
NAPO
SPh
OBn
OBn
OH
^{1}H-^{13}C HSQC (CDCl$_3$, 100 MHz)
f2 (ppm)
f1 (ppm)

^{1}H and ^{13}C NMR spectra of compound **85**

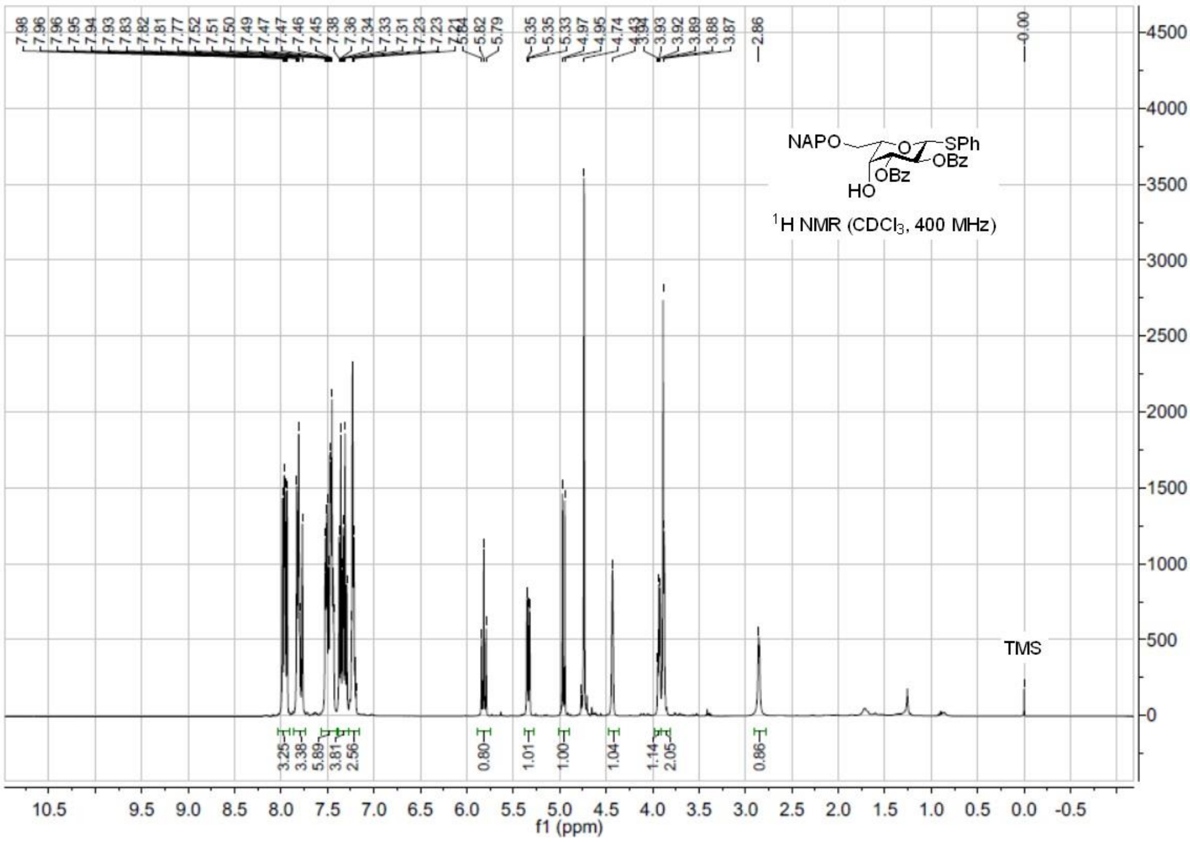

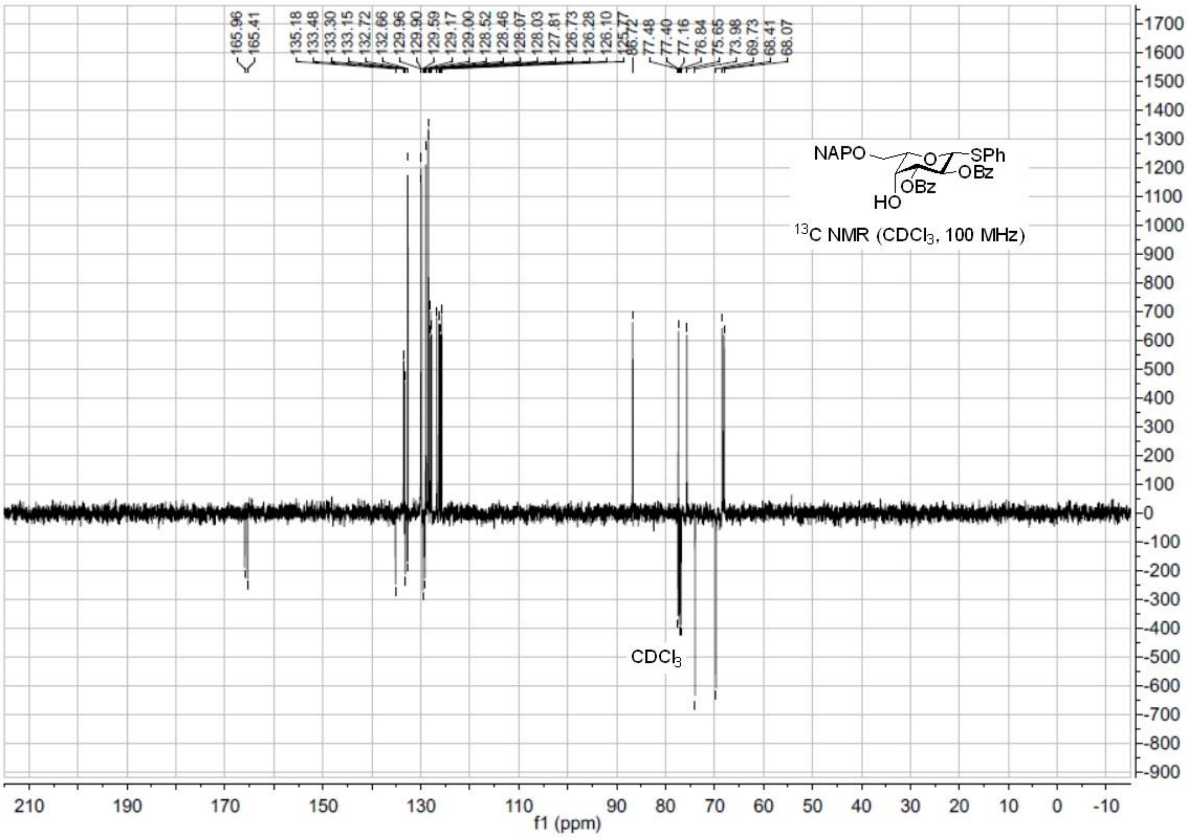

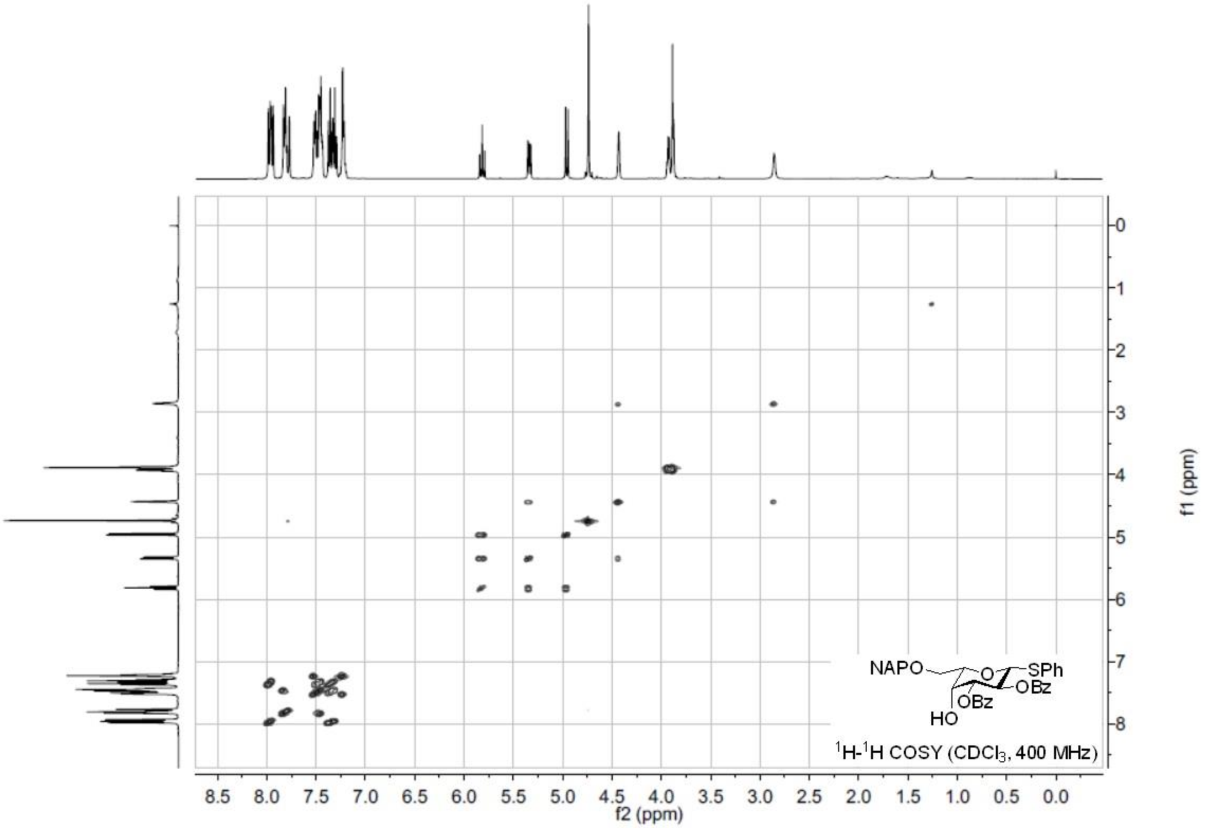
NAPO
SPh
OBz
OBz
HO
^{1}H-^{1}H COSY (CDCl$_3$, 400 MHz)
f2 (ppm)
f1 (ppm)

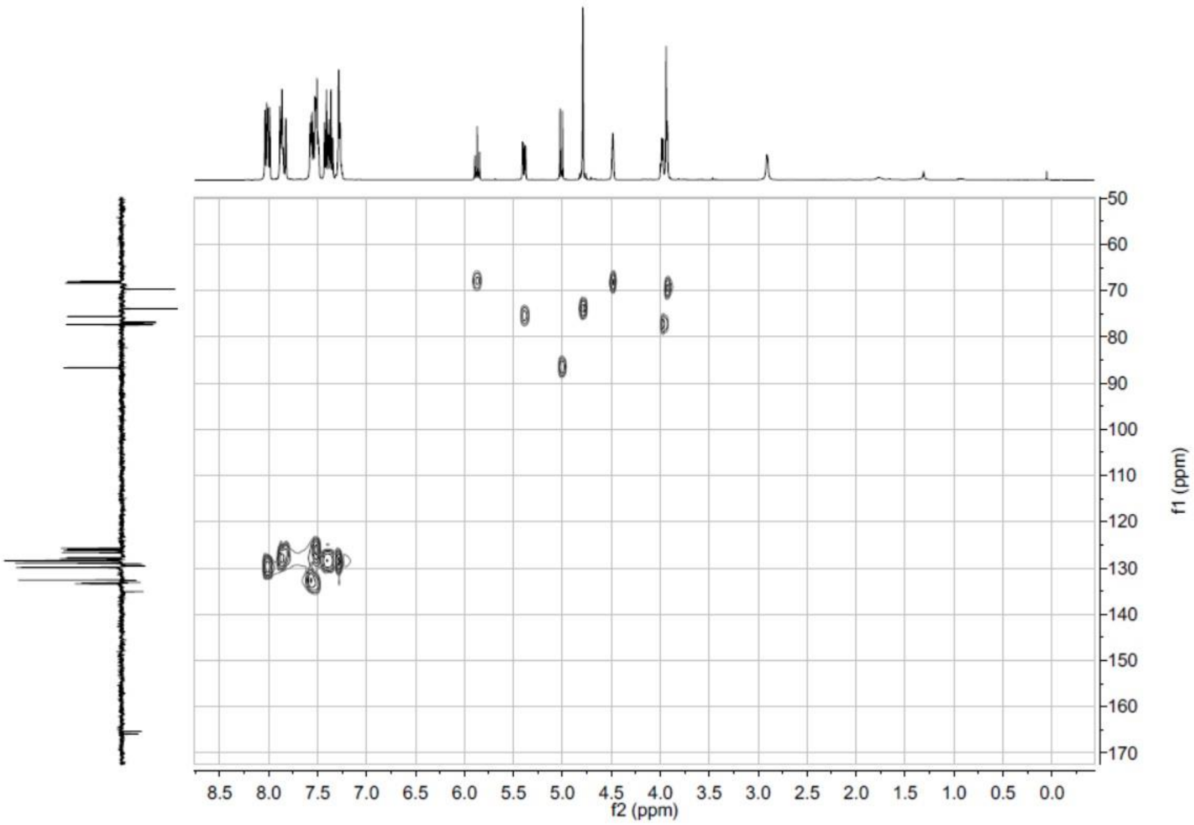
f2 (ppm)
f1 (ppm)

^{1}H and ^{13}C NMR spectra of compound **86**

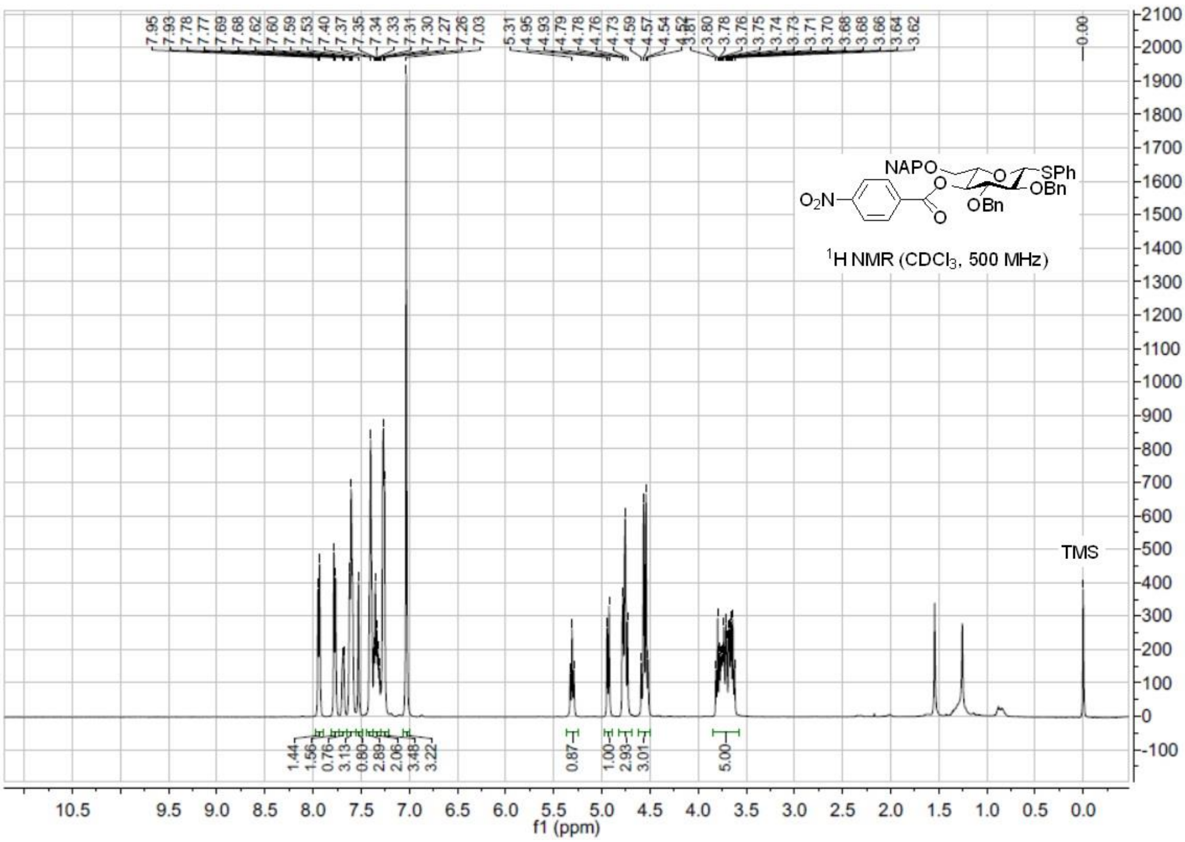

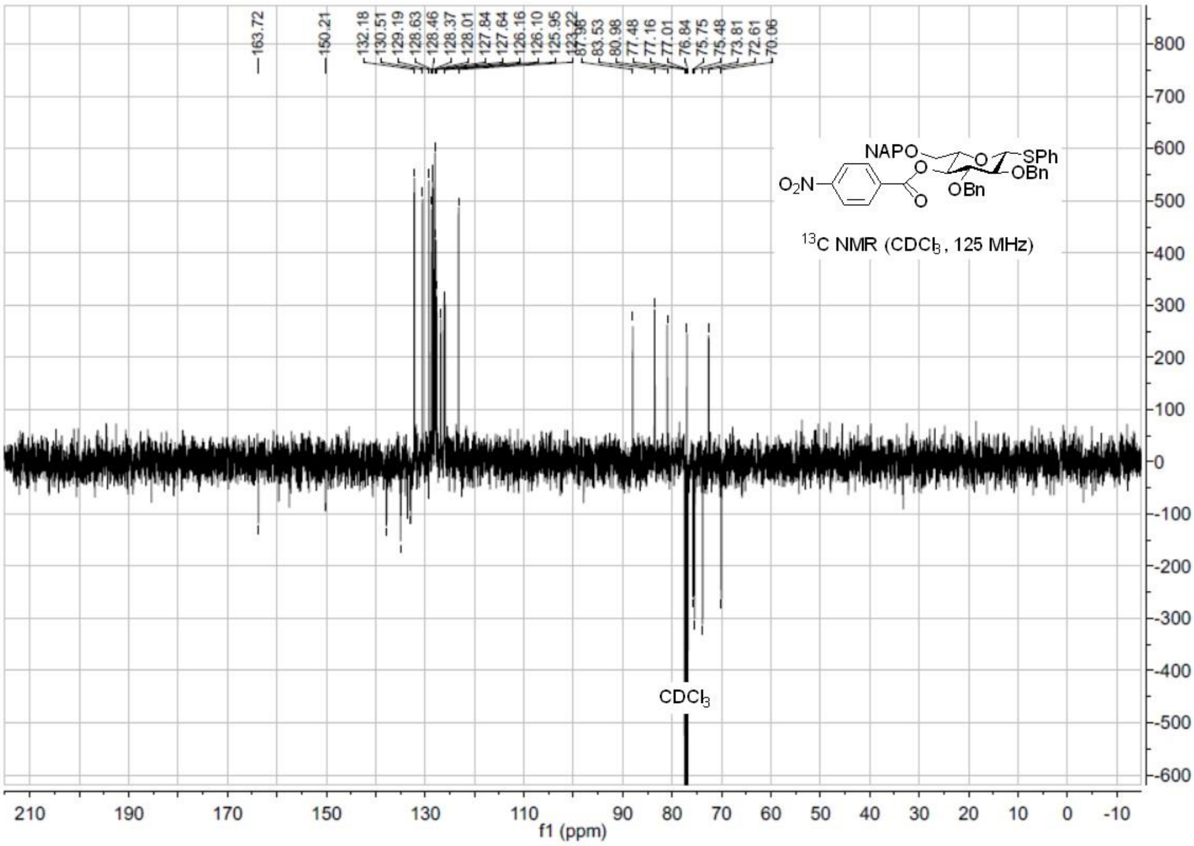

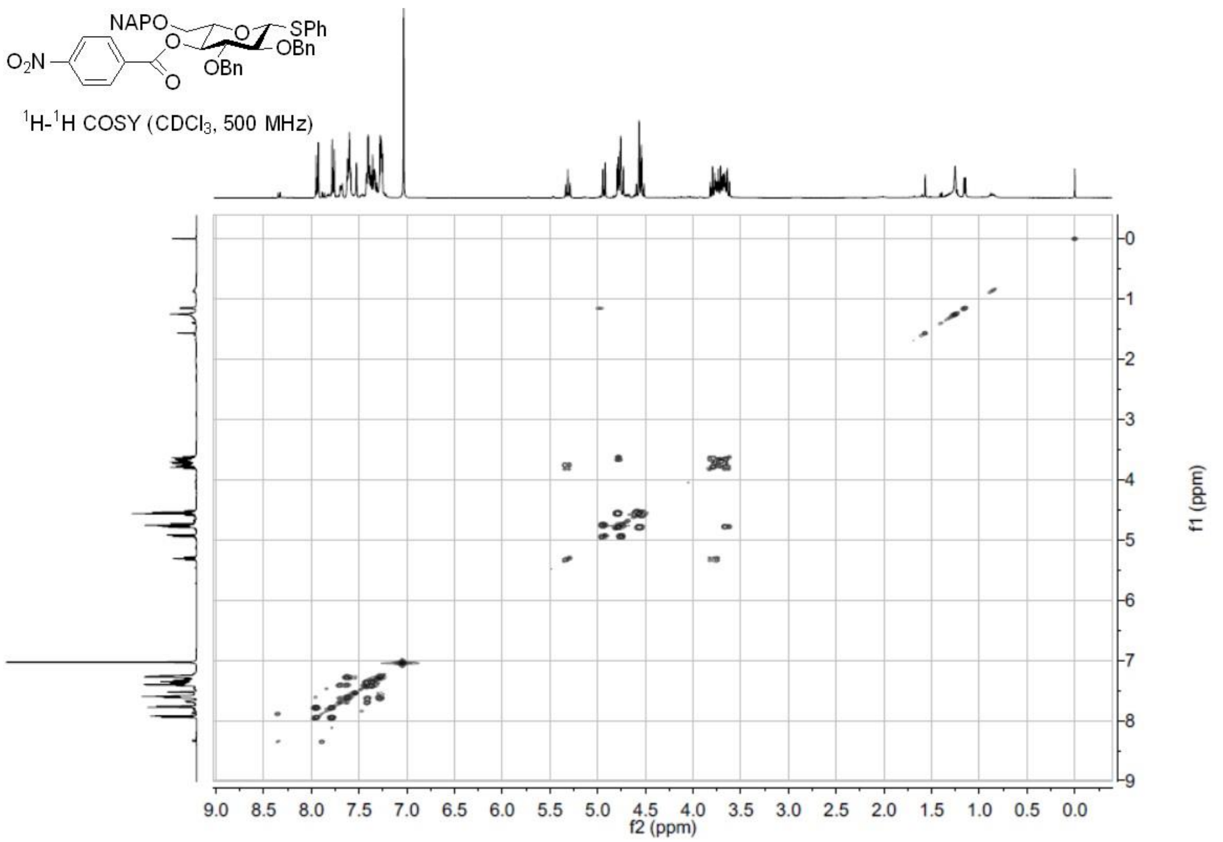
NAPO
O
SPh
OBn
O₂N
O
OBn
O
¹H-¹H COSY (CDCl₃, 500 MHz)
f1 (ppm)
f2 (ppm)

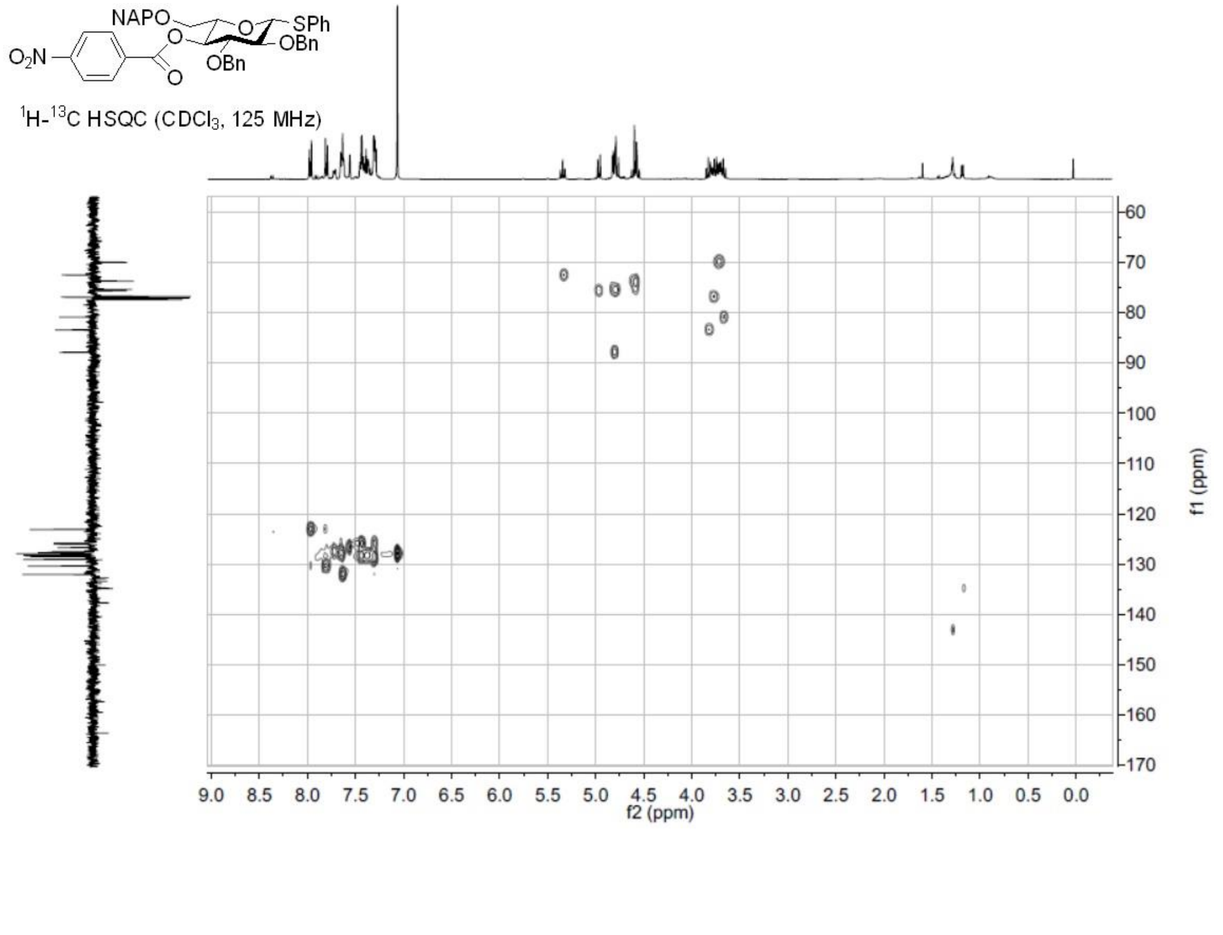
NAPO
O
SPh
OBn
O₂N
O
OBn
O
¹H-¹³C HSQC (CDCl₃, 125 MHz)
f1 (ppm)
f2 (ppm)

^{1}H and ^{13}C NMR spectra of compound **87**

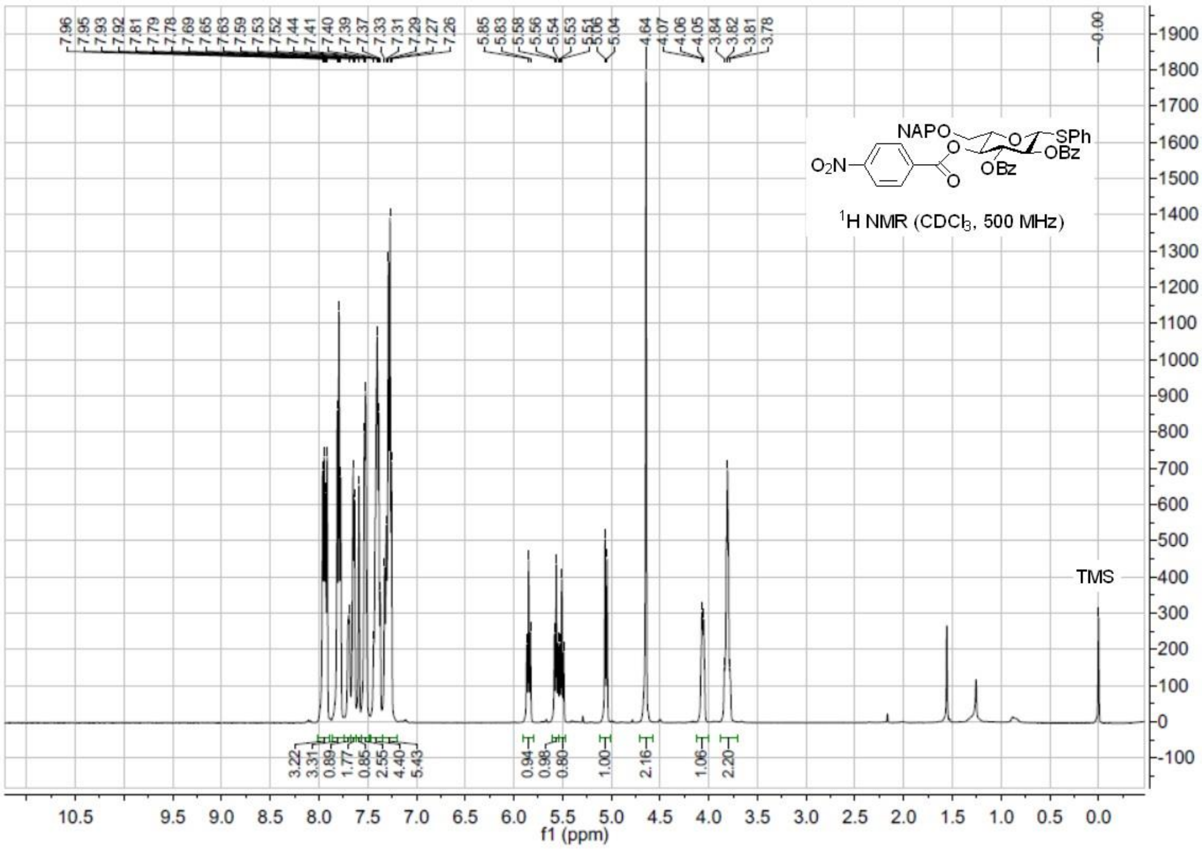

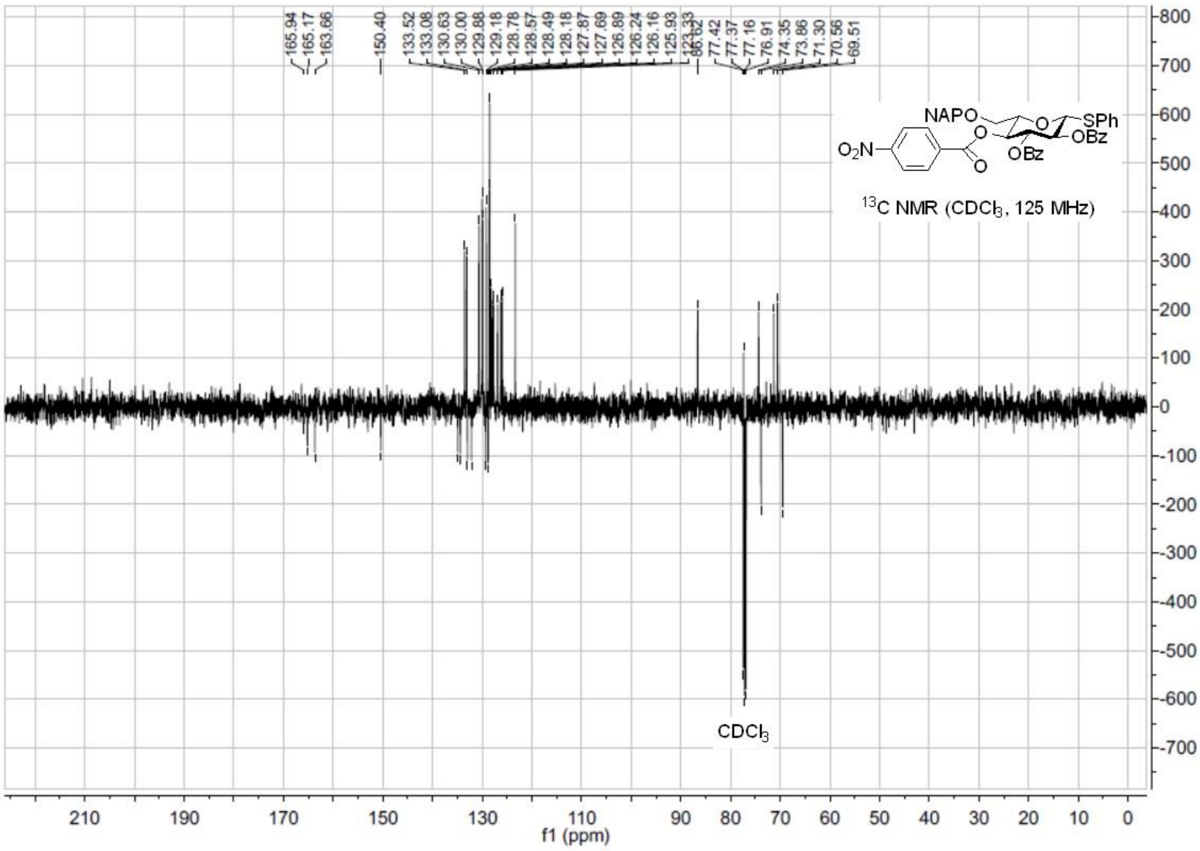

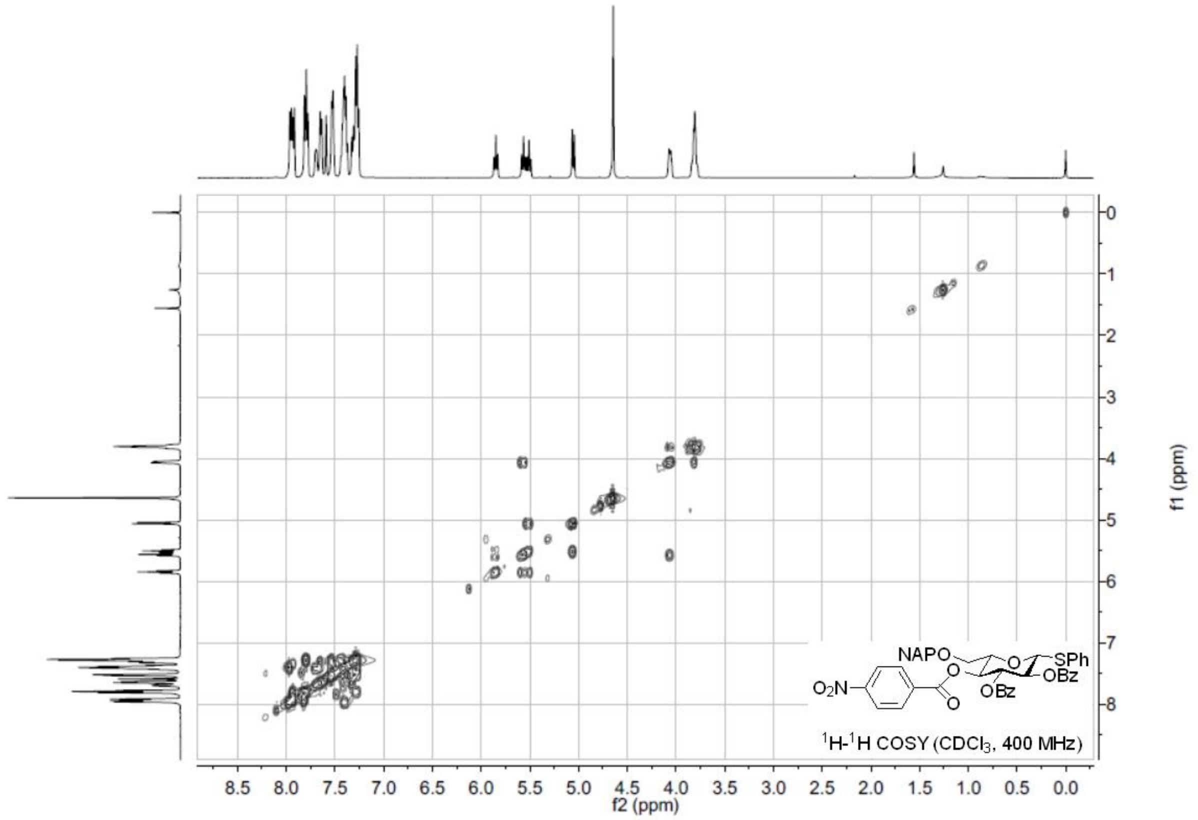
NAPO
SPh
OBz
O2N
OBz
1H-1H COSY (CDCl3, 400 MHz)
f2 (ppm)
f1 (ppm)

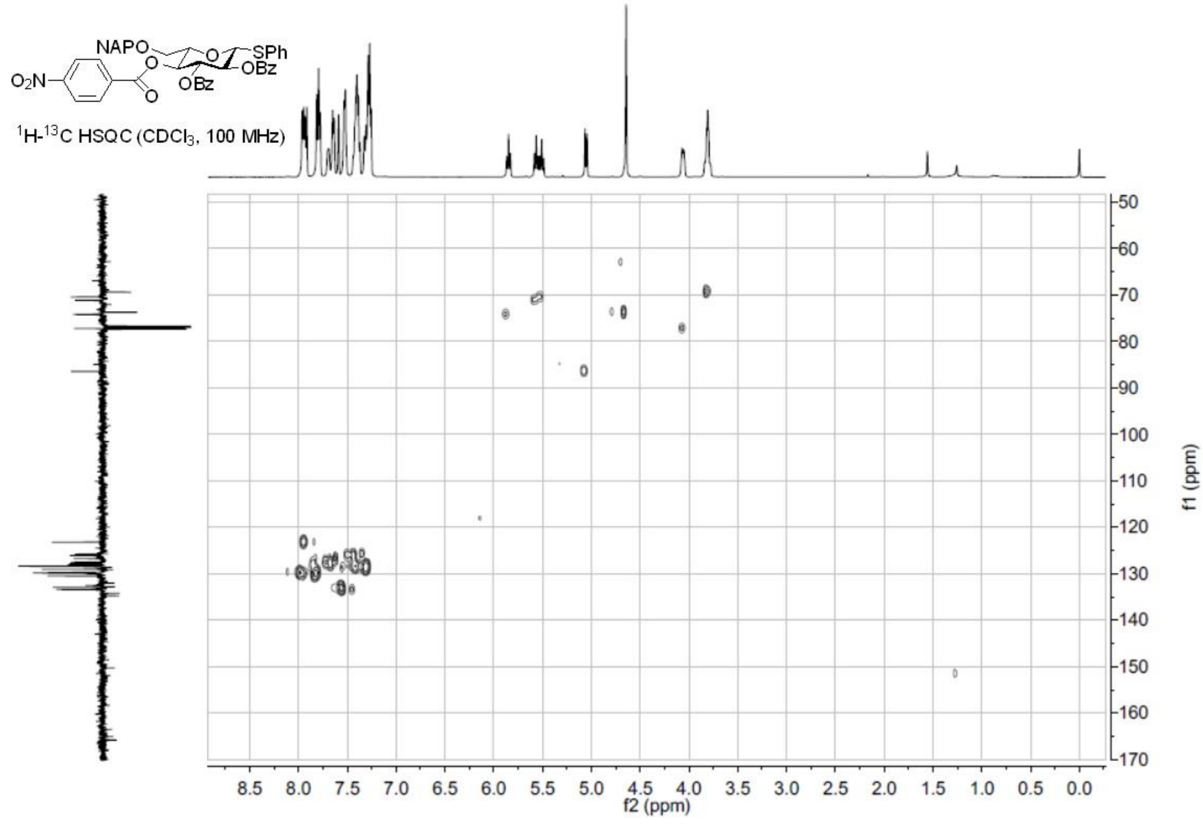
NAPO
SPh
OBz
O2N
OBz
1H-13C HSQC (CDCl3, 100 MHz)
f2 (ppm)
f1 (ppm)

^{1}H and ^{13}C NMR spectra of compound **88**

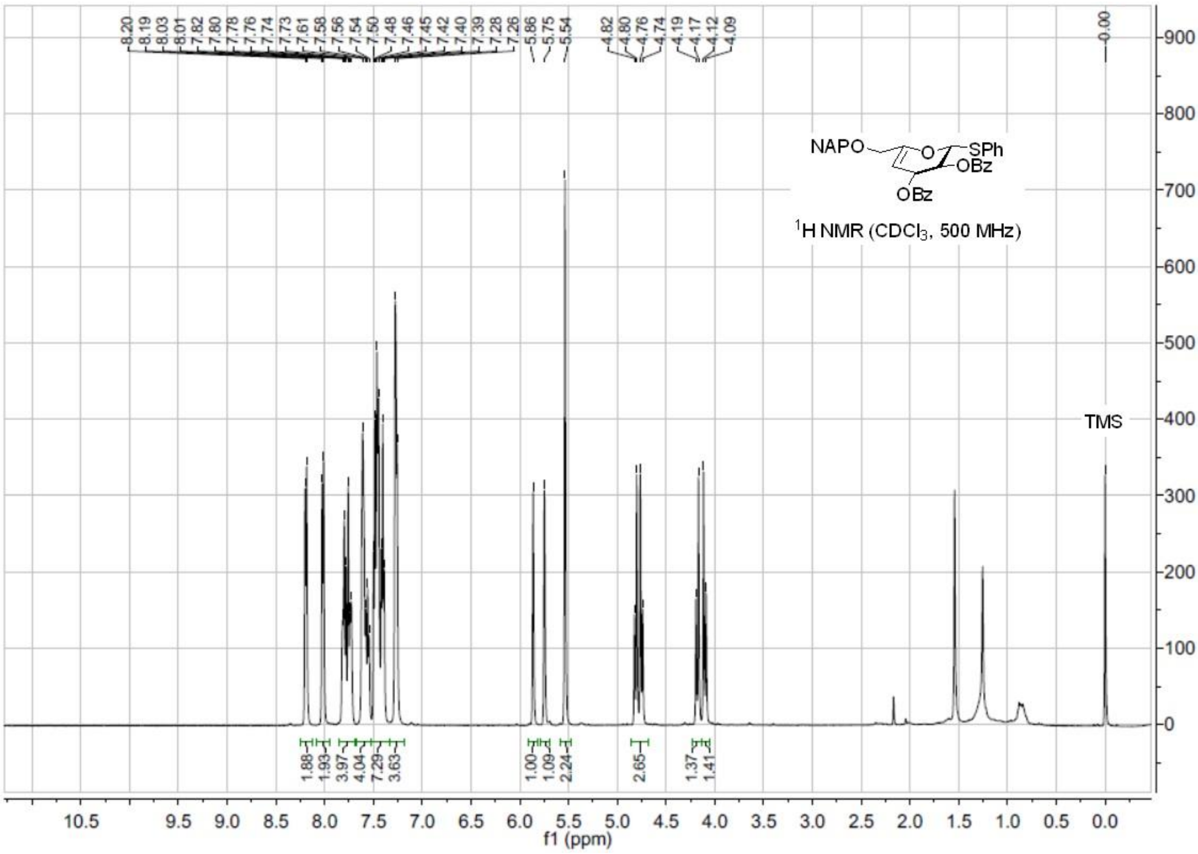

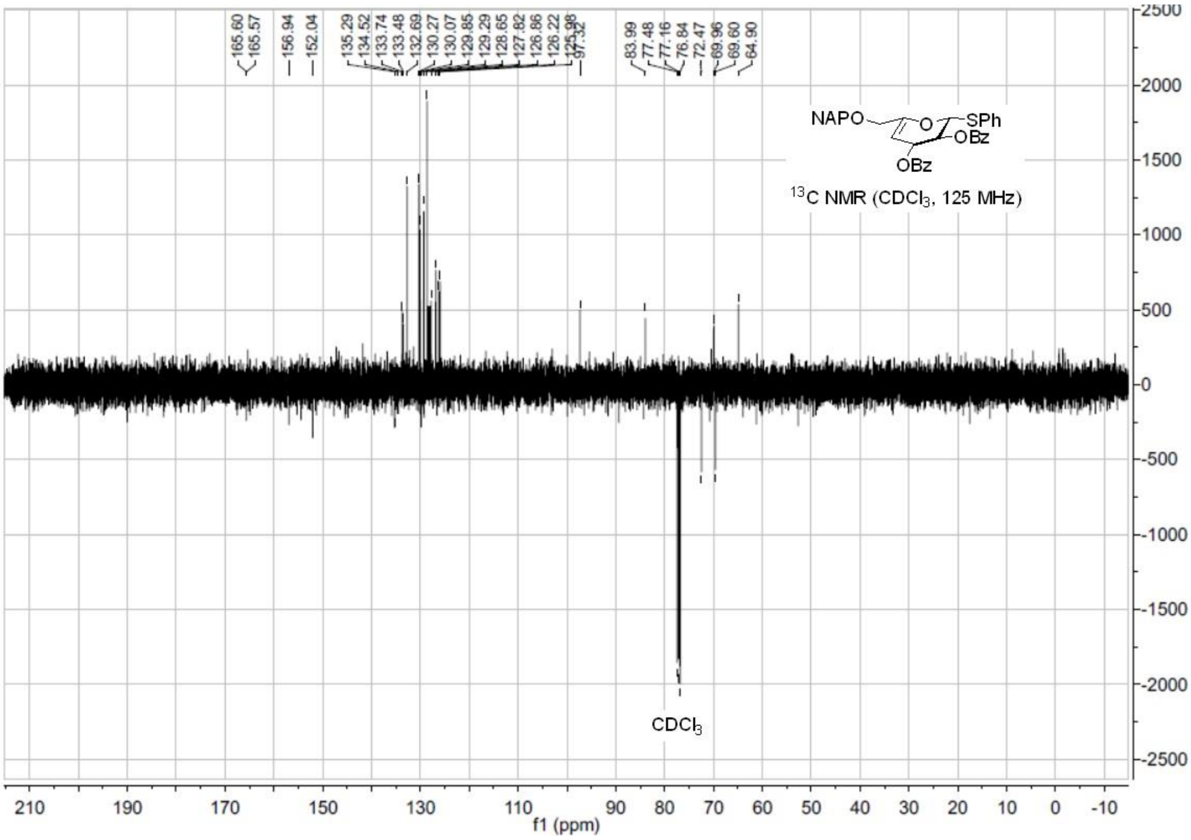

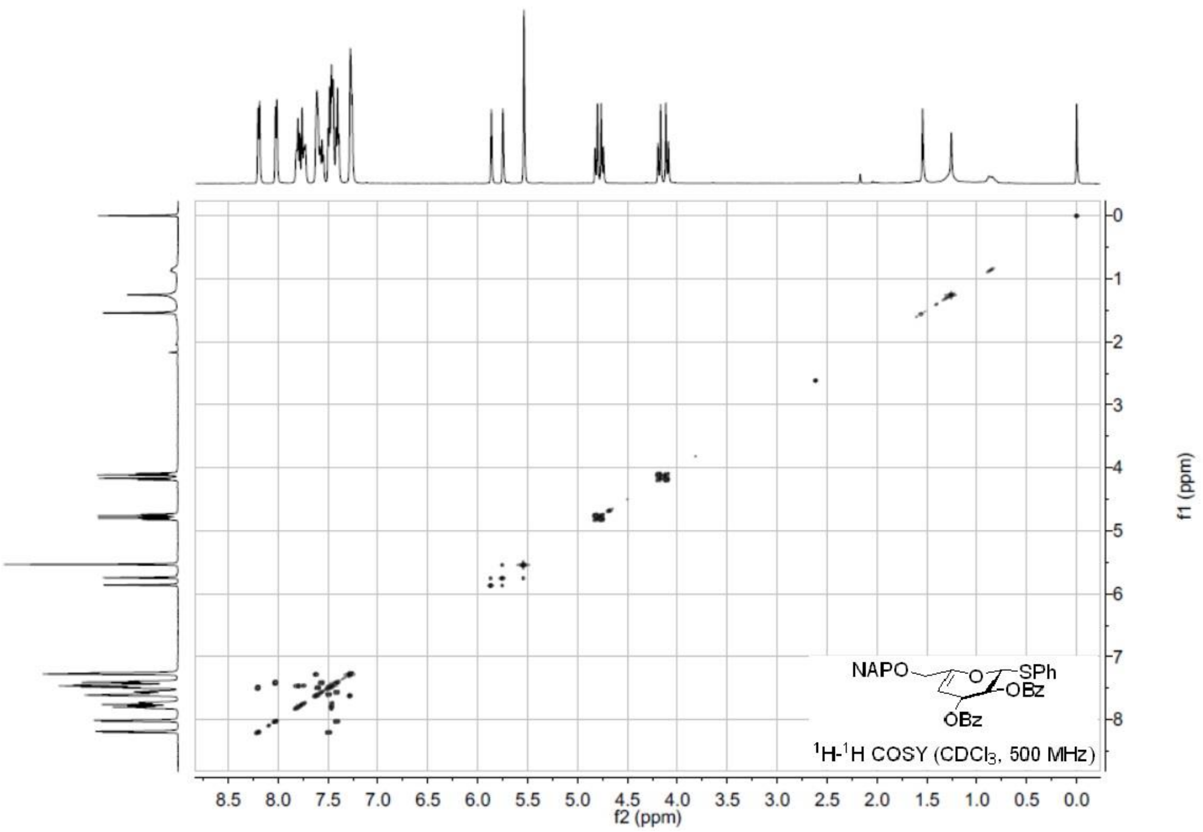
NAPO
O
SPh
OBz
OBz
^{1}H-^{1}H COSY (CDCl$_3$, 500 MHz)
f2 (ppm)
f1 (ppm)

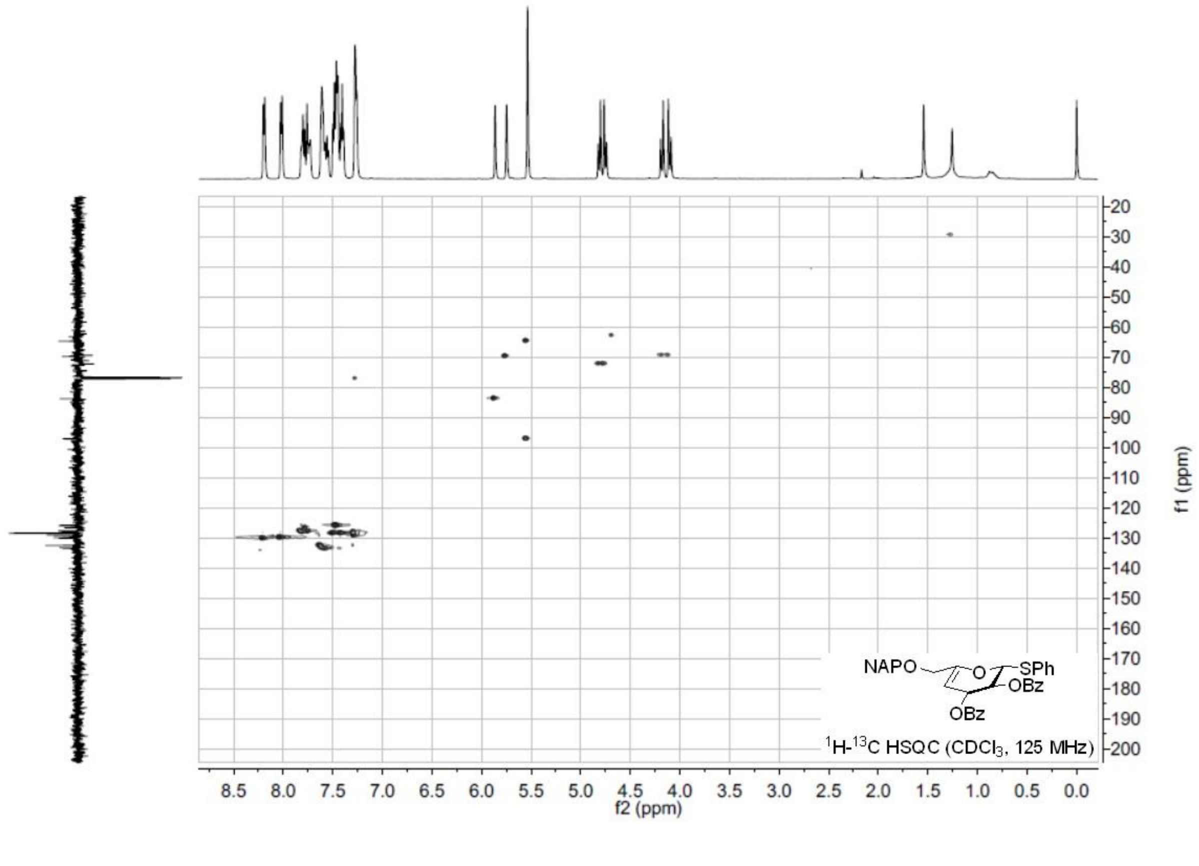
NAPO
O
SPh
OBz
OBz
^{1}H-^{13}C HSQC (CDCl$_3$, 125 MHz)
f2 (ppm)
f1 (ppm)

X-ray structure analysis of compound 83

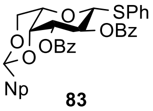

83

CCDC 2163274

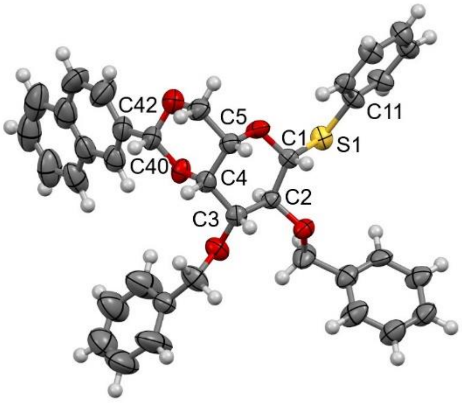

Experimental details

Crystal data	
Chemical formula	$C_{37}H_{34}O_5S$
M_r	590.70
Crystal system, space group	Monoclinic, *C*2
Temperature (K)	199
a, *b*, *c* (Å)	21.29 (2), 5.411 (4), 26.79 (2)
b (°)	95.97 (4)
V (Å^3)	3069 (5)
Z	4
Radiation type	Mo *K*a
m (mm^{-1})	0.15
Crystal size (mm)	0.35 × 0.08 × 0.05

Data collection	
Diffractometer	Bruker D8 VENTURE
Absorption correction	Multi-scan *SAINT* V8.38A (Bruker AXS Inc., 2017)
T_{min}, T_{max}	0.63, 0.99
No. of measured, independent and observed [$I > 2s(I)$] reflections	12038, 3399, 1822
R_{int}	0.164
q_{max} (°)	21.5
$(\sin q/l)_{max}$ (Å^{-1})	0.516
Refinement	
$R[F^2 > 2s(F^2)]$, $wR(F^2)$, S	0.098, 0.290, 1.05
No. of reflections	3399
No. of parameters	389
No. of restraints	343
H-atom treatment	H-atom parameters constrained
$D\rho_{max}$, $D\rho_{min}$ (e Å^{-3})	0.32, -0.26
Absolute structure	Flack x determined using 466 quotients [(I+)-(I-)]/[(I+)+(I-)] (Parsons, Flack and Wagner, Acta Cryst. B69 (2013) 249-259).
Absolute structure parameter	0.1 (2)

Computer programs: Bruker Instrument Service vV6.2.6, *APEX3* v2017.3-0 (Bruker AXS), *SAINT* V8.38A (Bruker AXS Inc., 2017), SHELXT 2014/5 (Sheldrick, 2014), *SHELXL2018*/3 (Sheldrick, 2018), *Mercury*, *WinGX*, *publCIF*.

References

Document origin: *publCIF* [Westrip, S. P. (2010). *J. Apply. Cryst.*, **43**, 920-925].

Computing details

Data collection: Bruker Instrument Service vV6.2.6; cell refinement: *APEX3* v2017.3-0 (Bruker AXS); data reduction: *SAINT* V8.38A (Bruker AXS Inc., 2017); program(s) used to solve structure: SHELXT 2014/5 (Sheldrick, 2014); program(s) used to refine structure: *SHELXL2018*/3 (Sheldrick, 2018); molecular graphics: *Mercury*; software used to prepare material for publication: *WinGX*, *publCIF*.

Crystal data

$C_{37}H_{34}O_5S$	*F*(000) = 1248
M_r = 590.70	D_x = 1.278 Mg m^{-3}
Monoclinic, *C*2	Mo *K*a radiation, l = 0.71073 Å
a = 21.29 (2) Å	Cell parameters from 1699 reflections
b = 5.411 (4) Å	q = 2.6–20.8°
c = 26.79 (2) Å	m = 0.15 mm^{-1}
b = 95.97 (4)°	*T* = 199 K
V = 3069 (5) Å^3	Needle, colourless
Z = 4	0.35 × 0.08 × 0.05 mm

Data collection

Bruker D8 VENTURE diffractometer	3399 independent reflections
Radiation source: microfocus sealed tube, INCOATEC ImS 3.0	1822 reflections with *I* > 2s(*I*)
Multilayer mirror INCOATEC monochromator	R_{int} = 0.164
Detector resolution: 7.3910 pixels mm^{-1}	q_{max} = 21.5°, q_{min} = 2.6°

w and p scan	h = -21®21
Absorption correction: multi-scan *SAINT* V8.38A (Bruker AXS Inc., 2017)	k = -5®5
T_{min} = 0.63, T_{max} = 0.99	l = -27®27
12038 measured reflections	

Refinement

Refinement on F^2	Hydrogen site location: inferred from neighbouring sites
Least-squares matrix: full	H-atom parameters constrained
$R[F^2 > 2s(F^2)] = 0.098$	$w = 1/[s^2(F_o^2) + (0.1335P)^2 + 3.4846P]$ where $P = (F_o^2 + 2F_c^2)/3$
$wR(F^2) = 0.290$	$(D/s)_{max} = 0.019$
S = 1.05	$D\rho_{max}$ = 0.32 e Å^{-3}
3399 reflections	$D\rho_{min}$ = -0.26 e Å^{-3}
389 parameters	Extinction correction: *SHELXL2018*/3 (Sheldrick 2018)
343 restraints	Extinction coefficient: 0.009 (2)
Primary atom site location: structure-invariant direct methods	Absolute structure: Flack x determined using 466 quotients [(I+)-(I-)]/[(I+)+(I-)] (Parsons, Flack and Wagner, Acta Cryst. B69 (2013) 249-259).
Secondary atom site location: difference Fourier map	Absolute structure parameter: 0.1 (2)

Special details

Geometry. All esds (except the esd in the dihedral angle between two l.s. planes) are estimated using the full covariance matrix. The cell esds are taken into account individually in the estimation of esds in distances, angles and torsion angles; correlations between esds in cell parameters are only used when they are defined by crystal symmetry. An approximate (isotropic) treatment of cell esds is used for estimating esds involving l.s. planes.

Fractional atomic coordinates and isotropic or equivalent isotropic displacement parameters (Å²) for compound ***83***

	x	y	z	U_{iso}*/U_{eq}
C1	0.2589 (7)	0.221 (3)	0.3468 (5)	0.048 (4)
H1	0.25576	0.365859	0.369712	0.057*
C2	0.2057 (7)	0.237 (3)	0.3056 (6)	0.050 (4)
H2	0.202681	0.073935	0.287767	0.06*
C3	0.2152 (7)	0.434 (3)	0.2676 (6)	0.057 (3)
H3	0.205917	0.597341	0.282828	0.069*
C4	0.2823 (8)	0.443 (4)	0.2535 (6)	0.060 (4)
H4	0.288064	0.59771	0.234074	0.072*
C5	0.3292 (7)	0.442 (4)	0.3005 (6)	0.063 (4)
H5	0.32614	0.5963	0.320592	0.075*
C6	0.3931 (8)	0.412 (4)	0.2837 (7)	0.077 (5)
H6A	0.404054	0.565941	0.266488	0.092*
H6AB	0.423985	0.39189	0.31364	0.092*
C11	0.2984 (8)	-0.024 (3)	0.4396 (6)	0.060 (4)
C12	0.2962 (8)	-0.208 (3)	0.4715 (7)	0.067 (5)
H12	0.269851	-0.346396	0.462764	0.08*
C13	0.3332 (8)	-0.200 (3)	0.5192 (7)	0.064 (5)

H13	0.328763	-0.324591	0.543622	0.077*
C14	0.3754 (9)	-0.010 (4)	0.5289 (7)	0.076 (5)
H14	0.402213	-0.006632	0.55953	0.091*
C15	0.3791 (8)	0.180 (3)	0.4939 (7)	0.068 (5)
H15	0.406652	0.315251	0.501867	0.082*
C16	0.3429 (8)	0.173 (3)	0.4476 (6)	0.056 (4)
H16	0.347532	0.293586	0.42255	0.068*
C20	0.0950 (8)	0.142 (4)	0.3066 (7)	0.087 (6)
H20A	0.10582	-0.033659	0.301803	0.105*
H20B	0.078869	0.212315	0.273572	0.105*
C21	0.0459 (9)	0.164 (4)	0.3429 (7)	0.074 (5)
C22	0.0435 (8)	-0.007 (4)	0.3816 (7)	0.080 (6)
H22	0.073017	-0.138598	0.385408	0.096*
C23	-0.0023 (8)	0.017 (4)	0.4144 (8)	0.084 (6)
H23	-0.003728	-0.0974	0.441152	0.101*
C24	-0.0447 (9)	0.201 (4)	0.4086 (8)	0.085 (6)
H24	-0.076431	0.213606	0.430939	0.102*
C25	-0.0428 (10)	0.367 (4)	0.3716 (8)	0.094 (7)
H25	-0.072777	0.497068	0.368126	0.113*
C26	0.0022 (10)	0.348 (4)	0.3387 (10)	0.096 (7)
H26	0.002881	0.466663	0.312575	0.115*
C30	0.1549 (11)	0.611 (3)	0.1950 (7)	0.087 (6)
H30A	0.19406	0.704277	0.191137	0.105*
H30B	0.125839	0.71992	0.211282	0.105*
C31	0.1248 (9)	0.534 (4)	0.1447 (8)	0.077 (6)
C32	0.0822 (13)	0.672 (6)	0.1176 (11)	0.157 (13)
H32	0.067284	0.822886	0.130294	0.188*

C33	0.0595 (15)	0.585 (7)	0.0681 (11)	0.160 (14)
H33	0.027039	0.674729	0.049055	0.192*
C34	0.0810 (15)	0.395 (6)	0.0493 (11)	0.130 (10)
H34	0.06718	0.346965	0.015876	0.156*
C35	0.1201 (16)	0.274 (7)	0.0753 (11)	0.152 (12)
H35	0.135178	0.126575	0.061371	0.182*
C36	0.1442 (16)	0.337 (6)	0.1238 (11)	0.155 (13)
H36	0.175116	0.235194	0.14175	0.186*
C40	0.3556 (9)	0.235 (4)	0.2085 (7)	0.079 (5)
H40	0.36324	0.395726	0.19176	0.095*
C41	0.3148 (11)	-0.064 (4)	0.1431 (7)	0.087 (5)
H41	0.27354	-0.001411	0.145647	0.104*
C42	0.3644 (12)	0.027 (4)	0.1722 (8)	0.088 (6)
C43	0.4214 (12)	-0.058 (6)	0.1671 (9)	0.124 (7)
H43	0.455301	0.0129	0.188229	0.149*
C44	0.4367 (13)	-0.234 (6)	0.1353 (10)	0.132 (9)
H44	0.478872	-0.289074	0.134098	0.159*
C45	0.3954 (18)	-0.537 (6)	0.0676 (12)	0.143 (9)
H45	0.437036	-0.596416	0.065121	0.172*
C44A	0.3828 (16)	-0.336 (5)	0.1023 (10)	0.116 (7)
C46	0.3442 (19)	-0.635 (5)	0.0390 (12)	0.139 (11)
H46	0.34864	-0.763565	0.015636	0.167*
C47	0.2878 (17)	-0.540 (5)	0.0456 (9)	0.132 (9)
H47	0.2523	-0.608883	0.02592	0.158*
C48	0.2766 (14)	-0.362 (4)	0.0764 (8)	0.112 (7)
H48	0.234617	-0.303568	0.077428	0.134*
C48A	0.3250 (13)	-0.259 (5)	0.1074 (8)	0.100 (6)

O2	0.1490 (5)	0.274 (2)	0.3271 (4)	0.060 (3)
O3	0.1699 (6)	0.3946 (19)	0.2263 (4)	0.070 (3)
O4	0.2916 (5)	0.2325 (19)	0.2223 (4)	0.065 (3)
O6	0.3182 (5)	0.225 (2)	0.3294 (4)	0.064 (3)
O60	0.3992 (6)	0.210 (3)	0.2510 (5)	0.086 (4)
S1	0.2496 (2)	-0.0558 (8)	0.38186 (16)	0.0641 (15)

*Atomic displacement parameters (Å^2) for compound **83***

	U^{11}	U^{22}	U^{33}	U^{12}	U^{13}	U^{23}
C1	0.056 (7)	0.033 (8)	0.053 (9)	-0.003 (7)	0.003 (6)	0.000 (7)
C2	0.047 (7)	0.051 (10)	0.054 (9)	-0.001 (8)	0.008 (6)	0.001 (7)
C3	0.065 (8)	0.045 (8)	0.062 (9)	0.002 (8)	0.008 (6)	-0.001 (8)
C4	0.071 (8)	0.046 (8)	0.065 (9)	0.003 (9)	0.014 (6)	0.008 (8)
C5	0.056 (8)	0.061 (9)	0.072 (9)	0.002 (9)	0.012 (6)	0.012 (9)
C6	0.059 (9)	0.080 (12)	0.092 (12)	-0.001 (10)	0.019 (8)	0.017 (9)
C11	0.076 (11)	0.041 (9)	0.058 (9)	0.003 (7)	-0.009 (8)	0.004 (7)
C12	0.073 (12)	0.040 (9)	0.083 (11)	-0.013 (9)	-0.012 (9)	0.011 (8)
C13	0.075 (12)	0.051 (9)	0.066 (10)	0.008 (8)	0.002 (8)	0.005 (9)
C14	0.085 (13)	0.075 (12)	0.066 (11)	-0.008 (9)	-0.001 (10)	0.003 (8)
C15	0.079 (12)	0.051 (9)	0.073 (10)	-0.008 (9)	-0.004 (8)	-0.007 (8)
C16	0.063 (10)	0.042 (8)	0.065 (9)	0.006 (7)	0.011 (7)	0.002 (8)
C20	0.055 (9)	0.132 (17)	0.075 (12)	-0.025 (10)	0.007 (8)	-0.026 (12)
C21	0.060 (10)	0.078 (11)	0.085 (12)	-0.010 (8)	0.014 (9)	-0.007 (9)
C22	0.038 (10)	0.096 (14)	0.104 (14)	0.010 (10)	0.003 (8)	0.017 (10)
C23	0.058 (12)	0.075 (13)	0.123 (16)	-0.007 (8)	0.023 (10)	0.023 (11)

C24	0.065 (12)	0.087 (13)	0.107 (15)	0.002 (9)	0.032 (11)	0.007 (11)
C25	0.084 (14)	0.076 (14)	0.128 (18)	0.011 (10)	0.038 (12)	0.019 (11)
C26	0.074 (13)	0.071 (13)	0.150 (19)	-0.007 (9)	0.042 (12)	0.027 (12)
C30	0.122 (17)	0.058 (11)	0.077 (11)	0.000 (11)	-0.015 (11)	0.005 (8)
C31	0.073 (13)	0.084 (13)	0.074 (11)	0.004 (9)	0.000 (9)	0.010 (8)
C32	0.16 (3)	0.17 (3)	0.124 (18)	0.09 (2)	-0.056 (17)	-0.028 (17)
C33	0.15 (3)	0.22 (3)	0.106 (19)	0.07 (2)	-0.046 (18)	-0.014 (18)
C34	0.13 (2)	0.15 (3)	0.099 (19)	-0.003 (17)	-0.008 (15)	-0.003 (16)
C35	0.19 (3)	0.16 (3)	0.091 (17)	0.05 (2)	-0.021 (17)	-0.026 (16)
C36	0.20 (3)	0.127 (19)	0.122 (18)	0.07 (2)	-0.050 (19)	-0.041 (16)
C40	0.072 (10)	0.099 (14)	0.071 (10)	0.008 (10)	0.029 (7)	0.013 (9)
C41	0.110 (14)	0.075 (12)	0.078 (13)	0.008 (12)	0.027 (10)	0.020 (10)
C42	0.095 (13)	0.105 (15)	0.070 (12)	0.017 (11)	0.032 (10)	0.013 (9)
C43	0.104 (13)	0.137 (19)	0.139 (19)	0.025 (14)	0.053 (14)	0.011 (15)
C44	0.111 (16)	0.15 (2)	0.14 (2)	0.059 (15)	0.051 (13)	0.018 (14)
C45	0.20 (3)	0.106 (18)	0.14 (2)	0.020 (18)	0.096 (18)	0.023 (14)
C44A	0.159 (16)	0.096 (16)	0.101 (16)	0.031 (14)	0.051 (13)	0.035 (11)
C46	0.23 (3)	0.079 (18)	0.12 (2)	0.019 (16)	0.087 (19)	0.017 (14)
C47	0.21 (3)	0.097 (16)	0.095 (16)	0.003 (17)	0.053 (17)	-0.020 (13)
C48	0.183 (19)	0.088 (15)	0.064 (14)	0.030 (14)	0.007 (12)	-0.003 (10)

C48A	0.143 (16)	0.096 (15)	0.062 (13)	0.025 (12)	0.018 (11)	0.017 (9)
O2	0.051 (6)	0.066 (7)	0.064 (7)	-0.002 (5)	0.006 (5)	-0.015 (6)
O3	0.084 (8)	0.058 (8)	0.065 (7)	-0.002 (6)	-0.007 (6)	-0.004 (6)
O4	0.076 (7)	0.054 (7)	0.069 (7)	-0.003 (6)	0.026 (6)	0.001 (5)
O6	0.045 (6)	0.058 (7)	0.090 (8)	0.007 (6)	0.007 (5)	0.014 (6)
O60	0.083 (8)	0.102 (9)	0.074 (8)	0.008 (8)	0.020 (6)	0.010 (7)
S1	0.078 (3)	0.052 (3)	0.063 (3)	-0.006 (3)	0.007 (2)	-0.003 (3)

*Geometric parameters (Å, º) for compound **83***

C1—O6	1.391 (17)	C24—H24	0.95
C1—C2	1.500 (19)	C25—C26	1.37 (3)
C1—S1	1.791 (15)	C25—H25	0.95
C1—H1	1.0	C26—H26	0.95
C2—O2	1.404 (17)	C30—O3	1.45 (2)
C2—C3	1.50 (2)	C30—C31	1.49 (3)
C2—H2	1.0	C30—H30A	0.99
C3—O3	1.409 (17)	C30—H30B	0.99
C3—C4	1.51 (2)	C31—C36	1.29 (3)
C3—H3	1.0	C31—C32	1.33 (3)
C4—O4	1.44 (2)	C32—C33	1.44 (4)
C4—C5	1.52 (2)	C32—H32	0.95
C4—H4	1.0	C33—C34	1.25 (4)
C5—O6	1.44 (2)	C33—H33	0.95
C5—C6	1.48 (2)	C34—C35	1.22 (4)
C5—H5	1.0	C34—H34	0.95
C6—O60	1.42 (2)	C35—C36	1.39 (3)
C6—H6A	0.99	C35—H35	0.95

C6—H6AB	0.99	C36—H36	0.95
C11—C12	1.32 (2)	C40—O60	1.40 (2)
C11—C16	1.43 (2)	C40—O4	1.449 (19)
C11—S1	1.779 (16)	C40—C42	1.51 (3)
C12—C13	1.43 (2)	C40—H40	1.0
C12—H12	0.95	C41—C42	1.34 (3)
C13—C14	1.38 (2)	C41—C48A	1.46 (3)
C13—H13	0.95	C41—H41	0.95
C14—C15	1.40 (2)	C42—C43	1.32 (3)
C14—H14	0.95	C43—C44	1.34 (4)
C15—C16	1.39 (2)	C43—H43	0.95
C15—H15	0.95	C44—C44A	1.48 (4)
C16—H16	0.95	C44—H44	0.95
C20—O2	1.416 (19)	C45—C46	1.37 (4)
C20—C21	1.51 (2)	C45—C44A	1.47 (4)
C20—H20A	0.99	C45—H45	0.95
C20—H20B	0.99	C44A—C48A	1.32 (3)
C21—C26	1.36 (3)	C46—C47	1.33 (4)
C21—C22	1.40 (3)	C46—H46	0.95
C22—C23	1.39 (3)	C47—C48	1.31 (3)
C22—H22	0.95	C47—H47	0.95
C23—C24	1.34 (2)	C48—C48A	1.37 (3)
C23—H23	0.95	C48—H48	0.95
C24—C25	1.34 (3)		
O6—C1—C2	113.2 (12)	C24—C25—C26	120.3 (19)
O6—C1—S1	110.0 (10)	C24—C25—H25	119.9

C2—C1—S1	108.4 (10)	C26—C25—H25	119.8
O6—C1—H1	108.4	C21—C26—C25	121 (2)
C2—C1—H1	108.4	C21—C26—H26	119.4
S1—C1—H1	108.4	C25—C26—H26	119.4
O2—C2—C3	110.8 (12)	O3—C30—C31	110.3 (15)
O2—C2—C1	108.7 (12)	O3—C30—H30A	109.6
C3—C2—C1	113.1 (13)	C31—C30—H30A	109.6
O2—C2—H2	108.0	O3—C30—H30B	109.6
C3—C2—H2	108.0	C31—C30—H30B	109.6
C1—C2—H2	108.0	H30A—C30—H30B	108.1
O3—C3—C2	107.1 (14)	C36—C31—C32	117 (2)
O3—C3—C4	113.2 (12)	C36—C31—C30	120 (2)
C2—C3—C4	112.9 (13)	C32—C31—C30	123 (2)
O3—C3—H3	107.8	C31—C32—C33	117 (3)
C2—C3—H3	107.8	C31—C32—H32	121.3
C4—C3—H3	107.8	C33—C32—H32	121.3
O4—C4—C3	108.1 (14)	C34—C33—C32	123 (3)
O4—C4—C5	111.0 (14)	C34—C33—H33	118.7
C3—C4—C5	110.4 (12)	C32—C33—H33	118.7
O4—C4—H4	109.1	C35—C34—C33	118 (4)
C3—C4—H4	109.1	C35—C34—H34	121.0
C5—C4—H4	109.1	C33—C34—H34	121.0
O6—C5—C6	106.4 (15)	C34—C35—C36	124 (3)
O6—C5—C4	108.5 (14)	C34—C35—H35	117.8
C6—C5—C4	107.0 (14)	C36—C35—H35	117.8
O6—C5—H5	111.6	C31—C36—C35	120 (3)
C6—C5—H5	111.6	C31—C36—H36	119.8

C4—C5—H5	111.6	C35—C36—H36	119.8
O60—C6—C5	114.7 (16)	O60—C40—O4	110.7 (14)
O60—C6—H6A	108.6	O60—C40—C42	109.7 (17)
C5—C6—H6A	108.6	O4—C40—C42	109.9 (17)
O60—C6—H6AB	108.6	O60—C40—H40	108.8
C5—C6—H6AB	108.6	O4—C40—H40	108.8
H6A—C6—H6AB	107.6	C42—C40—H40	108.8
C12—C11—C16	122.3 (15)	C42—C41—C48A	119 (2)
C12—C11—S1	115.7 (13)	C42—C41—H41	120.4
C16—C11—S1	121.5 (13)	C48A—C41—H41	120.4
C11—C12—C13	120.3 (16)	C43—C42—C41	119 (2)
C11—C12—H12	119.8	C43—C42—C40	120 (2)
C13—C12—H12	119.8	C41—C42—C40	120 (2)
C14—C13—C12	118.6 (17)	C42—C43—C44	127 (3)
C14—C13—H13	120.7	C42—C43—H43	116.5
C12—C13—H13	120.7	C44—C43—H43	116.5
C13—C14—C15	120.3 (18)	C43—C44—C44A	115 (3)
C13—C14—H14	119.8	C43—C44—H44	122.5
C15—C14—H14	119.8	C44A—C44—H44	122.5
C16—C15—C14	121.0 (17)	C46—C45—C44A	117 (3)
C16—C15—H15	119.5	C46—C45—H45	121.7
C14—C15—H15	119.5	C44A—C45—H45	121.7
C15—C16—C11	116.9 (15)	C48A—C44A—C45	122 (3)
C15—C16—H16	121.5	C48A—C44A—C44	119 (3)
C11—C16—H16	121.5	C45—C44A—C44	118 (3)
O2—C20—C21	107.4 (14)	C47—C46—C45	117 (3)
O2—C20—H20A	110.2	C47—C46—H46	121.5

C21—C20—H20A	110.2	C45—C46—H46	121.4
O2—C20—H20B	110.2	C48—C47—C46	126 (4)
C21—C20—H20B	110.2	C48—C47—H47	116.9
H20A—C20—H20B	108.5	C46—C47—H47	116.9
C26—C21—C22	117.9 (19)	C47—C48—C48A	120 (3)
C26—C21—C20	121 (2)	C47—C48—H48	119.8
C22—C21—C20	120.6 (19)	C48A—C48—H48	119.8
C23—C22—C21	119.6 (19)	C44A—C48A—C48	117 (3)
C23—C22—H22	120.2	C44A—C48A—C41	120 (3)
C21—C22—H22	120.2	C48—C48A—C41	123 (3)
C24—C23—C22	120 (2)	C2—O2—C20	117.6 (12)
C24—C23—H23	119.8	C3—O3—C30	115.0 (13)
C22—C23—H23	119.8	C4—O4—C40	109.3 (13)
C23—C24—C25	121 (2)	C1—O6—C5	113.5 (11)
C23—C24—H24	119.7	C40—O60—C6	109.5 (15)
C25—C24—H24	119.7	C11—S1—C1	106.7 (8)
O6—C1—C2—O2	-169.9 (11)	C48A—C41—C42—C43	2 (3)
S1—C1—C2—O2	67.8 (13)	C48A—C41—C42—C40	178.2 (17)
O6—C1—C2—C3	-46.4 (18)	O60—C40—C42—C43	-36 (3)
S1—C1—C2—C3	-168.7 (11)	O4—C40—C42—C43	-158 (2)
O2—C2—C3—O3	-69.9 (15)	O60—C40—C42—C41	147.5 (19)
C1—C2—C3—O3	167.8 (13)	O4—C40—C42—C41	26 (3)
O2—C2—C3—C4	164.8 (13)	C41—C42—C43—C44	-1 (4)
C1—C2—C3—C4	42.5 (19)	C40—C42—C43—C44	-177 (2)
O3—C3—C4—O4	-48.7 (19)	C42—C43—C44—C44A	1 (4)
C2—C3—C4—O4	73.1 (16)	C46—C45—C44A—C48A	3 (4)

O3—C3—C4—C5	-170.3 (15)	C46—C45—C44A—C44	177 (3)
C2—C3—C4—C5	-48 (2)	C43—C44—C44A—C48A	-3 (4)
O4—C4—C5—O6	-62.7 (16)	C43—C44—C44A—C45	-178 (2)
C3—C4—C5—O6	57.2 (19)	C44A—C45—C46—C47	-1 (4)
O4—C4—C5—C6	52 (2)	C45—C46—C47—C48	0 (4)
C3—C4—C5—C6	171.6 (15)	C46—C47—C48—C48A	-2 (4)
O6—C5—C6—O60	63.9 (18)	C45—C44A—C48A—C48	-4 (3)
C4—C5—C6—O60	-52 (2)	C44—C44A—C48A—C48	-179 (2)
C16—C11—C12—C13	8 (3)	C45—C44A—C48A—C41	178.5 (19)
S1—C11—C12—C13	-179.5 (14)	C44—C44A—C48A—C41	4 (4)
C11—C12—C13—C14	-6 (3)	C47—C48—C48A—C44A	4 (4)
C12—C13—C14—C15	4 (3)	C47—C48—C48A—C41	-179 (2)
C13—C14—C15—C16	-4 (3)	C42—C41—C48A—C44A	-3 (3)
C14—C15—C16—C11	5 (2)	C42—C41—C48A—C48	180 (2)
C12—C11—C16—C15	-8 (2)	C3—C2—O2—C20	96.5 (17)
S1—C11—C16—C15	-179.5 (13)	C1—C2—O2—C20	-138.7 (15)
O2—C20—C21—C26	92 (2)	C21—C20—O2—C2	166.5 (15)
O2—C20—C21—C22	-89 (2)	C2—C3—O3—C30	157.5 (14)
C26—C21—C22—C23	-1 (3)	C4—C3—O3—C30	-77.5 (19)
C20—C21—C22—C23	-179.7 (17)	C31—C30—O3—C3	160.8 (15)
C21—C22—C23—C24	1 (3)	C3—C4—O4—C40	-179.5 (13)
C22—C23—C24—C25	-1 (3)	C5—C4—O4—C40	-58.3 (17)
C23—C24—C25—C26	1 (3)	O60—C40—O4—C4	63.4 (19)
C22—C21—C26—C25	0 (3)	C42—C40—O4—C4	-175.3 (14)
C20—C21—C26—C25	179.4 (19)	C2—C1—O6—C5	58.2 (16)
C24—C25—C26—C21	0 (3)	S1—C1—O6—C5	179.6 (10)
O3—C30—C31—C36	-38 (3)	C6—C5—O6—C1	-178.3 (13)

O3—C30—C31—C32	149 (2)	C4—C5—O6—C1	-63.4 (17)
C36—C31—C32—C33	3 (5)	O4—C40—O60—C6	-62 (2)
C30—C31—C32—C33	176 (3)	C42—C40—O60—C6	176.9 (14)
C31—C32—C33—C34	-4 (6)	C5—C6—O60—C40	58 (2)
C32—C33—C34—C35	4 (6)	C12—C11—S1—C1	177.7 (14)
C33—C34—C35—C36	-2 (6)	C16—C11—S1—C1	-10.1 (15)
C32—C31—C36—C35	-1 (5)	O6—C1—S1—C11	72.2 (12)
C30—C31—C36—C35	-175 (3)	C2—C1—S1—C11	-163.6 (11)
C34—C35—C36—C31	1 (6)		
